# Diversity of Bacterial Secondary Metabolite Biosynthetic Gene Clusters in Three Vietnamese Sponges

**DOI:** 10.3390/md21010029

**Published:** 2022-12-29

**Authors:** Ton That Huu Dat, Georg Steinert, Nguyen Thi Kim Cuc, Pham Viet Cuong, Hauke Smidt, Detmer Sipkema

**Affiliations:** 1 Mientrung Institute for Scientific Research, Vietnam National Museum of Nature, Vietnam Academy of Science and Technology (VAST), 321 Huynh Thuc Khang, Hue City 531600, Vietnam; 2 Laboratory of Microbiology, Wageningen University & Research, Stippeneng 4, 6708 WE Wageningen, The Netherlands; 3 Institute of Marine Biochemistry, Vietnam Academy of Science and Technology (VAST), 18 Hoang Quoc Viet, Cau Giay, Hanoi 10072, Vietnam

**Keywords:** secondary metabolite, biosynthetic gene cluster, metagenome, sponge-associated bacteria, Porifera

## Abstract

Recent reviews have reinforced sponge-associated bacteria as a valuable source of structurally diverse secondary metabolites with potent biological properties, which makes these microbial communities promising sources of new drug candidates. However, the overall diversity of secondary metabolite biosynthetic potential present in bacteria is difficult to access due to the fact that the majority of bacteria are not readily cultured in the laboratory. Thus, use of cultivation-independent approaches may allow accessing “silent” and “cryptic” secondary metabolite biosynthetic gene clusters present in bacteria that cannot yet be cultured. In the present study, we investigated the diversity of secondary metabolite biosynthetic gene clusters (BGCs) in metagenomes of bacterial communities associated with three sponge species: *Clathria reinwardti*, *Rhabdastrella globostellata*, and *Spheciospongia* sp. The results reveal that the three metagenomes contain a high number of predicted BGCs, ranging from 282 to 463 BGCs per metagenome. The types of BGCs were diverse and represented 12 different cluster types. Clusters predicted to encode fatty acid synthases and polyketide synthases (PKS) were the most dominant BGC types, followed by clusters encoding synthesis of terpenes and bacteriocins. Based on BGC sequence similarity analysis, 363 gene cluster families (GCFs) were identified. Interestingly, no GCFs were assigned to pathways responsible for the production of known compounds, implying that the clusters detected might be responsible for production of several novel compounds. The KS gene sequences from PKS clusters were used to predict the taxonomic origin of the clusters involved. The KS sequences were related to 12 bacterial phyla with *Actinobacteria*, *Proteobacteria*, and *Firmicutes* as the most predominant. At the genus level, the KSs were most related to those found in the genera *Mycolicibacterium*, *Mycobacterium*, *Burkholderia*, and *Streptomyces*. Phylogenetic analysis of KS sequences resulted in detection of two known ‘sponge-specific’ BGCs, i.e., *SupA* and *SwfA,* as well as a new ‘sponge-specific’ cluster related to fatty acid synthesis in the phylum *Candidatus* Poribacteria and composed only by KS sequences of the three sponge-associated bacterial communities assessed here.

## 1. Introduction

Bacterial secondary metabolites, also referred to as natural products or specialized metabolites, are an important source of biologically-active compounds for healthcare and agriculture applications [1,2,3,4]. These valuable secondary metabolites represent a group of low-molecular-weight structurally diverse compounds including polyketides (PKs), non-ribosomal peptides (NRPs), ribosomally synthesized and post-translationally modified peptides (RiPPs), terpenoids, saccharides, and a plethora of hybrids. The genes responsible for the biosynthesis of secondary metabolites are physically co-located in a biosynthetic gene cluster (BGC) with a size from a few to more than 100 kb [5,6].

In bacteria, non-ribosomal peptide synthase (NRPS) and polyketide synthase (PKS) gene clusters are the two main clusters responsible for the biosynthesis of secondary metabolites [7]. Polyketides are typically produced by PKS assembly lines that are composed of different functional modules. Each module is responsible for the incorporation and tailoring of a two-carbon unit into the final pathway product [8]. The minimal domain of a PKS module consists of an acyltransferase (AT), which selects and loads coenzyme-A (CoA) thioester-derived extender units; an acyl carrier protein (ACP), which functions as site for the covalent tethering of both the growing product chain and AT-selected extender units; and a ketosynthase (KS) domain, required for catalysis of carbon–carbon bond formation between the downstream product chain and the upstream extender unit [9]. Non-ribosomal peptide synthetases (NRPS) also form multienzyme assembly lines that are similar to those of PKS and produce peptide-containing compounds. In the NRPS module, an adenylation (A) domain activates an amino acid, and loads it onto a peptidyl carrier protein (PCP). Subsequently, a condensation (C) domain generates an amide bond with the growing chain tethered to the upstream PCP domain [10]. Interestingly, many bacteria have been found to possess the hybrid NRPS-PKS enzymes [11,12,13,14,15] and show that both PKS and NRPS function simultaneously in the same assembly line, but the products synthesized by most of the hybrid PKS/NRPS enzymes are still unknown. With the advances in metagenome sequencing, numerous uncharacterized BGCs have been found in uncultivated bacteria, which are regarded as an untapped source for the discovery of new drugs [16,17,18,19].

Marine sponges are well known to be a particularly rich source of structurally unique secondary metabolites with a broad spectrum of biological properties [20,21,22], many of which are probably produced by their associated bacteria. For example, it is now known that polybrominated biphenyl ether antibiotics isolated from the sponge *Dysidea herbacea* are actually produced by the endosymbiotic cyanobacterium *Oscillatoria spongeliae* [23]. In addition, the antifungal peptide theopalauamide isolated from the marine sponge *Theonella swinhoei* has been found to be contained in a δ-proteobacterial symbiont [24]. Subsequent metagenomic analysis of sponge microbiomes revealed the bacterial origin of many polyketide and modified peptide secondary metabolites [17,25,26,27,28,29,30,31,32,33]. Although the chemical role of many symbiont-derived compounds in the sponge holobionts remains unclear, accumulated evidence shows that symbiont-derived secondary metabolites play a role in chemical defense against predators, biofouling, and competition for space [25,26,28,34,35,36]. These biological properties make these sponge-associated microbial communities promising sources of new drug candidates [37,38,39,40]. However, the overall diversity of secondary metabolite biosynthetic potential present in sponge-associated bacteria is difficult to access because the large majority of these bacteria are not readily cultured in the laboratory environment [41]. Thus, the use of cultivation-independent approaches (e.g., metagenomics, cloning, heterologous expression) for the investigation of the chemistry of sponge symbiotic bacteria may allow access to secondary metabolite biosynthetic gene clusters of the currently uncultivable symbiotic bacteria of sponges.

We have previously investigated the bacterial and archaeal diversity of the 18 marine sponge species and found that three species in particular *Spheciospongia* sp., *Rhabdastrella globostellata* and *Clathria reinwardti* harbored diverse communities of bacteria [42]. Furthermore, recent chemical investigations have revealed diverse natural products derived from these sponge species, i.e., *C. reinwardti* [43,44,45], *R. globostellata* [46,47,48,49,50,51,52,53], and *Spheciospongia* spp. [53,54,55,56,57]. However, it has remained unclear whether/which sponge-associated bacteria are responsible for the production of these natural products. Here, we investigate the diversity of secondary metabolite biosynthetic gene clusters in metagenomes of the bacterial communities associated with these three sponge species.

## 2. Results

### 2.1. Metagenomes of the Sponge-Associated Bacteria

The metagenomes of bacteria associated with the three sponge species *Spheciospongia* sp., *Rhabdastrella globostellata,* and *Clathria reinwardti* were sequenced. The numbers of paired-end reads obtained from the samples were in a range of 30,414,932 to 66,835,559 with average G+C content between 61% and 62 % (Table S1). Quality filtering of the reads resulted in a range of 27,783,369 to 62,722,556 high-quality reads that were used in downstream analyses. Assembling reads per sample generated 607,208, 466,098, and 504,262 contigs for the metagenomes of *C. reinwardti*, *R. globostellata*, and *Spheciospongia* sp., respectively. The N50 values of the contigs ranged from 21,342 to 56,925 and the number of protein-coding sequences ranged from 782,060 to 903,985 for the three samples.

### 2.2. Diversity of Secondary Metabolite Biosynthetic Gene Clusters from the Metagenomes of the Sponge-Associated Bacteria

For the prediction of secondary metabolite biosynthetic gene clusters (BGCs) from the metagenomes from sponge-associated bacteria, only contigs longer than 1000 bp were analyzed. These yielded a total of 1116 BGCs as identified by antiSMASH (Tables S2–S4). The number of BGCs identified per metagenome ranged from 282 to 463 (Table 1). Diverse types of BGCs were observed with 12 different cluster types. Cf fatty acid and type I PKS were the most dominant BGC types with 86–164 and 83–125 clusters per sample, respectively, followed by terpenes (58–97 clusters per sample), and bacteriocins (12–16 clusters per sample), whereas the remaining cluster types only accounted for less than 11 clusters per sample. Surprisingly, not a single NRPS cluster was detected from metagenomes of bacteria associated with three sponge species and interestingly, the use of the *KnownClusterBlast* function in antiSMASH suggested that none the identified BGCs was encoding for biosynthetic pathways of known compounds.

**Figure 1:**
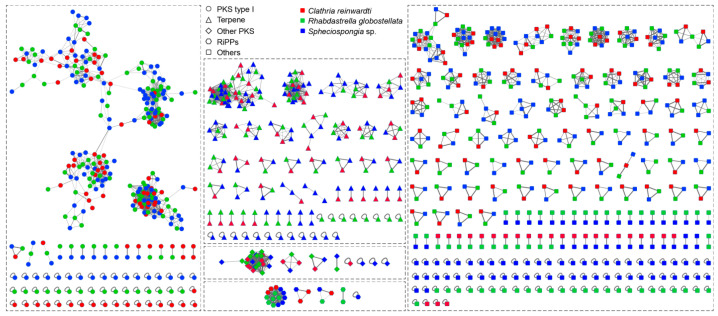
BGC sequence similarity network of the BGCs identified from the metagenomes of the sponge-associated bacteria. Each node represents one BGC identified by antiSMASH.

To identify orthologous clusters among BGCs across the different sponge metagenomes, we reconstructed a BGC sequence similarity network (Figure 1). The analysis of BGC sequence similarity showed that the connected components corresponded to 363 gene cluster families (GCFs), including 86 GCFs of type I PKSs, 5 GCFs of other PKSs, 63 GCFs of terpenes, 8 GCFs of ribosomally synthesized and post-translationally modified peptides (RiPPs), and 201 GCFs of other BGCs, including cf fatty acid (Figure 1). Several larger networks were composed of multiple GCFs because these networks could not be resolved into single GCF networks using default similarity cutoffs.

**Figure 2:**
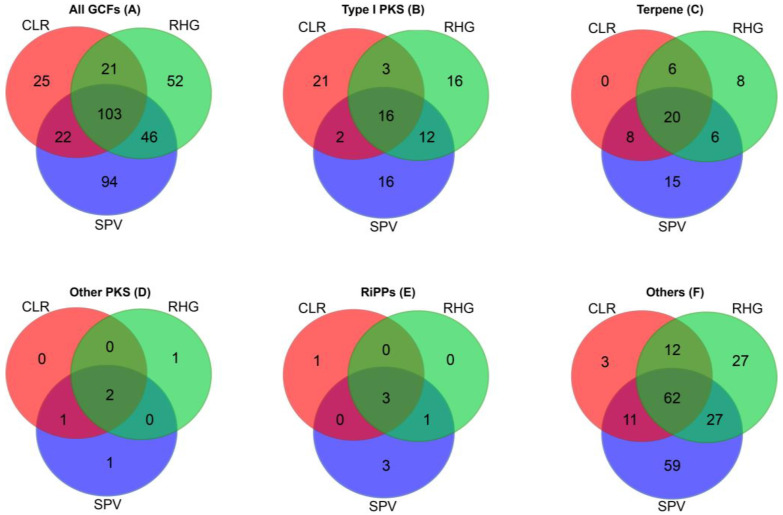
Venn diagrams of the identified GCFs from the metagenomes of three sponge-associated bacteria. CLR, *Clathria reinwardti*; RHG, *Rhabdastrella globostellata*; and SPV, *Spheciospongia* sp.

In general, approximately one-third (103) of GCFs were found in all specimens, 89 GCFs in 2 specimens, and 171 GCFs only in individual specimens (singletons) (Figure 2A). It appears that Type I PKSs are more often unique to a sponge species/sample, while terpenes, other PKS, and RiPPs are more often shared between species/samples (Figure 2B–E). Comparison of the BGCs identified with BGCs that encode known compounds in the MIBiG (Minimum Information about a Biosynthetic Gene cluster) reference database [58] showed that no GCFs from the metagenomes were assigned to pathways responsible for the production of known compounds (Tables S2–S4).

### 2.3. Ketosynthase Phylogeny of BGCs

**Figure 3:**
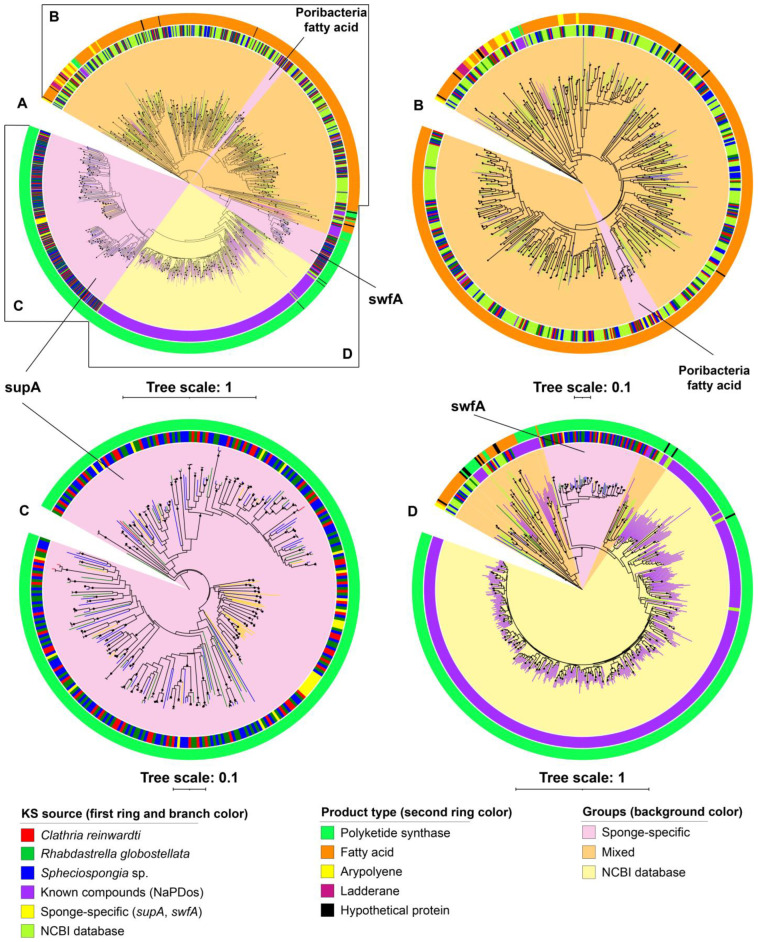
The phylogeny of the KS sequences from the identified BGCs from sponge-associated bacterial metagenomes including KS sequences from the NaPDoS database and NCBI GenBank (**A**) and its subtrees (**B**–**D**).

The ketosynthase (KS) domain is the most conserved catalytic domain of the PKS gene cluster and involved in the tailoring the polyketide by catalyzing chain condensation. Therefore, the KS sequences extracted from the identified BGCs were used for phylogenetic analysis (Figure 3). The KS sequences had low identity (21–66%) to the KS sequences of the characterized pathways from the NaPDos database (Tables S5–S7) as could be expected as BGCs from our study did not encode known pathways. In addition, many KS sequences of the identified BGCs from the metagenomes showed low identities (30–70%) to KS sequences from NCBI GenBank (Tables S8–S13).

Phylogenetic analysis of KS sequences showed that most of the KS sequences of the cf_fatty acid BGCs identified from the sponge bacteria metagenomes clustered together with previously found KS sequences from fatty acid biosynthesis gene clusters retrieved from NCBI GenBank (Figure 3B). Several KS sequences from fatty acid BGCs predicted from the sponge-associated bacteria metagenomes had KS sequences from fatty acid BGCs from *Candidatus* Poribacteria metagenome-assembled genomes as best hit and formed a phylogenetically distant clade from other KS sequences from fatty acid BGCs derived from other sources (Figure 3B). KS sequences from the aryl polyene and ladderane BGCs identified from the metagenomes formed subclusters within clades of KS sequences belonging to fatty acid synthesis pathways retrieved from NCBI Genbank. For PKS BGCs obtained from the sponge-associated bacteria metagenomes in the present study, only a few KS sequences were placed in the same clades as reference non-sponge KS sequences retrieved from NCBI GenBank and NaPDos, and the large majority of KS sequences had best hits with sponge-derived KS sequences and formed two separate clades that were phylogenetically distant to non-sponge-derived KS sequences (supA and swfA) (Figure 3C,D).

**Figure 4:**
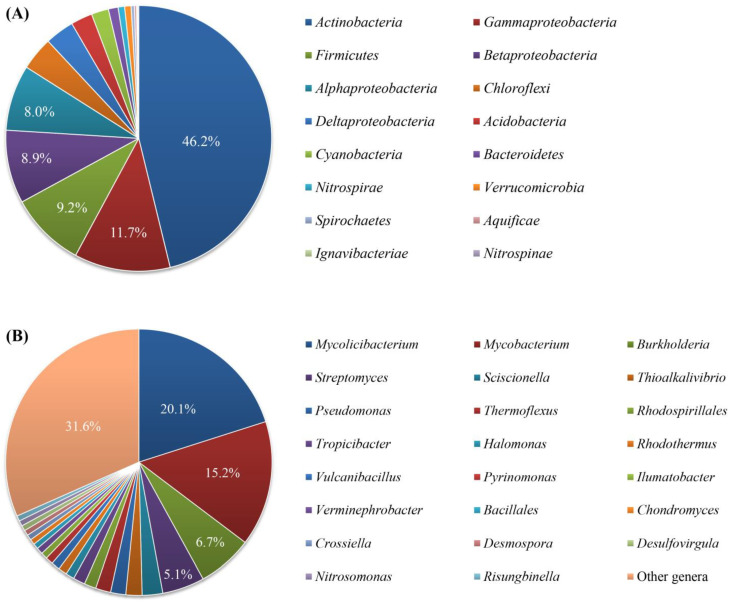
Taxonomy of the bacteria harboring KS sequences most identical to KS sequences (the best hit) identified from the metagenomes of the sponge-associated bacteria at (**A**) the phylum (or class level for *Proteobacteria*) and (**B**) genus level.

In addition, we used the BLASTX searches of the KS sequences to predict the bacterial taxa harboring these sequences and they were predicted to be related to KS sequences from diverse bacteria represented by 12 phyla, including *Actinomycetes*, *Proteobacteria*, *Firmicutes*, *Chloroflexi*, *Acidobacteria*, *Cyanobacteria*, *Bacteroidetes*, *Nitrospirae*, *Verrucomicrobia*, *Spirochaetes*, *Aquificae*, *Nitrospinae,* and *Ignavibacteriae* (Figure 4A, Tables S11–S13). Among them, *Actinobacteria* was the most predominant phylum (46.2% of the KS sequences), followed by the classes of *Proteobacteria* (3.5–11.7%) and the phylum *Firmicutes* (9.1%). Each of remaining phyla accounted for less than 5%. At the genus level, the KS sequences of the identified BGCs from the metagenomes were linked to 142 genera (Figure 4B). Of these, the genera *Mycolicibacterium*, *Mycobacterium*, *Burkholderia*, and *Streptomyces* were most dominant and accounted for 20.1, 15.2, 6.7, and 5.1% of the total KS sequences, respectively.

Except for the KS sequences retrieved from fatty acid BGCs, which were found from a wide range of bacterial phyla (Figure S1), KS sequences from other BGC types were non-uniformly distributed across sponge-associated bacterial taxa (Figures S2–S4). The KS sequences retrieved from PKS BGCs were predominantly related to those from *Actinobacteria* (80% of the PKS KS sequences), particularly the genera *Mycolicibacterium* (37%), *Mycobacterium* (28%), *Burkholderia* (12%), *Streptomyces* (7%), and *Sciscionella* (5%) (Figure S2). On the other hand, the KS sequences from aryl propylene BGCs were predominantly related to those from *Proteobacteria* (> 80% of the sequences), including *Betaproteobacteria* (45%), *Gammaproteobacteria* (26%), and *Deltaproteobacteria* (11%) with *Verminephrobacter*, *Cellvibrio*, *Syntrophus*, *Cupriavidus*, *Lechevalieria,* and *Thiobacillus* as main genera (Figure S3). In case of ladderane BGCs, the KS sequences were mainly related to only two classes of *Proteobacteria* (*Alpha*- and *Delta*-) and three genera *Tropicibacter*, *Sandaracinus,* and *Pseudovibrio* (Figure S4).

## 3. Discussion

### 3.1. Diversity of Secondary Metabolite Biosynthetic Gene Clusters from the Metagenomes of Sponge-Associated Bacteria

Gene clusters related to putative fatty acid synthesis is the most predominant BGC type identified from the metagenomes of sponge-associated bacteria in our study. This is expected because fatty acids are essential components in cell membranes of bacteria. Of particular interest is the detection of a separate cluster of KS genes related to fatty acid synthase from *Candidatus* Poribacteria, a sponge-specific bacterial candidate phylum. Members of the phylum *Candidatus* Poribacteria were not detected in the same sponge specimens by 16S rRNA amplicon sequencing [42]. However, this discrepancy between 16S rRNA sequencing and metagenomics has frequently been observed and the lack of *Candidatus* Poribacteria in the PCR amplicon-based study may be attributed to a bias of the specificity of primers [59]. Except for central roles in the integrity of cell membranes and energy storage [60], many fatty acids have been recently suggested to be potential antimicrobial, antifouling, and antivirulence agents [61,62,63]. Several reports have also shown fatty acids to enhance the antibacterial activities of antibiotics [64,65,66,67,68].

PKSs are among the most dominant BGC types in the metagenomes of sponge-associated bacteria. Of these, PKS type I is most dominant, while only a few PKS type II and type III clusters were detected in the metagenomes. Almost all type I PKSs detected in the metagenomes of sponge-associated bacteria in our study were the non-iterative type I PKSs. Previous investigations have revealed that the distribution of PKSs appears to be origin-dependent. The non-iterative type I PKSs are often found in bacteria, whereas iterative type I PKSs are mainly found in fungi [69,70,71]. Bacterial type I PKSs are particularly attractive targets for exploration and exploitation of their bioactive compounds because they are defined by a modular organization, remarkable versatility, and amenability for pathway engineering [72]. Several antibiotics derived from these multicatalytic enzymes include macrolides (erythromycin), polyenes (nystatin), as well as linear polyketides (tautomycetin) [73,74,75]. In our study type I PKSs from the sponge-associated bacteria metagenomes had a best BLAST hit to clusters encoding for the production of diverse bioactive compounds, such as puwainaphycins, ajudazol, polycyclic tetramate macrolactams, glycopeptidolipid, epothilone, phenalamide, crocacin, curacin, microsclerodermins, nostophycin, jamaicamide, chondrochloren, gephyronic acid, cylindrospermopsin, soraphen, amphotericin, reveromycin, stigmatellin, candicidin, myxalamid, gulmirecin, heat-stable antifungal factor, myxothiazol, tautomycin, and ECO-02301 (a new class of antifungal agent) (Tables S14–S16). Notably, several *trans*-AT PKSs were also detected in the sponge-associated bacteria metagenomes. *Trans*-AT PKSs lack integrated AT domains. Instead, a free-standing AT domain acts in *trans* to load acyl building blocks into the assembly line [76]. Nearly 40% of all bacterial modular PKSs belong to the *trans*-AT type, suggesting that the polyketides of these enzymes constitute a major natural product class [77]. Indeed, several drugs which are used as antibiotics were identified as *trans*-AT PKS products (e.g., mupirocin, streptogramins) [78,79]. Many compounds from sponges and their symbionts are known to be biosynthesized by *trans*-AT PKS pathways (e.g., diffusomycin, psymberin, onnamide A, calyculin A, swinhoeiamide A, swinhodile A, clavosine A, geoetricin A, misakinolide A, oocydin A, and mycalamide A) [80,81,82]. The *trans*-AT PKS gene clusters detected in the sponge-associated bacteria metagenomes in our study had a best BLAST hit to gene clusters involved in the production of puwainaphycins and epothilones (Tables S14–S16). However, none of the clusters in our dataset could be reliably assigned to a cluster encoding for the production of a known compounds (MiBIG database). These findings imply that the type I PKSs detected in our study are likely involved in the biosynthesis of currently unknown compounds.

Type II PKSs are found exclusively in bacteria and are responsible for producing aromatic polyketides such as the antibiotics actinorhodin, tetracycline or doxorubicin [83]. Although none of the type II PKSs detected in the metagenomes of sponge-associated bacteria in our study could be confidently assigned to gene clusters encoding known compounds, some BGCs had best BLAST hits related to clusters encoding compounds belonging to the class angucyclines (lomaiviticin) and benzoisochromanequinones (frenolicin) (Tables S14–S16). Type III PKSs are relatively small proteins that are mainly involved in the production of important plant compounds such as flavonoids and stilbenes (phenolic), but they are also found in bacteria and fungi [84]. All type III PKSs detected in the metagenomes of sponge-associated bacteria in our study had the cluster encoding for the proteins producing the alkylresorcinols as best BLAST hit (Tables S14–S16). Alkylresorcinols are synthesized by plants, fungi, and bacteria and have diverse biological activities including antioxidant, antibacterial, cytotoxic, and signaling properties [85,86,87].

Terpene BGCs were the third most predominant among BGCs predicted from the three metagenomes. Originally, terpenes were considered to be plant and fungal products. However, extensive bacterial genome analysis has shown that terpene synthase genes are also widespread in bacteria [88,89,90]. Recent genome analyses also detected BGCs of terpenes in genomes of sponge-associated bacteria [91,92,93], and several terpenes have been isolated from sponges and their associated symbionts [94,95]. Terpenes are among targets for drug development because they exhibit various biological properties, such as antimicrobial, anticancer, and antirespiratory activities [94]. Notably, the terpene gene clusters detected in the sponge-associated bacteria metagenomes in our study were, again, not closely related to gene clusters associated to known compounds. Best BLAST hits were clusters encoding the synthesis of diverse known terpenes, such as hopene, thalianol, lupeol, sioxanthin, tirucalla, brasilicardin A, clavaric acid, squalestatin, isorenieratene, arabidiol, baruol, and astaxanthin dideoxyglycoside (Tables S14–S16). Therefore, the terpene gene clusters are likely involved in the biosynthesis of similar, but still different terpenes.

Several RiPP BGC types were detected in the metagenome of sponge-associated bacteria in our study, including 12–16 bacteriocin BGCs and 1–3 lantipeptide BGCs per metagenome. Bacteriocins are ribosomally synthetized peptides or proteins that act as antimicrobial compounds against other Gram-positive and Gram-negative bacteria [96,97]. Genome analyses have detected BGCs of bacteriocins in sponge-associated bacteria [92,93,98]. Interestingly, Phelan, et al. [99] isolated a new bacteriocin (subtilomycin) from *Bacillus subtilis* MMA7 associated with the sponge *Haliclona simulans* and found that the subtilomycin biosynthetic cluster is widespread among *B. subtilis* strains isolated from different shallow and deep water marine sponges. However, the generally large discrepancy between bacteria isolated from sponges and the dominant bacteria present in sponges [41] is also translated to biosynthetic gene clusters present in isolates from sponges and those present in metagenomes from sponges. Indeed, no BGC related to subtilomycin biosynthesis was observed and, in fact, none of the bacteriocin gene clusters detected in the sponge-associated bacteria metagenomes was closely related to clusters encoding the proteins that catalyze biosynthesis of known bacteriocins. Best BLAST hits were obtained for clusters related to the production of bacteriocins, such as patellins, goadsporin, aeruginosamide, microcyclamide, trichamide, pheganomycin, patellamides, tenuecyclamides, and SRO15-3108. Lantipeptides are widely distributed among bacteria [100,101,102] and were previously found in genomes of sponge-associated bacteria [93,103,104,105,106]. A number of known lantipeptides have shown activity against microbial pathogens. Of these, several lantipeptides have been in pre-clinical trials as antibiotics for treatment of *Clostridium difficile* infections [107,108,109] and other Gram-positive bacterial infections, such as microbisporicin NAI-107, mutacin 1140, duramycin, NVB302, and lactacins 3147 α and β [110,111,112]. The best BLAST hits of lantipeptide BGCs were related to actagardine, SRO15-3108, and labyrinthopeptins biosynthesis but with only low identity scores.

Interestingly, no NRPS-like BGCs were detected in three sponge bacterial metagenomes in the present study. This may be attributed to several reasons. Firstly, an investigation of PKS and NRPS from microbes associated with *Discodermia dissoluta* from three metagenomic libraries (i.e., whole sponge tissue, and fractions enriched for unicellular or filamentous bacteria) revealed that NRPS sequences were only detected in the fraction enriched by filamentous bacteria, where no NRPS sequences were found in whole sponge tissue and the unicellular-enriched fraction [113]. During the enrichment for bacteria in our study prior to DNA extraction and metagenomic sequencing, most filamentous bacteria (if present) were probably lost and hence their BGCs not included in our dataset. In addition, some NRPS BGCs may be difficult to recover by next-generation sequencing approaches (e.g., from low-GC or high-AT species) due to biases of sequencing or assembly methods [114,115,116,117,118].

Previous chemical investigations have reported diverse natural products isolated from the three sponges *C. reinwardti* [43,44,45], *R. globostellata* [46,47,48,49,50,51,52], and *Spheciospongia* spp. [53,54,55,56,57]. The natural products from the reported sponge species and their associated cultivated bacteria included mainly terpenes and lipids and a few peptides, aromatic, phenolic, and indole compounds, which are products resulting from the main BGC classes (terpenes, PKSs, fatty acids, RiPPs) detected in sponge metagenomes in our study. The decadal challenge is now to link the striking novelty in sponge and other environmental BGCs to the natural products they produce.

### 3.2. Predicted Taxa of the Secondary Metabolite Biosynthetic Gene Clusters

Previous extensive investigations of BGCs in bacterial genomes revealed non-uniform distribution of BGCs in bacterial taxa. The phyla known to possess a high number of BGCs in their genomes include *Actinobacteria*, *Proteobacteria*, *Bacteroidetes*, *Firmicutes,* and *Cyanobacteria* [119]. Since the large majority of the BGCs in our sponge-derived metagenomes contained KS sequences (cf_fatty acids, PKSs, aryl polyenes and ladderanes), the KS sequences can be used to identify the major taxa that harbor BCGs in sponge-associated bacteria. Unsurprisingly, genera of the phylum *Actinobacteria*, such as *Mycolicibacterium*, *Mycobacterium*, and *Streptomyces,* were most dominant as predicted hosts of the KS sequences. Apart from *Streptomyces* and *Bacillus*, which are rich sources of natural products [120], other bacterial taxa are receiving increased attention as novel target genera with a high biosynthetic potential, including *Pseudomonas*, *Clostridium*, *Burkholderia*, *Pseudonocardia*, *Photorhabdus*, *Xenorhabdus*, *Chitinophaga*, *Herpetosiphon*, and *Planctomyces* [121,122]. Particularly in sponges, the genus “*Candidatus* Entotheonella” has been recognized for its unusually rich and varied arsenal of BGCs [33]. In our study, *Streptomyces*, *Pseudomonas,* and *Burkholderia* were among the prominent genera to which KS sequences were assigned, but “*Candidatus* Enthotheonella” was not identified, which corroborates with its absence in these three sponge species based on 16S rRNA gene analysis [42]. In addition, other genera, such as *Pseudovibrio* and *Bacillus,* that are frequently isolated from sponges and linked to a high prevalence of antimicrobial activity [39] were not identified here based on cultivation-independent means. 

Generally, the predicted taxa of bacteria harboring the BGCs in the present study corresponded to 16S rRNA gene analysis in a previous study at the phylum level, but their relative abundances are different [43]. This is likely due to different frequencies of BGCs among bacteria taxa [33,119].

### 3.3. Putative ‘Sponge-Specific’ Clusters

It is well known that marine sponges harbor specific microbial communities [123]. Interestingly, the KS sequence analysis also revealed the presence of several ‘sponge-specific’ PKS groups. In a systematic analysis of 150 KS sequences from metagenomic DNA from 20 different demosponges through PCR screenings of KS genes, Fieseler et al. [124] detected a cluster of sponge-specific PKS sequences, termed “symbiont ubiquitous type I PKS” (*Sup*). In another study, Sala et al. [125] detected another sponge-specific KS group, named “sponge (symbiont) widespread fatty acid synthase” (*Swf*), in several high-microbial-abundance sponges. Notably, both *SupA* and *SwfA* are involved in the synthesis of methyl branched fatty acids [125,126]. Phylogenetic reconstruction of KS sequences derived from the metagenomes of sponge-associated bacteria in our study showed three monophyletic sponge-specific clusters that were phylogenetically distant from KS sequences from other sources. Two sponge-specific KS groups were affiliated to the known sponge-specific KS groups, i.e., *SupA* and *SwfA*.

The third sponge-specific KS group is involved in fatty acids synthesis in *Candidatus* Poribacteria. This sponge-specific KS group includes KS sequences from our study and KS sequences retrieved from *Candidatus* Poribacteria metagenome-assembled genomes [59]. Borchert et al. [127] investigated the diversity of NRPS and PKS genes in microbiome of three deep-sea sponges *Inflatella pellicula*, *Poecillastra compressa*, and *Stelletta normani* through PCR screening of the KS domain and also found several sponge-specific KS groups. Additionally, other recent studies have identified other ‘sponge-specific’ gene clusters related to the biosynthesis of RIPP proteusins, bromotyrosine alkaloids, and ether lipids [19,32,128]. These findings imply that the sponge-specific specialized metabolites are prevalent and may play an important role in sponge holobiont defense or communication.

## 4. Materials and Methods

### 4.1. Collection and Identification of Sponges

Sponge specimens of *Spheciospongia* sp., *Rhabdastrella globostellata,* and *Clathria reinwardti* were collected by scuba diving from May to September 2015 from the central coastal region of Vietnam at 5–10 m depth and identified using molecular markers (18S rRNA and COI genes) in a previous study [42].

### 4.2. Enrichment of Sponge-Associated Bacteria

The specimens were rinsed three times with sterile artificial seawater to remove any debris attached to the sponge. Then the specimens were further cleaned with a sterile scalpel in order to remove sediment and other organisms more strongly attached to the sponges. Sponge-associated bacterial cells were enriched by stepwise centrifugation as described by [129]. In brief, the sponge tissue was cut into small pieces and ground in TEN buffer (3.5% sodium chloride, 10 mM tris-hydroxymethyl-aminomethane, 50 mM ethylenediaminetetraacetic acid, pH 8.5) with a sterilized mortar and pestle. The cell suspensions were then filtered through a large nylon mesh (20 µm) to further remove sediment particles and clumps of cells. Then the sponge cells and bacteria were separated by step-wise centrifugation. The cell suspension was first centrifuged at 500× *g* for 5 min at 4 °C. The supernatant was then transferred to another tube and centrifuged at 1000× *g* for 15 min at 4 °C. Next, the supernatant was transferred to another tube and centrifuged at 3000× *g* for 15 min at 4 °C. Finally, the supernatant was then transferred to another tube and centrifuged at 8000× *g* for 15 min at 4 °C. The pellet was washed twice with TEN buffer and centrifuged at 8000× *g* for 20 min at 4 °C. This cell pellet was used for metagenomic DNA extraction.

### 4.3. Metagenomic DNA Extraction, Sequencing and Assembly

Metagenomic DNA of sponge-associated bacteria was extracted using the ZymoBeadTM Genomic DNA Kit (Zymo Research, Irvine, CA, USA) according to the manufacturer’s protocol. The concentration of the extracted DNA was determined with a Nanodrop 1000 spectrophotometer (Nanodrop Technologies, Wilmington, DE, USA), and its integrity was examined by gel electrophoresis on a 1% (*w*/*v*) agarose gel. Metagenomic DNA was sequenced on an Illumina HiSeq2500 platform at BaseClear BV (Leiden, The Netherlands). Quality control of FASTQ files was done with FastQC v.0.11.7 (https://www.bioinformatics.babraham.ac.uk/projects/fastqc/, accessed on 25 January 2018). The raw reads were checked and the adapters, low-quality reads, artifacts, and PhiX contamination were removed using the command bbduk.sh of the BBmap v.34 (https://sourceforge.net/projects/bbmap/, accessed on 25 January 2018). Reads with a low Phred quality score (< 30) and reads shorter than 70 base pairs were filtered out. Next, reads were coverage-normalized with the command bbnorm.sh of BBMap v.34 at default settings. Reads were then assembled with SPAdes v.3.5.0 [130]. The non-normalized Illumina reads were mapped to the contigs with Bowtie2 v.2.2.2 at default settings [131]. The resulting SAM files were converted to BAM, sorted, and indexed with SAMtools v.0.1.18 [132]. HTSeq v.0.6.1 was used to calculate the average coverage of each contig [133]. Contigs longer than 1000 base pairs were used for further analysis. Open reading frames (ORFs) were called with Prodigal v.2.6.1 with -m and -p meta options enabled [134].

### 4.4. Prediction of Secondary Metabolite Gene Biosynthetic Clusters and Ketosynthase Phylogeny

The predicted protein-coding genes were subjected to a search for secondary metabolite biosynthetic gene clusters (BGCs) using antiSMASH v.4.1.0 (bacterial version). The KnownClusterBlast function in antiSMASH was enabled to identify BGCs that are responsible for the biosynthesis of known compounds in the metagenomes of the sponge-associated bacteria. The predicted biosynthetic gene clusters were also searched for known biosynthetic pathways using the natural product domain seeker (NaPdoS) (http://napdos.ucsd.edu/, accessed on 15 August 2018). Biosynthetic gene cluster similarity networks and gene cluster families were generated using BiG-SCAPE with default settings. The networks were visualized using Cytoscape v. 3.6.0 (https://cytoscape.org, accessed on 28 August 2018) [135].

For ketosynthase (KS) phylogeny, KS domain fragments of the biosynthetic gene clusters were extracted and trimmed using NaPdoS (http://napdos.ucsd.edu/, accessed on 15 August 2018) [136]. The KS sequences were used to search for homologous KS sequences in the NCBI Genbank database using *blastp* (http://www.ncbi.nlm.nih.gov/, accessed on 5 November 2020) against the *nr* database. KS sequences with a short length < 200 amino acids were excluded from the phylogenetic analysis. The KS sequences (from this study, their three most similar sequences, and sequences of known biosynthetic pathways from NaPdoS server) were aligned using the MAFFT (v.7.504) program with the FFT-NS-i strategy [137]. Poorly aligned positions and non-conserved regions were removed from the alignments using the trimAl v.1.2 [138]. The ketosynthase phylogeny was created using MEGA version 11.0.11 [139] with the neighbor-joining (NJ) method, the Poisson model and 1,000 bootstrap replicates. The phylogeny was visualized using iTOL v.5 [140] via the online server (https://itol.embl.de/, accessed on 23 April 2021). Taxonomy of the bacteria harboring the KS sequences were predicted based on the most homologous KS sequences (the first hit) by a BLAST search of the KS sequences against the *refseq_protein* database (v.2018) at NCBI using *blastp* (http://www.ncbi.nlm.nih.gov/, accessed on 5 May 2021).

## 5. Conclusions

The metagenomes of associated bacteria of three sponges contained a high number of predicted BGCs, ranging from 282 to 463 BGCs per metagenome, with 12 different BGC types. After cf_fatty_acid, PKSs were the most dominant BGCs, followed by terpene BGCs and bacteriocin BGCs. The BGCs were grouped into 363 gene cluster families (GCFs) based on sequence similarity. Interestingly, not a single GCF from the metagenomes was assigned to pathways responsible for the production of known compounds, implying that they might be responsible for production of novel compounds. Based on taxa of the closest reference KS sequences retrieved from the NCBI database, the KS sequences of the identified BGCs from the metagenomes related to 12 bacterial phyla, of which *Actinobacteria*, *Proteobacteria*, and *Firmicutes* were the most predominant. At the genus level, the KS were related to 142 genera, of which *Mycolicibacterium*, *Mycobacterium*, *Burkholderia,* and *Streptomyces* were the most predominant. Notably, a large number of KS sequences retrieved from PKS BGCs was present in two known ‘sponge-specific’ clusters, i.e., *SupA* and *SwfA*, whereas a part of KS sequences retrieved from fatty acid BGCs formed a new sponge-specific KS cluster related to the fatty acid synthesis in *Candidatus* Poribacteria. Our study reinforces that sponge-associated bacteria are a rich source of novel BGCs and as such most likely, bioactive compounds.

## Figures and Tables

**Table 1:** Secondary metabolite biosynthetic gene clusters identified in the metagenomes of the sponge-associated bacteria by antiSMASH.

Gene Cluster Type	*C. reinwardti*	*R. globostellata*	*Spheciospongia* sp.
Type I PKS	83	112	125
Type II PKS	1	1	1
Type III PKS	3	6	5
*Trans*-AT type I PKS	2	0	2
*Trans*-AT PKS	0	2	0
Terpene	58	66	97
Bacteriocin	12	16	14
Aryl polyene	8	10	9
Ladderane	3	4	2
Phosphonate	5	5	6
Lantipeptide	2	1	3
Cf_fatty_acid ^1^	86	119	164
Others	19	29	35
**Number of clusters**	**282**	**371**	**463**
**Number of contigs ≥ 1000**	**93,883**	**118,632**	**154,114**

^1^ Putative fatty acid cluster.

## Data Availability

Metagenome datasets have been deposited in the NCBI Sequence Read Archive under the BioProject accession number: PRJNA766414.

## Supplementary Materials

The following supporting information can be downloaded at: https://www.mdpi.com/article/10.3390/md21010029/s1, Figure S1: Taxonomy of the most homologous KS sequences with the KS sequences of cf_fatty acid BGCs identified from the metagenomes of the sponge-associated bacteria retrieved from NCBI Genbank at phylum/class level (A) and genus level (B); Figure S2: Taxonomy of the most homologous KS sequences with the KS sequences of PKS BGCs identified from the metagenomes of the sponge-associated bacteria retrieved from NCBI Genbank at phylum/class level (A) and genus level (B); Figure S3: Taxonomy of the most homologous KS sequences with the KS sequences of aryl propylene BGCs identified from the metagenomes of the sponge-associated bacteria retrieved from NCBI Genbank at phylum/class level (A) and genus level (B); Figure S4: Taxonomy of the most homologous KS sequences with the KS sequences of ladderane BGCs identified from the metagenomes of the sponge-associated bacteria retrieved from NCBI Genbank at phylum/class level (A) and genus level (B); Table S1: Basic data of the three metagenomes of sponge-associated bacteria; Table S2: BGCs identified from the metagenome of *C. reinwardti* by antiSMASH; Table S3: BGCs identified from the metagenome of *R. globostellata* by antiSMASH; Table S4: BGCs identified from the metagenome of *Spheciospongia* sp. by antiSMASH; Table S5: BLAST search of the KS sequences from the metagenome of *C. reinwardti* against the KS sequences of known products from the NaPDos database; Table S6: BLAST search of the KS sequences from the metagenome of *R. globostellata* against the KS sequences of known products from the NaPDos database; Table S7: BLAST search of the KS sequences from the metagenome of *Spheciospongia* sp. against the KS sequences of known products from the NaPDos database; Table S8: BLAST search of the KS sequences from the metagenome of *C. reinwardti* against the nr database in NCBI; Table S9: BLAST search of the KS sequences from the metagenome of *R. globostellata* against the nr database in NCBI; Table S10: BLAST search of the KS sequences from the metagenome of *Spheciospongia* sp. against the nr database in NCBI; Table S11: BLAST search of the KS sequences from the metagenome of *C. reinwardti* against the refseq_protein database in NCBI; Table S12: BLAST search of the KS sequences from the metagenome of *R. globostellata* against the refseq_protein database in NCBI; Table S13: BLAST search of the KS sequences from the metagenome of *Spheciospongia* sp. against refseq_protein database in NCBI; Table S14: BLAST search of the BGCs identified from the metagenome of *Clathria reinwardti* against the MiBIG database; Table S15: BLAST search of the BGCs identified from the metagenome of *Rhabdastrella globostellata* against the MiBIG database; Table S16: BLAST search of the BGCs identified from the metagenome of *Spheciospongia* sp. against the MiBIG database.
