# Supplementary material for: Diversity of Bacterial Secondary Metabolite Biosynthetic Gene Clusters in Three Vietnamese Sponges
Source: Mar Drugs. 2022 Dec 29;21(1):29. doi: 10.3390/md21010029 (PMC9864124; doi:10.3390/md21010029)
Supplement: Supplementary file 1 [file marinedrugs-21-00029-s001.zip › Figure S1-S4.pdf]

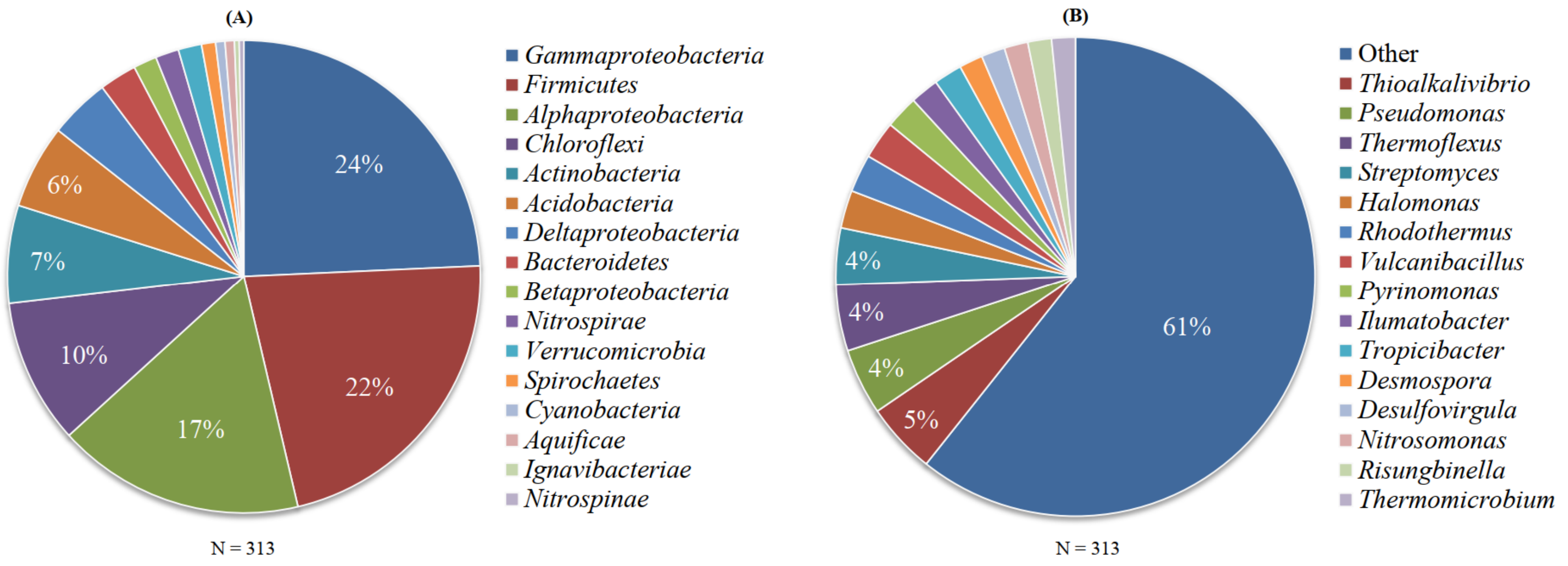

Figure S1. Taxonomy of the most homologous KS sequences with the KS sequences of cf_fatty acid BGCs identified from the metagenomes of the sponge-associated bacteria retrieved from NCBI Genbank at phylum/class level (A) and genus level (B)

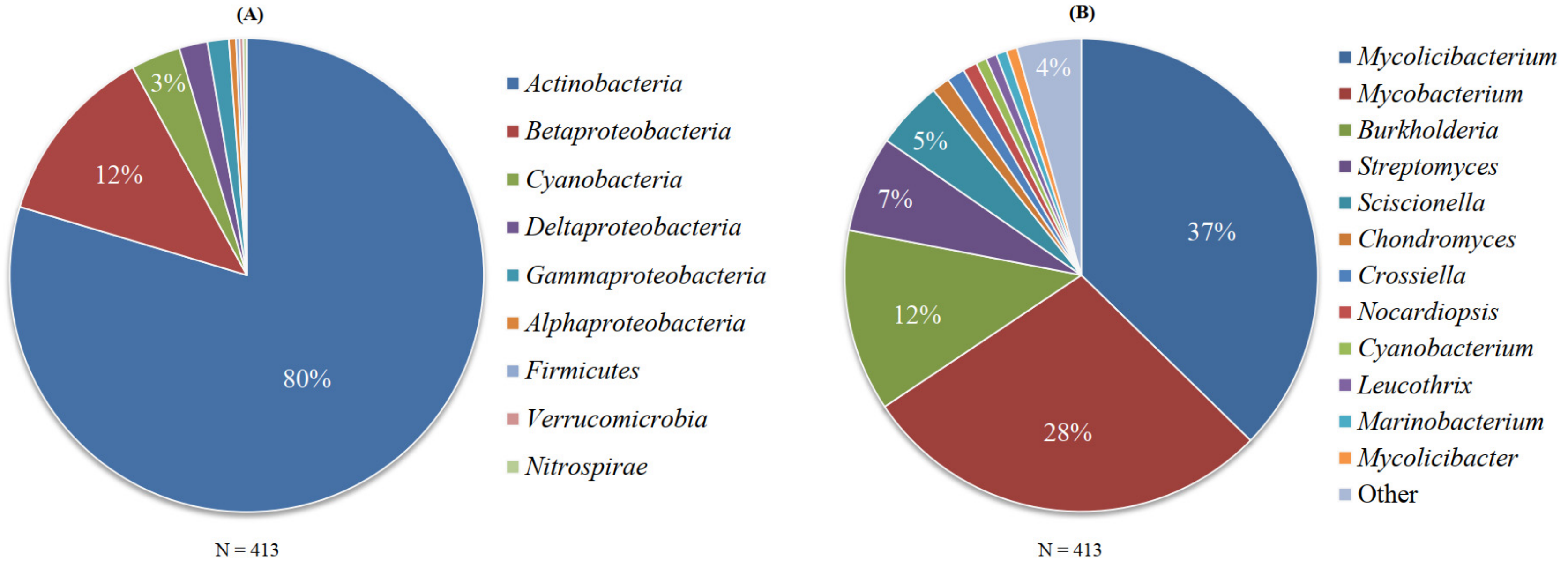

Figure S2. Taxonomy of the most homologous KS sequences with the KS sequences of PKS BGCs identified from the metagenomes of the sponge-associated bacteria retrieved from NCBI Genbank at phylum/class level (A) and genus level (B)

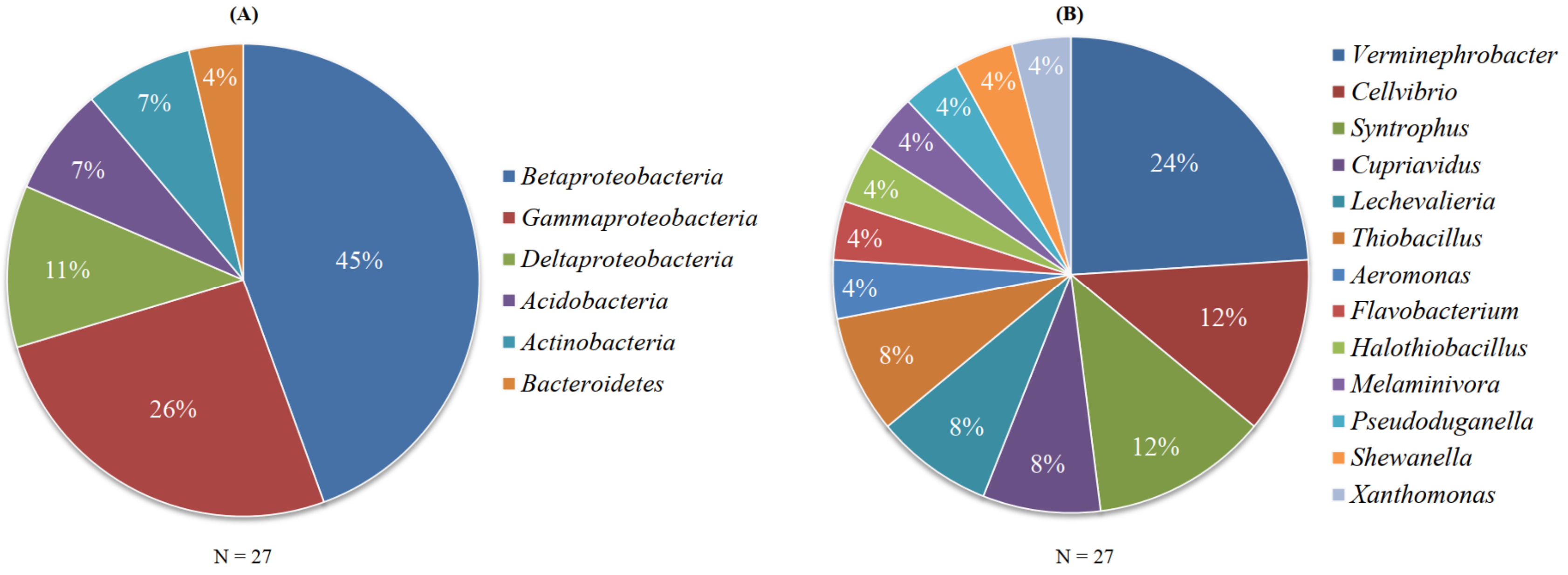

Figure S3. Taxonomy of the most homologous KS sequences with the KS sequences of aryl propylene BGCs identified from the metagenomes of the sponge-associated bacteria retrieved from NCBI Genbank at phylum/class level (A) and genus level (B)

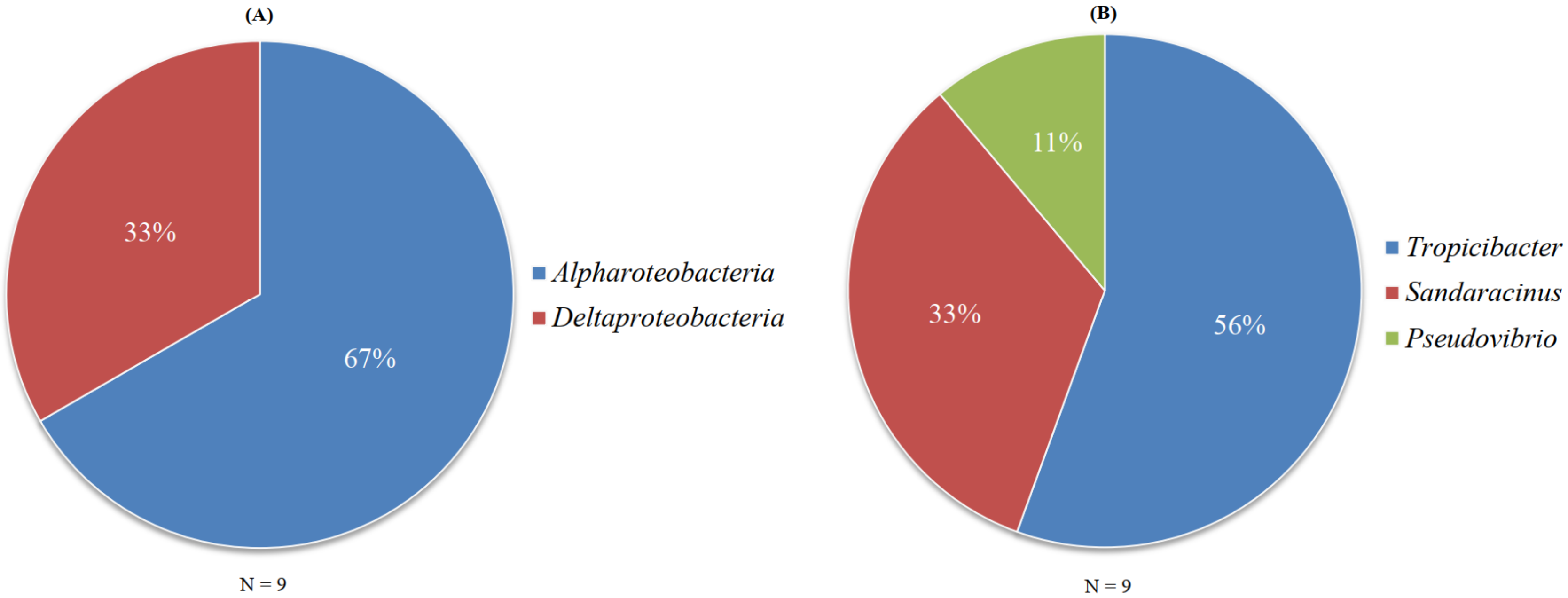

Figure S4. Taxonomy of the most homologous KS sequences with the KS sequences of ladderane BGCs identified from the metagenomes of the sponge-associated bacteria retrieved from NCBI Genbank at phylum/class level (A) and genus level (B)
