# Supplementary material for: Diversity of Bacterial Secondary Metabolite Biosynthetic Gene Clusters in Three Vietnamese Sponges
Source: Mar Drugs. 2022 Dec 29;21(1):29. doi: 10.3390/md21010029 (PMC9864124; doi:10.3390/md21010029)
Supplement: Supplementary file 1 [file marinedrugs-21-00029-s001.zip › Table S1-S16.pdf]

Table S1. Basic data of the three metagenomes of sponge-associated bacteria

Sample ID	Sponge species	No. reads	Contigs	Contigs sum (bp)	N50	Longest contig (bp)	GC content (%)	No. CDS
CLR	*C. reinwardti*	30,414,932	607,208	475,723,993	56,925	236,946	61	888,255
RHG	*R. globostellata*	49,526,287	466,098	486,959,254	21,342	377,852	62	782,060
SPV	*Spheciospongia* sp.	66,835,559	504,262	598,027,097	23,906	1,109,582	61	903,985

Table S2. BGCs identified from the metagenome of *C. reinwardti* by antiSMASH

BGC ID	BGC type	Length (nt)	Most similar known cluster	MIBiG ID
CLR_cluster_008	Arylpolyene	2496	-	-
CLR_cluster_032	Arylpolyene	1296	-	-
CLR_cluster_044	Arylpolyene	2508	-	-
CLR_cluster_070	Arylpolyene	1212	-	-
CLR_cluster_081	Arylpolyene	1917	-	-
CLR_cluster_102	Arylpolyene	1086	-	-
CLR_cluster_137	Arylpolyene	1290	-	-
CLR_cluster_221	Arylpolyene	1197	-	-
CLR_cluster_028	Bacteriocin	1302	-	-
CLR_cluster_037	Bacteriocin	2277	-	-
CLR_cluster_063	Bacteriocin	2328	-	-
CLR_cluster_067	Bacteriocin	2319	-	-
CLR_cluster_092	Bacteriocin	2322	-	-
CLR_cluster_119	Bacteriocin	2316	-	-
CLR_cluster_120	Bacteriocin	2280	-	-
CLR_cluster_169	Bacteriocin	1302	-	-
CLR_cluster_170	Bacteriocin	1206	-	-
CLR_cluster_195	Bacteriocin	2259	-	-
CLR_cluster_274	Bacteriocin	1026	-	-
CLR_cluster_280	Bacteriocin	1086	-	-
CLR_cluster_001	Cf_fatty_acid	1242	-	-
CLR_cluster_006	Cf_fatty_acid	1236	-	-
CLR_cluster_007	Cf_fatty_acid	1239	-	-
CLR_cluster_009	Cf_fatty_acid	1185	-	-
CLR_cluster_012	Cf_fatty_acid	1236	-	-
CLR_cluster_013	Cf_fatty_acid	1242	-	-
CLR_cluster_015	Cf_fatty_acid	1236	-	-
CLR_cluster_017	Cf_fatty_acid	1182	-	-
CLR_cluster_022	Cf_fatty_acid	1245	-	-
CLR_cluster_023	Cf_fatty_acid	1008	-	-
CLR_cluster_025	Cf_fatty_acid	1263	-	-
CLR_cluster_030	Cf_fatty_acid	1266	-	-
CLR_cluster_031	Cf_fatty_acid	1071	-	-
CLR_cluster_041	Cf_fatty_acid	1239	-	-
CLR_cluster_043	Cf_fatty_acid	1374	-	-
CLR_cluster_045	Cf_fatty_acid	1242	-	-
CLR_cluster_046	Cf_fatty_acid	1044	-	-
CLR_cluster_049	Cf_fatty_acid	1239	-	-
CLR_cluster_060	Cf_fatty_acid	1278	-	-
CLR_cluster_064	Cf_fatty_acid	1236	-	-
CLR_cluster_065	Cf_fatty_acid	1251	-	-

CLR_cluster_069	Cf_fatty_acid	1188	-	-
CLR_cluster_072	Cf_fatty_acid	1278	-	-
CLR_cluster_077	Cf_fatty_acid	1083	-	-
CLR_cluster_079	Cf_fatty_acid	1263	-	-
CLR_cluster_082	Cf_fatty_acid	1236	-	-
CLR_cluster_085	Cf_fatty_acid	1233	-	-
CLR_cluster_086	Cf_fatty_acid	1263	-	-
CLR_cluster_096	Cf_fatty_acid	1263	-	-
CLR_cluster_097	Cf_fatty_acid	1239	-	-
CLR_cluster_098	Cf_fatty_acid	1248	-	-
CLR_cluster_101	Cf_fatty_acid	1248	-	-
CLR_cluster_104	Cf_fatty_acid	1260	-	-
CLR_cluster_109	Cf_fatty_acid	1575	-	-
CLR_cluster_110	Cf_fatty_acid	1218	-	-
CLR_cluster_112	Cf_fatty_acid	1197	-	-
CLR_cluster_113	Cf_fatty_acid	1008	-	-
CLR_cluster_114	Cf_fatty_acid	1188	-	-
CLR_cluster_118	Cf_fatty_acid	1185	-	-
CLR_cluster_121	Cf_fatty_acid	1242	-	-
CLR_cluster_122	Cf_fatty_acid	1239	-	-
CLR_cluster_126	Cf_fatty_acid	1224	-	-
CLR_cluster_127	Cf_fatty_acid	1212	-	-
CLR_cluster_128	Cf_fatty_acid	1254	-	-
CLR_cluster_134	Cf_fatty_acid	1251	-	-
CLR_cluster_138	Cf_fatty_acid	1011	-	-
CLR_cluster_139	Cf_fatty_acid	1299	-	-
CLR_cluster_140	Cf_fatty_acid	1176	-	-
CLR_cluster_141	Cf_fatty_acid	1041	-	-
CLR_cluster_143	Cf_fatty_acid	1245	-	-
CLR_cluster_145	Cf_fatty_acid	1272	-	-
CLR_cluster_147	Cf_fatty_acid	1251	-	-
CLR_cluster_148	Cf_fatty_acid	1116	-	-
CLR_cluster_156	Cf_fatty_acid	1305	-	-
CLR_cluster_162	Cf_fatty_acid	1242	-	-
CLR_cluster_165	Cf_fatty_acid	1242	-	-
CLR_cluster_166	Cf_fatty_acid	1245	-	-
CLR_cluster_179	Cf_fatty_acid	1248	-	-
CLR_cluster_184	Cf_fatty_acid	1287	-	-
CLR_cluster_190	Cf_fatty_acid	1236	-	-
CLR_cluster_191	Cf_fatty_acid	1260	-	-
CLR_cluster_193	Cf_fatty_acid	1242	-	-
CLR_cluster_200	Cf_fatty_acid	1281	-	-
CLR_cluster_201	Cf_fatty_acid	1254	-	-

CLR_cluster_204	Cf_fatty_acid	1278	-	-
CLR_cluster_214	Cf_fatty_acid	1188	-	-
CLR_cluster_220	Cf_fatty_acid	1239	-	-
CLR_cluster_223	Cf_fatty_acid	1062	-	-
CLR_cluster_224	Cf_fatty_acid	1242	-	-
CLR_cluster_226	Cf_fatty_acid	1098	-	-
CLR_cluster_227	Cf_fatty_acid	1008	-	-
CLR_cluster_228	Cf_fatty_acid	1257	-	-
CLR_cluster_230	Cf_fatty_acid	1227	-	-
CLR_cluster_231	Cf_fatty_acid	1266	-	-
CLR_cluster_232	Cf_fatty_acid	1137	-	-
CLR_cluster_241	Cf_fatty_acid	1011	-	-
CLR_cluster_251	Cf_fatty_acid	1008	-	-
CLR_cluster_252	Cf_fatty_acid	1071	-	-
CLR_cluster_253	Cf_fatty_acid	1212	-	-
CLR_cluster_257	Cf_fatty_acid	1218	-	-
CLR_cluster_261	Cf_fatty_acid	1269	-	-
CLR_cluster_262	Cf_fatty_acid	1032	-	-
CLR_cluster_264	Cf_fatty_acid	1182	-	-
CLR_cluster_275	Cf_fatty_acid	1083	-	-
CLR_cluster_277	Cf_fatty_acid	1140	-	-
CLR_cluster_281	Cf_fatty_acid	1056	-	-
CLR_cluster_010	Ladderane	1260	-	-
CLR_cluster_042	Ladderane	1239	-	-
CLR_cluster_105	Ladderane	1929	-	-
CLR_cluster_187	Lantipeptide	3051	-	-
CLR_cluster_215	Lantipeptide	2685	-	-
CLR_cluster_011	Other	4272	-	-
CLR_cluster_018	Other	4245	-	-
CLR_cluster_036	Other	2793	-	-
CLR_cluster_051	Other	3084	-	-
CLR_cluster_054	Other	3087	-	-
CLR_cluster_083	Other	2586	-	-
CLR_cluster_090	Other	3261	-	-
CLR_cluster_094	Other	3534	-	-
CLR_cluster_111	Other	2643	-	-
CLR_cluster_142	Other	3066	-	-
CLR_cluster_151	Other	1716	-	-
CLR_cluster_164	Other	2586	-	-
CLR_cluster_182	Other	3705	-	-
CLR_cluster_196	Other	2919	-	-
CLR_cluster_238	Other	2169	-	-
CLR_cluster_258	Other	1674	-	-

CLR_cluster_259	Other	1569	-	-
CLR_cluster_273	Other	1239	-	-
CLR_cluster_276	Other	1182	-	-
CLR_cluster_158	Phosphonate	1650	-	-
CLR_cluster_171	Phosphonate	1368	-	-
CLR_cluster_209	Phosphonate	1635	-	-
CLR_cluster_240	Phosphonate	1626	-	-
CLR_cluster_267	Phosphonate	1446	-	-
CLR_cluster_002	Terpene	3462	-	-
CLR_cluster_003	Terpene	1083	-	-
CLR_cluster_004	Terpene	1074	-	-
CLR_cluster_016	Terpene	1062	-	-
CLR_cluster_019	Terpene	1734	-	-
CLR_cluster_033	Terpene	3435	-	-
CLR_cluster_035	Terpene	2166	-	-
CLR_cluster_038	Terpene	2139	-	-
CLR_cluster_039	Terpene	1017	-	-
CLR_cluster_052	Terpene	1071	-	-
CLR_cluster_061	Terpene	1296	-	-
CLR_cluster_071	Terpene	1020	-	-
CLR_cluster_073	Terpene	1674	-	-
CLR_cluster_074	Terpene	1050	-	-
CLR_cluster_078	Terpene	3228	-	-
CLR_cluster_084	Terpene	1692	-	-
CLR_cluster_088	Terpene	1824	-	-
CLR_cluster_091	Terpene	3405	-	-
CLR_cluster_095	Terpene	3453	-	-
CLR_cluster_099	Terpene	1047	-	-
CLR_cluster_100	Terpene	1044	-	-
CLR_cluster_115	Terpene	1068	-	-
CLR_cluster_116	Terpene	1140	-	-
CLR_cluster_129	Terpene	2082	-	-
CLR_cluster_150	Terpene	1008	-	-
CLR_cluster_152	Terpene	1647	-	-
CLR_cluster_157	Terpene	2010	-	-
CLR_cluster_167	Terpene	2313	-	-
CLR_cluster_168	Terpene	1887	-	-
CLR_cluster_172	Terpene	1680	-	-
CLR_cluster_178	Terpene	1041	-	-
CLR_cluster_185	Terpene	1728	-	-
CLR_cluster_186	Terpene	3264	-	-
CLR_cluster_188	Terpene	1899	-	-
CLR_cluster_189	Terpene	1239	-	-

CLR_cluster_198	Terpene	1065	-	-
CLR_cluster_199	Terpene	1050	-	-
CLR_cluster_205	Terpene	1332	-	-
CLR_cluster_207	Terpene	1974	-	-
CLR_cluster_210	Terpene	1035	-	-
CLR_cluster_211	Terpene	1068	-	-
CLR_cluster_213	Terpene	1035	-	-
CLR_cluster_216	Terpene	2109	-	-
CLR_cluster_217	Terpene	2028	-	-
CLR_cluster_229	Terpene	1020	-	-
CLR_cluster_233	Terpene	1050	-	-
CLR_cluster_236	Terpene	1482	-	-
CLR_cluster_237	Terpene	1524	-	-
CLR_cluster_239	Terpene	2001	-	-
CLR_cluster_244	Terpene	1614	-	-
CLR_cluster_254	Terpene	1011	-	-
CLR_cluster_256	Terpene	1005	-	-
CLR_cluster_260	Terpene	1665	-	-
CLR_cluster_266	Terpene	1209	-	-
CLR_cluster_269	Terpene	1215	-	-
CLR_cluster_271	Terpene	1131	-	-
CLR_cluster_272	Terpene	1266	-	-
CLR_cluster_282	Terpene	1014	-	-
CLR_cluster_048	TransAT-Type I PKS	8226	-	-
CLR_cluster_068	TransAT-Type I PKS	8994	-	-
CLR_cluster_005	Type I PKS	10320	-	-
CLR_cluster_014	Type I PKS	6555	-	-
CLR_cluster_020	Type I PKS	19140	-	-
CLR_cluster_021	Type I PKS	7794	-	-
CLR_cluster_024	Type I PKS	2562	-	-
CLR_cluster_026	Type I PKS	19746	-	-
CLR_cluster_027	Type I PKS	7008	-	-
CLR_cluster_029	Type I PKS	8106	-	-
CLR_cluster_034	Type I PKS	10374	-	-
CLR_cluster_040	Type I PKS	10056	-	-
CLR_cluster_047	Type I PKS	16260	-	-
CLR_cluster_050	Type I PKS	2832	-	-
CLR_cluster_053	Type I PKS	7077	-	-
CLR_cluster_055	Type I PKS	7068	-	-
CLR_cluster_056	Type I PKS	10275	-	-
CLR_cluster_057	Type I PKS	2772	-	-
CLR_cluster_058	Type I PKS	11181	-	-
CLR_cluster_059	Type I PKS	2649	-	-

CLR_cluster_062	Type I PKS	6495	-	-
CLR_cluster_066	Type I PKS	4437	-	-
CLR_cluster_075	Type I PKS	10881	-	-
CLR_cluster_076	Type I PKS	3072	-	-
CLR_cluster_080	Type I PKS	5496	-	-
CLR_cluster_087	Type I PKS	4038	-	-
CLR_cluster_089	Type I PKS	4959	-	-
CLR_cluster_093	Type I PKS	9165	-	-
CLR_cluster_103	Type I PKS	6642	-	-
CLR_cluster_106	Type I PKS	4266	-	-
CLR_cluster_107	Type I PKS	7161	-	-
CLR_cluster_108	Type I PKS	3225	-	-
CLR_cluster_117	Type I PKS	5385	-	-
CLR_cluster_123	Type I PKS	6360	-	-
CLR_cluster_124	Type I PKS	4455	-	-
CLR_cluster_125	Type I PKS	2550	-	-
CLR_cluster_130	Type I PKS	1446	-	-
CLR_cluster_131	Type I PKS	2880	-	-
CLR_cluster_132	Type I PKS	4416	-	-
CLR_cluster_133	Type I PKS	5451	-	-
CLR_cluster_135	Type I PKS	3354	-	-
CLR_cluster_136	Type I PKS	4158	-	-
CLR_cluster_144	Type I PKS	2556	-	-
CLR_cluster_146	Type I PKS	5475	-	-
CLR_cluster_149	Type I PKS	5166	-	-
CLR_cluster_153	Type I PKS	5187	-	-
CLR_cluster_154	Type I PKS	4743	-	-
CLR_cluster_155	Type I PKS	2772	-	-
CLR_cluster_159	Type I PKS	3222	-	-
CLR_cluster_160	Type I PKS	5052	-	-
CLR_cluster_161	Type I PKS	4944	-	-
CLR_cluster_163	Type I PKS	4758	-	-
CLR_cluster_173	Type I PKS	4287	-	-
CLR_cluster_174	Type I PKS	2730	-	-
CLR_cluster_175	Type I PKS	4134	-	-
CLR_cluster_176	Type I PKS	4062	-	-
CLR_cluster_177	Type I PKS	3267	-	-
CLR_cluster_180	Type I PKS	3852	-	-
CLR_cluster_181	Type I PKS	2946	-	-
CLR_cluster_183	Type I PKS	2442	-	-
CLR_cluster_192	Type I PKS	3393	-	-
CLR_cluster_194	Type I PKS	3096	-	-
CLR_cluster_197	Type I PKS	3294	-	-

CLR_cluster_202	Type I PKS	2442	-	-
CLR_cluster_203	Type I PKS	3012	-	-
CLR_cluster_206	Type I PKS	2760	-	-
CLR_cluster_208	Type I PKS	2421	-	-
CLR_cluster_212	Type I PKS	2661	-	-
CLR_cluster_218	Type I PKS	2649	-	-
CLR_cluster_219	Type I PKS	2529	-	-
CLR_cluster_222	Type I PKS	2601	-	-
CLR_cluster_225	Type I PKS	2532	-	-
CLR_cluster_234	Type I PKS	2259	-	-
CLR_cluster_235	Type I PKS	2211	-	-
CLR_cluster_242	Type I PKS	2094	-	-
CLR_cluster_245	Type I PKS	1557	-	-
CLR_cluster_246	Type I PKS	1977	-	-
CLR_cluster_247	Type I PKS	2019	-	-
CLR_cluster_248	Type I PKS	2004	-	-
CLR_cluster_249	Type I PKS	2004	-	-
CLR_cluster_250	Type I PKS	1980	-	-
CLR_cluster_255	Type I PKS	1830	-	-
CLR_cluster_265	Type I PKS	1509	-	-
CLR_cluster_268	Type I PKS	1419	-	-
CLR_cluster_278	Type I PKS	1131	-	-
CLR_cluster_279	Type II PKS	1026	-	-
CLR_cluster_243	Type III PKS	1011	-	-
CLR_cluster_263	Type III PKS	1032	-	-
CLR_cluster_270	Type III PKS	1086	-	-

"-": no significant hit

Table S3. BGCs identified from the metagenome of *R. globostellata* by antiSMASH

BGC ID	BGC type	Length (nt)	Most similar known cluster	MIBiG ID
RHG_cluster_046	Arylpolyene	2496	-	-
RHG_cluster_061	Arylpolyene	1197	-	-
RHG_cluster_120	Arylpolyene	1086	-	-
RHG_cluster_121	Arylpolyene	1212	-	-
RHG_cluster_125	Arylpolyene	1233	-	-
RHG_cluster_134	Arylpolyene	2508	-	-
RHG_cluster_184	Arylpolyene	1917	-	-
RHG_cluster_231	Arylpolyene	1230	-	-
RHG_cluster_232	Arylpolyene	1230	-	-
RHG_cluster_253	Arylpolyene	1290	-	-
RHG_cluster_005	Bacteriocin	2316	-	-
RHG_cluster_021	Bacteriocin	2355	-	-
RHG_cluster_024	Bacteriocin	1350	-	-
RHG_cluster_025	Bacteriocin	2277	-	-
RHG_cluster_032	Bacteriocin	2280	-	-
RHG_cluster_038	Bacteriocin	2322	-	-
RHG_cluster_083	Bacteriocin	2328	-	-
RHG_cluster_092	Bacteriocin	1929	-	-
RHG_cluster_115	Bacteriocin	2319	-	-
RHG_cluster_139	Bacteriocin	2292	-	-
RHG_cluster_181	Bacteriocin	2319	-	-
RHG_cluster_189	Bacteriocin	1308	-	-
RHG_cluster_251	Bacteriocin	1302	-	-
RHG_cluster_257	Bacteriocin	1911	-	-
RHG_cluster_297	Bacteriocin	2952	-	-
RHG_cluster_325	Bacteriocin	1197	-	-
RHG_cluster_004	Cf_fatty_acid	1263	-	-
RHG_cluster_007	Cf_fatty_acid	1236	-	-
RHG_cluster_009	Cf_fatty_acid	1236	-	-
RHG_cluster_010	Cf_fatty_acid	1008	-	-
RHG_cluster_012	Cf_fatty_acid	1242	-	-
RHG_cluster_018	Cf_fatty_acid	1239	-	-
RHG_cluster_020	Cf_fatty_acid	1254	-	-
RHG_cluster_023	Cf_fatty_acid	1248	-	-
RHG_cluster_030	Cf_fatty_acid	1248	-	-
RHG_cluster_031	Cf_fatty_acid	1266	-	-
RHG_cluster_033	Cf_fatty_acid	1212	-	-
RHG_cluster_035	Cf_fatty_acid	1245	-	-
RHG_cluster_039	Cf_fatty_acid	1257	-	-
RHG_cluster_045	Cf_fatty_acid	1185	-	-
RHG_cluster_049	Cf_fatty_acid	1236	-	-

RHG_cluster_051	Cf_fatty_acid	1182	-	-
RHG_cluster_059	Cf_fatty_acid	1182	-	-
RHG_cluster_065	Cf_fatty_acid	1278	-	-
RHG_cluster_068	Cf_fatty_acid	1236	-	-
RHG_cluster_073	Cf_fatty_acid	1218	-	-
RHG_cluster_074	Cf_fatty_acid	1206	-	-
RHG_cluster_075	Cf_fatty_acid	1254	-	-
RHG_cluster_077	Cf_fatty_acid	1287	-	-
RHG_cluster_078	Cf_fatty_acid	1299	-	-
RHG_cluster_079	Cf_fatty_acid	1266	-	-
RHG_cluster_081	Cf_fatty_acid	1311	-	-
RHG_cluster_084	Cf_fatty_acid	1263	-	-
RHG_cluster_085	Cf_fatty_acid	1188	-	-
RHG_cluster_086	Cf_fatty_acid	1260	-	-
RHG_cluster_089	Cf_fatty_acid	1032	-	-
RHG_cluster_095	Cf_fatty_acid	1212	-	-
RHG_cluster_097	Cf_fatty_acid	1281	-	-
RHG_cluster_098	Cf_fatty_acid	1242	-	-
RHG_cluster_099	Cf_fatty_acid	1005	-	-
RHG_cluster_103	Cf_fatty_acid	1155	-	-
RHG_cluster_108	Cf_fatty_acid	1218	-	-
RHG_cluster_112	Cf_fatty_acid	1008	-	-
RHG_cluster_114	Cf_fatty_acid	1233	-	-
RHG_cluster_122	Cf_fatty_acid	1236	-	-
RHG_cluster_127	Cf_fatty_acid	1263	-	-
RHG_cluster_130	Cf_fatty_acid	1251	-	-
RHG_cluster_133	Cf_fatty_acid	1374	-	-
RHG_cluster_138	Cf_fatty_acid	1239	-	-
RHG_cluster_141	Cf_fatty_acid	1080	-	-
RHG_cluster_142	Cf_fatty_acid	1245	-	-
RHG_cluster_143	Cf_fatty_acid	1254	-	-
RHG_cluster_144	Cf_fatty_acid	1236	-	-
RHG_cluster_148	Cf_fatty_acid	1242	-	-
RHG_cluster_150	Cf_fatty_acid	1263	-	-
RHG_cluster_151	Cf_fatty_acid	1197	-	-
RHG_cluster_153	Cf_fatty_acid	1224	-	-
RHG_cluster_155	Cf_fatty_acid	1272	-	-
RHG_cluster_156	Cf_fatty_acid	1227	-	-
RHG_cluster_172	Cf_fatty_acid	1260	-	-
RHG_cluster_173	Cf_fatty_acid	1005	-	-
RHG_cluster_175	Cf_fatty_acid	1044	-	-
RHG_cluster_176	Cf_fatty_acid	1956	-	-
RHG_cluster_178	Cf_fatty_acid	1254	-	-

RHG_cluster_182	Cf_fatty_acid	1854	-	-
RHG_cluster_185	Cf_fatty_acid	1251	-	-
RHG_cluster_186	Cf_fatty_acid	1032	-	-
RHG_cluster_188	Cf_fatty_acid	1242	-	-
RHG_cluster_190	Cf_fatty_acid	1101	-	-
RHG_cluster_196	Cf_fatty_acid	1116	-	-
RHG_cluster_211	Cf_fatty_acid	1071	-	-
RHG_cluster_212	Cf_fatty_acid	1176	-	-
RHG_cluster_214	Cf_fatty_acid	1260	-	-
RHG_cluster_216	Cf_fatty_acid	1272	-	-
RHG_cluster_218	Cf_fatty_acid	1242	-	-
RHG_cluster_220	Cf_fatty_acid	1245	-	-
RHG_cluster_222	Cf_fatty_acid	1263	-	-
RHG_cluster_225	Cf_fatty_acid	1242	-	-
RHG_cluster_227	Cf_fatty_acid	1233	-	-
RHG_cluster_228	Cf_fatty_acid	1044	-	-
RHG_cluster_235	Cf_fatty_acid	1239	-	-
RHG_cluster_236	Cf_fatty_acid	1185	-	-
RHG_cluster_239	Cf_fatty_acid	1035	-	-
RHG_cluster_241	Cf_fatty_acid	1218	-	-
RHG_cluster_244	Cf_fatty_acid	1254	-	-
RHG_cluster_245	Cf_fatty_acid	1077	-	-
RHG_cluster_250	Cf_fatty_acid	1212	-	-
RHG_cluster_254	Cf_fatty_acid	1011	-	-
RHG_cluster_259	Cf_fatty_acid	1188	-	-
RHG_cluster_263	Cf_fatty_acid	1239	-	-
RHG_cluster_264	Cf_fatty_acid	1248	-	-
RHG_cluster_265	Cf_fatty_acid	1170	-	-
RHG_cluster_270	Cf_fatty_acid	1542	-	-
RHG_cluster_271	Cf_fatty_acid	1293	-	-
RHG_cluster_273	Cf_fatty_acid	1188	-	-
RHG_cluster_274	Cf_fatty_acid	1242	-	-
RHG_cluster_275	Cf_fatty_acid	1092	-	-
RHG_cluster_277	Cf_fatty_acid	1263	-	-
RHG_cluster_278	Cf_fatty_acid	1233	-	-
RHG_cluster_281	Cf_fatty_acid	1245	-	-
RHG_cluster_286	Cf_fatty_acid	1008	-	-
RHG_cluster_292	Cf_fatty_acid	1086	-	-
RHG_cluster_293	Cf_fatty_acid	1005	-	-
RHG_cluster_301	Cf_fatty_acid	1251	-	-
RHG_cluster_303	Cf_fatty_acid	1221	-	-
RHG_cluster_305	Cf_fatty_acid	1245	-	-
RHG_cluster_310	Cf_fatty_acid	1164	-	-

RHG_cluster_313	Cf_fatty_acid	1254	-	-
RHG_cluster_315	Cf_fatty_acid	1101	-	-
RHG_cluster_317	Cf_fatty_acid	1263	-	-
RHG_cluster_319	Cf_fatty_acid	1248	-	-
RHG_cluster_321	Cf_fatty_acid	1113	-	-
RHG_cluster_324	Cf_fatty_acid	1236	-	-
RHG_cluster_328	Cf_fatty_acid	1002	-	-
RHG_cluster_332	Cf_fatty_acid	1191	-	-
RHG_cluster_334	Cf_fatty_acid	1215	-	-
RHG_cluster_343	Cf_fatty_acid	1104	-	-
RHG_cluster_344	Cf_fatty_acid	1242	-	-
RHG_cluster_347	Cf_fatty_acid	1242	-	-
RHG_cluster_348	Cf_fatty_acid	1260	-	-
RHG_cluster_352	Cf_fatty_acid	1020	-	-
RHG_cluster_355	Cf_fatty_acid	1014	-	-
RHG_cluster_359	Cf_fatty_acid	1110	-	-
RHG_cluster_360	Cf_fatty_acid	1212	-	-
RHG_cluster_365	Cf_fatty_acid	1026	-	-
RHG_cluster_044	Ladderane	1260	-	-
RHG_cluster_111	Ladderane	1929	-	-
RHG_cluster_116	Ladderane	1926	-	-
RHG_cluster_132	Ladderane	1239	-	-
RHG_cluster_093	Lantipeptide	3186	-	-
RHG_cluster_001	Other	4272	-	-
RHG_cluster_022	Other	2550	-	-
RHG_cluster_029	Other	2589	-	-
RHG_cluster_048	Other	4188	-	-
RHG_cluster_064	Other	2586	-	-
RHG_cluster_066	Other	1941	-	-
RHG_cluster_070	Other	2883	-	-
RHG_cluster_082	Other	2796	-	-
RHG_cluster_088	Other	3084	-	-
RHG_cluster_110	Other	2970	-	-
RHG_cluster_126	Other	2874	-	-
RHG_cluster_131	Other	2802	-	-
RHG_cluster_171	Other	2643	-	-
RHG_cluster_187	Other	2529	-	-
RHG_cluster_205	Other	3171	-	-
RHG_cluster_210	Other	2259	-	-
RHG_cluster_223	Other	3087	-	-
RHG_cluster_260	Other	2322	-	-
RHG_cluster_287	Other	2793	-	-
RHG_cluster_300	Other	2802	-	-

RHG_cluster_312	Other	2895	-	-
RHG_cluster_329	Other	2412	-	-
RHG_cluster_333	Other	2304	-	-
RHG_cluster_339	Other	1317	-	-
RHG_cluster_346	Other	1842	-	-
RHG_cluster_349	Other	1731	-	-
RHG_cluster_361	Other	1215	-	-
RHG_cluster_368	Other	1077	-	-
RHG_cluster_371	Other	1008	-	-
RHG_cluster_166	Phosphonate	1650	-	-
RHG_cluster_230	Phosphonate	1635	-	-
RHG_cluster_290	Phosphonate	1680	-	-
RHG_cluster_291	Phosphonate	1665	-	-
RHG_cluster_370	Phosphonate	1029	-	-
RHG_cluster_002	Terpene	1995	-	-
RHG_cluster_003	Terpene	3462	-	-
RHG_cluster_006	Terpene	1062	-	-
RHG_cluster_013	Terpene	1065	-	-
RHG_cluster_015	Terpene	2166	-	-
RHG_cluster_026	Terpene	1020	-	-
RHG_cluster_036	Terpene	1716	-	-
RHG_cluster_041	Terpene	3501	-	-
RHG_cluster_042	Terpene	1041	-	-
RHG_cluster_043	Terpene	3435	-	-
RHG_cluster_047	Terpene	1074	-	-
RHG_cluster_050	Terpene	3408	-	-
RHG_cluster_052	Terpene	1059	-	-
RHG_cluster_055	Terpene	3441	-	-
RHG_cluster_058	Terpene	3432	-	-
RHG_cluster_060	Terpene	1974	-	-
RHG_cluster_063	Terpene	1041	-	-
RHG_cluster_071	Terpene	1968	-	-
RHG_cluster_101	Terpene	1068	-	-
RHG_cluster_102	Terpene	3390	-	-
RHG_cluster_104	Terpene	2160	-	-
RHG_cluster_105	Terpene	1044	-	-
RHG_cluster_106	Terpene	1047	-	-
RHG_cluster_107	Terpene	1095	-	-
RHG_cluster_113	Terpene	1044	-	-
RHG_cluster_118	Terpene	1728	-	-
RHG_cluster_123	Terpene	3405	-	-
RHG_cluster_137	Terpene	1962	-	-
RHG_cluster_146	Terpene	2016	-	-

RHG_cluster_152	Terpene	2022	-	-
RHG_cluster_157	Terpene	3381	-	-
RHG_cluster_158	Terpene	1098	-	-
RHG_cluster_169	Terpene	3453	-	-
RHG_cluster_170	Terpene	1068	-	-
RHG_cluster_180	Terpene	1050	-	-
RHG_cluster_199	Terpene	1041	-	-
RHG_cluster_200	Terpene	1020	-	-
RHG_cluster_201	Terpene	1071	-	-
RHG_cluster_207	Terpene	1050	-	-
RHG_cluster_219	Terpene	1095	-	-
RHG_cluster_224	Terpene	3420	-	-
RHG_cluster_234	Terpene	2022	-	-
RHG_cluster_243	Terpene	1068	-	-
RHG_cluster_247	Terpene	3135	-	-
RHG_cluster_252	Terpene	1983	-	-
RHG_cluster_256	Terpene	2139	-	-
RHG_cluster_276	Terpene	1992	-	-
RHG_cluster_280	Terpene	1899	-	-
RHG_cluster_284	Terpene	2067	-	-
RHG_cluster_299	Terpene	1572	-	-
RHG_cluster_304	Terpene	1995	-	-
RHG_cluster_309	Terpene	2028	-	-
RHG_cluster_311	Terpene	1650	-	-
RHG_cluster_314	Terpene	1197	-	-
RHG_cluster_320	Terpene	1071	-	-
RHG_cluster_323	Terpene	1269	-	-
RHG_cluster_327	Terpene	1287	-	-
RHG_cluster_330	Terpene	1386	-	-
RHG_cluster_331	Terpene	1806	-	-
RHG_cluster_337	Terpene	1800	-	-
RHG_cluster_350	Terpene	1071	-	-
RHG_cluster_353	Terpene	1434	-	-
RHG_cluster_363	Terpene	1044	-	-
RHG_cluster_364	Terpene	1176	-	-
RHG_cluster_366	Terpene	1122	-	-
RHG_cluster_369	Terpene	1032	-	-
RHG_cluster_014	TransAT-PKS	4770	-	-
RHG_cluster_161	TransAT-PKS	4773	-	-
RHG_cluster_008	Type I PKS	10395	-	-
RHG_cluster_011	Type I PKS	7044	-	-
RHG_cluster_016	Type I PKS	7062	-	-
RHG_cluster_017	Type I PKS	1428	-	-

RHG_cluster_019	Type I PKS	22404	-	-
RHG_cluster_027	Type I PKS	10014	-	-
RHG_cluster_028	Type I PKS	7044	-	-
RHG_cluster_034	Type I PKS	7008	-	-
RHG_cluster_037	Type I PKS	10272	-	-
RHG_cluster_040	Type I PKS	9582	-	-
RHG_cluster_053	Type I PKS	9519	-	-
RHG_cluster_054	Type I PKS	9642	-	-
RHG_cluster_056	Type I PKS	10263	-	-
RHG_cluster_057	Type I PKS	10320	-	-
RHG_cluster_062	Type I PKS	6021	-	-
RHG_cluster_067	Type I PKS	7056	-	-
RHG_cluster_069	Type I PKS	10290	-	-
RHG_cluster_072	Type I PKS	7101	-	-
RHG_cluster_087	Type I PKS	9624	-	-
RHG_cluster_090	Type I PKS	10269	-	-
RHG_cluster_091	Type I PKS	7053	-	-
RHG_cluster_094	Type I PKS	4569	-	-
RHG_cluster_096	Type I PKS	7059	-	-
RHG_cluster_100	Type I PKS	10335	-	-
RHG_cluster_109	Type I PKS	5502	-	-
RHG_cluster_119	Type I PKS	29196	-	-
RHG_cluster_124	Type I PKS	9531	-	-
RHG_cluster_128	Type I PKS	7200	-	-
RHG_cluster_135	Type I PKS	7053	-	-
RHG_cluster_136	Type I PKS	7008	-	-
RHG_cluster_140	Type I PKS	23358	-	-
RHG_cluster_145	Type I PKS	7791	-	-
RHG_cluster_147	Type I PKS	10374	-	-
RHG_cluster_149	Type I PKS	8457	-	-
RHG_cluster_154	Type I PKS	10272	-	-
RHG_cluster_159	Type I PKS	13836	-	-
RHG_cluster_160	Type I PKS	8067	-	-
RHG_cluster_162	Type I PKS	10365	-	-
RHG_cluster_163	Type I PKS	4290	-	-
RHG_cluster_164	Type I PKS	10131	-	-
RHG_cluster_165	Type I PKS	10275	-	-
RHG_cluster_167	Type I PKS	4974	-	-
RHG_cluster_168	Type I PKS	2112	-	-
RHG_cluster_174	Type I PKS	4308	-	-
RHG_cluster_177	Type I PKS	10428	-	-
RHG_cluster_179	Type I PKS	10326	-	-
RHG_cluster_183	Type I PKS	6192	-	-

RHG_cluster_191	Type I PKS	4482	-	-
RHG_cluster_192	Type I PKS	4995	-	-
RHG_cluster_193	Type I PKS	12108	-	-
RHG_cluster_194	Type I PKS	8955	-	-
RHG_cluster_195	Type I PKS	8292	-	-
RHG_cluster_197	Type I PKS	6048	-	-
RHG_cluster_198	Type I PKS	8178	-	-
RHG_cluster_202	Type I PKS	6786	-	-
RHG_cluster_203	Type I PKS	11394	-	-
RHG_cluster_204	Type I PKS	7527	-	-
RHG_cluster_206	Type I PKS	4830	-	-
RHG_cluster_208	Type I PKS	3666	-	-
RHG_cluster_209	Type I PKS	7137	-	-
RHG_cluster_213	Type I PKS	7113	-	-
RHG_cluster_215	Type I PKS	2184	-	-
RHG_cluster_217	Type I PKS	5310	-	-
RHG_cluster_221	Type I PKS	1890	-	-
RHG_cluster_226	Type I PKS	4512	-	-
RHG_cluster_229	Type I PKS	5406	-	-
RHG_cluster_233	Type I PKS	8346	-	-
RHG_cluster_237	Type I PKS	2496	-	-
RHG_cluster_238	Type I PKS	2283	-	-
RHG_cluster_240	Type I PKS	7725	-	-
RHG_cluster_242	Type I PKS	2130	-	-
RHG_cluster_246	Type I PKS	7095	-	-
RHG_cluster_248	Type I PKS	4281	-	-
RHG_cluster_249	Type I PKS	2526	-	-
RHG_cluster_255	Type I PKS	1890	-	-
RHG_cluster_258	Type I PKS	6234	-	-
RHG_cluster_261	Type I PKS	2907	-	-
RHG_cluster_262	Type I PKS	3486	-	-
RHG_cluster_266	Type I PKS	5229	-	-
RHG_cluster_268	Type I PKS	5331	-	-
RHG_cluster_269	Type I PKS	2643	-	-
RHG_cluster_272	Type I PKS	1926	-	-
RHG_cluster_279	Type I PKS	2157	-	-
RHG_cluster_282	Type I PKS	1602	-	-
RHG_cluster_283	Type I PKS	4533	-	-
RHG_cluster_285	Type I PKS	4344	-	-
RHG_cluster_288	Type I PKS	3843	-	-
RHG_cluster_289	Type I PKS	4062	-	-
RHG_cluster_294	Type I PKS	2355	-	-
RHG_cluster_295	Type I PKS	3690	-	-

RHG_cluster_296	Type I PKS	2556	-	-
RHG_cluster_298	Type I PKS	3135	-	-
RHG_cluster_302	Type I PKS	2619	-	-
RHG_cluster_306	Type I PKS	3261	-	-
RHG_cluster_308	Type I PKS	1767	-	-
RHG_cluster_316	Type I PKS	2415	-	-
RHG_cluster_318	Type I PKS	1965	-	-
RHG_cluster_322	Type I PKS	2730	-	-
RHG_cluster_326	Type I PKS	2625	-	-
RHG_cluster_335	Type I PKS	2232	-	-
RHG_cluster_336	Type I PKS	2196	-	-
RHG_cluster_338	Type I PKS	2115	-	-
RHG_cluster_340	Type I PKS	2034	-	-
RHG_cluster_341	Type I PKS	2031	-	-
RHG_cluster_342	Type I PKS	1938	-	-
RHG_cluster_345	Type I PKS	1893	-	-
RHG_cluster_351	Type I PKS	1608	-	-
RHG_cluster_354	Type I PKS	1518	-	-
RHG_cluster_356	Type I PKS	1455	-	-
RHG_cluster_357	Type I PKS	1443	-	-
RHG_cluster_362	Type I PKS	1209	-	-
RHG_cluster_367	Type I PKS	1098	-	-
RHG_cluster_080	Type II PKS	1191	-	-
RHG_cluster_076	Type III PKS	1056	-	-
RHG_cluster_117	Type III PKS	1086	-	-
RHG_cluster_129	Type III PKS	1056	-	-
RHG_cluster_267	Type III PKS	1086	-	-
RHG_cluster_307	Type III PKS	1056	-	-
RHG_cluster_358	Type III PKS	1086	-	-

“-”: no significant hit

Table S4. BGCs identified from the metagenome of *Spheciospongia* sp. by antiSMASH

BGC ID	BGC type	Length (nt)	Most similar known cluster	MIBiG ID
SPV_cluster_094	Arylpolyene	2496	-	-
SPV_cluster_112	Arylpolyene	1296	-	-
SPV_cluster_123	Arylpolyene	2508	-	-
SPV_cluster_136	Arylpolyene	1233	-	-
SPV_cluster_137	Arylpolyene	1212	-	-
SPV_cluster_138	Arylpolyene	1224	-	-
SPV_cluster_312	Arylpolyene	1290	-	-
SPV_cluster_345	Arylpolyene	1176	-	-
SPV_cluster_376	Arylpolyene	1197	-	-
SPV_cluster_021	Bacteriocin	1302	-	-
SPV_cluster_064	Bacteriocin	1929	-	-
SPV_cluster_135	Bacteriocin	2280	-	-
SPV_cluster_139	Bacteriocin	2277	-	-
SPV_cluster_176	Bacteriocin	1302	-	-
SPV_cluster_198	Bacteriocin	1302	-	-
SPV_cluster_199	Bacteriocin	2319	-	-
SPV_cluster_297	Bacteriocin	2277	-	-
SPV_cluster_346	Bacteriocin	1800	-	-
SPV_cluster_350	Bacteriocin	2241	-	-
SPV_cluster_355	Bacteriocin	3021	-	-
SPV_cluster_398	Bacteriocin	1023	-	-
SPV_cluster_424	Bacteriocin	1629	-	-
SPV_cluster_431	Bacteriocin	1230	-	-
SPV_cluster_002	Cf_fatty_acid	1242	-	-
SPV_cluster_003	Cf_fatty_acid	1245	-	-
SPV_cluster_007	Cf_fatty_acid	1263	-	-
SPV_cluster_009	Cf_fatty_acid	1233	-	-
SPV_cluster_014	Cf_fatty_acid	1251	-	-
SPV_cluster_015	Cf_fatty_acid	1236	-	-
SPV_cluster_016	Cf_fatty_acid	1575	-	-
SPV_cluster_024	Cf_fatty_acid	1254	-	-
SPV_cluster_025	Cf_fatty_acid	1242	-	-
SPV_cluster_026	Cf_fatty_acid	1044	-	-
SPV_cluster_027	Cf_fatty_acid	1266	-	-
SPV_cluster_028	Cf_fatty_acid	1053	-	-
SPV_cluster_031	Cf_fatty_acid	1257	-	-
SPV_cluster_032	Cf_fatty_acid	1248	-	-
SPV_cluster_033	Cf_fatty_acid	1239	-	-
SPV_cluster_034	Cf_fatty_acid	1080	-	-
SPV_cluster_036	Cf_fatty_acid	1242	-	-
SPV_cluster_037	Cf_fatty_acid	1242	-	-

SPV_cluster_038	Cf_fatty_acid	1032	-	-
SPV_cluster_039	Cf_fatty_acid	1242	-	-
SPV_cluster_042	Cf_fatty_acid	1281	-	-
SPV_cluster_043	Cf_fatty_acid	1212	-	-
SPV_cluster_045	Cf_fatty_acid	1269	-	-
SPV_cluster_046	Cf_fatty_acid	1170	-	-
SPV_cluster_053	Cf_fatty_acid	1263	-	-
SPV_cluster_054	Cf_fatty_acid	1239	-	-
SPV_cluster_060	Cf_fatty_acid	1239	-	-
SPV_cluster_066	Cf_fatty_acid	1254	-	-
SPV_cluster_069	Cf_fatty_acid	1242	-	-
SPV_cluster_073	Cf_fatty_acid	1119	-	-
SPV_cluster_074	Cf_fatty_acid	1269	-	-
SPV_cluster_076	Cf_fatty_acid	1236	-	-
SPV_cluster_081	Cf_fatty_acid	1248	-	-
SPV_cluster_083	Cf_fatty_acid	1287	-	-
SPV_cluster_085	Cf_fatty_acid	1248	-	-
SPV_cluster_086	Cf_fatty_acid	1182	-	-
SPV_cluster_093	Cf_fatty_acid	1185	-	-
SPV_cluster_099	Cf_fatty_acid	1236	-	-
SPV_cluster_101	Cf_fatty_acid	1269	-	-
SPV_cluster_107	Cf_fatty_acid	1278	-	-
SPV_cluster_109	Cf_fatty_acid	1008	-	-
SPV_cluster_113	Cf_fatty_acid	1188	-	-
SPV_cluster_119	Cf_fatty_acid	1584	-	-
SPV_cluster_121	Cf_fatty_acid	1239	-	-
SPV_cluster_122	Cf_fatty_acid	1005	-	-
SPV_cluster_124	Cf_fatty_acid	1374	-	-
SPV_cluster_126	Cf_fatty_acid	1209	-	-
SPV_cluster_128	Cf_fatty_acid	1239	-	-
SPV_cluster_129	Cf_fatty_acid	1176	-	-
SPV_cluster_130	Cf_fatty_acid	1005	-	-
SPV_cluster_133	Cf_fatty_acid	1071	-	-
SPV_cluster_145	Cf_fatty_acid	1854	-	-
SPV_cluster_148	Cf_fatty_acid	1218	-	-
SPV_cluster_152	Cf_fatty_acid	1227	-	-
SPV_cluster_155	Cf_fatty_acid	1224	-	-
SPV_cluster_159	Cf_fatty_acid	1251	-	-
SPV_cluster_160	Cf_fatty_acid	1263	-	-
SPV_cluster_162	Cf_fatty_acid	1050	-	-
SPV_cluster_173	Cf_fatty_acid	1266	-	-
SPV_cluster_178	Cf_fatty_acid	1257	-	-
SPV_cluster_179	Cf_fatty_acid	1182	-	-

SPV_cluster_180	Cf_fatty_acid	1236	-	-
SPV_cluster_182	Cf_fatty_acid	1242	-	-
SPV_cluster_189	Cf_fatty_acid	1101	-	-
SPV_cluster_192	Cf_fatty_acid	1266	-	-
SPV_cluster_195	Cf_fatty_acid	1242	-	-
SPV_cluster_197	Cf_fatty_acid	1239	-	-
SPV_cluster_200	Cf_fatty_acid	1287	-	-
SPV_cluster_201	Cf_fatty_acid	1293	-	-
SPV_cluster_205	Cf_fatty_acid	1278	-	-
SPV_cluster_207	Cf_fatty_acid	1236	-	-
SPV_cluster_209	Cf_fatty_acid	1239	-	-
SPV_cluster_215	Cf_fatty_acid	1227	-	-
SPV_cluster_216	Cf_fatty_acid	1284	-	-
SPV_cluster_217	Cf_fatty_acid	1245	-	-
SPV_cluster_219	Cf_fatty_acid	1011	-	-
SPV_cluster_222	Cf_fatty_acid	1251	-	-
SPV_cluster_224	Cf_fatty_acid	1188	-	-
SPV_cluster_228	Cf_fatty_acid	1215	-	-
SPV_cluster_229	Cf_fatty_acid	1206	-	-
SPV_cluster_230	Cf_fatty_acid	1227	-	-
SPV_cluster_233	Cf_fatty_acid	1236	-	-
SPV_cluster_235	Cf_fatty_acid	1242	-	-
SPV_cluster_236	Cf_fatty_acid	1206	-	-
SPV_cluster_239	Cf_fatty_acid	1236	-	-
SPV_cluster_240	Cf_fatty_acid	1251	-	-
SPV_cluster_243	Cf_fatty_acid	1989	-	-
SPV_cluster_250	Cf_fatty_acid	1266	-	-
SPV_cluster_251	Cf_fatty_acid	1245	-	-
SPV_cluster_254	Cf_fatty_acid	1242	-	-
SPV_cluster_255	Cf_fatty_acid	1242	-	-
SPV_cluster_256	Cf_fatty_acid	1059	-	-
SPV_cluster_258	Cf_fatty_acid	1248	-	-
SPV_cluster_259	Cf_fatty_acid	1272	-	-
SPV_cluster_261	Cf_fatty_acid	1578	-	-
SPV_cluster_262	Cf_fatty_acid	1074	-	-
SPV_cluster_263	Cf_fatty_acid	1242	-	-
SPV_cluster_271	Cf_fatty_acid	1254	-	-
SPV_cluster_274	Cf_fatty_acid	1242	-	-
SPV_cluster_275	Cf_fatty_acid	1275	-	-
SPV_cluster_276	Cf_fatty_acid	1260	-	-
SPV_cluster_279	Cf_fatty_acid	1098	-	-
SPV_cluster_280	Cf_fatty_acid	1239	-	-
SPV_cluster_284	Cf_fatty_acid	1242	-	-

SPV_cluster_285	Cf_fatty_acid	1053	-	-
SPV_cluster_289	Cf_fatty_acid	1242	-	-
SPV_cluster_290	Cf_fatty_acid	1185	-	-
SPV_cluster_293	Cf_fatty_acid	1218	-	-
SPV_cluster_296	Cf_fatty_acid	1116	-	-
SPV_cluster_301	Cf_fatty_acid	1278	-	-
SPV_cluster_307	Cf_fatty_acid	1227	-	-
SPV_cluster_308	Cf_fatty_acid	1272	-	-
SPV_cluster_313	Cf_fatty_acid	1011	-	-
SPV_cluster_316	Cf_fatty_acid	1242	-	-
SPV_cluster_317	Cf_fatty_acid	1251	-	-
SPV_cluster_318	Cf_fatty_acid	1053	-	-
SPV_cluster_320	Cf_fatty_acid	1188	-	-
SPV_cluster_322	Cf_fatty_acid	1110	-	-
SPV_cluster_324	Cf_fatty_acid	1239	-	-
SPV_cluster_329	Cf_fatty_acid	1242	-	-
SPV_cluster_330	Cf_fatty_acid	1035	-	-
SPV_cluster_334	Cf_fatty_acid	1263	-	-
SPV_cluster_337	Cf_fatty_acid	1149	-	-
SPV_cluster_339	Cf_fatty_acid	1260	-	-
SPV_cluster_340	Cf_fatty_acid	1272	-	-
SPV_cluster_341	Cf_fatty_acid	1263	-	-
SPV_cluster_349	Cf_fatty_acid	1113	-	-
SPV_cluster_352	Cf_fatty_acid	1062	-	-
SPV_cluster_353	Cf_fatty_acid	1254	-	-
SPV_cluster_354	Cf_fatty_acid	1095	-	-
SPV_cluster_358	Cf_fatty_acid	1008	-	-
SPV_cluster_359	Cf_fatty_acid	1062	-	-
SPV_cluster_362	Cf_fatty_acid	1284	-	-
SPV_cluster_363	Cf_fatty_acid	1257	-	-
SPV_cluster_364	Cf_fatty_acid	1263	-	-
SPV_cluster_366	Cf_fatty_acid	1221	-	-
SPV_cluster_367	Cf_fatty_acid	1263	-	-
SPV_cluster_375	Cf_fatty_acid	1218	-	-
SPV_cluster_379	Cf_fatty_acid	1098	-	-
SPV_cluster_389	Cf_fatty_acid	1242	-	-
SPV_cluster_400	Cf_fatty_acid	1254	-	-
SPV_cluster_401	Cf_fatty_acid	1227	-	-
SPV_cluster_402	Cf_fatty_acid	1254	-	-
SPV_cluster_403	Cf_fatty_acid	1089	-	-
SPV_cluster_406	Cf_fatty_acid	1092	-	-
SPV_cluster_408	Cf_fatty_acid	1293	-	-
SPV_cluster_410	Cf_fatty_acid	1077	-	-

SPV_cluster_411	Cf_fatty_acid	1089	-	-
SPV_cluster_414	Cf_fatty_acid	1227	-	-
SPV_cluster_416	Cf_fatty_acid	1212	-	-
SPV_cluster_417	Cf_fatty_acid	1239	-	-
SPV_cluster_419	Cf_fatty_acid	1251	-	-
SPV_cluster_423	Cf_fatty_acid	1146	-	-
SPV_cluster_430	Cf_fatty_acid	1233	-	-
SPV_cluster_432	Cf_fatty_acid	1041	-	-
SPV_cluster_433	Cf_fatty_acid	1314	-	-
SPV_cluster_436	Cf_fatty_acid	1176	-	-
SPV_cluster_438	Cf_fatty_acid	1071	-	-
SPV_cluster_441	Cf_fatty_acid	1251	-	-
SPV_cluster_442	Cf_fatty_acid	1191	-	-
SPV_cluster_443	Cf_fatty_acid	1092	-	-
SPV_cluster_444	Cf_fatty_acid	1125	-	-
SPV_cluster_445	Cf_fatty_acid	1245	-	-
SPV_cluster_461	Cf_fatty_acid	1017	-	-
SPV_cluster_092	Ladderane	1260	-	-
SPV_cluster_125	Ladderane	1239	-	-
SPV_cluster_044	Lantipeptide	3168	-	-
SPV_cluster_061	Lantipeptide	2718	-	-
SPV_cluster_365	Lantipeptide	2688	-	-
SPV_cluster_012	Other	4239	-	-
SPV_cluster_017	Other	3618	-	-
SPV_cluster_018	Other	4284	-	-
SPV_cluster_022	Other	3084	-	-
SPV_cluster_068	Other	2793	-	-
SPV_cluster_088	Other	2586	-	-
SPV_cluster_097	Other	2871	-	-
SPV_cluster_098	Other	4452	-	-
SPV_cluster_104	Other	4272	-	-
SPV_cluster_117	Other	2064	-	-
SPV_cluster_132	Other	4272	-	-
SPV_cluster_157	Other	2943	-	-
SPV_cluster_165	Other	2676	-	-
SPV_cluster_185	Other	2910	-	-
SPV_cluster_193	Other	2787	-	-
SPV_cluster_238	Other	4326	-	-
SPV_cluster_252	Other	4371	-	-
SPV_cluster_265	Other	2802	-	-
SPV_cluster_267	Other	3087	-	-
SPV_cluster_270	Other	1983	-	-
SPV_cluster_277	Other	2247	-	-

SPV_cluster_283	Other	1527	-	-
SPV_cluster_294	Other	2898	-	-
SPV_cluster_295	Other	3534	-	-
SPV_cluster_342	Other	2556	-	-
SPV_cluster_343	Other	2574	-	-
SPV_cluster_344	Other	1740	-	-
SPV_cluster_361	Other	1389	-	-
SPV_cluster_369	Other	1023	-	-
SPV_cluster_392	Other	2340	-	-
SPV_cluster_393	Other	1896	-	-
SPV_cluster_407	Other	2016	-	-
SPV_cluster_420	Other	2049	-	-
SPV_cluster_437	Other	1557	-	-
SPV_cluster_449	Other	1347	-	-
SPV_cluster_089	Phosphonate	1680	-	-
SPV_cluster_158	Phosphonate	1629	-	-
SPV_cluster_170	Phosphonate	1626	-	-
SPV_cluster_319	Phosphonate	1635	-	-
SPV_cluster_335	Phosphonate	1479	-	-
SPV_cluster_452	Phosphonate	1284	-	-
SPV_cluster_004	Terpene	3408	-	-
SPV_cluster_006	Terpene	1035	-	-
SPV_cluster_010	Terpene	1059	-	-
SPV_cluster_011	Terpene	3462	-	-
SPV_cluster_013	Terpene	1824	-	-
SPV_cluster_019	Terpene	1083	-	-
SPV_cluster_023	Terpene	1719	-	-
SPV_cluster_041	Terpene	1719	-	-
SPV_cluster_048	Terpene	1074	-	-
SPV_cluster_050	Terpene	2079	-	-
SPV_cluster_055	Terpene	1035	-	-
SPV_cluster_056	Terpene	1044	-	-
SPV_cluster_058	Terpene	2004	-	-
SPV_cluster_059	Terpene	1050	-	-
SPV_cluster_062	Terpene	1062	-	-
SPV_cluster_063	Terpene	1068	-	-
SPV_cluster_070	Terpene	1047	-	-
SPV_cluster_072	Terpene	2166	-	-
SPV_cluster_075	Terpene	1917	-	-
SPV_cluster_077	Terpene	1737	-	-
SPV_cluster_079	Terpene	2022	-	-
SPV_cluster_080	Terpene	1011	-	-
SPV_cluster_087	Terpene	1962	-	-

SPV_cluster_095	Terpene	1692	-	-
SPV_cluster_100	Terpene	1974	-	-
SPV_cluster_103	Terpene	1098	-	-
SPV_cluster_105	Terpene	2016	-	-
SPV_cluster_110	Terpene	1023	-	-
SPV_cluster_116	Terpene	1071	-	-
SPV_cluster_118	Terpene	3405	-	-
SPV_cluster_120	Terpene	1068	-	-
SPV_cluster_127	Terpene	1704	-	-
SPV_cluster_140	Terpene	3393	-	-
SPV_cluster_142	Terpene	1068	-	-
SPV_cluster_143	Terpene	1041	-	-
SPV_cluster_144	Terpene	1020	-	-
SPV_cluster_147	Terpene	2010	-	-
SPV_cluster_156	Terpene	1068	-	-
SPV_cluster_164	Terpene	1068	-	-
SPV_cluster_175	Terpene	2031	-	-
SPV_cluster_177	Terpene	1179	-	-
SPV_cluster_183	Terpene	1047	-	-
SPV_cluster_184	Terpene	1047	-	-
SPV_cluster_186	Terpene	1260	-	-
SPV_cluster_187	Terpene	1047	-	-
SPV_cluster_188	Terpene	1095	-	-
SPV_cluster_190	Terpene	3228	-	-
SPV_cluster_196	Terpene	3426	-	-
SPV_cluster_203	Terpene	1068	-	-
SPV_cluster_223	Terpene	1050	-	-
SPV_cluster_231	Terpene	3405	-	-
SPV_cluster_232	Terpene	3357	-	-
SPV_cluster_234	Terpene	1041	-	-
SPV_cluster_242	Terpene	1935	-	-
SPV_cluster_244	Terpene	1992	-	-
SPV_cluster_247	Terpene	1017	-	-
SPV_cluster_248	Terpene	2139	-	-
SPV_cluster_249	Terpene	2001	-	-
SPV_cluster_257	Terpene	1050	-	-
SPV_cluster_272	Terpene	1995	-	-
SPV_cluster_282	Terpene	2028	-	-
SPV_cluster_286	Terpene	1935	-	-
SPV_cluster_288	Terpene	3348	-	-
SPV_cluster_298	Terpene	1473	-	-
SPV_cluster_303	Terpene	1716	-	-
SPV_cluster_304	Terpene	2037	-	-

SPV_cluster_321	Terpene	1887	-	-
SPV_cluster_325	Terpene	1995	-	-
SPV_cluster_327	Terpene	3501	-	-
SPV_cluster_328	Terpene	1353	-	-
SPV_cluster_331	Terpene	1716	-	-
SPV_cluster_332	Terpene	1050	-	-
SPV_cluster_336	Terpene	1701	-	-
SPV_cluster_351	Terpene	1068	-	-
SPV_cluster_370	Terpene	1119	-	-
SPV_cluster_372	Terpene	1674	-	-
SPV_cluster_374	Terpene	2211	-	-
SPV_cluster_381	Terpene	1134	-	-
SPV_cluster_383	Terpene	1116	-	-
SPV_cluster_385	Terpene	2850	-	-
SPV_cluster_387	Terpene	2148	-	-
SPV_cluster_388	Terpene	1926	-	-
SPV_cluster_390	Terpene	1599	-	-
SPV_cluster_395	Terpene	1098	-	-
SPV_cluster_397	Terpene	1680	-	-
SPV_cluster_399	Terpene	1212	-	-
SPV_cluster_409	Terpene	1602	-	-
SPV_cluster_415	Terpene	1569	-	-
SPV_cluster_427	Terpene	1578	-	-
SPV_cluster_429	Terpene	1629	-	-
SPV_cluster_434	Terpene	1386	-	-
SPV_cluster_435	Terpene	1473	-	-
SPV_cluster_439	Terpene	1317	-	-
SPV_cluster_453	Terpene	1194	-	-
SPV_cluster_458	Terpene	1116	-	-
SPV_cluster_462	Terpene	1113	-	-
SPV_cluster_463	Terpene	1104	-	-
SPV_cluster_051	Transatpks-Type I PKS	9711	-	-
SPV_cluster_115	Transatpks-Type I PKS	9690	-	-
SPV_cluster_001	Type I PKS	6555	-	-
SPV_cluster_005	Type I PKS	10242	-	-
SPV_cluster_008	Type I PKS	10932	-	-
SPV_cluster_020	Type I PKS	10269	-	-
SPV_cluster_029	Type I PKS	22494	-	-
SPV_cluster_030	Type I PKS	66639	-	-
SPV_cluster_035	Type I PKS	9531	-	-
SPV_cluster_040	Type I PKS	10272	-	-
SPV_cluster_047	Type I PKS	10278	-	-
SPV_cluster_049	Type I PKS	7140	-	-

SPV_cluster_052	Type I PKS	10341	-	-
SPV_cluster_057	Type I PKS	10272	-	-
SPV_cluster_065	Type I PKS	7008	-	-
SPV_cluster_067	Type I PKS	7200	-	-
SPV_cluster_071	Type I PKS	10014	-	-
SPV_cluster_078	Type I PKS	10257	-	-
SPV_cluster_082	Type I PKS	9732	-	-
SPV_cluster_084	Type I PKS	9597	-	-
SPV_cluster_091	Type I PKS	3741	-	-
SPV_cluster_096	Type I PKS	10149	-	-
SPV_cluster_102	Type I PKS	10395	-	-
SPV_cluster_106	Type I PKS	18342	-	-
SPV_cluster_108	Type I PKS	7059	-	-
SPV_cluster_111	Type I PKS	1602	-	-
SPV_cluster_114	Type I PKS	10290	-	-
SPV_cluster_134	Type I PKS	10272	-	-
SPV_cluster_141	Type I PKS	7053	-	-
SPV_cluster_146	Type I PKS	10323	-	-
SPV_cluster_149	Type I PKS	9012	-	-
SPV_cluster_150	Type I PKS	9024	-	-
SPV_cluster_151	Type I PKS	10374	-	-
SPV_cluster_153	Type I PKS	7068	-	-
SPV_cluster_154	Type I PKS	10290	-	-
SPV_cluster_161	Type I PKS	7068	-	-
SPV_cluster_163	Type I PKS	7017	-	-
SPV_cluster_166	Type I PKS	7068	-	-
SPV_cluster_167	Type I PKS	4179	-	-
SPV_cluster_168	Type I PKS	9810	-	-
SPV_cluster_169	Type I PKS	14874	-	-
SPV_cluster_171	Type I PKS	10275	-	-
SPV_cluster_172	Type I PKS	7176	-	-
SPV_cluster_174	Type I PKS	10344	-	-
SPV_cluster_181	Type I PKS	8808	-	-
SPV_cluster_191	Type I PKS	9558	-	-
SPV_cluster_194	Type I PKS	6999	-	-
SPV_cluster_202	Type I PKS	2454	-	-
SPV_cluster_204	Type I PKS	6495	-	-
SPV_cluster_206	Type I PKS	6789	-	-
SPV_cluster_208	Type I PKS	5577	-	-
SPV_cluster_210	Type I PKS	7722	-	-
SPV_cluster_211	Type I PKS	5358	-	-
SPV_cluster_212	Type I PKS	7938	-	-
SPV_cluster_213	Type I PKS	7770	-	-

SPV_cluster_214	Type I PKS	10272	-	-
SPV_cluster_220	Type I PKS	7098	-	-
SPV_cluster_221	Type I PKS	9519	-	-
SPV_cluster_225	Type I PKS	1842	-	-
SPV_cluster_226	Type I PKS	6498	-	-
SPV_cluster_227	Type I PKS	5679	-	-
SPV_cluster_237	Type I PKS	10971	-	-
SPV_cluster_241	Type I PKS	9267	-	-
SPV_cluster_245	Type I PKS	10314	-	-
SPV_cluster_246	Type I PKS	10095	-	-
SPV_cluster_253	Type I PKS	2619	-	-
SPV_cluster_260	Type I PKS	4767	-	-
SPV_cluster_264	Type I PKS	7161	-	-
SPV_cluster_266	Type I PKS	4014	-	-
SPV_cluster_268	Type I PKS	4956	-	-
SPV_cluster_269	Type I PKS	3126	-	-
SPV_cluster_273	Type I PKS	2433	-	-
SPV_cluster_278	Type I PKS	3573	-	-
SPV_cluster_281	Type I PKS	4620	-	-
SPV_cluster_287	Type I PKS	1602	-	-
SPV_cluster_291	Type I PKS	2580	-	-
SPV_cluster_292	Type I PKS	2121	-	-
SPV_cluster_299	Type I PKS	3537	-	-
SPV_cluster_300	Type I PKS	2034	-	-
SPV_cluster_302	Type I PKS	2016	-	-
SPV_cluster_305	Type I PKS	3834	-	-
SPV_cluster_306	Type I PKS	5826	-	-
SPV_cluster_309	Type I PKS	3051	-	-
SPV_cluster_310	Type I PKS	4779	-	-
SPV_cluster_311	Type I PKS	4293	-	-
SPV_cluster_314	Type I PKS	5205	-	-
SPV_cluster_315	Type I PKS	3672	-	-
SPV_cluster_323	Type I PKS	5337	-	-
SPV_cluster_326	Type I PKS	5010	-	-
SPV_cluster_333	Type I PKS	4863	-	-
SPV_cluster_338	Type I PKS	2817	-	-
SPV_cluster_347	Type I PKS	4140	-	-
SPV_cluster_348	Type I PKS	4137	-	-
SPV_cluster_356	Type I PKS	3888	-	-
SPV_cluster_357	Type I PKS	2904	-	-
SPV_cluster_360	Type I PKS	1842	-	-
SPV_cluster_368	Type I PKS	3276	-	-
SPV_cluster_371	Type I PKS	3120	-	-

SPV_cluster_373	Type I PKS	2307	-	-
SPV_cluster_377	Type I PKS	3108	-	-
SPV_cluster_378	Type I PKS	3069	-	-
SPV_cluster_382	Type I PKS	2985	-	-
SPV_cluster_384	Type I PKS	2238	-	-
SPV_cluster_386	Type I PKS	2808	-	-
SPV_cluster_391	Type I PKS	2721	-	-
SPV_cluster_394	Type I PKS	2664	-	-
SPV_cluster_396	Type I PKS	2610	-	-
SPV_cluster_405	Type I PKS	2376	-	-
SPV_cluster_412	Type I PKS	2337	-	-
SPV_cluster_413	Type I PKS	2286	-	-
SPV_cluster_418	Type I PKS	2148	-	-
SPV_cluster_421	Type I PKS	2010	-	-
SPV_cluster_422	Type I PKS	1977	-	-
SPV_cluster_425	Type I PKS	1851	-	-
SPV_cluster_426	Type I PKS	1839	-	-
SPV_cluster_428	Type I PKS	1809	-	-
SPV_cluster_440	Type I PKS	1503	-	-
SPV_cluster_446	Type I PKS	1365	-	-
SPV_cluster_447	Type I PKS	1359	-	-
SPV_cluster_448	Type I PKS	1347	-	-
SPV_cluster_450	Type I PKS	1323	-	-
SPV_cluster_451	Type I PKS	1314	-	-
SPV_cluster_454	Type I PKS	1251	-	-
SPV_cluster_455	Type I PKS	1218	-	-
SPV_cluster_456	Type I PKS	1218	-	-
SPV_cluster_457	Type I PKS	1203	-	-
SPV_cluster_459	Type I PKS	1200	-	-
SPV_cluster_380	Type II PKS	1248	-	-
SPV_cluster_090	Type III PKS	1056	-	-
SPV_cluster_131	Type III PKS	1056	-	-
SPV_cluster_218	Type III PKS	1086	-	-
SPV_cluster_404	Type III PKS	1086	-	-
SPV_cluster_460	Type III PKS	1098	-	-

"-": no significant hit

Table S5. BLAST search of the KS sequences from the metagenome of *C. reinwardti* against the KS sequences of known products from the NaPDos database

KS ID	Length (nt)	BGC ID	Identity (%)	Aligned length (aa)	E-value	NaPDos ID	Pathway product
KS_013	792	CLR_cluster_248	53	260	2.00E-77	EpoD_Q9L8C7_2mod	Epothilone
KS_029	1194	CLR_cluster_183	50	406	3.00E-103	EpoD_Q9L8C7_4mod	Epothilone
KS_030	792	CLR_cluster_249	53	260	2.00E-73	EpoD_Q9L8C7_2mod	Epothilone
KS_032	327	CLR_cluster_265	45	114	2.00E-20	VirA_BAF50727_4T	Virginiamycin
KS_039	1269	CLR_cluster_225	52	421	4.00E-99	EpoD_Q9L8C7_4mod	Epothilone
KS_041	975	CLR_cluster_202	51	320	2.00E-85	AveA2_Q9S0R7_3mod	Avermectin
KS_044	363	CLR_cluster_181	53	119	2.00E-36	EpoD_Q9L8C7_4mod	Epothilone
KS_061	1113	CLR_cluster_192	53	367	2.00E-107	EpoD_Q9L8C7_2mod	Epothilone
KS_064	1221	CLR_cluster_021	56	413	5.00E-113	EpoD_Q9L8C7_4mod	Epothilone
KS_066	702	CLR_cluster_246	55	231	7.00E-70	EpoD_Q9L8C7_4mod	Epothilone
KS_073	1112	CLR_cluster_222	52	369	9.00E-106	EpoF_Q9L8C5_1mod	Epothilone
KS_078	1064	CLR_cluster_252	56	353	1.00E-111	FabF_Bacillus_FAS	Fatty acid
KS_079	713	CLR_cluster_062	42	224	1.00E-44	TetA_BAE93722_KS1	Tetronomycin
KS_084	977	CLR_cluster_279	32	221	5.00E-24	FabF_Bacillus_FAS	Fatty acid
KS_095	1049	CLR_cluster_281	58	350	1.00E-117	FabF_Ecoli_FAS	Fatty acid
KS_101	1100	CLR_cluster_081	41	267	1.00E-42	FabF_Ecoli_FAS	Fatty acid
KS_102	1097	CLR_cluster_105	32	392	3.00E-43	FabF_Bacillus_FAS	Fatty acid
KS_105	1223	CLR_cluster_122	53	409	6.00E-122	FabF_Bacillus_FAS	Fatty acid
KS_106	1223	CLR_cluster_041	52	408	3.00E-120	FabF_Bacillus_FAS	Fatty acid
KS_111	1223	CLR_cluster_006	62	407	8.00E-138	FabF_Ecoli_FAS	Fatty acid
KS_115	1223	CLR_cluster_007	53	407	3.00E-114	FabF_Bacillus_FAS	Fatty acid
KS_116	1202	CLR_cluster_110	62	400	1.00E-141	FabB_Ecoli_FAS	Fatty acid
KS_117	1250	CLR_cluster_086	53	413	9.00E-108	FabF_Bacillus_FAS	Fatty acid
KS_120	1226	CLR_cluster_193	51	408	2.00E-105	FabF_Bacillus_FAS	Fatty acid
KS_126	1184	CLR_cluster_230	58	398	1.00E-128	FabB_Ecoli_FAS	Fatty acid
KS_134	1199	CLR_cluster_253	40	404	1.00E-86	FabF_Bacillus_FAS	Fatty acid
KS_138	1223	CLR_cluster_097	53	408	2.00E-118	FabF_Bacillus_FAS	Fatty acid
KS_142	1208	CLR_cluster_126	64	405	8.00E-134	FabB_Ecoli_FAS	Fatty acid

KS_146	1223	CLR_cluster_082	61	407	1.00E-132	FabF_Ecoli_FAS	Fatty acid
KS_150	1196	CLR_cluster_127	63	402	2.00E-117	FabB_Ecoli_FAS	Fatty acid
KS_154	1223	CLR_cluster_013	58	407	6.00E-140	FabF_Bacillus_FAS	Fatty acid
KS_159	1223	CLR_cluster_101	53	408	3.00E-113	FabF_Bacillus_FAS	Fatty acid
KS_163	1229	CLR_cluster_065	54	410	7.00E-110	FabF_Bacillus_FAS	Fatty acid
KS_170	1217	CLR_cluster_085	51	407	1.00E-123	FabF_Bacillus_FAS	Fatty acid
KS_179	1202	CLR_cluster_257	60	402	1.00E-125	FabB_Ecoli_FAS	Fatty acid
KS_183	1223	CLR_cluster_165	54	407	5.00E-122	FabF_Bacillus_FAS	Fatty acid
KS_196	1280	CLR_cluster_139	27	434	2.00E-25	FabF_Ecoli_FAS	Fatty acid
KS_198	1223	CLR_cluster_001	61	408	2.00E-117	FabF_Ecoli_FAS	Fatty acid
KS_199	1157	CLR_cluster_264	43	406	7.00E-72	FabF_Bacillus_FAS	Fatty acid
KS_201	1226	CLR_cluster_022	51	408	2.00E-114	FabF_Bacillus_FAS	Fatty acid
KS_204	1163	CLR_cluster_064	61	383	2.00E-119	FabF_Ecoli_FAS	Fatty acid
KS_206	1226	CLR_cluster_143	56	408	1.00E-113	FabF_Ecoli_FAS	Fatty acid
KS_208	1223	CLR_cluster_179	56	405	6.00E-111	FabF_Bacillus_FAS	Fatty acid
KS_212	1217	CLR_cluster_190	51	407	1.00E-123	FabF_Bacillus_FAS	Fatty acid
KS_217	1217	CLR_cluster_015	62	405	3.00E-139	FabF_Ecoli_FAS	Fatty acid
KS_221	1064	CLR_cluster_102	46	272	1.00E-57	FabF_Ecoli_FAS	Fatty acid
KS_222	1229	CLR_cluster_134	46	411	3.00E-80	FabF_Bacillus_FAS	Fatty acid
KS_227	1217	CLR_cluster_220	59	406	3.00E-134	FabF_Ecoli_FAS	Fatty acid
KS_231	1217	CLR_cluster_049	61	407	9.00E-135	FabF_Ecoli_FAS	Fatty acid
KS_232	1223	CLR_cluster_224	60	407	3.00E-135	FabF_Ecoli_FAS	Fatty acid
KS_239	1226	CLR_cluster_166	57	408	3.00E-114	FabF_Ecoli_FAS	Fatty acid
KS_247	1223	CLR_cluster_162	61	407	6.00E-127	FabF_Ecoli_FAS	Fatty acid
KS_250	1340	CLR_cluster_014	49	451	1.00E-89	EpoE_Q9L8C6_1mod	Epothilone
KS_253	1229	CLR_cluster_228	51	409	1.00E-114	FabF_Bacillus_FAS	Fatty acid
KS_262	1220	CLR_cluster_045	55	406	5.00E-122	FabF_Bacillus_FAS	Fatty acid
KS_263	1292	CLR_cluster_076	39	443	1.00E-69	EpoD_Q9L8C7_2mod	Epothilone
KS_266	1151	CLR_cluster_140	44	405	9.00E-76	FabF_Bacillus_FAS	Fatty acid
KS_279	1229	CLR_cluster_147	55	409	1.00E-119	FabF_Bacillus_FAS	Fatty acid
KS_283	1286	CLR_cluster_131	52	423	4.00E-118	EpoD_Q9L8C7_4mod	Epothilone
KS_284	1292	CLR_cluster_108	37	438	2.00E-67	EpoD_Q9L8C7_2mod	Epothilone

KS_294	1223	CLR_cluster_060	55	407	5.00E-132	FabF_Bacillus_FAS	Fatty acid
KS_302	1154	CLR_cluster_043	24	366	9.00E+00	FabF_Bacillus_FAS	Fatty acid
KS_309	1160	CLR_cluster_114	45	354	8.00E-77	FabF_Bacillus_FAS	Fatty acid
KS_311	1259	CLR_cluster_137	33	424	4.00E-44	FabB_Ecoli_FAS	Fatty acid
KS_314	1160	CLR_cluster_214	45	354	2.00E-73	FabF_Bacillus_FAS	Fatty acid
KS_322	1214	CLR_cluster_098	46	404	7.00E-84	FabF_Bacillus_FAS	Fatty acid
KS_325	1286	CLR_cluster_056	48	433	8.00E-105	EpoD_Q9L8C7_2mod	Epothilone
KS_327	1154	CLR_cluster_009	25	409	5.00E-28	FabF_Bacillus_FAS	Fatty acid
KS_329	1160	CLR_cluster_069	43	406	3.00E-71	FabF_Bacillus_FAS	Fatty acid
KS_330	1223	CLR_cluster_201	57	408	1.00E-121	FabF_Bacillus_FAS	Fatty acid
KS_334	1229	CLR_cluster_079	53	410	6.00E-106	FabF_Bacillus_FAS	Fatty acid
KS_338	1292	CLR_cluster_133	39	441	1.00E-74	EpoD_Q9L8C7_2mod	Epothilone
KS_340	1154	CLR_cluster_118	43	406	6.00E-65	FabF_Bacillus_FAS	Fatty acid
KS_342	1292	CLR_cluster_089	39	440	1.00E-65	HSAF_ABL86391_i	HSAF (heat stable antifungal factor)
KS_346	1229	CLR_cluster_096	53	410	1.00E-116	FabF_Bacillus_FAS	Fatty acid
KS_354	1232	CLR_cluster_231	51	410	7.00E-120	FabF_Bacillus_FAS	Fatty acid
KS_358	1292	CLR_cluster_055	36	414	1.00E-61	EpoD_Q9L8C7_2mod	Epothilone
KS_364	1292	CLR_cluster_027	34	440	2.00E-66	FUMON_AAD43562_i	Fumonisin
KS_366	1229	CLR_cluster_025	51	414	1.00E-122	FabF_Bacillus_FAS	Fatty acid
KS_368	1247	CLR_cluster_204	53	414	1.00E-121	FabF_Bacillus_FAS	Fatty acid
KS_372	1298	CLR_cluster_132	39	441	3.00E-71	EpoD_Q9L8C7_2mod	Epothilone
KS_373	1220	CLR_cluster_030	59	408	3.00E-130	FabF_Bacillus_FAS	Fatty acid
KS_375	1100	CLR_cluster_032	40	261	3.00E-39	FabF_Ecoli_FAS	Fatty acid
KS_376	1007	CLR_cluster_275	46	334	2.00E-87	FabF_Bacillus_FAS	Fatty acid
KS_382	1292	CLR_cluster_087	38	441	2.00E-71	EpoD_Q9L8C7_2mod	Epothilone
KS_384	1220	CLR_cluster_261	50	408	4.00E-116	FabF_Bacillus_FAS	Fatty acid
KS_393	1247	CLR_cluster_200	51	416	1.00E-118	FabF_Bacillus_FAS	Fatty acid
KS_395	995	CLR_cluster_262	53	328	1.00E-91	FabF_Bacillus_FAS	Fatty acid
KS_403	1286	CLR_cluster_135	51	432	2.00E-113	EpoD_Q9L8C7_4mod	Epothilone
KS_407	1184	CLR_cluster_010	33	406	7.00E-46	FabF_Bacillus_FAS	Fatty acid
KS_412	1223	CLR_cluster_191	50	408	2.00E-113	FabF_Bacillus_FAS	Fatty acid
KS_413	1184	CLR_cluster_042	32	406	3.00E-39	FabF_Bacillus_FAS	Fatty acid

KS_414	1292	CLR_cluster_106	38	437	2.00E-63	FUMON_AAD43562_i	Fumonisin
KS_417	965	CLR_cluster_251	43	320	2.00E-55	FabF_Bacillus_FAS	Fatty acid
KS_419	1286	CLR_cluster_212	50	438	5.00E-114	EpoD_Q9L8C7_2mod	Epothilone
KS_421	710	CLR_cluster_278	51	237	7.00E-68	EpoD_Q9L8C7_1mod	Epothilone
KS_423	1064	CLR_cluster_218	56	354	3.00E-97	EpoD_Q9L8C7_4mod	Epothilone
KS_424	1211	CLR_cluster_104	55	399	2.00E-117	FabF_Bacillus_FAS	Fatty acid
KS_428	1292	CLR_cluster_053	39	441	2.00E-73	EpoD_Q9L8C7_2mod	Epothilone
KS_431	1235	CLR_cluster_184	55	412	9.00E-115	FabF_Bacillus_FAS	Fatty acid
KS_440	1286	CLR_cluster_219	47	437	2.00E-100	EpoF_Q9L8C5_1mod	Epothilone
KS_447	1292	CLR_cluster_177	39	439	4.00E-67	TetA_BAE93722_KS1	Tetronomycin
KS_454	1184	CLR_cluster_121	60	393	5.00E-123	FabF_Ecoli_FAS	Fatty acid
KS_455	1286	CLR_cluster_203	50	431	7.00E-104	EpoD_Q9L8C7_2mod	Epothilone
KS_461	1211	CLR_cluster_145	52	401	5.00E-117	FabF_Bacillus_FAS	Fatty acid
KS_465	983	CLR_cluster_141	53	327	1.00E-84	FabF_Bacillus_FAS	Fatty acid
KS_466	1190	CLR_cluster_128	51	398	1.00E-106	FabF_Bacillus_FAS	Fatty acid
KS_473	1175	CLR_cluster_012	61	391	7.00E-128	FabF_Ecoli_FAS	Fatty acid
KS_479	1184	CLR_cluster_112	57	394	2.00E-131	FabF_Bacillus_FAS	Fatty acid
KS_483	1112	CLR_cluster_017	45	358	3.00E-64	FabF_Bacillus_FAS	Fatty acid
KS_488	1076	CLR_cluster_277	57	359	6.00E-101	FabF_Bacillus_FAS	Fatty acid
KS_503	1286	CLR_cluster_174	51	434	2.00E-105	EpoD_Q9L8C7_4mod	Epothilone
KS_504	1157	CLR_cluster_176	42	385	7.00E-72	TetA_BAE93722_KS1	Tetronomycin
KS_505	1286	CLR_cluster_005	48	433	3.00E-109	EpoD_Q9L8C7_2mod	Epothilone
KS_508	1292	CLR_cluster_107	38	444	2.00E-66	HSAF_ABL86391_i	HSAF (heat stable antifungal factor)
KS_514	1184	CLR_cluster_072	55	394	5.00E-133	FabF_Bacillus_FAS	Fatty acid
KS_518	1244	CLR_cluster_144	51	426	2.00E-105	EpoD_Q9L8C7_2mod	Epothilone
KS_521	1220	CLR_cluster_156	50	404	4.00E-84	FabF_Ecoli_FAS	Fatty acid
KS_527	1286	CLR_cluster_149	52	428	9.00E-116	EpoD_Q9L8C7_2mod	Epothilone
KS_528	1292	CLR_cluster_024	49	429	3.00E-110	EpoD_Q9L8C7_2mod	Epothilone
KS_536	1292	CLR_cluster_029	49	439	3.00E-113	EpoD_Q9L8C7_2mod	Epothilone
KS_537	1286	CLR_cluster_153	51	431	3.00E-107	EpoF_Q9L8C5_1mod	Epothilone
KS_540	1286	CLR_cluster_040	48	434	9.00E-106	EpoD_Q9L8C7_4mod	Epothilone
KS_546	1286	CLR_cluster_159	50	434	7.00E-114	EpoD_Q9L8C7_2mod	Epothilone

KS_549	1283	CLR_cluster_123	52	429	2.00E-117	EpoD_Q9L8C7_4mod	Epothilone
KS_550	1277	CLR_cluster_066	53	425	9.00E-120	EpoD_Q9L8C7_4mod	Epothilone
KS_557	1301	CLR_cluster_194	51	432	1.00E-111	EpoD_Q9L8C7_4mod	Epothilone
KS_558	1250	CLR_cluster_059	53	428	2.00E-104	AveA2_Q9S0R7_4mod	Avermectin
KS_563	1292	CLR_cluster_034	49	428	7.00E-115	EpoF_Q9L8C5_1mod	Epothilone
KS_564	1286	CLR_cluster_124	47	439	1.00E-104	EpoD_Q9L8C7_2mod	Epothilone
KS_576	1094	CLR_cluster_232	44	363	3.00E-87	FabF_Bacillus_FAS	Fatty acid
KS_579	1178	CLR_cluster_008	36	405	4.00E-53	FabF_Bacillus_FAS	Fatty acid
KS_580	1010	CLR_cluster_070	35	209	6.00E-31	FabF_Bacillus_FAS	Fatty acid
KS_585	989	CLR_cluster_221	35	260	9.00E-38	FabF_Bacillus_FAS	Fatty acid
KS_586	1184	CLR_cluster_044	35	376	8.00E-52	FabF_Bacillus_FAS	Fatty acid
KS_590	1094	CLR_cluster_020	59	365	1.00E-115	TetF_BAE93731_2mod	Tetronomycin
KS_591	1283	CLR_cluster_026	53	428	5.00E-121	EpoD_Q9L8C7_4mod	Epothilone
KS_602	722	CLR_cluster_247	40	245	3.00E-40	TylGI_O33954_KS1	Tylosin
KS_605	851	CLR_cluster_109	25	269	1.00E+00	FabF_Bacillus_FAS	Fatty acid
KS_607	653	CLR_cluster_068	59	215	8.00E-70	EpoD_Q9L8C7_1mod	Epothilone
KS_608	599	CLR_cluster_234	61	196	2.00E-61	EpoA_Q9L8C9_mod	Epothilone
KS_609	545	CLR_cluster_163	56	178	4.00E-52	EpoD_Q9L8C7_4mod	Epothilone
KS_611	449	CLR_cluster_235	45	155	8.00E-29	VirA_BAF50727_4T	Virginiamycin
KS_613	362	CLR_cluster_180	53	119	2.00E-36	EpoD_Q9L8C7_4mod	Epothilone
KS_615	1262	CLR_cluster_197	50	428	7.00E-106	EpoD_Q9L8C7_4mod	Epothilone
KS_616	341	CLR_cluster_245	59	111	3.00E-34	NysI_Q9L4X3_5mod	Nystatin
KS_617	233	CLR_cluster_250	53	74	3.00E-21	EpoD_Q9L8C7_4mod	Epothilone
KS_618	233	CLR_cluster_255	51	74	1.00E-20	EpoD_Q9L8C7_4mod	Epothilone
KS_619	245	CLR_cluster_268	48	80	7.00E-21	EpoD_Q9L8C7_4mod	Epothilone
KS_620	1259	CLR_cluster_125	53	423	7.00E-116	EpoD_Q9L8C7_4mod	Epothilone
KS_626	1265	CLR_cluster_136	51	427	2.00E-103	EpoD_Q9L8C7_4mod	Epothilone
KS_630	1259	CLR_cluster_175	54	423	8.00E-119	EpoD_Q9L8C7_4mod	Epothilone
KS_633	1265	CLR_cluster_093	54	424	3.00E-119	EpoD_Q9L8C7_4mod	Epothilone
KS_637	1262	CLR_cluster_160	54	424	8.00E-121	EpoD_Q9L8C7_4mod	Epothilone
KS_640	1259	CLR_cluster_154	50	425	1.00E-113	EpoD_Q9L8C7_2mod	Epothilone
KS_644	1256	CLR_cluster_026	52	435	5.00E-113	TetF_BAE93731_2mod	Tetronomycin

KS_645	1259	CLR_cluster_161	49	424	1.00E-107	EpoD_Q9L8C7_4mod	Epothilone
KS_646	1256	CLR_cluster_117	51	422	5.00E-99	EpoD_Q9L8C7_4mod	Epothilone
KS_649	1259	CLR_cluster_080	49	422	7.00E-104	EpoD_Q9L8C7_4mod	Epothilone
KS_654	1244	CLR_cluster_058	51	436	3.00E-107	EpoD_Q9L8C7_2mod	Epothilone
KS_659	1259	CLR_cluster_047	53	421	8.00E-119	EpoF_Q9L8C5_1mod	Epothilone
KS_661	1265	CLR_cluster_103	48	428	2.00E-101	EpoD_Q9L8C7_2mod	Epothilone
KS_669	1235	CLR_cluster_208	50	418	1.00E-106	EpoD_Q9L8C7_4mod	Epothilone
KS_671	1259	CLR_cluster_048	52	424	9.00E-116	EpoD_Q9L8C7_4mod	Epothilone
KS_674	1268	CLR_cluster_020	55	421	2.00E-115	EpoF_Q9L8C5_1mod	Epothilone
KS_681	1277	CLR_cluster_075	52	424	4.00E-117	EpoD_Q9L8C7_4mod	Epothilone
KS_683	1268	CLR_cluster_075	56	424	3.00E-120	EpoD_Q9L8C7_4mod	Epothilone
KS_688	1259	CLR_cluster_068	52	423	7.00E-115	EpoD_Q9L8C7_4mod	Epothilone
KS_695	1259	CLR_cluster_056	53	428	3.00E-115	EpoD_Q9L8C7_2mod	Epothilone
KS_701	1271	CLR_cluster_173	54	427	8.00E-120	TetE_BAE93730_1mod	Tetronomycin
KS_705	1259	CLR_cluster_206	48	424	1.00E-111	EpoD_Q9L8C7_4mod	Epothilone
KS_708	1262	CLR_cluster_242	54	425	7.00E-113	EpoD_Q9L8C7_4mod	Epothilone
KS_720	1259	CLR_cluster_155	49	427	7.00E-109	EpoD_Q9L8C7_2mod	Epothilone
KS_726	1259	CLR_cluster_005	50	423	2.00E-112	EpoD_Q9L8C7_4mod	Epothilone
KS_728	1259	CLR_cluster_057	50	422	2.00E-107	EpoD_Q9L8C7_4mod	Epothilone
KS_730	1265	CLR_cluster_040	52	424	2.00E-101	EpoD_Q9L8C7_4mod	Epothilone
KS_732	1262	CLR_cluster_034	49	425	2.00E-102	EpoD_Q9L8C7_4mod	Epothilone
KS_747	1109	CLR_cluster_146	56	367	2.00E-108	TetF_BAE93731_2mod	Tetronomycin
KS_749	1319	CLR_cluster_050	48	444	6.00E-108	EpoD_Q9L8C7_4mod	Epothilone
KS_751	224	CLR_cluster_130	54	72	1.00E+00	RapA_Q54297_2mod	Rapamycin
KS_755	1262	CLR_cluster_047	54	425	5.00E-122	EpoD_Q9L8C7_2mod	Epothilone
KS_757	1250	CLR_cluster_020	53	428	4.00E-116	EpoD_Q9L8C7_2mod	Epothilone
KS_761	1277	CLR_cluster_026	51	424	5.00E-112	NysJ_Q9L4X2_2mod	Nystatin

Table S6. BLAST search of the KS sequences from the metagenome of *R. globostellata* against the KS sequences of known products from the NaPDos database

KS ID	Length (nt)	BGC ID	Identity (%)	Aligned length (aa)	E-value	NaPDos ID	Pathway product
KS_001	696	RHG_cluster_258	59	229	9.00E-71	EpoD_Q9L8C7_4mod	Epothilone
KS_004	1185	RHG_cluster_332	47	398	1.00E-82	FabF_Bacillus_FAS	Fatty acid
KS_006	1128	RHG_cluster_316	51	364	5.00E-97	NysJ_Q9L4X2_2mod	Nystatin
KS_007	1059	RHG_cluster_341	38	350	5.00E-58	TetA_BAE93722_KS1	Tetronomycin
KS_010	1176	RHG_cluster_342	56	378	2.00E-111	EpoD_Q9L8C7_4mod	Epothilone
KS_011	801	RHG_cluster_340	52	272	2.00E-72	Strep_ZP_06279092_i	Unkown
KS_012	429	RHG_cluster_282	58	139	1.00E-44	NysC_Q9L4W3_2mod	Nystatin
KS_014	1086	RHG_cluster_275	47	361	5.00E-94	FabF_Bacillus_FAS	Fatty acid
KS_016	1239	RHG_cluster_191	52	414	2.00E-91	EpoF_Q9L8C5_1mod	Epothilone
KS_019	1017	RHG_cluster_352	63	341	2.00E-113	FabB_Ecoli_FAS	Fatty acid
KS_020	705	RHG_cluster_308	57	229	2.00E-72	EpoD_Q9L8C7_2mod	Epothilone
KS_021	771	RHG_cluster_354	41	254	3.00E-53	FUMON_AAD43562_i	Fumonisin
KS_026	1080	RHG_cluster_321	56	359	2.00E-116	FabF_Bacillus_FAS	Fatty acid
KS_028	591	RHG_cluster_221	57	194	1.00E-60	EpoD_Q9L8C7_2mod	Epothilone
KS_033	1266	RHG_cluster_087	54	432	3.00E-117	EpoD_Q9L8C7_4mod	Epothilone
KS_036	792	RHG_cluster_345	54	258	2.00E-76	EpoD_Q9L8C7_4mod	Epothilone
KS_037	390	RHG_cluster_351	65	127	7.00E-43	EpoD_Q9L8C7_4mod	Epothilone
KS_040	1038	RHG_cluster_295	52	344	4.00E-100	EpoD_Q9L8C7_2mod	Epothilone
KS_043	540	RHG_cluster_283	44	185	2.00E-26	VirA_BAF50727_4T	Virginiamycin
KS_045	741	RHG_cluster_338	56	241	6.00E-65	EpoD_Q9L8C7_1mod	Epothilone
KS_046	354	RHG_cluster_362	44	115	3.00E+00	FUMON_AAD43562_i	Fumonisin
KS_048	1266	RHG_cluster_136	38	430	6.00E-70	EpoD_Q9L8C7_2mod	Epothilone
KS_052	285	RHG_cluster_367	52	91	5.00E-26	AveA2_Q9S0R7_3mod	Avermectin
KS_053	543	RHG_cluster_357	63	177	5.00E-60	EpoD_Q9L8C7_4mod	Epothilone
KS_054	525	RHG_cluster_356	65	172	8.00E-53	EpoD_Q9L8C7_4mod	Epothilone
KS_056	1008	RHG_cluster_355	53	336	2.00E-93	FabF_Bacillus_FAS	Fatty acid
KS_060	444	RHG_cluster_183	44	153	3.00E-29	VirA_BAF50727_4T	Virginiamycin
KS_062	657	RHG_cluster_318	55	216	3.00E-67	EpoD_Q9L8C7_2mod	Epothilone

KS_063	1230	RHG_cluster_019	52	420	8.00E-104	EpoD_Q9L8C7_2mod	Epothilone
KS_065	591	RHG_cluster_255	57	194	3.00E-60	EpoD_Q9L8C7_2mod	Epothilone
KS_067	1272	RHG_cluster_124	52	424	1.00E-112	EpoD_Q9L8C7_4mod	Epothilone
KS_069	825	RHG_cluster_168	54	272	5.00E-73	EpoD_Q9L8C7_4mod	Epothilone
KS_071	1152	RHG_cluster_294	53	364	3.00E-105	EpoD_Q9L8C7_2mod	Epothilone
KS_074	1187	RHG_cluster_151	53	396	3.00E-99	FabF_Bacillus_FAS	Fatty acid
KS_075	1136	RHG_cluster_164	54	351	4.00E-104	EpoF_Q9L8C5_1mod	Epothilone
KS_077	1097	RHG_cluster_310	65	362	7.00E-131	FabF_Ecoli_FAS	Fatty acid
KS_080	1094	RHG_cluster_343	56	365	3.00E-114	FabF_Bacillus_FAS	Fatty acid
KS_083	1130	RHG_cluster_336	49	375	2.00E-95	TetF_BAE93731_2mod	Tetronomycin
KS_088	893	RHG_cluster_279	55	292	9.00E-77	EpoA_Q9L8C9_mod	Epothilone
KS_091	977	RHG_cluster_119	56	322	1.00E-95	EpoD_Q9L8C7_2mod	Epothilone
KS_094	1097	RHG_cluster_111	32	392	3.00E-43	FabF_Bacillus_FAS	Fatty acid
KS_096	1097	RHG_cluster_116	35	385	5.00E-43	FabF_Bacillus_FAS	Fatty acid
KS_098	1100	RHG_cluster_184	41	267	1.00E-42	FabF_Ecoli_FAS	Fatty acid
KS_103	1007	RHG_cluster_365	51	335	5.00E-85	FabF_Bacillus_FAS	Fatty acid
KS_104	1223	RHG_cluster_007	62	407	8.00E-138	FabF_Ecoli_FAS	Fatty acid
KS_107	1223	RHG_cluster_018	53	407	3.00E-114	FabF_Bacillus_FAS	Fatty acid
KS_108	1217	RHG_cluster_278	63	405	1.00E-139	FabB_Ecoli_FAS	Fatty acid
KS_109	1208	RHG_cluster_153	64	405	8.00E-134	FabB_Ecoli_FAS	Fatty acid
KS_110	1202	RHG_cluster_073	62	400	1.00E-141	FabB_Ecoli_FAS	Fatty acid
KS_112	1250	RHG_cluster_127	53	413	4.00E-120	FabF_Bacillus_FAS	Fatty acid
KS_113	1259	RHG_cluster_237	48	431	7.00E-106	TylGIII_O33956_2mod	Tylosin
KS_130	1091	RHG_cluster_315	58	365	7.00E-124	FabF_Ecoli_FAS	Fatty acid
KS_131	1226	RHG_cluster_098	51	408	2.00E-105	FabF_Bacillus_FAS	Fatty acid
KS_135	1199	RHG_cluster_033	39	404	7.00E-85	FabF_Bacillus_FAS	Fatty acid
KS_137	1184	RHG_cluster_156	57	395	4.00E-124	FabB_Ecoli_FAS	Fatty acid
KS_140	1250	RHG_cluster_277	53	413	9.00E-108	FabF_Bacillus_FAS	Fatty acid
KS_143	1223	RHG_cluster_049	61	407	1.00E-132	FabF_Ecoli_FAS	Fatty acid
KS_144	1223	RHG_cluster_138	53	408	2.00E-118	FabF_Bacillus_FAS	Fatty acid
KS_148	1214	RHG_cluster_231	45	245	3.00E-50	FabF_Ecoli_FAS	Fatty acid
KS_151	1196	RHG_cluster_250	63	402	2.00E-117	FabB_Ecoli_FAS	Fatty acid

KS_153	1250	RHG_cluster_079	38	423	3.00E-61	FabF_Bacillus_FAS	Fatty acid
KS_161	1073	RHG_cluster_292	66	359	1.00E-124	FabB_Ecoli_FAS	Fatty acid
KS_162	1202	RHG_cluster_108	60	402	5.00E-125	FabB_Ecoli_FAS	Fatty acid
KS_166	1223	RHG_cluster_023	53	408	9.00E-114	FabF_Bacillus_FAS	Fatty acid
KS_167	1151	RHG_cluster_271	58	384	2.00E-119	FabF_Bacillus_FAS	Fatty acid
KS_173	1253	RHG_cluster_326	52	423	3.00E-118	EpoD_Q9L8C7_4mod	Epothilone
KS_174	1229	RHG_cluster_130	53	410	7.00E-109	FabF_Bacillus_FAS	Fatty acid
KS_175	1217	RHG_cluster_114	51	407	1.00E-123	FabF_Bacillus_FAS	Fatty acid
KS_176	1265	RHG_cluster_335	53	427	8.00E-102	EpoD_Q9L8C7_4mod	Epothilone
KS_178	1187	RHG_cluster_217	52	369	5.00E-106	EpoF_Q9L8C5_1mod	Epothilone
KS_181	1223	RHG_cluster_188	58	407	1.00E-137	FabF_Bacillus_FAS	Fatty acid
KS_182	1223	RHG_cluster_225	57	408	1.00E-134	FabF_Bacillus_FAS	Fatty acid
KS_186	1280	RHG_cluster_078	27	434	3.00E-25	FabF_Ecoli_FAS	Fatty acid
KS_188	1217	RHG_cluster_009	62	405	3.00E-139	FabF_Ecoli_FAS	Fatty acid
KS_189	1196	RHG_cluster_334	60	400	7.00E-132	FabF_Ecoli_FAS	Fatty acid
KS_191	1226	RHG_cluster_142	56	408	1.00E-113	FabF_Ecoli_FAS	Fatty acid
KS_193	1091	RHG_cluster_359	57	364	2.00E-112	FabF_Ecoli_FAS	Fatty acid
KS_195	1163	RHG_cluster_122	61	383	2.00E-119	FabF_Ecoli_FAS	Fatty acid
KS_197	1226	RHG_cluster_305	51	408	4.00E-114	FabF_Bacillus_FAS	Fatty acid
KS_202	1223	RHG_cluster_344	56	382	3.00E-112	FabF_Ecoli_FAS	Fatty acid
KS_203	1217	RHG_cluster_235	61	407	6.00E-135	FabF_Ecoli_FAS	Fatty acid
KS_205	1064	RHG_cluster_120	46	272	1.00E-57	FabF_Ecoli_FAS	Fatty acid
KS_218	1136	RHG_cluster_080	21	371	7.00E+00	FabF_Bacillus_FAS	Fatty acid
KS_220	1223	RHG_cluster_274	63	383	2.00E-133	FabF_Ecoli_FAS	Fatty acid
KS_223	1223	RHG_cluster_012	61	408	2.00E-117	FabF_Ecoli_FAS	Fatty acid
KS_224	1259	RHG_cluster_296	52	424	3.00E-107	EpoD_Q9L8C7_4mod	Epothilone
KS_225	1223	RHG_cluster_319	56	405	6.00E-111	FabF_Bacillus_FAS	Fatty acid
KS_235	1217	RHG_cluster_324	52	407	1.00E-123	FabF_Bacillus_FAS	Fatty acid
KS_236	1223	RHG_cluster_347	61	407	6.00E-127	FabF_Ecoli_FAS	Fatty acid
KS_237	1223	RHG_cluster_281	54	408	1.00E-124	FabF_Bacillus_FAS	Fatty acid
KS_240	1157	RHG_cluster_051	43	406	7.00E-72	FabF_Bacillus_FAS	Fatty acid
KS_244	1220	RHG_cluster_263	60	407	6.00E-131	FabF_Ecoli_FAS	Fatty acid

KS_249	1226	RHG_cluster_220	56	408	1.00E-113	FabF_Ecoli_FAS	Fatty acid
KS_252	1292	RHG_cluster_011	39	445	5.00E-70	EpoD_Q9L8C7_2mod	Epothilone
KS_254	1226	RHG_cluster_301	58	409	5.00E-123	FabF_Bacillus_FAS	Fatty acid
KS_256	1229	RHG_cluster_039	51	409	1.00E-114	FabF_Bacillus_FAS	Fatty acid
KS_259	1220	RHG_cluster_148	38	407	2.00E-69	FabF_Bacillus_FAS	Fatty acid
KS_261	1223	RHG_cluster_035	63	408	1.00E-141	FabF_Ecoli_FAS	Fatty acid
KS_264	1214	RHG_cluster_227	55	404	1.00E-121	FabF_Bacillus_FAS	Fatty acid
KS_267	1292	RHG_cluster_028	39	443	1.00E-69	EpoD_Q9L8C7_2mod	Epothilone
KS_269	1151	RHG_cluster_212	44	405	9.00E-76	FabF_Bacillus_FAS	Fatty acid
KS_270	1229	RHG_cluster_185	55	409	1.00E-119	FabF_Bacillus_FAS	Fatty acid
KS_278	1226	RHG_cluster_020	58	408	2.00E-136	FabF_Bacillus_FAS	Fatty acid
KS_285	1280	RHG_cluster_081	43	431	3.00E-88	FabF_Bacillus_FAS	Fatty acid
KS_290	1292	RHG_cluster_091	37	438	2.00E-67	EpoD_Q9L8C7_2mod	Epothilone
KS_292	1292	RHG_cluster_096	39	450	6.00E-75	Avi_AAK83194_i	Avilamycin
KS_295	1286	RHG_cluster_094	52	423	4.00E-118	EpoD_Q9L8C7_4mod	Epothilone
KS_296	1229	RHG_cluster_244	52	407	2.00E-102	FabF_Bacillus_FAS	Fatty acid
KS_298	1259	RHG_cluster_253	33	424	3.00E-44	FabB_Ecoli_FAS	Fatty acid
KS_299	1160	RHG_cluster_273	45	354	2.00E-73	FabF_Bacillus_FAS	Fatty acid
KS_300	1226	RHG_cluster_075	56	407	1.00E-129	FabF_Bacillus_FAS	Fatty acid
KS_304	1154	RHG_cluster_045	25	409	5.00E-28	FabF_Bacillus_FAS	Fatty acid
KS_313	1226	RHG_cluster_178	55	407	6.00E-126	FabF_Bacillus_FAS	Fatty acid
KS_316	1217	RHG_cluster_264	44	404	1.00E-81	FabF_Bacillus_FAS	Fatty acid
KS_318	1214	RHG_cluster_030	46	404	7.00E-84	FabF_Bacillus_FAS	Fatty acid
KS_319	1160	RHG_cluster_259	45	357	9.00E-77	FabF_Bacillus_FAS	Fatty acid
KS_320	1286	RHG_cluster_215	47	437	2.00E-100	EpoF_Q9L8C5_1mod	Epothilone
KS_324	1223	RHG_cluster_313	57	408	1.00E-121	FabF_Bacillus_FAS	Fatty acid
KS_328	1154	RHG_cluster_133	24	366	9.00E+00	FabF_Bacillus_FAS	Fatty acid
KS_331	1160	RHG_cluster_085	43	406	3.00E-71	FabF_Bacillus_FAS	Fatty acid
KS_333	1286	RHG_cluster_165	48	433	8.00E-105	EpoD_Q9L8C7_2mod	Epothilone
KS_335	1298	RHG_cluster_302	39	441	3.00E-71	EpoD_Q9L8C7_2mod	Epothilone
KS_343	1229	RHG_cluster_222	52	410	1.00E-110	FabF_Bacillus_FAS	Fatty acid
KS_344	1292	RHG_cluster_016	39	440	1.00E-65	HSAF_ABL86391_i	HSAF (heat stable antifungal factor)

KS_347	1292	RHG_cluster_034	34	440	2.00E-66	FUMON_AAD43562_i	Fumonisin
KS_348	1226	RHG_cluster_348	49	408	6.00E-106	FabF_Bacillus_FAS	Fatty acid
KS_352	1229	RHG_cluster_004	51	414	1.00E-122	FabF_Bacillus_FAS	Fatty acid
KS_355	1154	RHG_cluster_236	43	406	6.00E-65	FabF_Bacillus_FAS	Fatty acid
KS_359	1292	RHG_cluster_206	38	430	3.00E-70	RapC_Q54299_2mod	Rapamycin
KS_365	1220	RHG_cluster_031	59	408	1.00E-129	FabF_Bacillus_FAS	Fatty acid
KS_367	1229	RHG_cluster_084	53	410	6.00E-106	FabF_Bacillus_FAS	Fatty acid
KS_374	1184	RHG_cluster_360	47	394	1.00E-93	FabF_Bacillus_FAS	Fatty acid
KS_377	1229	RHG_cluster_150	48	408	1.00E-110	FabF_Bacillus_FAS	Fatty acid
KS_380	1292	RHG_cluster_135	38	441	2.00E-71	EpoD_Q9L8C7_2mod	Epothilone
KS_387	1292	RHG_cluster_067	39	441	1.00E-74	EpoD_Q9L8C7_2mod	Epothilone
KS_388	1229	RHG_cluster_317	52	410	4.00E-119	FabF_Bacillus_FAS	Fatty acid
KS_390	1223	RHG_cluster_172	59	406	9.00E-132	FabF_Bacillus_FAS	Fatty acid
KS_392	1247	RHG_cluster_097	51	416	1.00E-119	FabF_Bacillus_FAS	Fatty acid
KS_396	1292	RHG_cluster_269	39	439	2.00E-74	EpoD_Q9L8C7_2mod	Epothilone
KS_397	1292	RHG_cluster_202	38	444	3.00E-67	HSAF_ABL86391_i	HSAF (heat stable antifungal factor)
KS_398	1286	RHG_cluster_167	48	433	1.00E-104	EpoD_Q9L8C7_2mod	Epothilone
KS_400	1184	RHG_cluster_132	32	406	3.00E-39	FabF_Bacillus_FAS	Fatty acid
KS_406	1184	RHG_cluster_044	33	406	7.00E-46	FabF_Bacillus_FAS	Fatty acid
KS_408	1292	RHG_cluster_128	39	435	5.00E-68	TetA_BAE93722_KS1	Tetronomycin
KS_410	1286	RHG_cluster_037	51	432	2.00E-113	EpoD_Q9L8C7_4mod	Epothilone
KS_411	1223	RHG_cluster_214	50	408	2.00E-113	FabF_Bacillus_FAS	Fatty acid
KS_429	1292	RHG_cluster_040	50	434	2.00E-112	EpoD_Q9L8C7_2mod	Epothilone
KS_430	1292	RHG_cluster_109	39	441	1.00E-72	EpoD_Q9L8C7_2mod	Epothilone
KS_437	1235	RHG_cluster_077	55	412	1.00E-114	FabF_Bacillus_FAS	Fatty acid
KS_439	1211	RHG_cluster_086	55	399	2.00E-117	FabF_Bacillus_FAS	Fatty acid
KS_446	1289	RHG_cluster_069	48	438	1.00E-101	EpoD_Q9L8C7_2mod	Epothilone
KS_448	1292	RHG_cluster_209	39	439	4.00E-67	TetA_BAE93722_KS1	Tetronomycin
KS_449	1160	RHG_cluster_241	59	389	2.00E-123	FabB_Ecoli_FAS	Fatty acid
KS_452	1286	RHG_cluster_249	52	435	2.00E-113	EpoD_Q9L8C7_2mod	Epothilone
KS_453	1286	RHG_cluster_174	50	431	7.00E-104	EpoD_Q9L8C7_2mod	Epothilone
KS_459	1184	RHG_cluster_218	60	393	2.00E-123	FabF_Ecoli_FAS	Fatty acid

KS_460	1145	RHG_cluster_074	39	386	3.00E-49	FabF_Bacillus_FAS	Fatty acid
KS_462	1190	RHG_cluster_143	51	398	1.00E-106	FabF_Bacillus_FAS	Fatty acid
KS_468	1175	RHG_cluster_068	61	391	7.00E-128	FabF_Ecoli_FAS	Fatty acid
KS_469	1286	RHG_cluster_100	47	437	2.00E-99	EpoF_Q9L8C5_1mod	Epothilone
KS_471	1175	RHG_cluster_144	60	391	4.00E-131	FabF_Ecoli_FAS	Fatty acid
KS_474	1211	RHG_cluster_216	51	401	3.00E-116	FabF_Bacillus_FAS	Fatty acid
KS_476	1133	RHG_cluster_061	38	356	5.00E-52	FabB_Ecoli_FAS	Fatty acid
KS_481	1154	RHG_cluster_303	60	385	6.00E-127	FabB_Ecoli_FAS	Fatty acid
KS_484	1298	RHG_cluster_298	50	434	3.00E-106	EpoD_Q9L8C7_4mod	Epothilone
KS_486	1112	RHG_cluster_059	45	358	3.00E-64	FabF_Bacillus_FAS	Fatty acid
KS_490	1292	RHG_cluster_213	40	440	3.00E-75	EpoD_Q9L8C7_4mod	Epothilone
KS_491	1286	RHG_cluster_154	47	434	6.00E-106	EpoD_Q9L8C7_2mod	Epothilone
KS_494	1292	RHG_cluster_072	39	435	6.00E-68	TetA_BAE93722_KS1	Tetronomycin
KS_495	1292	RHG_cluster_272	39	442	6.00E-78	EpoD_Q9L8C7_2mod	Epothilone
KS_497	1286	RHG_cluster_163	51	434	2.00E-105	EpoD_Q9L8C7_4mod	Epothilone
KS_498	1286	RHG_cluster_322	47	437	1.00E-100	EpoF_Q9L8C5_1mod	Epothilone
KS_499	1094	RHG_cluster_265	53	365	3.00E-114	FabF_Bacillus_FAS	Fatty acid
KS_501	1286	RHG_cluster_204	49	439	3.00E-110	EpoD_Q9L8C7_2mod	Epothilone
KS_506	1286	RHG_cluster_179	53	430	6.00E-104	EpoD_Q9L8C7_4mod	Epothilone
KS_507	1286	RHG_cluster_057	48	433	3.00E-109	EpoD_Q9L8C7_2mod	Epothilone
KS_511	1292	RHG_cluster_090	52	432	9.00E-116	EpoD_Q9L8C7_4mod	Epothilone
KS_516	1292	RHG_cluster_162	49	433	2.00E-111	EpoD_Q9L8C7_2mod	Epothilone
KS_517	1220	RHG_cluster_155	50	404	4.00E-84	FabF_Ecoli_FAS	Fatty acid
KS_519	1184	RHG_cluster_065	55	394	5.00E-133	FabF_Bacillus_FAS	Fatty acid
KS_520	1286	RHG_cluster_195	48	429	9.00E-111	EpoD_Q9L8C7_2mod	Epothilone
KS_525	1286	RHG_cluster_192	50	430	1.00E-114	EpoD_Q9L8C7_2mod	Epothilone
KS_526	1148	RHG_cluster_103	61	384	1.00E-124	FabF_Ecoli_FAS	Fatty acid
KS_529	1292	RHG_cluster_194	49	429	3.00E-110	EpoD_Q9L8C7_2mod	Epothilone
KS_532	1286	RHG_cluster_266	52	428	6.00E-116	EpoD_Q9L8C7_2mod	Epothilone
KS_538	1286	RHG_cluster_160	50	429	1.00E-113	EpoD_Q9L8C7_2mod	Epothilone
KS_541	1286	RHG_cluster_027	48	434	9.00E-106	EpoD_Q9L8C7_4mod	Epothilone
KS_543	1286	RHG_cluster_054	51	431	3.00E-107	EpoF_Q9L8C5_1mod	Epothilone

KS_545	1292	RHG_cluster_242	49	439	6.00E-113	EpoD_Q9L8C7_2mod	Epothilone
KS_548	1277	RHG_cluster_159	53	426	2.00E-120	EpoD_Q9L8C7_4mod	Epothilone
KS_551	1292	RHG_cluster_147	49	428	7.00E-115	EpoF_Q9L8C5_1mod	Epothilone
KS_553	1250	RHG_cluster_053	53	428	2.00E-104	AveA2_Q9S0R7_4mod	Avermectin
KS_554	1283	RHG_cluster_008	52	429	2.00E-117	EpoD_Q9L8C7_4mod	Epothilone
KS_555	1286	RHG_cluster_177	47	439	1.00E-104	EpoD_Q9L8C7_2mod	Epothilone
KS_559	1301	RHG_cluster_285	51	432	2.00E-111	EpoD_Q9L8C7_4mod	Epothilone
KS_569	1292	RHG_cluster_238	48	440	4.00E-109	EpoD_Q9L8C7_2mod	Epothilone
KS_570	1073	RHG_cluster_095	33	362	2.00E-39	FabF_Bacillus_FAS	Fatty acid
KS_572	1286	RHG_cluster_288	48	441	2.00E-110	EpoD_Q9L8C7_2mod	Epothilone
KS_573	1292	RHG_cluster_056	50	433	6.00E-112	EpoD_Q9L8C7_2mod	Epothilone
KS_575	1067	RHG_cluster_232	31	314	4.00E-37	FabF_Bacillus_FAS	Fatty acid
KS_577	1178	RHG_cluster_046	36	405	4.00E-53	FabF_Bacillus_FAS	Fatty acid
KS_581	1010	RHG_cluster_121	35	209	6.00E-31	FabF_Bacillus_FAS	Fatty acid
KS_583	1184	RHG_cluster_134	35	376	8.00E-52	FabF_Bacillus_FAS	Fatty acid
KS_588	1016	RHG_cluster_125	37	260	6.00E-38	FabF_Bacillus_FAS	Fatty acid
KS_593	1283	RHG_cluster_306	53	428	5.00E-121	EpoD_Q9L8C7_4mod	Epothilone
KS_596	1259	RHG_cluster_261	49	424	2.00E-104	EpoD_Q9L8C7_4mod	Epothilone
KS_599	1079	RHG_cluster_182	28	336	2.00E+00	FabF_Bacillus_FAS	Fatty acid
KS_603	851	RHG_cluster_270	27	259	2.00E+00	FabB_Streptomyces_FAS	Fatty acid
KS_614	1259	RHG_cluster_262	50	427	1.00E-109	EpoD_Q9L8C7_4mod	Epothilone
KS_621	1259	RHG_cluster_208	53	423	7.00E-116	EpoD_Q9L8C7_4mod	Epothilone
KS_625	1262	RHG_cluster_289	50	428	5.00E-106	EpoD_Q9L8C7_4mod	Epothilone
KS_627	1265	RHG_cluster_162	51	427	2.00E-103	EpoD_Q9L8C7_4mod	Epothilone
KS_628	1277	RHG_cluster_248	50	433	4.00E-112	EpoD_Q9L8C7_2mod	Epothilone
KS_636	1262	RHG_cluster_119	54	424	8.00E-121	EpoD_Q9L8C7_4mod	Epothilone
KS_638	1259	RHG_cluster_226	50	426	2.00E-108	EpoD_Q9L8C7_4mod	Epothilone
KS_642	713	RHG_cluster_140	60	235	7.00E-74	EpoD_Q9L8C7_1mod	Epothilone
KS_643	1256	RHG_cluster_233	52	435	5.00E-113	TetF_BAE93731_2mod	Tetronomycin
KS_647	1256	RHG_cluster_229	51	422	3.00E-99	EpoD_Q9L8C7_4mod	Epothilone
KS_650	1235	RHG_cluster_191	55	423	8.00E-107	EpoD_Q9L8C7_4mod	Epothilone
KS_652	1259	RHG_cluster_268	49	427	2.00E-103	TetF_BAE93731_2mod	Tetronomycin

KS_655	1244	RHG_cluster_203	51	436	3.00E-107	EpoD_Q9L8C7_2mod	Epothilone
KS_657	1259	RHG_cluster_140	53	421	8.00E-119	EpoF_Q9L8C5_1mod	Epothilone
KS_660	1259	RHG_cluster_197	50	423	2.00E-112	EpoD_Q9L8C7_4mod	Epothilone
KS_662	1265	RHG_cluster_062	48	428	2.00E-101	EpoD_Q9L8C7_2mod	Epothilone
KS_664	1277	RHG_cluster_145	51	424	4.00E-112	NysJ_Q9L4X2_2mod	Nystatin
KS_666	1265	RHG_cluster_246	52	428	3.00E-116	EpoD_Q9L8C7_2mod	Epothilone
KS_668	1259	RHG_cluster_240	50	426	3.00E-110	EpoD_Q9L8C7_4mod	Epothilone
KS_670	1259	RHG_cluster_014	52	424	9.00E-116	EpoD_Q9L8C7_4mod	Epothilone
KS_672	1262	RHG_cluster_198	48	429	4.00E-106	TetE_BAE93730_1mod	Tetronomycin
KS_673	1268	RHG_cluster_119	55	421	2.00E-115	EpoF_Q9L8C5_1mod	Epothilone
KS_677	1259	RHG_cluster_149	51	427	1.00E-113	EpoD_Q9L8C7_2mod	Epothilone
KS_679	1253	RHG_cluster_053	50	420	3.00E-106	EpoF_Q9L8C5_1mod	Epothilone
KS_682	1277	RHG_cluster_140	52	424	4.00E-117	EpoD_Q9L8C7_4mod	Epothilone
KS_685	1268	RHG_cluster_140	56	424	3.00E-120	EpoD_Q9L8C7_4mod	Epothilone
KS_689	1259	RHG_cluster_161	52	423	7.00E-115	EpoD_Q9L8C7_4mod	Epothilone
KS_691	1259	RHG_cluster_164	51	422	2.00E-111	EpoD_Q9L8C7_4mod	Epothilone
KS_692	1259	RHG_cluster_124	51	429	1.00E-113	EpoD_Q9L8C7_4mod	Epothilone
KS_693	1259	RHG_cluster_037	53	427	1.00E-112	EpoD_Q9L8C7_4mod	Epothilone
KS_697	1265	RHG_cluster_165	53	428	4.00E-115	EpoD_Q9L8C7_2mod	Epothilone
KS_700	1271	RHG_cluster_019	54	427	8.00E-120	TetE_BAE93730_1mod	Tetronomycin
KS_704	1262	RHG_cluster_154	49	424	4.00E-113	EpoD_Q9L8C7_4mod	Epothilone
KS_707	1262	RHG_cluster_069	54	425	7.00E-113	EpoD_Q9L8C7_4mod	Epothilone
KS_712	1259	RHG_cluster_100	52	427	5.00E-117	EpoD_Q9L8C7_2mod	Epothilone
KS_714	1256	RHG_cluster_040	50	423	2.00E-110	EpoF_Q9L8C5_1mod	Epothilone
KS_715	1259	RHG_cluster_090	49	427	2.00E-110	EpoD_Q9L8C7_2mod	Epothilone
KS_719	1259	RHG_cluster_056	49	427	7.00E-109	EpoD_Q9L8C7_2mod	Epothilone
KS_723	1256	RHG_cluster_054	51	430	9.00E-114	EpoD_Q9L8C7_2mod	Epothilone
KS_724	1259	RHG_cluster_057	50	423	2.00E-112	EpoD_Q9L8C7_4mod	Epothilone
KS_727	1259	RHG_cluster_179	50	422	2.00E-107	EpoD_Q9L8C7_4mod	Epothilone
KS_729	1265	RHG_cluster_027	52	424	2.00E-101	EpoD_Q9L8C7_4mod	Epothilone
KS_733	1262	RHG_cluster_147	47	426	7.00E-102	EpoD_Q9L8C7_2mod	Epothilone
KS_735	1124	RHG_cluster_194	49	379	1.00E-92	EpoD_Q9L8C7_4mod	Epothilone

KS_738	1277	RHG_cluster_159	53	435	2.00E-114	EpoD_Q9L8C7_4mod	Epothilone
KS_741	1259	RHG_cluster_008	51	422	1.00E-113	EpoD_Q9L8C7_4mod	Epothilone
KS_744	1262	RHG_cluster_087	49	424	4.00E-107	TetE_BAE93730_1mod	Tetronomycin
KS_746	1274	RHG_cluster_177	49	428	9.00E-99	EpoD_Q9L8C7_4mod	Epothilone
KS_754	1262	RHG_cluster_193	54	425	5.00E-122	EpoD_Q9L8C7_2mod	Epothilone
KS_758	1250	RHG_cluster_119	53	428	4.00E-116	EpoD_Q9L8C7_2mod	Epothilone
KS_759	1268	RHG_cluster_019	57	430	1.00E-117	EpoD_Q9L8C7_4mod	Epothilone

Table S7. BLAST search of the KS sequences from the metagenome of *Speciospongia* sp. against the KS sequences of known products from the NaPDos database

KS ID	Length (nt)	BGC ID	Identity (%)	Aligned length (aa)	E-value	NaPDos ID	Pathway product
KS_002	1086	SPV_cluster_403	62	359	4.00E-122	FabF_Ecoli_FAS	Fatty acid
KS_003	459	SPV_cluster_447	62	150	2.00E-49	EpoD_Q9L8C7_4mod	Epothilone
KS_005	1266	SPV_cluster_194	38	431	3.00E-68	EpoD_Q9L8C7_2mod	Epothilone
KS_008	1266	SPV_cluster_115	53	432	6.00E-115	EpoD_Q9L8C7_4mod	Epothilone
KS_009	663	SPV_cluster_191	56	209	6.00E-64	EpoD_Q9L8C7_4mod	Epothilone
KS_015	435	SPV_cluster_422	59	143	3.00E-47	EpoD_Q9L8C7_4mod	Epothilone
KS_017	696	SPV_cluster_454	43	228	1.00E-44	FUMON_AAD43562_i	Fumonisin
KS_018	1266	SPV_cluster_241	54	432	6.00E-117	EpoD_Q9L8C7_4mod	Epothilone
KS_022	783	SPV_cluster_440	53	258	2.00E-75	EpoD_Q9L8C7_2mod	Epothilone
KS_023	297	SPV_cluster_287	52	96	4.00E-24	EpoD_Q9L8C7_4mod	Epothilone
KS_024	945	SPV_cluster_405	55	286	5.00E-87	EpoD_Q9L8C7_1mod	Epothilone
KS_025	1221	SPV_cluster_202	51	406	4.00E-110	EpoD_Q9L8C7_4mod	Epothilone
KS_027	351	SPV_cluster_456	55	114	6.00E-33	EpoD_Q9L8C7_4mod	Epothilone
KS_031	744	SPV_cluster_302	54	246	8.00E-67	EpoD_Q9L8C7_4mod	Epothilone
KS_034	1077	SPV_cluster_360	54	355	2.00E-93	EpoD_Q9L8C7_4mod	Epothilone
KS_035	1014	SPV_cluster_461	63	339	9.00E-107	FabF_Ecoli_FAS	Fatty acid
KS_038	1113	SPV_cluster_096	52	368	1.00E-101	EpoD_Q9L8C7_2mod	Epothilone
KS_042	669	SPV_cluster_382	58	212	9.00E-61	EpoD_Q9L8C7_4mod	Epothilone
KS_047	1113	SPV_cluster_418	53	365	3.00E-103	EpoD_Q9L8C7_4mod	Epothilone
KS_049	438	SPV_cluster_450	53	143	3.00E-43	EpoD_Q9L8C7_2mod	Epothilone
KS_050	279	SPV_cluster_446	54	90	6.00E+00	NysC_Q9L4W3_2mod	Nystatin
KS_051	531	SPV_cluster_451	56	171	7.00E-53	AveA2_Q9S0R7_2mod	Avermectin
KS_055	312	SPV_cluster_391	45	109	2.00E+00	VirA_BAF50727_4T	Virginiamycin
KS_057	849	SPV_cluster_378	57	276	4.00E-78	EpoD_Q9L8C7_1mod	Epothilone
KS_058	954	SPV_cluster_384	52	322	1.00E-86	EpoA_Q9L8C9_mod	Epothilone
KS_059	570	SPV_cluster_455	60	186	3.00E-51	EpoA_Q9L8C9_mod	Epothilone
KS_068	888	SPV_cluster_333	53	279	2.00E-74	EpoD_Q9L8C7_1mod	Epothilone
KS_070	1023	SPV_cluster_408	26	336	2.00E-22	FabF_Bacillus_FAS	Fatty acid

KS_072	912	SPV_cluster_428	57	285	3.00E-88	EpoD_Q9L8C7_4mod	Epothilone
KS_076	713	SPV_cluster_204	42	224	1.00E-44	TetA_BAE93722_KS1	Tetronomycin
KS_081	1118	SPV_cluster_373	52	377	8.00E-94	TetF_BAE93731_2mod	Tetronomycin
KS_082	1166	SPV_cluster_273	54	367	5.00E-105	EpoA_Q9L8C9_mod	Epothilone
KS_085	1112	SPV_cluster_396	52	369	9.00E-106	EpoF_Q9L8C5_1mod	Epothilone
KS_086	671	SPV_cluster_323	56	229	1.00E-66	EpoA_Q9L8C9_mod	Epothilone
KS_087	1097	SPV_cluster_425	52	365	1.00E-99	EpoD_Q9L8C7_2mod	Epothilone
KS_089	929	SPV_cluster_347	58	285	4.00E-88	EpoD_Q9L8C7_1mod	Epothilone
KS_090	398	SPV_cluster_386	50	140	2.00E-29	VirA_BAF50727_4T	Virginiamycin
KS_092	1133	SPV_cluster_423	54	378	9.00E-116	FabF_Bacillus_FAS	Fatty acid
KS_093	1085	SPV_cluster_279	60	361	5.00E-114	FabF_Bacillus_FAS	Fatty acid
KS_097	914	SPV_cluster_292	50	273	6.00E-75	NysJ_Q9L4X2_2mod	Nystatin
KS_099	1061	SPV_cluster_262	48	355	2.00E-72	FabF_Bacillus_FAS	Fatty acid
KS_100	1292	SPV_cluster_356	38	439	7.00E-73	EpoD_Q9L8C7_2mod	Epothilone
KS_114	1043	SPV_cluster_318	61	349	1.00E-124	FabB_Ecoli_FAS	Fatty acid
KS_118	1223	SPV_cluster_209	53	407	7.00E-113	FabF_Bacillus_FAS	Fatty acid
KS_119	1199	SPV_cluster_043	40	404	2.00E-86	FabF_Bacillus_FAS	Fatty acid
KS_121	1223	SPV_cluster_417	53	408	4.00E-121	FabF_Bacillus_FAS	Fatty acid
KS_122	1184	SPV_cluster_401	57	398	1.00E-128	FabB_Ecoli_FAS	Fatty acid
KS_123	1223	SPV_cluster_099	61	407	1.00E-132	FabF_Ecoli_FAS	Fatty acid
KS_124	1202	SPV_cluster_375	62	401	3.00E-132	FabB_Ecoli_FAS	Fatty acid
KS_125	1250	SPV_cluster_160	53	413	9.00E-108	FabF_Bacillus_FAS	Fatty acid
KS_127	1205	SPV_cluster_228	63	403	7.00E-140	FabB_Ecoli_FAS	Fatty acid
KS_128	1223	SPV_cluster_054	52	408	3.00E-120	FabF_Bacillus_FAS	Fatty acid
KS_129	1223	SPV_cluster_033	53	409	6.00E-122	FabF_Bacillus_FAS	Fatty acid
KS_132	1196	SPV_cluster_416	63	402	2.00E-117	FabB_Ecoli_FAS	Fatty acid
KS_133	998	SPV_cluster_358	44	335	5.00E-80	FabF_Bacillus_FAS	Fatty acid
KS_136	1223	SPV_cluster_324	52	408	3.00E-122	FabF_Bacillus_FAS	Fatty acid
KS_139	1202	SPV_cluster_293	62	400	1.00E-141	FabB_Ecoli_FAS	Fatty acid
KS_141	1211	SPV_cluster_230	60	401	3.00E-123	FabB_Ecoli_FAS	Fatty acid
KS_145	1256	SPV_cluster_074	49	414	3.00E-106	FabF_Bacillus_FAS	Fatty acid
KS_147	1208	SPV_cluster_155	64	405	2.00E-134	FabB_Ecoli_FAS	Fatty acid

KS_149	1223	SPV_cluster_076	62	407	4.00E-137	FabF_Ecoli_FAS	Fatty acid
KS_152	1226	SPV_cluster_251	51	408	6.00E-117	FabF_Bacillus_FAS	Fatty acid
KS_155	1058	SPV_cluster_438	60	354	3.00E-110	FabB_Ecoli_FAS	Fatty acid
KS_156	1100	SPV_cluster_349	54	364	2.00E-80	FabF_Bacillus_FAS	Fatty acid
KS_157	1223	SPV_cluster_032	53	408	3.00E-113	FabF_Bacillus_FAS	Fatty acid
KS_158	1220	SPV_cluster_233	39	406	8.00E-55	FabF_Bacillus_FAS	Fatty acid
KS_160	1286	SPV_cluster_245	53	428	2.00E-117	EpoD_Q9L8C7_4mod	Epothilone
KS_164	1223	SPV_cluster_329	56	408	7.00E-125	FabF_Bacillus_FAS	Fatty acid
KS_165	1223	SPV_cluster_039	54	407	5.00E-122	FabF_Bacillus_FAS	Fatty acid
KS_168	1223	SPV_cluster_002	58	407	6.00E-140	FabF_Bacillus_FAS	Fatty acid
KS_169	1253	SPV_cluster_413	52	423	3.00E-118	EpoD_Q9L8C7_4mod	Epothilone
KS_171	1223	SPV_cluster_235	57	407	6.00E-129	FabF_Bacillus_FAS	Fatty acid
KS_172	1229	SPV_cluster_014	54	410	9.00E-111	FabF_Bacillus_FAS	Fatty acid
KS_177	1292	SPV_cluster_421	39	441	1.00E-73	EpoD_Q9L8C7_2mod	Epothilone
KS_180	1028	SPV_cluster_432	52	343	3.00E-92	FabF_Bacillus_FAS	Fatty acid
KS_184	1217	SPV_cluster_430	50	407	2.00E-121	FabF_Bacillus_FAS	Fatty acid
KS_185	1052	SPV_cluster_411	32	361	8.00E-38	FabF_Bacillus_FAS	Fatty acid
KS_187	1223	SPV_cluster_254	60	407	3.00E-135	FabF_Ecoli_FAS	Fatty acid
KS_190	1223	SPV_cluster_284	63	382	5.00E-133	FabF_Ecoli_FAS	Fatty acid
KS_192	1202	SPV_cluster_138	36	408	1.00E-59	FabF_Bacillus_FAS	Fatty acid
KS_194	1229	SPV_cluster_317	46	411	3.00E-80	FabF_Bacillus_FAS	Fatty acid
KS_200	1223	SPV_cluster_258	56	405	6.00E-111	FabF_Bacillus_FAS	Fatty acid
KS_207	1223	SPV_cluster_389	59	383	2.00E-128	FabF_Ecoli_FAS	Fatty acid
KS_209	1220	SPV_cluster_128	59	408	2.00E-140	FabF_Ecoli_FAS	Fatty acid
KS_210	1214	SPV_cluster_009	57	407	1.00E-128	FabF_Ecoli_FAS	Fatty acid
KS_211	1340	SPV_cluster_001	49	451	1.00E-89	EpoE_Q9L8C6_1mod	Epothilone
KS_213	1229	SPV_cluster_441	49	408	1.00E-114	FabF_Bacillus_FAS	Fatty acid
KS_214	1223	SPV_cluster_289	51	408	2.00E-121	FabF_Bacillus_FAS	Fatty acid
KS_215	1214	SPV_cluster_239	50	406	7.00E-94	FabF_Bacillus_FAS	Fatty acid
KS_216	1226	SPV_cluster_003	57	408	3.00E-114	FabF_Ecoli_FAS	Fatty acid
KS_219	1199	SPV_cluster_148	44	403	2.00E-95	FabF_Bacillus_FAS	Fatty acid
KS_226	1157	SPV_cluster_086	43	406	7.00E-72	FabF_Bacillus_FAS	Fatty acid

KS_228	1223	SPV_cluster_195	48	407	2.00E-99	FabF_Bacillus_FAS	Fatty acid
KS_229	1217	SPV_cluster_280	60	407	2.00E-134	FabF_Ecoli_FAS	Fatty acid
KS_230	1223	SPV_cluster_037	61	408	2.00E-117	FabF_Ecoli_FAS	Fatty acid
KS_233	1217	SPV_cluster_060	59	406	3.00E-134	FabF_Ecoli_FAS	Fatty acid
KS_234	1217	SPV_cluster_121	61	407	2.00E-134	FabF_Ecoli_FAS	Fatty acid
KS_238	1217	SPV_cluster_180	51	407	1.00E-123	FabF_Bacillus_FAS	Fatty acid
KS_241	1163	SPV_cluster_207	60	382	1.00E-119	FabF_Ecoli_FAS	Fatty acid
KS_242	1226	SPV_cluster_445	56	408	1.00E-113	FabF_Ecoli_FAS	Fatty acid
KS_243	1217	SPV_cluster_015	62	405	3.00E-139	FabF_Ecoli_FAS	Fatty acid
KS_245	1235	SPV_cluster_178	47	414	5.00E-90	FabF_Ecoli_FAS	Fatty acid
KS_246	1187	SPV_cluster_126	43	251	6.00E-47	FabF_Bacillus_FAS	Fatty acid
KS_248	1220	SPV_cluster_197	61	408	1.00E-132	FabF_Ecoli_FAS	Fatty acid
KS_251	1247	SPV_cluster_101	55	414	2.00E-97	FabF_Ecoli_FAS	Fatty acid
KS_255	1229	SPV_cluster_222	55	409	1.00E-119	FabF_Bacillus_FAS	Fatty acid
KS_257	1292	SPV_cluster_426	38	439	2.00E-67	EpoD_Q9L8C7_2mod	Epothilone
KS_258	1220	SPV_cluster_036	38	407	9.00E-70	FabF_Bacillus_FAS	Fatty acid
KS_260	1226	SPV_cluster_419	58	409	5.00E-123	FabF_Bacillus_FAS	Fatty acid
KS_265	1292	SPV_cluster_091	39	443	1.00E-69	EpoD_Q9L8C7_2mod	Epothilone
KS_268	1292	SPV_cluster_172	38	435	2.00E-63	TetA_BAE93722_KS1	Tetronomycin
KS_271	1220	SPV_cluster_255	54	406	2.00E-107	FabF_Bacillus_FAS	Fatty acid
KS_272	1286	SPV_cluster_005	47	434	5.00E-109	EpoD_Q9L8C7_4mod	Epothilone
KS_273	1220	SPV_cluster_025	55	406	5.00E-122	FabF_Bacillus_FAS	Fatty acid
KS_274	1226	SPV_cluster_024	58	408	2.00E-136	FabF_Bacillus_FAS	Fatty acid
KS_275	1229	SPV_cluster_031	51	409	6.00E-114	FabF_Bacillus_FAS	Fatty acid
KS_276	1223	SPV_cluster_217	62	408	1.00E-140	FabF_Ecoli_FAS	Fatty acid
KS_277	1151	SPV_cluster_129	44	405	9.00E-76	FabF_Bacillus_FAS	Fatty acid
KS_280	1151	SPV_cluster_229	28	407	3.00E-31	FabF_Bacillus_FAS	Fatty acid
KS_281	1292	SPV_cluster_035	51	429	2.00E-115	EpoD_Q9L8C7_2mod	Epothilone
KS_282	1226	SPV_cluster_159	50	409	7.00E-114	FabF_Bacillus_FAS	Fatty acid
KS_286	1229	SPV_cluster_402	52	407	2.00E-102	FabF_Bacillus_FAS	Fatty acid
KS_287	1289	SPV_cluster_214	47	438	3.00E-110	EpoD_Q9L8C7_2mod	Epothilone
KS_288	1289	SPV_cluster_040	47	438	1.00E-110	EpoD_Q9L8C7_2mod	Epothilone

KS_289	1286	SPV_cluster_225	52	423	3.00E-118	EpoD_Q9L8C7_4mod	Epothilone
KS_291	1223	SPV_cluster_107	55	407	5.00E-132	FabF_Bacillus_FAS	Fatty acid
KS_293	1292	SPV_cluster_161	37	438	2.00E-67	EpoD_Q9L8C7_2mod	Epothilone
KS_297	1214	SPV_cluster_085	46	404	3.00E-84	FabF_Bacillus_FAS	Fatty acid
KS_301	1154	SPV_cluster_093	25	409	5.00E-28	FabF_Bacillus_FAS	Fatty acid
KS_303	1286	SPV_cluster_084	52	429	6.00E-115	EpoD_Q9L8C7_4mod	Epothilone
KS_305	1067	SPV_cluster_406	50	331	7.00E-87	FabF_Bacillus_FAS	Fatty acid
KS_306	1286	SPV_cluster_268	50	433	1.00E-101	EpoD_Q9L8C7_2mod	Epothilone
KS_307	1259	SPV_cluster_312	33	424	6.00E-44	FabB_Ecoli_FAS	Fatty acid
KS_308	1154	SPV_cluster_124	24	366	9.00E+00	FabF_Bacillus_FAS	Fatty acid
KS_310	1223	SPV_cluster_400	57	408	1.00E-121	FabF_Bacillus_FAS	Fatty acid
KS_312	1160	SPV_cluster_113	45	354	8.00E-77	FabF_Bacillus_FAS	Fatty acid
KS_315	1160	SPV_cluster_320	43	406	3.00E-71	FabF_Bacillus_FAS	Fatty acid
KS_317	1160	SPV_cluster_224	45	354	2.00E-73	FabF_Bacillus_FAS	Fatty acid
KS_321	1217	SPV_cluster_081	44	404	9.00E-78	FabF_Bacillus_FAS	Fatty acid
KS_323	1286	SPV_cluster_253	48	428	2.00E-99	EpoF_Q9L8C5_1mod	Epothilone
KS_326	1223	SPV_cluster_353	53	408	5.00E-100	FabF_Bacillus_FAS	Fatty acid
KS_332	1286	SPV_cluster_171	48	433	3.00E-105	EpoD_Q9L8C7_2mod	Epothilone
KS_336	1292	SPV_cluster_163	40	438	1.00E-74	TetA_BAE93722_KS1	Tetronomycin
KS_337	1292	SPV_cluster_153	36	436	2.00E-56	FUMON_AAD43562_i	Fumonisin
KS_339	1292	SPV_cluster_310	36	440	1.00E-62	FUMON_AAD43562_i	Fumonisin
KS_341	1226	SPV_cluster_276	49	408	8.00E-106	FabF_Bacillus_FAS	Fatty acid
KS_345	1154	SPV_cluster_290	43	406	6.00E-65	FabF_Bacillus_FAS	Fatty acid
KS_349	1247	SPV_cluster_205	54	414	2.00E-122	FabF_Bacillus_FAS	Fatty acid
KS_350	1292	SPV_cluster_300	38	439	3.00E-74	EpoD_Q9L8C7_2mod	Epothilone
KS_351	1148	SPV_cluster_436	53	382	1.00E-111	FabF_Bacillus_FAS	Fatty acid
KS_353	1292	SPV_cluster_108	38	452	2.00E-70	KirAII_CAN89632_5T	Kirromycin
KS_356	1292	SPV_cluster_166	36	414	1.00E-61	EpoD_Q9L8C7_2mod	Epothilone
KS_357	1292	SPV_cluster_281	37	439	2.00E-70	FUMON_AAD43562_i	Fumonisin
KS_360	1232	SPV_cluster_192	51	410	2.00E-120	FabF_Bacillus_FAS	Fatty acid
KS_361	1292	SPV_cluster_065	34	440	2.00E-66	FUMON_AAD43562_i	Fumonisin
KS_362	1220	SPV_cluster_271	57	407	1.00E-125	FabF_Bacillus_FAS	Fatty acid

KS_363	1220	SPV_cluster_045	50	408	4.00E-116	FabF_Bacillus_FAS	Fatty acid
KS_369	1229	SPV_cluster_007	51	414	7.00E-123	FabF_Bacillus_FAS	Fatty acid
KS_370	1100	SPV_cluster_112	40	261	3.00E-39	FabF_Ecoli_FAS	Fatty acid
KS_371	1298	SPV_cluster_141	39	441	3.00E-71	EpoD_Q9L8C7_2mod	Epothilone
KS_378	1223	SPV_cluster_363	51	409	7.00E-127	FabF_Bacillus_FAS	Fatty acid
KS_379	1253	SPV_cluster_362	52	416	3.00E-120	FabF_Bacillus_FAS	Fatty acid
KS_381	1229	SPV_cluster_367	47	408	1.00E-99	FabF_Bacillus_FAS	Fatty acid
KS_383	1220	SPV_cluster_027	59	408	3.00E-130	FabF_Bacillus_FAS	Fatty acid
KS_385	1229	SPV_cluster_341	53	410	1.00E-116	FabF_Bacillus_FAS	Fatty acid
KS_386	1232	SPV_cluster_173	52	410	3.00E-120	FabF_Bacillus_FAS	Fatty acid
KS_389	1247	SPV_cluster_042	51	416	1.00E-116	FabF_Bacillus_FAS	Fatty acid
KS_391	1154	SPV_cluster_442	48	382	2.00E-93	FabF_Bacillus_FAS	Fatty acid
KS_394	1226	SPV_cluster_053	43	410	3.00E-94	FabF_Bacillus_FAS	Fatty acid
KS_399	1286	SPV_cluster_057	51	432	2.00E-113	EpoD_Q9L8C7_4mod	Epothilone
KS_401	1184	SPV_cluster_125	32	406	4.00E-39	FabF_Bacillus_FAS	Fatty acid
KS_402	1184	SPV_cluster_092	33	406	8.00E-46	FabF_Bacillus_FAS	Fatty acid
KS_404	1223	SPV_cluster_275	44	407	9.00E-92	FabF_Bacillus_FAS	Fatty acid
KS_405	1292	SPV_cluster_049	38	437	2.00E-63	FUMON_AAD43562_i	Fumonisin
KS_409	1292	SPV_cluster_067	39	439	1.00E-68	TetA_BAE93722_KS1	Tetronomycin
KS_415	1232	SPV_cluster_308	44	410	2.00E-95	FabF_Bacillus_FAS	Fatty acid
KS_416	1286	SPV_cluster_168	50	438	5.00E-114	EpoD_Q9L8C7_2mod	Epothilone
KS_418	1088	SPV_cluster_444	53	363	8.00E-106	FabF_Bacillus_FAS	Fatty acid
KS_420	1286	SPV_cluster_371	49	430	6.00E-107	EpoF_Q9L8C5_1mod	Epothilone
KS_422	1286	SPV_cluster_149	47	442	7.00E-107	EpoD_Q9L8C7_2mod	Epothilone
KS_425	1232	SPV_cluster_201	49	411	5.00E-119	FabF_Bacillus_FAS	Fatty acid
KS_426	1211	SPV_cluster_339	55	399	2.00E-117	FabF_Bacillus_FAS	Fatty acid
KS_427	1175	SPV_cluster_215	48	373	1.00E-83	FabF_Bacillus_FAS	Fatty acid
KS_432	1286	SPV_cluster_146	47	437	2.00E-100	EpoF_Q9L8C5_1mod	Epothilone
KS_433	1175	SPV_cluster_307	50	373	9.00E-87	FabF_Bacillus_FAS	Fatty acid
KS_434	1223	SPV_cluster_340	49	406	1.00E-109	FabF_Bacillus_FAS	Fatty acid
KS_435	1094	SPV_cluster_345	26	350	8.00E+00	FabF_Bacillus_FAS	Fatty acid
KS_436	1232	SPV_cluster_216	44	410	2.00E-97	FabF_Bacillus_FAS	Fatty acid

KS_438	1223	SPV_cluster_301	50	406	1.00E-105	FabF_Bacillus_FAS	Fatty acid
KS_441	1046	SPV_cluster_443	49	347	3.00E-69	FabF_Bacillus_FAS	Fatty acid
KS_442	1175	SPV_cluster_152	50	373	5.00E-88	FabF_Bacillus_FAS	Fatty acid
KS_443	1175	SPV_cluster_414	48	365	6.00E-74	FabF_Bacillus_FAS	Fatty acid
KS_444	1235	SPV_cluster_083	55	412	6.00E-115	FabF_Bacillus_FAS	Fatty acid
KS_445	1286	SPV_cluster_071	48	439	2.00E-105	EpoD_Q9L8C7_2mod	Epothilone
KS_450	1292	SPV_cluster_412	37	439	6.00E-67	FUMON_AAD43562_i	Fumonisin
KS_451	1289	SPV_cluster_114	48	438	7.00E-102	EpoD_Q9L8C7_2mod	Epothilone
KS_456	1184	SPV_cluster_263	60	393	3.00E-123	FabF_Ecoli_FAS	Fatty acid
KS_457	1286	SPV_cluster_047	50	431	7.00E-104	EpoD_Q9L8C7_2mod	Epothilone
KS_458	1145	SPV_cluster_236	40	386	9.00E-51	FabF_Bacillus_FAS	Fatty acid
KS_463	1190	SPV_cluster_066	51	398	7.00E-107	FabF_Bacillus_FAS	Fatty acid
KS_464	1178	SPV_cluster_069	54	391	6.00E-100	FabF_Bacillus_FAS	Fatty acid
KS_467	1196	SPV_cluster_364	50	408	4.00E-110	FabF_Bacillus_FAS	Fatty acid
KS_470	977	SPV_cluster_330	49	323	1.00E-81	FabF_Bacillus_FAS	Fatty acid
KS_472	1211	SPV_cluster_259	51	401	3.00E-116	FabF_Bacillus_FAS	Fatty acid
KS_475	1154	SPV_cluster_366	59	385	3.00E-126	FabB_Ecoli_FAS	Fatty acid
KS_477	1286	SPV_cluster_291	52	433	4.00E-106	EpoD_Q9L8C7_2mod	Epothilone
KS_478	1142	SPV_cluster_380	32	221	4.00E-24	FabF_Bacillus_FAS	Fatty acid
KS_480	1199	SPV_cluster_334	53	394	4.00E-116	FabF_Bacillus_FAS	Fatty acid
KS_482	1172	SPV_cluster_182	55	390	2.00E-119	FabF_Bacillus_FAS	Fatty acid
KS_485	1298	SPV_cluster_226	51	434	2.00E-106	EpoD_Q9L8C7_4mod	Epothilone
KS_487	1112	SPV_cluster_179	45	358	3.00E-64	FabF_Bacillus_FAS	Fatty acid
KS_489	1292	SPV_cluster_220	37	440	3.00E-68	FUMON_AAD43562_i	Fumonisin
KS_492	1172	SPV_cluster_240	46	387	1.00E-89	FabF_Bacillus_FAS	Fatty acid
KS_493	1286	SPV_cluster_134	47	431	4.00E-107	EpoD_Q9L8C7_2mod	Epothilone
KS_496	1166	SPV_cluster_274	54	389	3.00E-116	FabF_Bacillus_FAS	Fatty acid
KS_500	1286	SPV_cluster_278	52	438	1.00E-116	EpoD_Q9L8C7_2mod	Epothilone
KS_502	1286	SPV_cluster_213	50	434	5.00E-98	EpoD_Q9L8C7_4mod	Epothilone
KS_509	1292	SPV_cluster_154	51	437	2.00E-114	EpoD_Q9L8C7_4mod	Epothilone
KS_510	1292	SPV_cluster_020	52	432	3.00E-115	EpoD_Q9L8C7_4mod	Epothilone
KS_512	1292	SPV_cluster_264	38	444	2.00E-66	HSAF_ABL86391_i	HSAF (heat stable antifungal factor)

KS_513	1160	SPV_cluster_316	54	385	1.00E-110	FabF_Bacillus_FAS	Fatty acid
KS_515	1244	SPV_cluster_029	51	426	2.00E-105	EpoD_Q9L8C7_2mod	Epothilone
KS_522	1229	SPV_cluster_433	53	409	1.00E-109	FabF_Bacillus_FAS	Fatty acid
KS_523	1283	SPV_cluster_208	52	429	1.00E-116	EpoD_Q9L8C7_4mod	Epothilone
KS_524	1286	SPV_cluster_051	50	430	3.00E-115	EpoD_Q9L8C7_2mod	Epothilone
KS_530	1292	SPV_cluster_174	49	429	4.00E-110	EpoD_Q9L8C7_2mod	Epothilone
KS_531	1058	SPV_cluster_337	48	258	5.00E-53	FabF_Bacillus_FAS	Fatty acid
KS_533	1292	SPV_cluster_368	37	439	2.00E-67	FUMON_AAD43562_i	Fumonisin
KS_534	1286	SPV_cluster_326	52	428	9.00E-116	EpoD_Q9L8C7_2mod	Epothilone
KS_535	1286	SPV_cluster_269	49	434	1.00E-103	EpoD_Q9L8C7_4mod	Epothilone
KS_539	1286	SPV_cluster_102	50	434	7.00E-114	EpoD_Q9L8C7_2mod	Epothilone
KS_542	1286	SPV_cluster_299	49	433	2.00E-104	EpoD_Q9L8C7_2mod	Epothilone
KS_544	1286	SPV_cluster_078	55	428	2.00E-115	EpoD_Q9L8C7_4mod	Epothilone
KS_547	1292	SPV_cluster_008	49	439	3.00E-113	EpoD_Q9L8C7_2mod	Epothilone
KS_552	1277	SPV_cluster_169	53	425	9.00E-120	EpoD_Q9L8C7_4mod	Epothilone
KS_556	1286	SPV_cluster_212	48	439	7.00E-105	EpoD_Q9L8C7_2mod	Epothilone
KS_560	1283	SPV_cluster_082	51	427	3.00E-110	EpoF_Q9L8C5_1mod	Epothilone
KS_561	1250	SPV_cluster_221	53	428	2.00E-104	AveA2_Q9S0R7_4mod	Avermectin
KS_562	1292	SPV_cluster_151	49	428	1.00E-114	EpoF_Q9L8C5_1mod	Epothilone
KS_565	1259	SPV_cluster_030	56	427	3.00E-123	EpoD_Q9L8C7_4mod	Epothilone
KS_566	1286	SPV_cluster_311	47	437	1.00E-101	EpoF_Q9L8C5_1mod	Epothilone
KS_567	1301	SPV_cluster_246	51	432	3.00E-111	EpoD_Q9L8C7_4mod	Epothilone
KS_568	1166	SPV_cluster_250	38	350	7.00E-51	FabB_Ecoli_FAS	Fatty acid
KS_571	1082	SPV_cluster_376	38	348	3.00E-52	FabB_Ecoli_FAS	Fatty acid
KS_574	1292	SPV_cluster_052	50	433	6.00E-112	EpoD_Q9L8C7_2mod	Epothilone
KS_578	1178	SPV_cluster_094	36	405	4.00E-53	FabF_Bacillus_FAS	Fatty acid
KS_582	1010	SPV_cluster_137	35	209	6.00E-31	FabF_Bacillus_FAS	Fatty acid
KS_584	1025	SPV_cluster_136	37	260	1.00E-38	FabF_Bacillus_FAS	Fatty acid
KS_587	1184	SPV_cluster_123	35	376	8.00E-52	FabF_Bacillus_FAS	Fatty acid
KS_589	1259	SPV_cluster_338	50	425	1.00E-110	EpoD_Q9L8C7_2mod	Epothilone
KS_592	1277	SPV_cluster_106	53	428	5.00E-121	EpoD_Q9L8C7_4mod	Epothilone
KS_594	293	SPV_cluster_111	51	93	1.00E-24	NysJ_Q9L4X2_2mod	Nystatin

KS_595	1259	SPV_cluster_357	51	425	5.00E-109	EpoD_Q9L8C7_2mod	Epothilone
KS_597	1256	SPV_cluster_309	51	422	1.00E-98	EpoD_Q9L8C7_4mod	Epothilone
KS_598	1259	SPV_cluster_394	51	422	6.00E-111	EpoD_Q9L8C7_4mod	Epothilone
KS_600	1079	SPV_cluster_145	28	336	2.00E+00	FabF_Bacillus_FAS	Fatty acid
KS_601	992	SPV_cluster_261	30	259	3.00E+00	FabB_Streptomyces_FAS	Fatty acid
KS_604	851	SPV_cluster_016	25	269	1.00E+00	FabF_Bacillus_FAS	Fatty acid
KS_606	653	SPV_cluster_348	59	215	1.00E-69	EpoD_Q9L8C7_1mod	Epothilone
KS_610	287	SPV_cluster_459	63	92	2.00E-29	EpoD_Q9L8C7_4mod	Epothilone
KS_612	1256	SPV_cluster_106	52	435	5.00E-113	TetF_BAE93731_2mod	Tetronomycin
KS_622	1259	SPV_cluster_315	44	427	2.00E-92	EpoD_Q9L8C7_2mod	Epothilone
KS_623	1259	SPV_cluster_305	51	425	3.00E-110	EpoD_Q9L8C7_4mod	Epothilone
KS_624	1259	SPV_cluster_266	49	422	3.00E-105	EpoD_Q9L8C7_4mod	Epothilone
KS_629	1259	SPV_cluster_314	54	423	8.00E-119	EpoD_Q9L8C7_4mod	Epothilone
KS_631	1235	SPV_cluster_377	55	423	2.00E-106	EpoD_Q9L8C7_4mod	Epothilone
KS_632	1259	SPV_cluster_167	51	424	3.00E-107	EpoD_Q9L8C7_4mod	Epothilone
KS_634	1265	SPV_cluster_030	54	424	3.00E-119	EpoD_Q9L8C7_4mod	Epothilone
KS_635	1262	SPV_cluster_030	54	424	8.00E-121	EpoD_Q9L8C7_4mod	Epothilone
KS_639	1259	SPV_cluster_260	50	422	5.00E-113	EpoD_Q9L8C7_4mod	Epothilone
KS_641	1259	SPV_cluster_052	50	425	1.00E-113	EpoD_Q9L8C7_2mod	Epothilone
KS_648	1265	SPV_cluster_211	50	427	1.00E-112	EpoD_Q9L8C7_2mod	Epothilone
KS_651	1256	SPV_cluster_227	50	422	8.00E-107	EpoD_Q9L8C7_4mod	Epothilone
KS_653	1259	SPV_cluster_306	50	422	2.00E-112	EpoD_Q9L8C7_4mod	Epothilone
KS_656	1244	SPV_cluster_237	51	436	3.00E-107	EpoD_Q9L8C7_2mod	Epothilone
KS_658	1259	SPV_cluster_030	53	421	8.00E-119	EpoF_Q9L8C5_1mod	Epothilone
KS_663	1259	SPV_cluster_206	49	427	4.00E-103	TetF_BAE93731_2mod	Tetronomycin
KS_665	1259	SPV_cluster_210	53	426	5.00E-115	EpoA_Q9L8C9_mod	Epothilone
KS_667	1259	SPV_cluster_181	51	425	6.00E-111	EpoD_Q9L8C7_4mod	Epothilone
KS_675	1268	SPV_cluster_030	55	421	2.00E-115	EpoF_Q9L8C5_1mod	Epothilone
KS_676	1259	SPV_cluster_082	51	427	1.00E-113	EpoD_Q9L8C7_2mod	Epothilone
KS_678	1253	SPV_cluster_221	50	420	3.00E-106	EpoF_Q9L8C5_1mod	Epothilone
KS_680	1277	SPV_cluster_030	52	424	4.00E-117	EpoD_Q9L8C7_4mod	Epothilone
KS_684	1268	SPV_cluster_030	56	424	3.00E-120	EpoD_Q9L8C7_4mod	Epothilone

KS_686	1256	SPV_cluster_191	54	428	3.00E-116	EpoD_Q9L8C7_4mod	Epothilone
KS_687	1259	SPV_cluster_051	52	423	7.00E-115	EpoD_Q9L8C7_4mod	Epothilone
KS_690	1259	SPV_cluster_096	52	422	2.00E-114	EpoD_Q9L8C7_4mod	Epothilone
KS_694	1259	SPV_cluster_057	53	424	5.00E-113	EpoD_Q9L8C7_4mod	Epothilone
KS_696	1259	SPV_cluster_171	53	428	3.00E-115	EpoD_Q9L8C7_2mod	Epothilone
KS_698	1259	SPV_cluster_040	48	427	2.00E-111	EpoD_Q9L8C7_2mod	Epothilone
KS_699	1259	SPV_cluster_214	48	427	3.00E-107	EpoD_Q9L8C7_2mod	Epothilone
KS_702	1271	SPV_cluster_029	54	427	8.00E-120	TetE_BAE93730_1mod	Tetronomycin
KS_703	1259	SPV_cluster_134	48	424	1.00E-111	EpoD_Q9L8C7_4mod	Epothilone
KS_706	1259	SPV_cluster_035	52	422	1.00E-115	EpoD_Q9L8C7_4mod	Epothilone
KS_709	1265	SPV_cluster_071	52	424	1.00E-100	EpoD_Q9L8C7_4mod	Epothilone
KS_710	1262	SPV_cluster_114	53	425	4.00E-112	EpoD_Q9L8C7_4mod	Epothilone
KS_711	1259	SPV_cluster_084	48	427	2.00E-103	EpoD_Q9L8C7_2mod	Epothilone
KS_713	1259	SPV_cluster_146	52	427	5.00E-117	EpoD_Q9L8C7_2mod	Epothilone
KS_716	1259	SPV_cluster_020	50	427	1.00E-111	EpoD_Q9L8C7_2mod	Epothilone
KS_717	1265	SPV_cluster_047	48	428	3.00E-101	EpoD_Q9L8C7_2mod	Epothilone
KS_718	1259	SPV_cluster_154	51	423	5.00E-112	EpoD_Q9L8C7_4mod	Epothilone
KS_721	1211	SPV_cluster_149	49	411	1.00E-101	EpoF_Q9L8C5_1mod	Epothilone
KS_722	1259	SPV_cluster_245	54	433	9.00E-120	EpoD_Q9L8C7_4mod	Epothilone
KS_725	1259	SPV_cluster_150	50	423	2.00E-112	EpoD_Q9L8C7_4mod	Epothilone
KS_731	1262	SPV_cluster_151	49	425	2.00E-102	EpoD_Q9L8C7_4mod	Epothilone
KS_734	1259	SPV_cluster_174	50	422	1.00E-104	EpoD_Q9L8C7_4mod	Epothilone
KS_736	1259	SPV_cluster_005	50	424	4.00E-112	NysI_Q9L4X3_5mod	Nystatin
KS_737	1277	SPV_cluster_169	53	435	2.00E-114	EpoD_Q9L8C7_4mod	Epothilone
KS_739	1259	SPV_cluster_102	49	424	6.00E-108	EpoD_Q9L8C7_4mod	Epothilone
KS_740	1262	SPV_cluster_246	50	428	1.00E-105	EpoD_Q9L8C7_4mod	Epothilone
KS_742	1262	SPV_cluster_115	48	424	3.00E-107	TetE_BAE93730_1mod	Tetronomycin
KS_743	1262	SPV_cluster_241	48	424	1.00E-106	TetE_BAE93730_1mod	Tetronomycin
KS_745	1259	SPV_cluster_078	52	424	3.00E-99	EpoD_Q9L8C7_4mod	Epothilone
KS_748	1262	SPV_cluster_168	48	429	2.00E-107	TetE_BAE93730_1mod	Tetronomycin
KS_750	1319	SPV_cluster_008	48	444	6.00E-108	EpoD_Q9L8C7_4mod	Epothilone
KS_752	176	SPV_cluster_457	54	56	5.00E+00	TylGIII_O33956_2mod	Tylosin

KS_753	1262	SPV_cluster_030	54	425	5.00E-122	EpoD_Q9L8C7_2mod	Epothilone
KS_756	1250	SPV_cluster_030	53	428	4.00E-116	EpoD_Q9L8C7_2mod	Epothilone
KS_760	1268	SPV_cluster_029	57	430	1.00E-117	EpoD_Q9L8C7_4mod	Epothilone
KS_762	1277	SPV_cluster_106	51	424	5.00E-112	NysJ_Q9L4X2_2mod	Nystatin

Table S8. BLAST search of the KS sequences from the metagenome of *C. reinwardti* against the nr database in NCBI

KS ID	Length (nt)	BGC ID	Identity (%)	Alignment length (aa)	E-value	Sponge-specific cluster	The homologenus sequences	Accession number
KS_013	792	CLR_cluster_248	75.769	260	3.50E-131	SupA	SupA [Theonella swinhoei bacterial symbiont clone pSW1H8]	ABE03935
KS_029	1194	CLR_cluster_183	83.709	399	0	SupA	SupA [Aplysina aerophoba bacterial symbiont clone pAPKS18]	ABE03915
KS_030	792	CLR_cluster_249	76.154	260	1.52E-131	SupA	SupA [Theonella swinhoei bacterial symbiont clone pSW1H8]	ABE03935
KS_032	327	CLR_cluster_265	81.308	107	6.47E-52	SwfA	Type I polyketide synthase [bacterium symbiont of Plakortis simplex pPS11G3]	AGH13577
KS_039	1269	CLR_cluster_225	64.439	419	4.33E-173	SupA	SupA [uncultured marine bacterium 1k6]	ACX49735
KS_041	975	CLR_cluster_202	61.25	320	1.46E-117		SA1_PKSA [uncultured bacterial symbiont of Discodermia dissoluta]	AAY00025
KS_044	363	CLR_cluster_181	76.471	119	1.32E-54	SupA	SupA [Theonella swinhoei bacterial symbiont clone pSW1H8]	ABE03935
KS_061	1113	CLR_cluster_192	82.289	367	0	SupA	SupA [Theonella swinhoei bacterial symbiont clone pSW1H8]	ABE03935
KS_064	1221	CLR_cluster_021	74.568	405	0		SA1_PKSA [uncultured bacterial symbiont of Discodermia dissoluta]	AAY00025
KS_066	702	CLR_cluster_246	76.623	231	1.76E-111	SupA	SupA [Aplysina aerophoba bacterial symbiont clone pAPKS18]	ABE03915
KS_073	1112	CLR_cluster_222	80.488	369	0		Putative polyketide synthase [uncultured bacterium 2063G]	ABB73286
KS_078	1064	CLR_cluster_252	78.917	351	0		3-Oxoacyl-[acyl-carrier-protein] synthase 2 [Alphaproteobacteria bacterium MarineAlpha10_Bin2]	PPR24356
KS_079	713	CLR_cluster_062	79.638	221	1.43E-110	SwfA	Type I polyketide synthase [bacterium symbiont of Plakortis simplex pPS11G3]	AGH13577
KS_084	977	CLR_cluster_279	49.296	213	1.09E-62		3-Oxoacyl-[acyl-carrier-protein] synthase II [Chloroflexi bacterium]	PZC49279
KS_095	1049	CLR_cluster_281	71.06	349	1.41E-178		Beta-ketoacyl-[acyl-carrier-protein] synthase II [Gammaproteobacteria bacterium]	TDJ41741
KS_101	1100	CLR_cluster_081	52.61	249	6.33E-79		Putative Beta-ketoacyl synthase [Sorangium cellulosum So ce56]	CAN93510
KS_102	1097	CLR_cluster_105	49.57	349	2.55E-96		Putative Beta-ketoacyl synthase [Sorangium cellulosum So ce56]	CAN93510
KS_105	1223	CLR_cluster_122	94.363	408	0		Beta-ketoacyl-[acyl-carrier-protein] synthase II [Candidatus Poribacteria bacterium]	RKU30153
KS_106	1223	CLR_cluster_041	87.5	408	0		Beta-ketoacyl-[acyl-carrier-protein] synthase II [Candidatus Poribacteria bacterium]	RKU31990
KS_111	1223	CLR_cluster_006	82.02	406	0		Beta-ketoacyl-ACP synthase II [Thioalkalivibrio sp. HK1]	WP_038098656
KS_115	1223	CLR_cluster_007	93.857	407	0		Beta-ketoacyl-[acyl-carrier-protein] synthase II [Candidatus Poribacteria bacterium]	RKU31990
KS_116	1202	CLR_cluster_110	73.116	398	0		3-Oxoacyl-ACP synthase [OM182 bacterium BACL3 MAG-120507-bin80]	KRO71573
KS_117	1250	CLR_cluster_086	73.366	413	0		Beta-ketoacyl-ACP synthase II [Rhodospirillales bacterium	WP_027300572

							URHD0088]	
KS_120	1226	CLR_cluster_193	58.333	408	5.12E-174		Beta-ketoacyl-[acyl-carrier-protein] synthase II [SAR202 cluster bacterium Casp-Chloro-G4]	PKB61328
KS_126	1184	CLR_cluster_230	75.949	395	0		Beta-ketoacyl-ACP synthase I [Methylarcula marina]	WP_106692425
KS_134	1199	CLR_cluster_253	67.506	397	0		Beta-ketoacyl-[acyl-carrier-protein] synthase family protein [Halocynthiibacter namhaensis]	WP_039018003
KS_138	1223	CLR_cluster_097	93.366	407	0		Beta-ketoacyl-[acyl-carrier-protein] synthase II [Candidatus Poribacteria bacterium]	RKU30153
KS_142	1208	CLR_cluster_126	78.164	403	0		3-Oxoacyl-[acyl-carrier-protein] synthase 1 [Alphaproteobacteria bacterium MarineAlpha3_Bin7]	PPR64109
KS_146	1223	CLR_cluster_082	75.862	406	0		Beta-ketoacyl-[acyl-carrier-protein] synthase II [Gammaproteobacteria bacterium]	TDJ17613
KS_150	1196	CLR_cluster_127	70.603	398	0		Beta-ketoacyl-[acyl-carrier-protein] synthase I [OM182 bacterium MED-G28]	PDH34065
KS_154	1223	CLR_cluster_013	64.619	407	0		3-Oxoacyl-ACP synthase [candidate division Zixibacteria bacterium SM23_81]	KPL19508
KS_159	1223	CLR_cluster_101	81.818	407	0		Beta-ketoacyl-[acyl-carrier-protein] synthase II [bacterium]	RZP29941
KS_163	1229	CLR_cluster_065	68.447	412	0		Beta-ketoacyl-[acyl-carrier-protein] synthase II [Gemmatimonadetes bacterium]	RMH15744
KS_170	1217	CLR_cluster_085	69.136	405	0		3-Oxoacyl-[acyl-carrier-protein] synthase 2 [bacterium HR37]	GBD38054
KS_179	1202	CLR_cluster_257	73.684	399	0		Beta-ketoacyl-[acyl-carrier-protein] synthase I [Gammaproteobacteria bacterium RIFCSPLOWO2_02_FULL_61_13]	OGT79717
KS_183	1223	CLR_cluster_165	94.103	407	0		Beta-ketoacyl-[acyl-carrier-protein] synthase II [Candidatus Poribacteria bacterium]	RKU10113
KS_196	1280	CLR_cluster_139	64.623	424	1.87E-173		Hypothetical protein A3I72_03985 [Candidatus Tectomicrobia bacterium RIFCSPLOWO2_02_FULL_70_19]	OGL60875
KS_198	1223	CLR_cluster_001	71.253	407	0		MULTISPECIES: Beta-ketoacyl-ACP synthase II [Ectothiorhodospira]	WP_025281708
KS_199	1157	CLR_cluster_264	62.565	382	8.48E-158		Beta-ketoacyl synthase [Actinobacteria bacterium]	RUA23523
KS_201	1226	CLR_cluster_022	61.52	408	7.39E-180		Beta-ketoacyl synthase [Nitrosomonas europaea]	KXK43820
KS_204	1163	CLR_cluster_064	68.325	382	0		Beta-ketoacyl-[acyl-carrier-protein] synthase II [Gammaproteobacteria bacterium]	TDJ41741
KS_206	1226	CLR_cluster_143	62.162	407	0		Beta-ketoacyl-[acyl-carrier-protein] synthase II [Proteobacteria bacterium]	PZN30994
KS_208	1223	CLR_cluster_179	68.395	405	0		Beta-ketoacyl-[acyl-carrier-protein] synthase II [Acidobacteria bacterium]	RPH64142
KS_212	1217	CLR_cluster_190	69.136	405	0		3-Oxoacyl-[acyl-carrier-protein] synthase 2 [bacterium HR37]	GBD38054
KS_217	1217	CLR_cluster_015	70.968	403	0		Beta-ketoacyl-[acyl-carrier-protein] synthase II [Gammaproteobacteria bacterium]	TDJ36329
KS_221	1064	CLR_cluster_102	70.566	265	1.35E-131		3-Oxoacyl-ACP synthase [Ralstonia pickettii DTP0602]	AGW95093

KS_222	1229	CLR_cluster_134	58.477	407	1.39E-150		Beta-ketoacyl-[acyl-carrier-protein] synthase II [Deltaproteobacteria bacterium]	TDJ21427
KS_227	1217	CLR_cluster_220	69.136	405	0		Beta-ketoacyl-[acyl-carrier-protein] synthase II [Gammaproteobacteria bacterium RBG_16_51_14]	OGT30519
KS_231	1217	CLR_cluster_049	69.458	406	0		Beta-ketoacyl-[acyl-carrier-protein] synthase II [Gammaproteobacteria bacterium RBG_16_51_14]	OGT30519
KS_232	1223	CLR_cluster_224	67.734	406	0		Beta-ketoacyl-[acyl-carrier-protein] synthase II [Gammaproteobacteria bacterium]	TDJ29040
KS_239	1226	CLR_cluster_166	61.425	407	4.00E-179		MULTISPECIES: Beta-ketoacyl-ACP synthase II [Pseudomonadaceae]	WP_096004673
KS_247	1223	CLR_cluster_162	71.429	406	0		Beta-ketoacyl-[acyl-carrier-protein] synthase II [OM182 bacterium MED-G28]	PDH35184
KS_250	1340	CLR_cluster_014	53.437	451	7.41E-146		Hypothetical protein D7X12_09075, partial [Corallococcus sicarius]	RKH45034
KS_253	1229	CLR_cluster_228	65.366	410	0		Beta-ketoacyl-[acyl-carrier-protein] synthase II [Gemmatimonadetes bacterium]	RMH15744
KS_262	1220	CLR_cluster_045	68.966	406	0		Beta-ketoacyl-[acyl-carrier-protein] synthase II [Acidobacteria bacterium]	RMH20421
KS_263	1292	CLR_cluster_076	78.322	429	0	SwfA	Type I polyketide synthase [bacterium symbiont of Plakortis simplex pPSA11D7]	AGH13590
KS_266	1151	CLR_cluster_140	78.272	382	0		Beta-ketoacyl synthase [Acidimicrobiales bacterium MED-G01]	PDH69975
KS_279	1229	CLR_cluster_147	62.347	409	0		Beta-ketoacyl-ACP synthase II [Nitrosomonas aestuarii]	WP_090702733
KS_283	1286	CLR_cluster_131	64.371	421	0		SDR family NAD(P)-dependent oxidoreductase [Cocleimonas flava]	WP_131906443
KS_284	1292	CLR_cluster_108	83.411	428	0	SwfA	Type I polyketide synthase [bacterium symbiont of Plakortis simplex pPSA11D7]	AGH13590
KS_294	1223	CLR_cluster_060	69.779	407	0		Beta-ketoacyl-[acyl-carrier-protein] synthase II [Polyangiaceae bacterium UTPRO1]	OQY68774
KS_302	1154	CLR_cluster_043	51.558	353	8.38E-110		Hypothetical protein [Pelagimonas phthalica]	WP_099241578
KS_309	1160	CLR_cluster_114	70.605	347	1.84E-166		Beta-ketoacyl synthase [Actinobacteria bacterium]	RUA23523
KS_311	1259	CLR_cluster_137	71.599	419	0		Beta-ketoacyl-[acyl-carrier-protein] synthase family protein [Deltaproteobacteria bacterium]	TDJ13656
KS_314	1160	CLR_cluster_214	70.893	347	1.48E-168		Beta-ketoacyl synthase [Actinobacteria bacterium]	RUA23523
KS_322	1214	CLR_cluster_098	67.822	404	0		Beta-ketoacyl-[acyl-carrier-protein] synthase II [Chloroflexi bacterium]	RME76096
KS_325	1286	CLR_cluster_056	80.328	427	0		Putative polyketide synthase [uncultured bacterium 2063G]	ABB73286
KS_327	1154	CLR_cluster_009	53.786	383	3.88E-131		Hypothetical protein [Pelagimonas phthalica]	WP_099241578
KS_329	1160	CLR_cluster_069	69.713	383	0		Beta-ketoacyl synthase [Actinobacteria bacterium]	RUA23523
KS_330	1223	CLR_cluster_201	66.585	407	0		Beta-ketoacyl-[acyl-carrier-protein] synthase II [Chloroflexi bacterium]	TDI92680
KS_334	1229	CLR_cluster_079	67.397	411	0		Beta-ketoacyl-[acyl-carrier-protein] synthase II [Gemmatimonadetes bacterium]	RMH15744

KS_338	1292	CLR_cluster_133	75.058	429	0	SwfA	Type I polyketide synthase [bacterium symbiont of Plakortis simplex pPSA11D7]	AGH13590
KS_340	1154	CLR_cluster_118	59.791	383	1.03E-161		Beta-ketoacyl synthase [Acidimicrobiaceae bacterium TMED77]	OUV00831
KS_342	1292	CLR_cluster_089	75.991	429	0	SwfA	Type I polyketide synthase [bacterium symbiont of Plakortis simplex pPSA11D7]	AGH13590
KS_346	1229	CLR_cluster_096	69.343	411	0		Beta-ketoacyl-[acyl-carrier-protein] synthase II [Gemmatimonadetes bacterium]	RMH15744
KS_354	1232	CLR_cluster_231	70	410	0		Beta-ketoacyl-ACP synthase II [Rhodothermus marinus]	WP_014068140
KS_358	1292	CLR_cluster_055	71.852	405	0	SwfA	Type I polyketide synthase [bacterium symbiont of Plakortis simplex pPSA11D7]	AGH13590
KS_364	1292	CLR_cluster_027	61.593	427	7.04E-175	SwfA	Type I polyketide synthase [bacterium symbiont of Plakortis simplex pPSA11D7]	AGH13590
KS_366	1229	CLR_cluster_025	67.64	411	0		Beta-ketoacyl-[acyl-carrier-protein] synthase II [Gemmatimonadetes bacterium]	RMH15744
KS_368	1247	CLR_cluster_204	75.061	413	0		3-Oxoacyl-[acyl-carrier-protein] synthase 2 [Alphaproteobacteria bacterium MarineAlpha10_Bin2]	PPR24356
KS_372	1298	CLR_cluster_132	78.089	429	0	SwfA	Type I polyketide synthase [bacterium symbiont of Plakortis simplex pPSA11D7]	AGH13590
KS_373	1220	CLR_cluster_030	76.733	404	0		Beta-ketoacyl-[acyl-carrier-protein] synthase II [Nitrospirae bacterium]	TAJ27615
KS_375	1100	CLR_cluster_032	52.381	252	7.01E-80		Putative Beta-ketoacyl synthase [Sorangium cellulosum So ce56]	CAN93510
KS_376	1007	CLR_cluster_275	60.778	334	1.05E-148		Beta-ketoacyl-[acyl-carrier-protein] synthase II [Chloroflexi bacterium TMED70]	OUU77203
KS_382	1292	CLR_cluster_087	77.622	429	0	SwfA	Type I polyketide synthase [bacterium symbiont of Plakortis simplex pPSA11D7]	AGH13590
KS_384	1220	CLR_cluster_261	62.654	407	0		Beta-ketoacyl-[acyl-carrier-protein] synthase II [Chloroflexi bacterium]	RMG78893
KS_393	1247	CLR_cluster_200	71.498	414	0		Beta-ketoacyl-ACP synthase II [Rhodospirillaceae bacterium SYSU D60014]	WP_119459592
KS_395	995	CLR_cluster_262	75.229	327	4.79E-168		3-Oxoacyl-[acyl-carrier-protein] synthase 2 [bacterium HR40]	GBD44717
KS_403	1286	CLR_cluster_135	91.569	427	0		Putative polyketide synthase [uncultured bacterium 2063G]	ABB73286
KS_407	1184	CLR_cluster_010	63.265	392	9.60E-171		Beta-ketoacyl-[acyl-carrier-protein] synthase family protein [Pelagimonas phthalicica]	WP_099241574
KS_412	1223	CLR_cluster_191	58.333	408	4.30E-179		Beta-ketoacyl-[acyl-carrier-protein] synthase II [Chloroflexi bacterium RBG_16_64_32]	OGO48822
KS_413	1184	CLR_cluster_042	62.5	392	8.30E-169		Beta-ketoacyl-[acyl-carrier-protein] synthase family protein [Pelagimonas phthalicica]	WP_099241574
KS_414	1292	CLR_cluster_106	88.038	418	0	SwfA	Type I polyketide synthase [bacterium symbiont of Plakortis simplex pPS11G3]	AGH13577
KS_417	965	CLR_cluster_251	68.339	319	6.17E-152		Beta-ketoacyl-[acyl-carrier-protein] synthase II [Chloroflexi bacterium]	RME76096
KS_419	1286	CLR_cluster_212	80.751	426	0	SupA	SupA [uncultured marine bacterium 1k6]	ACX49735

KS_421	710	CLR_cluster_278	77.637	237	3.95E-118	SupA	SupA [Theonella swinhoei bacterial symbiont clone pSW1H8]	ABE03935
KS_423	1064	CLR_cluster_218	91.193	352	0	SupA	SupA [Aplysina aerophoba bacterial symbiont clone pAPKS18]	ABE03915
KS_424	1211	CLR_cluster_104	76.19	399	0		Beta-ketoacyl-ACP synthase II [Rhodospirillaceae bacterium SYSU D60014]	WP_119459592
KS_428	1292	CLR_cluster_053	76.923	429	0	SwfA	Type I polyketide synthase [bacterium symbiont of Plakortis simplex pPSA11D7]	AGH13590
KS_431	1235	CLR_cluster_184	68.856	411	0		Beta-ketoacyl-[acyl-carrier-protein] synthase II [Gemmatimonadetes bacterium]	RMH15744
KS_440	1286	CLR_cluster_219	82.904	427	0		Putative polyketide synthase [uncultured bacterium 2063G]	ABB73286
KS_447	1292	CLR_cluster_177	88.095	420	0	SwfA	Type I polyketide synthase [bacterium symbiont of Plakortis simplex pPS11G3]	AGH13577
KS_454	1184	CLR_cluster_121	72.237	389	0		3-Oxoacyl-ACP synthase [Candidatus Tenderia electrophaga]	ALP54806
KS_455	1286	CLR_cluster_203	67.529	425	0	SupA	SupA [Aplysina aerophoba bacterial symbiont clone pAPKS18]	ABE03915
KS_461	1211	CLR_cluster_145	74.121	398	0		Beta-ketoacyl-ACP synthase II [Telmatospirillum siberiense]	WP_101252614
KS_465	983	CLR_cluster_141	67.485	326	3.70E-159		Beta-ketoacyl-ACP synthase II [Bryobacter aggregatus]	WP_031496688
KS_466	1190	CLR_cluster_128	69.424	399	0		Beta-ketoacyl-ACP synthase II [Pararhodobacter aggregans]	WP_107749575
KS_473	1175	CLR_cluster_012	81.538	390	0		Beta-ketoacyl-ACP synthase II [Thioalkalivibrio sp. HK1]	WP_038098656
KS_479	1184	CLR_cluster_112	71.688	385	0		Beta-ketoacyl-[acyl-carrier-protein] synthase II [Candidatus Handelsmanbacteria bacterium RIFCSPLOWO2_12_FULL_64_10]	OGG49603
KS_483	1112	CLR_cluster_017	74.857	350	0		Beta-ketoacyl synthase [Acidimicrobiales bacterium MED-G01]	PDH69975
KS_488	1076	CLR_cluster_277	61.281	359	2.06E-157		Beta-ketoacyl-[acyl-carrier-protein] synthase II [Candidatus Handelsmanbacteria bacterium RIFCSPLOWO2_12_FULL_64_10]	OGG49603
KS_503	1286	CLR_cluster_174	78.454	427	0	SupA	SupA [Theonella swinhoei bacterial symbiont clone pSW1H8]	ABE03935
KS_504	1157	CLR_cluster_176	75.198	379	0	SwfA	Type I polyketide synthase [bacterium symbiont of Plakortis simplex pPSA11D7]	AGH13590
KS_505	1286	CLR_cluster_005	79.859	427	0	SupA	SupA [Theonella swinhoei bacterial symbiont clone pSW1H8]	ABE03935
KS_508	1292	CLR_cluster_107	87.857	420	0	SwfA	Type I polyketide synthase [bacterium symbiont of Plakortis simplex pPS11G3]	AGH13577
KS_514	1184	CLR_cluster_072	72.843	394	0		Beta-ketoacyl-[acyl-carrier-protein] synthase II [Candidatus Tectomicrobia bacterium RIFCSPLOWO2_12_FULL_69_37]	OGL61959
KS_518	1244	CLR_cluster_144	72.104	423	0		SA1_PKSA [uncultured bacterial symbiont of Discodermia dissoluta]	AAY00025
KS_521	1220	CLR_cluster_156	63.027	403	0		Beta-ketoacyl-ACP synthase II [Albimonas donghaensis]	WP_092679786
KS_527	1286	CLR_cluster_149	80.986	426	0	SupA	SupA [Aplysina aerophoba bacterial symbiont clone pAPKS18]	ABE03915
KS_528	1292	CLR_cluster_024	82.904	427	0	SupA	SupA [Theonella swinhoei bacterial symbiont clone pSW1H8]	ABE03935
KS_536	1292	CLR_cluster_029	80.094	427	0	SupA	SupA [Theonella swinhoei bacterial symbiont clone pSW1H8]	ABE03935

KS_537	1286	CLR_cluster_153	76.581	427	0	SupA	SupA [Theonella swinhoei bacterial symbiont clone pSW1H8]	ABE03935
KS_540	1286	CLR_cluster_040	80.094	427	0	SupA	SupA [Theonella swinhoei bacterial symbiont clone pSW1H8]	ABE03935
KS_546	1286	CLR_cluster_159	88.263	426	0	SupA	Polyketide synthase, partial [Aplysina aerophoba bacterial symbiont clone pAE27P20]	ABE03895
KS_549	1283	CLR_cluster_123	87.324	426	0	SupA	SupA [Aplysina aerophoba bacterial symbiont clone pAPKS18]	ABE03915
KS_550	1277	CLR_cluster_066	74.533	428	0		SA1_PKSA [uncultured bacterial symbiont of Discodermia dissoluta]	AAY00025
KS_557	1301	CLR_cluster_194	75	428	0	SupA	SupA [Aplysina aerophoba bacterial symbiont clone pAPKS18]	ABE03915
KS_558	1250	CLR_cluster_059	70.29	414	1.36E-178		SA1_PKSA [uncultured bacterial symbiont of Discodermia dissoluta]	AAY00025
KS_563	1292	CLR_cluster_034	86.651	427	0	SupA	SupA [Theonella swinhoei bacterial symbiont clone pSW1H8]	ABE03935
KS_564	1286	CLR_cluster_124	82.436	427	0	SupA	SupA [Theonella swinhoei bacterial symbiont clone pSW1H8]	ABE03935
KS_576	1094	CLR_cluster_232	60.665	361	1.16E-162		Beta-ketoacyl-[acyl-carrier-protein] synthase II [Chloroflexi bacterium]	PZN55531
KS_579	1178	CLR_cluster_008	61.675	394	3.52E-156		Beta-ketoacyl-[acyl-carrier-protein] synthase family protein [Verminephrobacter aporrectodeae]	WP_081479930
KS_580	1010	CLR_cluster_070	55.67	194	3.63E-63		Beta-ketoacyl-ACP synthase [Xanthomonadaceae bacterium]	RYD16404
KS_585	989	CLR_cluster_221	66.116	242	1.98E-105		Beta-ketoacyl-[acyl-carrier-protein] synthase family protein [Parasulfuritortus cantonensis]	WP_131445552
KS_586	1184	CLR_cluster_044	59.836	366	3.15E-136		Beta-ketoacyl-[acyl-carrier-protein] synthase family protein [Verminephrobacter aporrectodeae]	WP_081479930
KS_590	1094	CLR_cluster_020	79.945	364	0		SA1_PKSA [uncultured bacterial symbiont of Discodermia dissoluta]	AAY00025
KS_591	1283	CLR_cluster_026	74.941	423	0		SA1_PKSA [uncultured bacterial symbiont of Discodermia dissoluta]	AAY00025
KS_602	722	CLR_cluster_247	79.412	238	5.35E-118	SwfA	Type I polyketide synthase [bacterium symbiont of Plakortis simplex pPS11G3]	AGH13577
KS_605	851	CLR_cluster_109	72.587	259	1.25E-129		Beta-ketoacyl synthase [Acidobacteria bacterium]	PYQ67687
KS_607	653	CLR_cluster_068	79.07	215	3.73E-108		Putative polyketide synthase [uncultured bacterium 2063G]	ABB73286
KS_608	599	CLR_cluster_234	88.265	196	3.09E-109	SupA	SupA [Theonella swinhoei bacterial symbiont clone pSW1H8]	ABE03935
KS_609	545	CLR_cluster_163	84.831	178	1.29E-91		Putative polyketide synthase [uncultured bacterium 2063G]	ABB73286
KS_611	449	CLR_cluster_235	84.459	148	1.14E-77	SwfA	Type I polyketide synthase [bacterium symbiont of Plakortis simplex pPS11G3]	AGH13577
KS_613	362	CLR_cluster_180	75.63	119	1.13E-53	SupA	SupA [Theonella swinhoei bacterial symbiont clone pSW1H8]	ABE03935
KS_615	1262	CLR_cluster_197	69.928	419	0	SupA	SupA [Theonella swinhoei bacterial symbiont clone pSW1H8]	ABE03935
KS_616	341	CLR_cluster_245	75.676	111	7.95E-51	SupA	SupA [Theonella swinhoei bacterial symbiont clone pSW1H8]	ABE03935
KS_617	233	CLR_cluster_250	77.333	75	4.24E-30	SupA	SupA [Theonella swinhoei bacterial symbiont clone pSW1H8]	ABE03935
KS_618	233	CLR_cluster_255	77.333	75	3.59E-30	SupA	SupA [Theonella swinhoei bacterial symbiont clone pSW1H8]	ABE03935

KS_619	245	CLR_cluster_268	77.5	80	2.20E-34	SupA	SupA [Theonella swinhoei bacterial symbiont clone pSW1H8]	ABE03935
KS_620	1259	CLR_cluster_125	80.336	417	0		Putative polyketide synthase [uncultured bacterium 2063G]	ABB73286
KS_626	1265	CLR_cluster_136	84.928	418	0	SupA	SupA [Theonella swinhoei bacterial symbiont clone pSW1H8]	ABE03935
KS_630	1259	CLR_cluster_175	80.096	417	0		Putative polyketide synthase [uncultured bacterium 2063G]	ABB73286
KS_633	1265	CLR_cluster_093	75.177	423	0		SA1_PKSA [uncultured bacterial symbiont of Discodermia dissoluta]	AAY00025
KS_637	1262	CLR_cluster_160	76.123	423	0		SA1_PKSA [uncultured bacterial symbiont of Discodermia dissoluta]	AAY00025
KS_640	1259	CLR_cluster_154	71.223	417	0	SupA	SupA [Aplysina aerophoba bacterial symbiont clone pAPKS18]	ABE03915
KS_644	1256	CLR_cluster_026	75.352	426	0		SA1_PKSA [uncultured bacterial symbiont of Discodermia dissoluta]	AAY00025
KS_645	1259	CLR_cluster_161	81.818	418	0	SupA	Polyketide synthase, partial [Aplysina aerophoba bacterial symbiont clone pAE27P20]	ABE03895
KS_646	1256	CLR_cluster_117	75.12	418	0	SupA	SupA [Theonella swinhoei bacterial symbiont clone pSW1H8]	ABE03935
KS_649	1259	CLR_cluster_080	70.504	417	0	SupA	SupA [Aplysina aerophoba bacterial symbiont clone pAPKS18]	ABE03915
KS_654	1244	CLR_cluster_058	66.118	425	2.01E-175		SA1_PKSA [uncultured bacterial symbiont of Discodermia dissoluta]	AAY00025
KS_659	1259	CLR_cluster_047	75.65	423	0		SA1_PKSA [uncultured bacterial symbiont of Discodermia dissoluta]	AAY00025
KS_661	1265	CLR_cluster_103	63.397	418	7.15E-173	SupA	SupA [Theonella swinhoei bacterial symbiont clone pSW1H8]	ABE03935
KS_669	1235	CLR_cluster_208	71.359	412	0	SupA	SupA [Theonella swinhoei bacterial symbiont clone pSW1H8]	ABE03935
KS_671	1259	CLR_cluster_048	68.106	417	0	SupA	SupA [Aplysina aerophoba bacterial symbiont clone pAPKS18]	ABE03915
KS_674	1268	CLR_cluster_020	74.292	424	0		SA1_PKSA [uncultured bacterial symbiont of Discodermia dissoluta]	AAY00025
KS_681	1277	CLR_cluster_075	73.995	423	0		SA1_PKSA [uncultured bacterial symbiont of Discodermia dissoluta]	AAY00025
KS_683	1268	CLR_cluster_075	74.704	423	0		SA1_PKSA [uncultured bacterial symbiont of Discodermia dissoluta]	AAY00025
KS_688	1259	CLR_cluster_068	68.106	417	0		SupA [Aplysina aerophoba bacterial symbiont clone pAPKS18]	ABE03915
KS_695	1259	CLR_cluster_056	80.096	417	0		Putative polyketide synthase [uncultured bacterium 2063G]	ABB73286
KS_701	1271	CLR_cluster_173	74.232	423	0		SA1_PKSA [uncultured bacterial symbiont of Discodermia dissoluta]	AAY00025
KS_705	1259	CLR_cluster_206	72.249	418	0	SupA	SupA [Theonella swinhoei bacterial symbiont clone pSW1H8]	ABE03935
KS_708	1262	CLR_cluster_242	82.297	418	0	SupA	SupA [Theonella swinhoei bacterial symbiont clone pSW1H8]	ABE03935
KS_720	1259	CLR_cluster_155	70.743	417	0	SupA	SupA [Aplysina aerophoba bacterial symbiont clone pAPKS18]	ABE03915
KS_726	1259	CLR_cluster_005	73.684	418	0	SupA	SupA [Theonella swinhoei bacterial symbiont clone pSW1H8]	ABE03935
KS_728	1259	CLR_cluster_057	87.05	417	0	SupA	SupA [Aplysina aerophoba bacterial symbiont clone pAPKS18]	ABE03915
KS_730	1265	CLR_cluster_040	68.9	418	0	SupA	SupA [Theonella swinhoei bacterial symbiont clone pSW1H8]	ABE03935

KS_732	1262	CLR_cluster_034	74.224	419	0	SupA	Polyketide synthase, partial [Aplysina aerophoba bacterial symbiont clone pAE27P20]	ABE03895
KS_747	1109	CLR_cluster_146	79.564	367	0		SA1_PKSA [uncultured bacterial symbiont of Discodermia dissoluta]	AAY00025
KS_749	1319	CLR_cluster_050	64.76	437	2.71E-174	SupA	SupA [Aplysina aerophoba bacterial symbiont clone pAPKS18]	ABE03915
KS_751	224	CLR_cluster_130	70.833	72	6.11E-26		Putative polyketide synthase [uncultured bacterium 2063G]	ABB73286
KS_755	1262	CLR_cluster_047	75.887	423	0		SA1_PKSA [uncultured bacterial symbiont of Discodermia dissoluta]	AAY00025
KS_757	1250	CLR_cluster_020	72.471	425	0		SA1_PKSA [uncultured bacterial symbiont of Discodermia dissoluta]	AAY00025
KS_761	1277	CLR_cluster_026	67.376	423	0		SA1_PKSA [uncultured bacterial symbiont of Discodermia dissoluta]	AAY00025

Table S9. BLAST search of the KS sequences from the metagenome of *R. globostellata* against the nr database in NCBI

KS ID	Length (nt)	BGC ID	Identity (%)	Alignment length (aa)	E-value	Sponge-specific cluster	The homologenus sequences	Accession number
KS_001	696	RHG_cluster_258	89.52	229	1.64E-130	SupA	SupA [Aplysina aerophoba bacterial symbiont clone pAPKS18]	ABE03915
KS_004	1185	RHG_cluster_332	68.608	395	0		Beta-ketoacyl-[acyl-carrier-protein] synthase II [Chloroflexi bacterium]	RLT43681
KS_006	1128	RHG_cluster_316	65.193	362	1.66E-155	SupA	SupA [Theonella swinhoei bacterial symbiont clone pSW1H8]	ABE03935
KS_007	1059	RHG_cluster_341	73.547	344	5.51E-163	SwfA	Type I polyketide synthase [bacterium symbiont of Plakortis simplex pPSA11D7]	AGH13590
KS_010	1176	RHG_cluster_342	71.85	373	7.92E-175	SupA	SupA [Theonella swinhoei bacterial symbiont clone pSW1H8]	ABE03935
KS_011	801	RHG_cluster_340	56.667	270	3.28E-94		Type I polyketide synthase, partial [Lentisphaerales bacterium]	TFH18080
KS_012	429	RHG_cluster_282	66.667	138	5.19E-57	SupA	SupA [Theonella swinhoei bacterial symbiont clone pSW1H8]	ABE03935
KS_014	1086	RHG_cluster_275	63.435	361	1.84E-170		Beta-ketoacyl-[acyl-carrier-protein] synthase II [Chloroflexi bacterium TMED230]	OUW96072
KS_016	1239	RHG_cluster_191	67.073	410	8.81E-169		SA1_PKSA [uncultured bacterial symbiont of Discodermia dissoluta]	AAY00025
KS_019	1017	RHG_cluster_352	77.811	338	0		Beta-ketoacyl-[acyl-carrier-protein] synthase I [Gammaproteobacteria bacterium RIFCSPLOWO2_02_FULL_61_13]	OGT79717
KS_020	705	RHG_cluster_308	74.236	229	2.69E-113	SupA	SupA [Theonella swinhoei bacterial symbiont clone pSW1H8]	ABE03935
KS_021	771	RHG_cluster_354	71.146	253	1.08E-110	SwfA	Type I polyketide synthase [bacterium symbiont of Plakortis simplex pPS11G3]	AGH13577
KS_026	1080	RHG_cluster_321	68.156	358	0		Beta-ketoacyl-[acyl-carrier-protein] synthase II [Deltaproteobacteria bacterium RIFOXYA2_FULL_55_11]	OGQ91897
KS_028	591	RHG_cluster_221	75.258	194	7.05E-89	SupA	SupA [Aplysina aerophoba bacterial symbiont clone pAPKS18]	ABE03915
KS_033	1266	RHG_cluster_087	80.523	421	0	SupA	SupA [uncultured marine bacterium 1k6]	ACX49735
KS_036	792	RHG_cluster_345	72.481	258	8.15E-122	SupA	SupA [Theonella swinhoei bacterial symbiont clone pSW1H8]	ABE03935
KS_037	390	RHG_cluster_351	85.039	127	5.54E-64	SupA	SupA [Aplysina aerophoba bacterial symbiont clone pAPKS18]	ABE03915
KS_040	1038	RHG_cluster_295	88.663	344	0		Putative polyketide synthase [uncultured bacterium 2063G]	ABB73286
KS_043	540	RHG_cluster_283	84.831	178	2.92E-89	SwfA	Type I polyketide synthase [bacterium symbiont of Plakortis simplex pPSA11D7]	AGH13590
KS_045	741	RHG_cluster_338	81.743	241	1.20E-124	SupA	SupA [Theonella swinhoei bacterial symbiont clone pSW1H8]	ABE03935
KS_046	354	RHG_cluster_362	81.739	115	1.59E-56	SwfA	Type I polyketide synthase [bacterium symbiont of Plakortis simplex pPS11G3]	AGH13577
KS_048	1266	RHG_cluster_136	74.048	420	0	SwfA	Type I polyketide synthase [bacterium symbiont of Plakortis simplex pPSA11D7]	AGH13590
KS_052	285	RHG_cluster_367	64.835	91	1.60E-34		SA1_PKSB [uncultured bacterial symbiont of Discodermia dissoluta]	AAY00026

KS_053	543	RHG_cluster_357	86.441	177	2.91E-95	SupA	SupA [Aplysina aerophoba bacterial symbiont clone pAPKS18]	ABE03915
KS_054	525	RHG_cluster_356	88.953	172	8.75E-96	SupA	SupA [Aplysina aerophoba bacterial symbiont clone pAPKS18]	ABE03915
KS_056	1008	RHG_cluster_355	75.821	335	0		Beta-ketoacyl-[acyl-carrier-protein] synthase II, partial [Bacteroidetes bacterium]	RMF59781
KS_060	444	RHG_cluster_183	80.137	146	8.94E-72	SwfA	Type I polyketide synthase [bacterium symbiont of Plakortis simplex pPS11G3]	AGH13577
KS_062	657	RHG_cluster_318	74.074	216	4.61E-101	SupA	SupA [Theonella swinhoei bacterial symbiont clone pSW1H8]	ABE03935
KS_063	1230	RHG_cluster_019	72.422	417	0		SA1_PKSA [uncultured bacterial symbiont of Discodermia dissoluta]	AAY00025
KS_065	591	RHG_cluster_255	75.258	194	3.22E-88	SupA	SupA [Aplysina aerophoba bacterial symbiont clone pAPKS18]	ABE03915
KS_067	1272	RHG_cluster_124	82.185	421	0	SupA	SupA [Aplysina aerophoba bacterial symbiont clone pAPKS18]	ABE03915
KS_069	825	RHG_cluster_168	74.632	272	3.07E-131	SupA	SupA [Theonella swinhoei bacterial symbiont clone pSW1H8]	ABE03935
KS_071	1152	RHG_cluster_294	72.299	361	7.87E-172	SupA	SupA [Theonella swinhoei bacterial symbiont clone pSW1H8]	ABE03935
KS_074	1187	RHG_cluster_151	66.667	396	0		Beta-ketoacyl-ACP synthase II [Bryobacter aggregatus]	WP_031496688
KS_075	1136	RHG_cluster_164	87.464	351	0	SupA	SupA [Aplysina aerophoba bacterial symbiont clone pAPKS18]	ABE03915
KS_077	1097	RHG_cluster_310	73.889	360	0		3-Oxoacyl-[acyl-carrier-protein] synthase2C KASII [gamma proteobacterium IMCC2047]	EGG99443
KS_080	1094	RHG_cluster_343	66.575	365	2.31E-170		3-Oxoacyl-[acyl-carrier-protein] synthase II [Chloroflexi bacterium]	PZC47099
KS_083	1130	RHG_cluster_336	66.216	370	3.15E-156	SupA	SupA [Theonella swinhoei bacterial symbiont clone pSW1H8]	ABE03935
KS_088	893	RHG_cluster_279	82.414	290	1.78E-155	SupA	SupA [Theonella swinhoei bacterial symbiont clone pSW1H8]	ABE03935
KS_091	977	RHG_cluster_119	78.505	321	2.18E-159		SA1_PKSA [uncultured bacterial symbiont of Discodermia dissoluta]	AAY00025
KS_094	1097	RHG_cluster_111	49.57	349	2.55E-96		Putative Beta-ketoacyl synthase [Sorangium cellulosum So ce56]	CAN93510
KS_096	1097	RHG_cluster_116	51.734	346	1.27E-98		Putative Beta-ketoacyl synthase [Sorangium cellulosum So ce56]	CAN93510
KS_098	1100	RHG_cluster_184	52.61	249	6.33E-79		Putative Beta-ketoacyl synthase [Sorangium cellulosum So ce56]	CAN93510
KS_103	1007	RHG_cluster_365	71.56	327	2.23E-154		Beta-ketoacyl-[acyl-carrier-protein] synthase II [Chloroflexi bacterium]	RLT35298
KS_104	1223	RHG_cluster_007	82.02	406	0		Beta-ketoacyl-ACP synthase II [Thioalkalivibrio sp. HK1]	WP_038098656
KS_107	1223	RHG_cluster_018	93.857	407	0		Beta-ketoacyl-[acyl-carrier-protein] synthase II [Candidatus Poribacteria bacterium]	RKU31990
KS_108	1217	RHG_cluster_278	95.05	404	0		Beta-ketoacyl-ACP synthase I [Rhodospirillaceae bacterium Spongia-Bin9]	WP_108794197
KS_109	1208	RHG_cluster_153	78.164	403	0		3-Oxoacyl-[acyl-carrier-protein] synthase 1 [Alphaproteobacteria bacterium MarineAlpha3_Bin7]	PPR64109
KS_110	1202	RHG_cluster_073	73.116	398	0		3-Oxoacyl-ACP synthase [OM182 bacterium BACL3 MAG-120507-bin80]	KRO71573
KS_112	1250	RHG_cluster_127	79.177	413	0		Beta-ketoacyl-ACP synthase II [Rhodospirillaceae bacterium SYSU D60014]	WP_119459592

KS_113	1259	RHG_cluster_237	61.244	418	5.64E-164	SupA	SupA [Theonella swinhoei bacterial symbiont clone pSW1H8]	ABE03935
KS_130	1091	RHG_cluster_315	70.055	364	0		Beta-ketoacyl-[acyl-carrier-protein] synthase II [Gammaproteobacteria bacterium]	TDJ41741
KS_131	1226	RHG_cluster_098	58.333	408	5.12E-174		Beta-ketoacyl-[acyl-carrier-protein] synthase II [SAR202 cluster bacterium Casp-Chloro-G4]	PKB61328
KS_135	1199	RHG_cluster_033	67.758	397	0		Beta-ketoacyl-[acyl-carrier-protein] synthase family protein [Rhodovulum sp. MB263]	WP_080616320
KS_137	1184	RHG_cluster_156	77.215	395	0		Beta-ketoacyl-ACP synthase I [Albidovulum inexpectatum]	WP_104068930
KS_140	1250	RHG_cluster_277	73.366	413	0		Beta-ketoacyl-ACP synthase II [Rhodospirillales bacterium URHD0088]	WP_027300572
KS_143	1223	RHG_cluster_049	75.862	406	0		Beta-ketoacyl-[acyl-carrier-protein] synthase II [Gammaproteobacteria bacterium]	TDJ17613
KS_144	1223	RHG_cluster_138	93.366	407	0		Beta-ketoacyl-[acyl-carrier-protein] synthase II [Candidatus Poribacteria bacterium]	RKU30153
KS_148	1214	RHG_cluster_231	67.782	239	1.81E-117		Beta-ketoacyl-ACP synthase [Aeromonas sp. HMWF016]	WP_108570661
KS_151	1196	RHG_cluster_250	70.603	398	0		Beta-ketoacyl-[acyl-carrier-protein] synthase I [OM182 bacterium MED-G28]	PDH34065
KS_153	1250	RHG_cluster_079	72.488	418	0		Hypothetical protein A3I72_04005 [Candidatus Tectomicrobia bacterium RIFCSPLOWO2_02_FULL_70_19]	OGL60879
KS_161	1073	RHG_cluster_292	77.933	358	0		Beta-ketoacyl-ACP synthase I [Gammaproteobacteria bacterium]	RLA45169
KS_162	1202	RHG_cluster_108	73.684	399	0		Beta-ketoacyl-[acyl-carrier-protein] synthase I [Gammaproteobacteria bacterium RIFCSPLOWO2_02_FULL_61_13]	OGT79717
KS_166	1223	RHG_cluster_023	82.555	407	0		Beta-ketoacyl-[acyl-carrier-protein] synthase II [bacterium]	RZP29941
KS_167	1151	RHG_cluster_271	73.958	384	0		Beta-ketoacyl-[acyl-carrier-protein] synthase II [Acidobacteria bacterium]	TDI09580
KS_173	1253	RHG_cluster_326	71.053	418	0	SupA	SupA [Theonella swinhoei bacterial symbiont clone pSW1H8]	ABE03935
KS_174	1229	RHG_cluster_130	68.447	412	0		Beta-ketoacyl-[acyl-carrier-protein] synthase II [Gemmatimonadetes bacterium]	RMH15744
KS_175	1217	RHG_cluster_114	69.136	405	0		3-Oxoacyl-[acyl-carrier-protein] synthase 2 [bacterium HR37]	GBD38054
KS_176	1265	RHG_cluster_335	72.01	418	0	SupA	SupA [Theonella swinhoei bacterial symbiont clone pSW1H8]	ABE03935
KS_178	1187	RHG_cluster_217	79.348	368	0	SupA	SupA [Theonella swinhoei bacterial symbiont clone pSW1H8]	ABE03935
KS_181	1223	RHG_cluster_188	64.532	406	0		Beta-ketoacyl-ACP synthase II [Moorella humiferrea]	WP_106006374
KS_182	1223	RHG_cluster_225	74.265	408	0		Beta-ketoacyl-[acyl-carrier-protein] synthase II [Chloroflexi bacterium]	TDI92680
KS_186	1280	RHG_cluster_078	64.858	424	4.20E-174		Hypothetical protein A3I72_03985 [Candidatus Tectomicrobia bacterium RIFCSPLOWO2_02_FULL_70_19]	OGL60875
KS_188	1217	RHG_cluster_009	70.968	403	0		Beta-ketoacyl-[acyl-carrier-protein] synthase II [Gammaproteobacteria bacterium]	TDJ36329
KS_189	1196	RHG_cluster_334	67.669	399	0		Beta-ketoacyl-[acyl-carrier-protein] synthase II [Gammaproteobacteria bacterium]	TDJ29040

KS_191	1226	RHG_cluster_142	62.162	407	0		Beta-ketoacyl-[acyl-carrier-protein] synthase II [Proteobacteria bacterium]	PZN30994
KS_193	1091	RHG_cluster_359	69.78	364	0		Beta-ketoacyl-[acyl-carrier-protein] synthase II [Candidatus Muproteobacteria bacterium RBG_19FT_COMBO_61_10]	OGI58435
KS_195	1163	RHG_cluster_122	68.325	382	0		Beta-ketoacyl-[acyl-carrier-protein] synthase II [Gammaproteobacteria bacterium]	TDJ41741
KS_197	1226	RHG_cluster_305	61.52	408	1.50E-179		Beta-ketoacyl synthase [Nitrosomonas europaea]	KXK43820
KS_202	1223	RHG_cluster_344	66.23	382	0		Beta-ketoacyl-[acyl-carrier-protein] synthase II [Candidatus Muproteobacteria bacterium RBG_19FT_COMBO_61_10]	OGI58435
KS_203	1217	RHG_cluster_235	69.704	406	0		Beta-ketoacyl-[acyl-carrier-protein] synthase II [Gammaproteobacteria bacterium RBG_16_51_14]	OGT30519
KS_205	1064	RHG_cluster_120	70.566	265	1.35E-131		3-Oxoacyl-ACP synthase [Ralstonia pickettii DTP0602]	AGW95093
KS_218	1136	RHG_cluster_080	44.764	382	6.50E-63		Hypothetical protein A3J27_09500 [Candidatus Tectomicrobia bacterium RIFCSPLOWO2_12_FULL_69_37]	OGL68338
KS_220	1223	RHG_cluster_274	73.037	382	0		Beta-ketoacyl-[acyl-carrier-protein] synthase II [SAR86 cluster bacterium]	PCJ26052
KS_223	1223	RHG_cluster_012	71.253	407	0		MULTISPECIES: Beta-ketoacyl-ACP synthase II [Ectothiorhodospira]	WP_025281708
KS_224	1259	RHG_cluster_296	79.426	418	0	SupA	SupA [Theonella swinhoei bacterial symbiont clone pSW1H8]	ABE03935
KS_225	1223	RHG_cluster_319	68.395	405	0		Beta-ketoacyl-[acyl-carrier-protein] synthase II [Acidobacteria bacterium]	RPH64142
KS_235	1217	RHG_cluster_324	66.584	404	0		Beta-ketoacyl-[acyl-carrier-protein] synthase II [Thermodesulfobacteriales bacterium]	TFG75955
KS_236	1223	RHG_cluster_347	71.429	406	0		Beta-ketoacyl-[acyl-carrier-protein] synthase II [OM182 bacterium MED-G28]	PDH35184
KS_237	1223	RHG_cluster_281	76.961	408	0		Beta-ketoacyl-[acyl-carrier-protein] synthase II [Acidobacteria bacterium RIFCSPLOWO2_02_FULL_67_36]	OFW09771
KS_240	1157	RHG_cluster_051	62.565	382	8.48E-158		Beta-ketoacyl synthase [Actinobacteria bacterium]	RUA23523
KS_244	1220	RHG_cluster_263	70.443	406	0		Beta-ketoacyl-[acyl-carrier-protein] synthase II [Gammaproteobacteria bacterium RBG_16_51_14]	OGT30519
KS_249	1226	RHG_cluster_220	62.162	407	0		Beta-ketoacyl-[acyl-carrier-protein] synthase II [Proteobacteria bacterium]	PZN30994
KS_252	1292	RHG_cluster_011	78.788	429	0	SwfA	Type I polyketide synthase [bacterium symbiont of Plakortis simplex pPSA11D7]	AGH13590
KS_254	1226	RHG_cluster_301	62.347	409	0		Beta-ketoacyl-[acyl-carrier-protein] synthase II [Candidatus Handelsmanbacteria bacterium RIFCSPLOWO2_12_FULL_64_10]	OGG49603
KS_256	1229	RHG_cluster_039	65.366	410	0		Beta-ketoacyl-[acyl-carrier-protein] synthase II [Gemmatimonadetes bacterium]	RMH15744
KS_259	1220	RHG_cluster_148	64.02	403	1.48E-166		Beta-ketoacyl-[acyl-carrier-protein] synthase family protein [Acidobacteria bacterium]	REJ81214
KS_261	1223	RHG_cluster_035	73.399	406	0		Beta-ketoacyl-[acyl-carrier-protein] synthase II [Gammaproteobacteria bacterium TMED119]	OUV63604

KS_264	1214	RHG_cluster_227	69.059	404	0		Beta-ketoacyl-[acyl-carrier-protein] synthase II [Acidobacteria bacterium]	RMH20421
KS_267	1292	RHG_cluster_028	78.322	429	0	SwfA	Type I polyketide synthase [bacterium symbiont of Plakortis simplex pPSA11D7]	AGH13590
KS_269	1151	RHG_cluster_212	78.272	382	0		Beta-ketoacyl synthase [Acidimicrobiales bacterium MED-G01]	PDH69975
KS_270	1229	RHG_cluster_185	62.347	409	0		Beta-ketoacyl-ACP synthase II [Nitrosomonas aestuarii]	WP_090702733
KS_278	1226	RHG_cluster_020	71.144	402	0		Beta-ketoacyl-[acyl-carrier-protein] synthase II [Candidatus Handelsmanbacteria bacterium RIFCSPLOWO2_12_FULL_64_10]	OGG49603
KS_285	1280	RHG_cluster_081	72.104	423	0		Hypothetical protein A3I72_04025 [Candidatus Tectomicrobia bacterium RIFCSPLOWO2_02_FULL_70_19]	OGL60883
KS_290	1292	RHG_cluster_091	83.411	428	0	SwfA	Type I polyketide synthase [bacterium symbiont of Plakortis simplex pPSA11D7]	AGH13590
KS_292	1292	RHG_cluster_096	69.07	430	0	SwfA	Type I polyketide synthase [bacterium symbiont of Plakortis simplex pPSA11D7]	AGH13590
KS_295	1286	RHG_cluster_094	64.371	421	0		SDR family NAD(P)-dependent oxidoreductase [Cocleimonas flava]	WP_131906443
KS_296	1229	RHG_cluster_244	63.882	407	0		Beta-ketoacyl-[acyl-carrier-protein] synthase II [Actinobacteria bacterium RBG_16_68_21]	OFW66625
KS_298	1259	RHG_cluster_253	71.599	419	0		Beta-ketoacyl-[acyl-carrier-protein] synthase family protein [Acidobacteria bacterium]	TDI25416
KS_299	1160	RHG_cluster_273	70.893	347	1.48E-168		Beta-ketoacyl synthase [Actinobacteria bacterium]	RUA23523
KS_300	1226	RHG_cluster_075	71.499	407	0		Beta-ketoacyl-[acyl-carrier-protein] synthase II [bacterium]	TAK06915
KS_304	1154	RHG_cluster_045	53.786	383	3.88E-131		Hypothetical protein [Pelagimonas phthalicica]	WP_099241578
KS_313	1226	RHG_cluster_178	72.482	407	0		Beta-ketoacyl-[acyl-carrier-protein] synthase II [bacterium]	TAK06915
KS_316	1217	RHG_cluster_264	66.832	404	0		Beta-ketoacyl-[acyl-carrier-protein] synthase II [Chloroflexi bacterium]	RME76096
KS_318	1214	RHG_cluster_030	67.822	404	0		Beta-ketoacyl-[acyl-carrier-protein] synthase II [Chloroflexi bacterium]	RME76096
KS_319	1160	RHG_cluster_259	70.317	347	3.44E-166		Beta-ketoacyl synthase [Actinobacteria bacterium]	RUA23523
KS_320	1286	RHG_cluster_215	80.328	427	0		Putative polyketide synthase [uncultured bacterium 2063G]	ABB73286
KS_324	1223	RHG_cluster_313	66.585	407	0		Beta-ketoacyl-[acyl-carrier-protein] synthase II [Chloroflexi bacterium]	TDI92680
KS_328	1154	RHG_cluster_133	51.558	353	8.38E-110		Hypothetical protein [Pelagimonas phthalicica]	WP_099241578
KS_331	1160	RHG_cluster_085	69.713	383	0		Beta-ketoacyl synthase [Actinobacteria bacterium]	RUA23523
KS_333	1286	RHG_cluster_165	80.328	427	0		Putative polyketide synthase [uncultured bacterium 2063G]	ABB73286
KS_335	1298	RHG_cluster_302	78.089	429	0	SwfA	Type I polyketide synthase [bacterium symbiont of Plakortis simplex pPSA11D7]	AGH13590
KS_343	1229	RHG_cluster_222	67.64	411	0		Beta-ketoacyl-[acyl-carrier-protein] synthase II [Gemmatimonadetes bacterium]	RMH15744

KS_344	1292	RHG_cluster_016	75.991	429	0	SwfA	Type I polyketide synthase [bacterium symbiont of Plakortis simplex pPSA11D7]	AGH13590
KS_347	1292	RHG_cluster_034	61.593	427	1.30E-174	SwfA	Type I polyketide synthase [bacterium symbiont of Plakortis simplex pPSA11D7]	AGH13590
KS_348	1226	RHG_cluster_348	70.098	408	0		Beta-ketoacyl-[acyl-carrier-protein] synthase II [Chloroflexi bacterium]	RLT28345
KS_352	1229	RHG_cluster_004	67.64	411	0		Beta-ketoacyl-[acyl-carrier-protein] synthase II [Gemmatimonadetes bacterium]	RMH15744
KS_355	1154	RHG_cluster_236	59.791	383	1.03E-161		Beta-ketoacyl synthase [Acidimicrobiaceae bacterium TMED77]	OUV00831
KS_359	1292	RHG_cluster_206	75.878	427	0	SwfA	Type I polyketide synthase [bacterium symbiont of Plakortis simplex pPSA11D7]	AGH13590
KS_365	1220	RHG_cluster_031	76.485	404	0		Beta-ketoacyl-[acyl-carrier-protein] synthase II [Nitrospirae bacterium]	TAJ27615
KS_367	1229	RHG_cluster_084	67.397	411	0		Beta-ketoacyl-[acyl-carrier-protein] synthase II [Gemmatimonadetes bacterium]	RMH15744
KS_374	1184	RHG_cluster_360	60.66	394	8.82E-179		Beta-ketoacyl-[acyl-carrier-protein] synthase II [Chloroflexi bacterium TMED70]	OUU77203
KS_377	1229	RHG_cluster_150	61.52	408	0		Beta-ketoacyl-[acyl-carrier-protein] synthase II [Chloroflexi bacterium TMED230]	OUW96072
KS_380	1292	RHG_cluster_135	77.622	429	0	SwfA	Type I polyketide synthase [bacterium symbiont of Plakortis simplex pPSA11D7]	AGH13590
KS_387	1292	RHG_cluster_067	75.058	429	0	SwfA	Type I polyketide synthase [bacterium symbiont of Plakortis simplex pPSA11D7]	AGH13590
KS_388	1229	RHG_cluster_317	68.049	410	0		Beta-ketoacyl-[acyl-carrier-protein] synthase II [Gemmatimonadetes bacterium]	RMH15744
KS_390	1223	RHG_cluster_172	64.532	406	0		Beta-ketoacyl-[acyl-carrier-protein] synthase II [Candidatus Handelsmanbacteria bacterium RIFCSPLOWO2_12_FULL_64_10]	OGG49603
KS_392	1247	RHG_cluster_097	71.981	414	0		Beta-ketoacyl-ACP synthase II [Rhodospirillaceae bacterium SYSU D60014]	WP_119459592
KS_396	1292	RHG_cluster_269	72.028	429	0	SwfA	Type I polyketide synthase [bacterium symbiont of Plakortis simplex pPSA11D7]	AGH13590
KS_397	1292	RHG_cluster_202	88.095	420	0	SwfA	Type I polyketide synthase [bacterium symbiont of Plakortis simplex pPS11G3]	AGH13577
KS_398	1286	RHG_cluster_167	81.733	427	0		Putative polyketide synthase [uncultured bacterium 2063G]	ABB73286
KS_400	1184	RHG_cluster_132	62.5	392	8.30E-169		Beta-ketoacyl-[acyl-carrier-protein] synthase family protein [Pelagimonas phthalicica]	WP_099241574
KS_406	1184	RHG_cluster_044	63.265	392	9.60E-171		Beta-ketoacyl-[acyl-carrier-protein] synthase family protein [Pelagimonas phthalicica]	WP_099241574
KS_408	1292	RHG_cluster_128	91.905	420	0	SwfA	Type I polyketide synthase [bacterium symbiont of Plakortis simplex pPS11G3]	AGH13577
KS_410	1286	RHG_cluster_037	91.569	427	0		Putative polyketide synthase [uncultured bacterium 2063G]	ABB73286
KS_411	1223	RHG_cluster_214	58.333	408	4.30E-179		Beta-ketoacyl-[acyl-carrier-protein] synthase II [Chloroflexi bacterium RBG_16_64_32]	OGO48822

KS_429	1292	RHG_cluster_040	66.667	426	0	SupA	Polyketide synthase, partial [Aplysina aerophoba bacterial symbiont clone pAE27P20]	ABE03895
KS_430	1292	RHG_cluster_109	76.69	429	0	SwfA	Type I polyketide synthase [bacterium symbiont of Plakortis simplex pPSA11D7]	AGH13590
KS_437	1235	RHG_cluster_077	68.856	411	0		Beta-ketoacyl-[acyl-carrier-protein] synthase II [Gemmatimonadetes bacterium]	RMH15744
KS_439	1211	RHG_cluster_086	76.19	399	0		Beta-ketoacyl-ACP synthase II [Rhodospirillaceae bacterium SYSU D60014]	WP_119459592
KS_446	1289	RHG_cluster_069	86.651	427	0	SupA	SupA [Theonella swinhoei bacterial symbiont clone pSW1H8]	ABE03935
KS_448	1292	RHG_cluster_209	88.095	420	0	SwfA	Type I polyketide synthase [bacterium symbiont of Plakortis simplex pPS11G3]	AGH13577
KS_449	1160	RHG_cluster_241	69.171	386	0		Beta-ketoacyl-ACP synthase I [Gammaproteobacteria bacterium]	RLA03002
KS_452	1286	RHG_cluster_249	75.878	427	0	SupA	SupA [Theonella swinhoei bacterial symbiont clone pSW1H8]	ABE03935
KS_453	1286	RHG_cluster_174	67.529	425	0	SupA	SupA [Aplysina aerophoba bacterial symbiont clone pAPKS18]	ABE03915
KS_459	1184	RHG_cluster_218	72.237	389	0		3-Oxoacyl-ACP synthase [Candidatus Tenderia electrophaga]	ALP54806
KS_460	1145	RHG_cluster_074	56.614	378	1.02E-132		Beta-ketoacyl-[acyl-carrier-protein] synthase family protein [Lysobacter sp.]	TBR14181
KS_462	1190	RHG_cluster_143	69.424	399	0		Beta-ketoacyl-ACP synthase II [Pararhodobacter aggregans]	WP_107749575
KS_468	1175	RHG_cluster_068	81.538	390	0		Beta-ketoacyl-ACP synthase II [Thioalkalivibrio sp. HK1]	WP_038098656
KS_469	1286	RHG_cluster_100	83.138	427	0		Putative polyketide synthase [uncultured bacterium 2063G]	ABB73286
KS_471	1175	RHG_cluster_144	80	390	0		Beta-ketoacyl-ACP synthase II [Thioalkalivibrio sp. HK1]	WP_038098656
KS_474	1211	RHG_cluster_216	73.869	398	0		Beta-ketoacyl-ACP synthase II [Telmatospirillum siberiense]	WP_101252614
KS_476	1133	RHG_cluster_061	61.047	344	2.96E-146		Beta-ketoacyl-ACP synthase I [Deltaproteobacteria bacterium]	RLB53742
KS_481	1154	RHG_cluster_303	72.063	383	0		Beta-ketoacyl-ACP synthase I [Thermopetrobacter sp. TC1]	WP_038035251
KS_484	1298	RHG_cluster_298	64.419	430	8.68E-176	SupA	SupA [Theonella swinhoei bacterial symbiont clone pSW1H8]	ABE03935
KS_486	1112	RHG_cluster_059	74.857	350	0		Beta-ketoacyl synthase [Acidimicrobiales bacterium MED-G01]	PDH69975
KS_490	1292	RHG_cluster_213	70	430	0	SwfA	Type I polyketide synthase [bacterium symbiont of Plakortis simplex pPSA11D7]	AGH13590
KS_491	1286	RHG_cluster_154	81.499	427	0	SupA	SupA [Theonella swinhoei bacterial symbiont clone pSW1H8]	ABE03935
KS_494	1292	RHG_cluster_072	77.622	429	0	SwfA	Type I polyketide synthase [bacterium symbiont of Plakortis simplex pPSA11D7]	AGH13590
KS_495	1292	RHG_cluster_272	76.224	429	0	SwfA	Type I polyketide synthase [bacterium symbiont of Plakortis simplex pPSA11D7]	AGH13590
KS_497	1286	RHG_cluster_163	78.454	427	0	SupA	SupA [Theonella swinhoei bacterial symbiont clone pSW1H8]	ABE03935
KS_498	1286	RHG_cluster_322	82.201	427	0		Putative polyketide synthase [uncultured bacterium 2063G]	ABB73286
KS_499	1094	RHG_cluster_265	73.973	365	0		Beta-ketoacyl-ACP synthase II [Rhodothermus marinus]	WP_014068140
KS_501	1286	RHG_cluster_204	87.119	427	0	SupA	SupA [Theonella swinhoei bacterial symbiont clone pSW1H8]	ABE03935

KS_506	1286	RHG_cluster_179	90.376	426	0	SupA	SupA [Aplysina aerophoba bacterial symbiont clone pAPKS18]	ABE03915
KS_507	1286	RHG_cluster_057	79.859	427	0	SupA	SupA [Theonella swinhoei bacterial symbiont clone pSW1H8]	ABE03935
KS_511	1292	RHG_cluster_090	85.948	427	0		Putative polyketide synthase [uncultured bacterium 2063G]	ABB73286
KS_516	1292	RHG_cluster_162	87.822	427	0	SupA	SupA [Theonella swinhoei bacterial symbiont clone pSW1H8]	ABE03935
KS_517	1220	RHG_cluster_155	63.027	403	0		Beta-ketoacyl-ACP synthase II [Albimonas donghaensis]	WP_092679786
KS_519	1184	RHG_cluster_065	72.843	394	0		Beta-ketoacyl-[acyl-carrier-protein] synthase II [Candidatus Tectomicrobia bacterium RIFCSPLOWO2_12_FULL_69_37]	OGL61959
KS_520	1286	RHG_cluster_195	83.372	427	0	SupA	SupA [Theonella swinhoei bacterial symbiont clone pSW1H8]	ABE03935
KS_525	1286	RHG_cluster_192	81.03	427	0		Putative polyketide synthase [uncultured bacterium 2063G]	ABB73286
KS_526	1148	RHG_cluster_103	69.895	382	0		Beta-ketoacyl-[acyl-carrier-protein] synthase II [Gammaproteobacteria bacterium RBG_16_51_14]	OGT30519
KS_529	1292	RHG_cluster_194	82.904	427	0	SupA	SupA [Theonella swinhoei bacterial symbiont clone pSW1H8]	ABE03935
KS_532	1286	RHG_cluster_266	80.751	426	0	SupA	SupA [Aplysina aerophoba bacterial symbiont clone pAPKS18]	ABE03915
KS_538	1286	RHG_cluster_160	65.728	426	0	SupA	SupA [Aplysina aerophoba bacterial symbiont clone pAPKS18]	ABE03915
KS_541	1286	RHG_cluster_027	80.094	427	0	SupA	SupA [Theonella swinhoei bacterial symbiont clone pSW1H8]	ABE03935
KS_543	1286	RHG_cluster_054	76.581	427	0	SupA	SupA [Theonella swinhoei bacterial symbiont clone pSW1H8]	ABE03935
KS_545	1292	RHG_cluster_242	79.625	427	0	SupA	SupA [Theonella swinhoei bacterial symbiont clone pSW1H8]	ABE03935
KS_548	1277	RHG_cluster_159	74.533	428	0		SA1_PKSA [uncultured bacterial symbiont of Discodermia dissoluta]	AAY00025
KS_551	1292	RHG_cluster_147	86.651	427	0	SupA	SupA [Theonella swinhoei bacterial symbiont clone pSW1H8]	ABE03935
KS_553	1250	RHG_cluster_053	70.29	414	1.36E-178		SA1_PKSA [uncultured bacterial symbiont of Discodermia dissoluta]	AAY00025
KS_554	1283	RHG_cluster_008	87.324	426	0	SupA	SupA [Aplysina aerophoba bacterial symbiont clone pAPKS18]	ABE03915
KS_555	1286	RHG_cluster_177	82.436	427	0	SupA	SupA [Theonella swinhoei bacterial symbiont clone pSW1H8]	ABE03935
KS_559	1301	RHG_cluster_285	75	428	0	SupA	SupA [Aplysina aerophoba bacterial symbiont clone pAPKS18]	ABE03915
KS_569	1292	RHG_cluster_238	84.778	427	0	SupA	SupA [Theonella swinhoei bacterial symbiont clone pSW1H8]	ABE03935
KS_570	1073	RHG_cluster_095	54.294	326	9.24E-117		Beta-ketoacyl-[acyl-carrier-protein] synthase family protein [Rhodospirillaceae bacterium SYSU D60007]	WP_119262600
KS_572	1286	RHG_cluster_288	83.411	428	0	SupA	SupA [Theonella swinhoei bacterial symbiont clone pSW1H8]	ABE03935
KS_573	1292	RHG_cluster_056	86.651	427	0		Putative polyketide synthase [uncultured bacterium 2063G]	ABB73286
KS_575	1067	RHG_cluster_232	56.376	298	1.05E-118		Beta-ketoacyl-ACP synthase [Alteromonas sp. RKMC-009]	WP_119502388
KS_577	1178	RHG_cluster_046	61.675	394	3.52E-156		Beta-ketoacyl-[acyl-carrier-protein] synthase family protein [Verminephrobacter aporrectodeae]	WP_081479930
KS_581	1010	RHG_cluster_121	55.67	194	3.63E-63		Beta-ketoacyl-ACP synthase [Xanthomonadaceae bacterium]	RYD16404
KS_583	1184	RHG_cluster_134	59.836	366	3.15E-136		Beta-ketoacyl-[acyl-carrier-protein] synthase family protein [Verminephrobacter aporrectodeae]	WP_081479930

KS_588	1016	RHG_cluster_125	66.803	244	2.53E-108		Beta-ketoacyl-[acyl-carrier-protein] synthase family protein [Parasulfuritortus cantonensis]	WP_131445552
KS_593	1283	RHG_cluster_306	74.941	423	0		SA1_PKSA [uncultured bacterial symbiont of Discodermia dissoluta]	AAY00025
KS_596	1259	RHG_cluster_261	63.309	417	5.76E-172	SupA	SupA [Theonella swinhoei bacterial symbiont clone pSW1H8]	ABE03935
KS_599	1079	RHG_cluster_182	65.138	327	7.89E-143		Beta-ketoacyl synthase [Gammaproteobacteria bacterium]	TDJ41416
KS_603	851	RHG_cluster_270	73.469	245	1.73E-122		Beta-ketoacyl synthase [Acidobacteria bacterium]	PYQ67687
KS_614	1259	RHG_cluster_262	75.54	417	0	SupA	SupA [Aplysina aerophoba bacterial symbiont clone pAPKS18]	ABE03915
KS_621	1259	RHG_cluster_208	80.336	417	0		Putative polyketide synthase [uncultured bacterium 2063G]	ABB73286
KS_625	1262	RHG_cluster_289	70.167	419	0	SupA	SupA [Theonella swinhoei bacterial symbiont clone pSW1H8]	ABE03935
KS_627	1265	RHG_cluster_162	84.928	418	0	SupA	SupA [Theonella swinhoei bacterial symbiont clone pSW1H8]	ABE03935
KS_628	1277	RHG_cluster_248	66.667	423	1.04E-176	SupA	SupA [Aplysina aerophoba bacterial symbiont clone pAPKS18]	ABE03915
KS_636	1262	RHG_cluster_119	76.123	423	0		SA1_PKSA [uncultured bacterial symbiont of Discodermia dissoluta]	AAY00025
KS_638	1259	RHG_cluster_226	80.144	418	0	SupA	SupA [Theonella swinhoei bacterial symbiont clone pSW1H8]	ABE03935
KS_642	713	RHG_cluster_140	79.149	235	3.70E-116		SA1_PKSA [uncultured bacterial symbiont of Discodermia dissoluta]	AAY00025
KS_643	1256	RHG_cluster_233	75.352	426	0		SA1_PKSA [uncultured bacterial symbiont of Discodermia dissoluta]	AAY00025
KS_647	1256	RHG_cluster_229	75.12	418	0	SupA	SupA [Theonella swinhoei bacterial symbiont clone pSW1H8]	ABE03935
KS_650	1235	RHG_cluster_191	72.705	414	0		SA1_PKSA [uncultured bacterial symbiont of Discodermia dissoluta]	AAY00025
KS_652	1259	RHG_cluster_268	68.345	417	0	SupA	SupA [Aplysina aerophoba bacterial symbiont clone pAPKS18]	ABE03915
KS_655	1244	RHG_cluster_203	66.118	425	2.01E-175		SA1_PKSA [uncultured bacterial symbiont of Discodermia dissoluta]	AAY00025
KS_657	1259	RHG_cluster_140	75.65	423	0		SA1_PKSA [uncultured bacterial symbiont of Discodermia dissoluta]	AAY00025
KS_660	1259	RHG_cluster_197	71.531	418	0	SupA	SupA [Theonella swinhoei bacterial symbiont clone pSW1H8]	ABE03935
KS_662	1265	RHG_cluster_062	63.397	418	7.15E-173	SupA	SupA [Theonella swinhoei bacterial symbiont clone pSW1H8]	ABE03935
KS_664	1277	RHG_cluster_145	67.376	423	0		SA1_PKSA [uncultured bacterial symbiont of Discodermia dissoluta]	AAY00025
KS_666	1265	RHG_cluster_246	80.336	417	0		Putative polyketide synthase [uncultured bacterium 2063G]	ABB73286
KS_668	1259	RHG_cluster_240	71.905	420	0	SupA	SupA [Theonella swinhoei bacterial symbiont clone pSW1H8]	ABE03935
KS_670	1259	RHG_cluster_014	68.106	417	0	SupA	SupA [Aplysina aerophoba bacterial symbiont clone pAPKS18]	ABE03915
KS_672	1262	RHG_cluster_198	67.703	418	0	SupA	SupA [Theonella swinhoei bacterial symbiont clone pSW1H8]	ABE03935
KS_673	1268	RHG_cluster_119	74.292	424	0		SA1_PKSA [uncultured bacterial symbiont of Discodermia dissoluta]	AAY00025
KS_677	1259	RHG_cluster_149	69.856	418	0	SupA	SupA [Theonella swinhoei bacterial symbiont clone pSW1H8]	ABE03935

KS_679	1253	RHG_cluster_053	58.554	415	6.49E-145		SA1_PKSA [uncultured bacterial symbiont of Discodermia dissoluta]	AAY00025
KS_682	1277	RHG_cluster_140	73.995	423	0		SA1_PKSA [uncultured bacterial symbiont of Discodermia dissoluta]	AAY00025
KS_685	1268	RHG_cluster_140	74.704	423	0		SA1_PKSA [uncultured bacterial symbiont of Discodermia dissoluta]	AAY00025
KS_689	1259	RHG_cluster_161	68.106	417	0	SupA	SupA [Aplysina aerophoba bacterial symbiont clone pAPKS18]	ABE03915
KS_691	1259	RHG_cluster_164	83.213	417	0	SupA	SupA [Aplysina aerophoba bacterial symbiont clone pAPKS18]	ABE03915
KS_692	1259	RHG_cluster_124	76.555	418	0	SupA	SupA [Theonella swinhoei bacterial symbiont clone pSW1H8]	ABE03935
KS_693	1259	RHG_cluster_037	94.724	417	0		Putative polyketide synthase [uncultured bacterium 2063G]	ABB73286
KS_697	1265	RHG_cluster_165	80.096	417	0		Putative polyketide synthase [uncultured bacterium 2063G]	ABB73286
KS_700	1271	RHG_cluster_019	74.232	423	0		SA1_PKSA [uncultured bacterial symbiont of Discodermia dissoluta]	AAY00025
KS_704	1262	RHG_cluster_154	72.01	418	0	SupA	SupA [Theonella swinhoei bacterial symbiont clone pSW1H8]	ABE03935
KS_707	1262	RHG_cluster_069	82.297	418	0	SupA	SupA [Theonella swinhoei bacterial symbiont clone pSW1H8]	ABE03935
KS_712	1259	RHG_cluster_100	80.815	417	0		Putative polyketide synthase [uncultured bacterium 2063G]	ABB73286
KS_714	1256	RHG_cluster_040	61.631	417	5.15E-171	SupA	SupA [uncultured marine bacterium 1k6]	ACX49735
KS_715	1259	RHG_cluster_090	68.9	418	0		SupA [Theonella swinhoei bacterial symbiont clone pSW1H8]	ABE03935
KS_719	1259	RHG_cluster_056	70.743	417	0		SupA [Aplysina aerophoba bacterial symbiont clone pAPKS18]	ABE03915
KS_723	1256	RHG_cluster_054	66.905	420	0	SupA	SupA [Theonella swinhoei bacterial symbiont clone pSW1H8]	ABE03935
KS_724	1259	RHG_cluster_057	73.684	418	0	SupA	SupA [Theonella swinhoei bacterial symbiont clone pSW1H8]	ABE03935
KS_727	1259	RHG_cluster_179	87.05	417	0	SupA	SupA [Aplysina aerophoba bacterial symbiont clone pAPKS18]	ABE03915
KS_729	1265	RHG_cluster_027	68.9	418	0	SupA	SupA [Theonella swinhoei bacterial symbiont clone pSW1H8]	ABE03935
KS_733	1262	RHG_cluster_147	74.463	419	0	SupA	Polyketide synthase, partial [Aplysina aerophoba bacterial symbiont clone pAE27P20]	ABE03895
KS_735	1124	RHG_cluster_194	70.588	374	2.24E-170	SupA	SupA [Aplysina aerophoba bacterial symbiont clone pAPKS18]	ABE03915
KS_738	1277	RHG_cluster_159	76.359	423	0		SA1_PKSA [uncultured bacterial symbiont of Discodermia dissoluta]	AAY00025
KS_741	1259	RHG_cluster_008	75.3	417	0	SupA	SupA [Aplysina aerophoba bacterial symbiont clone pAPKS18]	ABE03915
KS_744	1262	RHG_cluster_087	66.986	418	0	SupA	SupA [Theonella swinhoei bacterial symbiont clone pSW1H8]	ABE03935
KS_746	1274	RHG_cluster_177	68.779	426	0	SupA	SupA [Theonella swinhoei bacterial symbiont clone pSW1H8]	ABE03935
KS_754	1262	RHG_cluster_193	75.887	423	0		SA1_PKSA [uncultured bacterial symbiont of Discodermia dissoluta]	AAY00025
KS_758	1250	RHG_cluster_119	72.471	425	0		SA1_PKSA [uncultured bacterial symbiont of Discodermia dissoluta]	AAY00025
KS_759	1268	RHG_cluster_019	74.468	423	0		SA1_PKSA [uncultured bacterial symbiont of Discodermia dissoluta]	AAY00025

Table S10. BLAST search of the KS sequences from the metagenome of *Spheciospongia* sp. against the nr database in NCBI

KS ID	Length (nt)	BGC ID	Identity (%)	Alignment length (aa)	E-value	Sponge-specific cluster	The homologenus sequences	Accession number
KS_002	1086	SPV_cluster_403	81.844	358	0		Beta-ketoacyl-ACP synthase II [Thioalkalivibrio sp. HK1]	WP_038098656
KS_003	459	SPV_cluster_447	84.667	150	7.00E-77	SupA	SupA [Aplysina aerophoba bacterial symbiont clone pAPKS18]	ABE03915
KS_005	1266	SPV_cluster_194	74.762	420	0	SwfA	Type I polyketide synthase [bacterium symbiont of Plakortis simplex pPSA11D7]	AGH13590
KS_008	1266	SPV_cluster_115	80.523	421	0	SupA	SupA [uncultured marine bacterium 1k6]	ACX49735
KS_009	663	SPV_cluster_191	68.9	209	2.71E-92		SA1_PKSA [uncultured bacterial symbiont of Discodermia dissoluta]	AAY00025
KS_015	435	SPV_cluster_422	84.615	143	3.46E-73		Putative polyketide synthase [uncultured bacterium 2063G]	ABB73286
KS_017	696	SPV_cluster_454	81.498	227	8.97E-112	SwfA	Type I polyketide synthase [bacterium symbiont of Plakortis simplex pPS11G3]	AGH13577
KS_018	1266	SPV_cluster_241	80.523	421	0	SupA	SupA [uncultured marine bacterium 1k6]	ACX49735
KS_022	783	SPV_cluster_440	71.318	258	7.56E-114	SupA	Polyketide synthase, partial [Aplysina aerophoba bacterial symbiont clone pAE27P20]	ABE03895
KS_023	297	SPV_cluster_287	63.542	96	1.37E-32	SupA	SupA [Theonella swinhoei bacterial symbiont clone pSW1H8]	ABE03935
KS_024	945	SPV_cluster_405	87.762	286	1.14E-162		Putative polyketide synthase [uncultured bacterium 2063G]	ABB73286
KS_025	1221	SPV_cluster_202	70.324	401	0	SupA	SupA [Theonella swinhoei bacterial symbiont clone pSW1H8]	ABE03935
KS_027	351	SPV_cluster_456	77.193	114	1.29E-50	SupA	SupA [Theonella swinhoei bacterial symbiont clone pSW1H8]	ABE03935
KS_031	744	SPV_cluster_302	69.919	246	9.68E-108	SupA	SupA [Theonella swinhoei bacterial symbiont clone pSW1H8]	ABE03935
KS_034	1077	SPV_cluster_360	68.451	355	6.99E-155		SA1_PKSA [uncultured bacterial symbiont of Discodermia dissoluta]	AAY00025
KS_035	1014	SPV_cluster_461	68.249	337	3.54E-167		Beta-ketoacyl-[acyl-carrier-protein] synthase II [Gammaproteobacteria bacterium TMED30]	OUU04838
KS_038	1113	SPV_cluster_096	82.88	368	0	SupA	SupA [Aplysina aerophoba bacterial symbiont clone pAPKS18]	ABE03915
KS_042	669	SPV_cluster_382	79.524	210	4.05E-107	SupA	SupA [Aplysina aerophoba bacterial symbiont clone pAPKS18]	ABE03915
KS_047	1113	SPV_cluster_418	66.209	364	1.80E-158	SupA	SupA [Theonella swinhoei bacterial symbiont clone pSW1H8]	ABE03935
KS_049	438	SPV_cluster_450	69.231	143	2.14E-58	SupA	SupA [Aplysina aerophoba bacterial symbiont clone pAPKS18]	ABE03915
KS_050	279	SPV_cluster_446	68.889	90	5.62E-35	SupA	SupA [uncultured marine bacterium 1k6]	ACX49735
KS_051	531	SPV_cluster_451	71.345	171	1.02E-73	SupA	SupA [Aplysina aerophoba bacterial symbiont clone pAPKS18]	ABE03915
KS_055	312	SPV_cluster_391	93.137	102	3.31E-57	SwfA	Type I polyketide synthase [bacterium symbiont of Plakortis simplex pPS11G3]	AGH13577
KS_057	849	SPV_cluster_378	80.727	275	1.51E-143		Putative polyketide synthase [uncultured bacterium 2063G]	ABB73286
KS_058	954	SPV_cluster_384	79.43	316	4.34E-162	SupA	SupA [Theonella swinhoei bacterial symbiont clone pSW1H8]	ABE03935

KS_059	570	SPV_cluster_455	84.409	186	2.30E-95	SupA	SupA [Theonella swinhoei bacterial symbiont clone pSW1H8]	ABE03935
KS_068	888	SPV_cluster_333	83.154	279	4.69E-149	SupA	SupA [Theonella swinhoei bacterial symbiont clone pSW1H8]	ABE03935
KS_070	1023	SPV_cluster_408	68.373	332	5.49E-170		Beta-ketoacyl synthase [marine gamma proteobacterium HTCC2148]	WP_007229230
KS_072	912	SPV_cluster_428	89.825	285	2.82E-165	SupA	SupA [Aplysina aerophoba bacterial symbiont clone pAPKS18]	ABE03915
KS_076	713	SPV_cluster_204	79.638	221	1.43E-110	SwfA	Type I polyketide synthase [bacterium symbiont of Plakortis simplex pPS11G3]	AGH13577
KS_081	1118	SPV_cluster_373	68.633	373	1.14E-166	SupA	SupA [Theonella swinhoei bacterial symbiont clone pSW1H8]	ABE03935
KS_082	1166	SPV_cluster_273	75.758	363	0	SupA	SupA [Theonella swinhoei bacterial symbiont clone pSW1H8]	ABE03935
KS_085	1112	SPV_cluster_396	79.076	368	0	SupA	SupA [Theonella swinhoei bacterial symbiont clone pSW1H8]	ABE03935
KS_086	671	SPV_cluster_323	85.047	214	1.38E-116	SupA	SupA [Theonella swinhoei bacterial symbiont clone pSW1H8]	ABE03935
KS_087	1097	SPV_cluster_425	83.516	364	0	SupA	SupA [Theonella swinhoei bacterial symbiont clone pSW1H8]	ABE03935
KS_089	929	SPV_cluster_347	89.123	285	1.89E-163	SupA	SupA [Aplysina aerophoba bacterial symbiont clone pAPKS18]	ABE03915
KS_090	398	SPV_cluster_386	80	130	2.33E-62	SwfA	Type I polyketide synthase [bacterium symbiont of Plakortis simplex pPSA11D7]	AGH13590
KS_092	1133	SPV_cluster_423	98.143	377	0		Beta-ketoacyl-[acyl-carrier-protein] synthase II [Candidatus Poribacteria bacterium]	RKU17865
KS_093	1085	SPV_cluster_279	72.222	360	0		Beta-ketoacyl-[acyl-carrier-protein] synthase II [Acidobacteria bacterium]	PYV30312
KS_097	914	SPV_cluster_292	68.864	273	2.78E-120	SupA	SupA [Theonella swinhoei bacterial symbiont clone pSW1H8]	ABE03935
KS_099	1061	SPV_cluster_262	69.516	351	1.88E-180		Beta-ketoacyl-[acyl-carrier-protein] synthase II [Chloroflexi bacterium]	RLT43681
KS_100	1292	SPV_cluster_356	74.126	429	0	SwfA	Type I polyketide synthase [bacterium symbiont of Plakortis simplex pPSA11D7]	AGH13590
KS_114	1043	SPV_cluster_318	74.203	345	0		Beta-ketoacyl-ACP synthase I [Neptuniibacter pectenicola]	WP_067983865
KS_118	1223	SPV_cluster_209	93.857	407	0		Beta-ketoacyl-[acyl-carrier-protein] synthase II [Candidatus Poribacteria bacterium]	RKU31990
KS_119	1199	SPV_cluster_043	67.506	397	0		Beta-ketoacyl-[acyl-carrier-protein] synthase family protein [Halocynthiibacter namhaensis]	WP_039018003
KS_121	1223	SPV_cluster_417	86.765	408	0		Beta-ketoacyl-[acyl-carrier-protein] synthase II [Candidatus Poribacteria bacterium]	RKU31990
KS_122	1184	SPV_cluster_401	75.949	395	0		Beta-ketoacyl-ACP synthase I [Methylarcula marina]	WP_106692425
KS_123	1223	SPV_cluster_099	75.862	406	0		Beta-ketoacyl-[acyl-carrier-protein] synthase II [Gammaproteobacteria bacterium]	TDJ17613
KS_124	1202	SPV_cluster_375	75.689	399	0		Beta-ketoacyl-[acyl-carrier-protein] synthase I [Gammaproteobacteria bacterium RIFCSPLOWO2_02_FULL_61_13]	OGT79717
KS_125	1250	SPV_cluster_160	73.366	413	0		Beta-ketoacyl-ACP synthase II [Rhodospirillales bacterium URHD0088]	WP_027300572
KS_127	1205	SPV_cluster_228	96.02	402	0		Beta-ketoacyl-ACP synthase I [Rhodospirillaceae bacterium	WP_108794197

							Spongia-Bin9]	
KS_128	1223	SPV_cluster_054	87.5	408	0		Beta-ketoacyl-[acyl-carrier-protein] synthase II [Candidatus Poribacteria bacterium]	RKU31990
KS_129	1223	SPV_cluster_033	94.363	408	0		Beta-ketoacyl-[acyl-carrier-protein] synthase II [Candidatus Poribacteria bacterium]	RKU30153
KS_132	1196	SPV_cluster_416	70.352	398	0		Beta-ketoacyl-[acyl-carrier-protein] synthase I [OM182 bacterium MED-G28]	PDH34065
KS_133	998	SPV_cluster_358	68.358	335	5.97E-169		Beta-ketoacyl-[acyl-carrier-protein] synthase II [Chloroflexi bacterium]	RIK44110
KS_136	1223	SPV_cluster_324	92.875	407	0		Beta-ketoacyl-[acyl-carrier-protein] synthase II [Candidatus Poribacteria bacterium]	RKU30153
KS_139	1202	SPV_cluster_293	73.116	398	0		3-Oxoacyl-ACP synthase [OM182 bacterium BACL3 MAG-120507-bin80]	KRO71573
KS_141	1211	SPV_cluster_230	78.5	400	0		Beta-ketoacyl-ACP synthase I [Paracoccus sphaerophysae]	WP_036717493
KS_145	1256	SPV_cluster_074	70.12	415	0		Beta-ketoacyl-ACP synthase II [alpha proteobacterium BAL199]	WP_040746409
KS_147	1208	SPV_cluster_155	78.412	403	0		3-Oxoacyl-[acyl-carrier-protein] synthase 1 [Alphaproteobacteria bacterium MarineAlpha3_Bin7]	PPR64109
KS_149	1223	SPV_cluster_076	81.773	406	0		Beta-ketoacyl-ACP synthase II [Thioalkalivibrio sp. HK1]	WP_038098656
KS_152	1226	SPV_cluster_251	58.824	408	0		Beta-ketoacyl-[acyl-carrier-protein] synthase II [Spirochaetes bacterium RBG_13_68_11]	OHD73861
KS_155	1058	SPV_cluster_438	73.295	352	0		Beta-ketoacyl-[acyl-carrier-protein] synthase I [Gammaproteobacteria bacterium RIFCSPLOWO2_02_FULL_61_13]	OGT79717
KS_156	1100	SPV_cluster_349	61.538	364	1.08E-160		Beta-ketoacyl-ACP synthase II [Spirochaeta lutea]	WP_037548043
KS_157	1223	SPV_cluster_032	81.818	407	0		Beta-ketoacyl-[acyl-carrier-protein] synthase II [bacterium]	RZP29941
KS_158	1220	SPV_cluster_233	66.501	403	5.66E-180		Beta-ketoacyl-[acyl-carrier-protein] synthase family protein [Acidobacteria bacterium]	REJ81214
KS_160	1286	SPV_cluster_245	80.986	426	0	SupA	SupA [Aplysina aerophoba bacterial symbiont clone pAPKS18]	ABE03915
KS_164	1223	SPV_cluster_329	72.059	408	0		Beta-ketoacyl-[acyl-carrier-protein] synthase II [Acidobacteria bacterium RIFCSPLOWO2_02_FULL_67_21]	OFW15863
KS_165	1223	SPV_cluster_039	94.103	407	0		Beta-ketoacyl-[acyl-carrier-protein] synthase II [Candidatus Poribacteria bacterium]	RKU10113
KS_168	1223	SPV_cluster_002	64.619	407	0		3-Oxoacyl-ACP synthase [candidate division Zixibacteria bacterium SM23_81]	KPL19508
KS_169	1253	SPV_cluster_413	71.053	418	0	SupA	SupA [Theonella swinhoei bacterial symbiont clone pSW1H8]	ABE03935
KS_171	1223	SPV_cluster_235	74.201	407	0		Beta-ketoacyl-[acyl-carrier-protein] synthase II [Acidobacteria bacterium]	TDI09580
KS_172	1229	SPV_cluster_014	68.689	412	0		Beta-ketoacyl-[acyl-carrier-protein] synthase II [Gemmatimonadetes bacterium]	RMH15744
KS_177	1292	SPV_cluster_421	75.291	429	0	SwfA	Type I polyketide synthase [bacterium symbiont of Plakortis simplex pPSA11D7]	AGH13590

KS_180	1028	SPV_cluster_432	65.116	344	5.45E-153		Beta-ketoacyl-ACP synthase II [Chloroflexi bacterium G233]	WP_098504503
KS_184	1217	SPV_cluster_430	68.148	405	0		3-Oxoacyl-[acyl-carrier-protein] synthase 2 [bacterium HR37]	GBD38054
KS_185	1052	SPV_cluster_411	53.374	326	3.08E-114		Beta-ketoacyl-[acyl-carrier-protein] synthase family protein [Rhodospirillaceae bacterium SYSU D60007]	WP_119262600
KS_187	1223	SPV_cluster_254	67.734	406	0		Beta-ketoacyl-[acyl-carrier-protein] synthase II [Gammaproteobacteria bacterium]	TDJ29040
KS_190	1223	SPV_cluster_284	73.037	382	0		Beta-ketoacyl-[acyl-carrier-protein] synthase II [SAR86 cluster bacterium]	PCJ26052
KS_192	1202	SPV_cluster_138	61.443	402	2.00E-176		Beta-ketoacyl-ACP synthase [Pseudoduganella violaceinigra]	WP_028104973
KS_194	1229	SPV_cluster_317	58.477	407	1.39E-150		Beta-ketoacyl-[acyl-carrier-protein] synthase II [Deltaproteobacteria bacterium]	TDJ21427
KS_200	1223	SPV_cluster_258	68.395	405	0		Beta-ketoacyl-[acyl-carrier-protein] synthase II [Acidobacteria bacterium]	RPH64142
KS_207	1223	SPV_cluster_389	75.979	383	0		Beta-ketoacyl-[acyl-carrier-protein] synthase II [Gammaproteobacteria bacterium]	TDJ17613
KS_209	1220	SPV_cluster_128	69.458	406	0		Beta-ketoacyl-[acyl-carrier-protein] synthase II [Gammaproteobacteria bacterium]	TDJ41741
KS_210	1214	SPV_cluster_009	68.564	404	0		3-Oxoacyl-(acyl-carrier-protein) synthase [uncultured actinobacterium HF0770_13M05]	ADI23284
KS_211	1340	SPV_cluster_001	53.437	451	7.41E-146		Hypothetical protein D7X12_09075, partial [Corallococcus sicarius]	RKH45034
KS_213	1229	SPV_cluster_441	54.657	408	5.13E-165		Beta-ketoacyl-ACP synthase II [Spirochaeta lutea]	WP_037548043
KS_214	1223	SPV_cluster_289	70.588	408	0		Beta-ketoacyl-[acyl-carrier-protein] synthase II [Gammaproteobacteria bacterium]	TDJ29040
KS_215	1214	SPV_cluster_239	71.921	406	7.58E-180		Beta-ketoacyl-ACP synthase II [Chloroflexi bacterium G233]	WP_098504331
KS_216	1226	SPV_cluster_003	61.425	407	4.00E-179		MULTISPECIES: Beta-ketoacyl-ACP synthase II [Pseudomonadaceae]	WP_096004673
KS_219	1199	SPV_cluster_148	72.475	396	0		MULTISPECIES: Beta-ketoacyl-[acyl-carrier-protein] synthase family protein [Rhodovulum]	WP_075786012
KS_226	1157	SPV_cluster_086	62.565	382	8.48E-158		Beta-ketoacyl synthase [Actinobacteria bacterium]	RUA23523
KS_228	1223	SPV_cluster_195	56.02	407	2.04E-173		Beta-ketoacyl-[acyl-carrier-protein] synthase II [Chloroflexi bacterium]	RMG78893
KS_229	1217	SPV_cluster_280	69.212	406	0		Beta-ketoacyl-[acyl-carrier-protein] synthase II [Gammaproteobacteria bacterium RBG_16_51_14]	OGT30519
KS_230	1223	SPV_cluster_037	71.253	407	0		MULTISPECIES: Beta-ketoacyl-ACP synthase II [Ectothiorhodospira]	WP_025281708
KS_233	1217	SPV_cluster_060	69.136	405	0		Beta-ketoacyl-[acyl-carrier-protein] synthase II [Gammaproteobacteria bacterium RBG_16_51_14]	OGT30519
KS_234	1217	SPV_cluster_121	69.458	406	0		Beta-ketoacyl-[acyl-carrier-protein] synthase II [Gammaproteobacteria bacterium RBG_16_51_14]	OGT30519
KS_238	1217	SPV_cluster_180	69.136	405	0		3-Oxoacyl-[acyl-carrier-protein] synthase 2 [bacterium HR37]	GBD38054
KS_241	1163	SPV_cluster_207	68.504	381	0		Beta-ketoacyl-[acyl-carrier-protein] synthase II	TDJ41741

							[Gammaproteobacteria bacterium]	
KS_242	1226	SPV_cluster_445	62.162	407	0		Beta-ketoacyl-[acyl-carrier-protein] synthase II [Proteobacteria bacterium]	PZN30994
KS_243	1217	SPV_cluster_015	70.968	403	0		Beta-ketoacyl-[acyl-carrier-protein] synthase II [Gammaproteobacteria bacterium]	TDJ36329
KS_245	1235	SPV_cluster_178	69.082	414	0		Beta-ketoacyl-ACP synthase II [Meinhardsimonia xiamenensis]	WP_092498652
KS_246	1187	SPV_cluster_126	50.598	251	1.93E-71		Beta-ketoacyl-[acyl-carrier-protein] synthase family protein [Legionella drozanskii]	WP_058494995
KS_248	1220	SPV_cluster_197	69.458	406	0		Beta-ketoacyl-[acyl-carrier-protein] synthase II [Gammaproteobacteria bacterium]	TDJ41741
KS_251	1247	SPV_cluster_101	73.058	412	0		Beta-ketoacyl-ACP synthase II [Rhodospirillaceae bacterium SYSU D60014]	WP_119459592
KS_255	1229	SPV_cluster_222	62.347	409	0		Beta-ketoacyl-ACP synthase II [Nitrosomonas aestuarii]	WP_090702733
KS_257	1292	SPV_cluster_426	75.524	429	0	SwfA	Type I polyketide synthase [bacterium symbiont of Plakortis simplex pPSA11D7]	AGH13590
KS_258	1220	SPV_cluster_036	64.268	403	2.41E-167		Beta-ketoacyl-[acyl-carrier-protein] synthase family protein [Acidobacteria bacterium]	REJ81214
KS_260	1226	SPV_cluster_419	62.347	409	0		Beta-ketoacyl-[acyl-carrier-protein] synthase II [Candidatus Handelsmanbacteria bacterium RIFCSPLOWO2_12_FULL_64_10]	OGG49603
KS_265	1292	SPV_cluster_091	78.322	429	0	SwfA	Type I polyketide synthase [bacterium symbiont of Plakortis simplex pPSA11D7]	AGH13590
KS_268	1292	SPV_cluster_172	92.381	420	0	SwfA	Type I polyketide synthase [bacterium symbiont of Plakortis simplex pPS11G3]	AGH13577
KS_271	1220	SPV_cluster_255	67.241	406	0		Beta-ketoacyl-[acyl-carrier-protein] synthase II [Acidobacteria bacterium]	RMH20421
KS_272	1286	SPV_cluster_005	80.14	428	0	SupA	SupA [Theonella swinhoei bacterial symbiont clone pSW1H8]	ABE03935
KS_273	1220	SPV_cluster_025	68.966	406	0		Beta-ketoacyl-[acyl-carrier-protein] synthase II [Acidobacteria bacterium]	RMH20421
KS_274	1226	SPV_cluster_024	71.144	402	0		Beta-ketoacyl-[acyl-carrier-protein] synthase II [Candidatus Handelsmanbacteria bacterium RIFCSPLOWO2_12_FULL_64_10]	OGG49603
KS_275	1229	SPV_cluster_031	65.366	410	0		Beta-ketoacyl-[acyl-carrier-protein] synthase II [Gemmatimonadetes bacterium]	RMH15744
KS_276	1223	SPV_cluster_217	72.414	406	0		Beta-ketoacyl-[acyl-carrier-protein] synthase II [Gammaproteobacteria bacterium TMED119]	OUV63604
KS_277	1151	SPV_cluster_129	78.272	382	0		Beta-ketoacyl synthase [Acidimicrobiales bacterium MED-G01]	PDH69975
KS_280	1151	SPV_cluster_229	79.167	384	2.73E-175		3-Oxoacyl-[acyl-carrier-protein] synthase II [Chloroflexi bacterium]	PZC49279
KS_281	1292	SPV_cluster_035	79.859	427	0	SupA	SupA [Theonella swinhoei bacterial symbiont clone pSW1H8]	ABE03935
KS_282	1226	SPV_cluster_159	83.619	409	0		3-Oxoacyl-[acyl-carrier-protein] synthase II [Chloroflexi bacterium]	PZC46532

KS_286	1229	SPV_cluster_402	63.882	407	0		Beta-ketoacyl-[acyl-carrier-protein] synthase II [Actinobacteria bacterium RBG_16_68_21]	OFW66625
KS_287	1289	SPV_cluster_214	71.262	428	0	SupA	SupA [Theonella swinhoei bacterial symbiont clone pSW1H8]	ABE03935
KS_288	1289	SPV_cluster_040	71.963	428	0	SupA	SupA [Theonella swinhoei bacterial symbiont clone pSW1H8]	ABE03935
KS_289	1286	SPV_cluster_225	64.371	421	0		SDR family NAD(P)-dependent oxidoreductase [Cocleimonas flava]	WP_131906443
KS_291	1223	SPV_cluster_107	69.779	407	0		Beta-ketoacyl-[acyl-carrier-protein] synthase II [Polyangiaceae bacterium UTPRO1]	OQY68774
KS_293	1292	SPV_cluster_161	83.411	428	0	SwfA	Type I polyketide synthase [bacterium symbiont of Plakortis simplex pPSA11D7]	AGH13590
KS_297	1214	SPV_cluster_085	68.069	404	0		Beta-ketoacyl-[acyl-carrier-protein] synthase II [Chloroflexi bacterium]	RME76096
KS_301	1154	SPV_cluster_093	53.786	383	3.88E-131		Hypothetical protein [Pelagimonas phthalicica]	WP_099241578
KS_303	1286	SPV_cluster_084	80.047	426	0	SupA	Polyketide synthase, partial [Aplysina aerophoba bacterial symbiont clone pAE27P20]	ABE03895
KS_305	1067	SPV_cluster_406	63.746	331	2.33E-150		Beta-ketoacyl-[acyl-carrier-protein] synthase II [Chloroflexi bacterium HGW-Chloroflexi-9]	PKN82890
KS_306	1286	SPV_cluster_268	79.859	427	0		Putative polyketide synthase [uncultured bacterium 2063G]	ABB73286
KS_307	1259	SPV_cluster_312	71.36	419	0		Beta-ketoacyl-[acyl-carrier-protein] synthase family protein [Deltaproteobacteria bacterium]	TDJ13656
KS_308	1154	SPV_cluster_124	51.558	353	8.38E-110		Hypothetical protein [Pelagimonas phthalicica]	WP_099241578
KS_310	1223	SPV_cluster_400	66.585	407	0		Beta-ketoacyl-[acyl-carrier-protein] synthase II [Chloroflexi bacterium]	TDI92680
KS_312	1160	SPV_cluster_113	70.605	347	1.84E-166		Beta-ketoacyl synthase [Actinobacteria bacterium]	RUA23523
KS_315	1160	SPV_cluster_320	69.452	383	0		Beta-ketoacyl synthase [Actinobacteria bacterium]	RUA23523
KS_317	1160	SPV_cluster_224	70.893	347	1.43E-168		Beta-ketoacyl synthase [Actinobacteria bacterium]	RUA23523
KS_321	1217	SPV_cluster_081	66.832	404	0		Beta-ketoacyl-[acyl-carrier-protein] synthase II [Chloroflexi bacterium]	RME76096
KS_323	1286	SPV_cluster_253	81.499	427	0		Putative polyketide synthase [uncultured bacterium 2063G]	ABB73286
KS_326	1223	SPV_cluster_353	80.882	408	0		3-Oxoacyl-[acyl-carrier-protein] synthase II [Chloroflexi bacterium]	PZC47099
KS_332	1286	SPV_cluster_171	80.562	427	0		Putative polyketide synthase [uncultured bacterium 2063G]	ABB73286
KS_336	1292	SPV_cluster_163	74.825	429	0	SwfA	Type I polyketide synthase [bacterium symbiont of Plakortis simplex pPSA11D7]	AGH13590
KS_337	1292	SPV_cluster_153	84.309	427	0	SwfA	Type I polyketide synthase [bacterium symbiont of Plakortis simplex pPSA11D7]	AGH13590
KS_339	1292	SPV_cluster_310	76.347	427	0	SwfA	Type I polyketide synthase [bacterium symbiont of Plakortis simplex pPSA11D7]	AGH13590
KS_341	1226	SPV_cluster_276	70.588	408	0		Beta-ketoacyl-[acyl-carrier-protein] synthase II [Chloroflexi bacterium]	RLT28345
KS_345	1154	SPV_cluster_290	59.791	383	1.03E-161		Beta-ketoacyl synthase [Acidimicrobiaceae bacterium TMED77]	OUV00831

KS_349	1247	SPV_cluster_205	73.913	414	0		3-Oxoacyl-[acyl-carrier-protein] synthase 2 [Alphaproteobacteria bacterium MarineAlpha10_Bin2]	PPR24356
KS_350	1292	SPV_cluster_300	73.893	429	0	SwfA	Type I polyketide synthase [bacterium symbiont of Plakortis simplex pPSA11D7]	AGH13590
KS_351	1148	SPV_cluster_436	74.084	382	0		3-Oxoacyl-[acyl-carrier-protein] synthase 2 [Alphaproteobacteria bacterium MarineAlpha10_Bin1]	PPR27313
KS_353	1292	SPV_cluster_108	76.566	431	0	SwfA	Type I polyketide synthase [bacterium symbiont of Plakortis simplex pPSA11D7]	AGH13590
KS_356	1292	SPV_cluster_166	71.852	405	0	SwfA	Type I polyketide synthase [bacterium symbiont of Plakortis simplex pPSA11D7]	AGH13590
KS_357	1292	SPV_cluster_281	77.049	427	0	SwfA	Type I polyketide synthase [bacterium symbiont of Plakortis simplex pPSA11D7]	AGH13590
KS_360	1232	SPV_cluster_192	70.244	410	0		Beta-ketoacyl-ACP synthase II [Rhodothermus marinus]	WP_014068140
KS_361	1292	SPV_cluster_065	61.593	427	7.04E-175	SwfA	Type I polyketide synthase [bacterium symbiont of Plakortis simplex pPSA11D7]	AGH13590
KS_362	1220	SPV_cluster_271	66.256	406	0		Beta-ketoacyl-ACP synthase II [Opitutaceae bacterium EW11]	WP_107743231
KS_363	1220	SPV_cluster_045	62.654	407	0		Beta-ketoacyl-[acyl-carrier-protein] synthase II [Chloroflexi bacterium]	RMG78893
KS_369	1229	SPV_cluster_007	67.883	411	0		Beta-ketoacyl-[acyl-carrier-protein] synthase II [Gemmatimonadetes bacterium]	RMH15744
KS_370	1100	SPV_cluster_112	52.381	252	7.01E-80		Putative Beta-ketoacyl synthase [Sorangium cellulosum So ce56]	CAN93510
KS_371	1298	SPV_cluster_141	78.089	429	0	SwfA	Type I polyketide synthase [bacterium symbiont of Plakortis simplex pPSA11D7]	AGH13590
KS_378	1223	SPV_cluster_363	75.567	397	0		Beta-ketoacyl-[acyl-carrier-protein] synthase II [Chloroflexi bacterium]	RLT35297
KS_379	1253	SPV_cluster_362	74.217	415	0		3-Oxoacyl-[acyl-carrier-protein] synthase 2 [Alphaproteobacteria bacterium MarineAlpha10_Bin2]	PPR24356
KS_381	1229	SPV_cluster_367	60.784	408	0		Beta-ketoacyl-[acyl-carrier-protein] synthase II [Chloroflexi bacterium TMED70]	OUU77203
KS_383	1220	SPV_cluster_027	76.733	404	0		Beta-ketoacyl-[acyl-carrier-protein] synthase II [Nitrospirae bacterium]	TAJ27615
KS_385	1229	SPV_cluster_341	69.343	411	0		Beta-ketoacyl-[acyl-carrier-protein] synthase II [Gemmatimonadetes bacterium]	RMH15744
KS_386	1232	SPV_cluster_173	69.756	410	0		Beta-ketoacyl-ACP synthase II [Rhodothermus marinus]	WP_014068140
KS_389	1247	SPV_cluster_042	71.671	413	0		Beta-ketoacyl-ACP synthase II [Rhodospirillaceae bacterium SYSU D60014]	WP_119459592
KS_391	1154	SPV_cluster_442	71.99	382	0		Beta-ketoacyl-ACP synthase II [Chloroflexi bacterium G233]	WP_098502948
KS_394	1226	SPV_cluster_053	59.459	407	1.95E-180		Beta-ketoacyl-[acyl-carrier-protein] synthase II [Chloroflexi bacterium]	PZN55531
KS_399	1286	SPV_cluster_057	91.335	427	0		Putative polyketide synthase [uncultured bacterium 2063G]	ABB73286
KS_401	1184	SPV_cluster_125	60.204	392	2.63E-169		Beta-ketoacyl-[acyl-carrier-protein] synthase family protein [Pseudovibrio ascidiaceicola]	WP_109314797

KS_402	1184	SPV_cluster_092	63.52	392	3.79E-171		Beta-ketoacyl-[acyl-carrier-protein] synthase family protein [Pelagimonas phthalicica]	WP_099241574
KS_404	1223	SPV_cluster_275	76.413	407	0		Beta-ketoacyl-[acyl-carrier-protein] synthase family protein [Chloroflexi bacterium]	RLT38592
KS_405	1292	SPV_cluster_049	88.038	418	0	SwfA	Type I polyketide synthase [bacterium symbiont of Plakortis simplex pPS11G3]	AGH13577
KS_409	1292	SPV_cluster_067	91.905	420	0	SwfA	Type I polyketide synthase [bacterium symbiont of Plakortis simplex pPS11G3]	AGH13577
KS_415	1232	SPV_cluster_308	61.481	405	2.38E-169		3-Oxoacyl-[acyl-carrier-protein] synthase II [Chloroflexi bacterium]	PZC48600
KS_416	1286	SPV_cluster_168	80.751	426	0	SupA	SupA [uncultured marine bacterium 1k6]	ACX49735
KS_418	1088	SPV_cluster_444	57.576	363	2.15E-151		Beta-ketoacyl-ACP synthase II [Desulfuribacillus stibiiarsenatis]	WP_069701864
KS_420	1286	SPV_cluster_371	79.625	427	0	SupA	SupA [Theonella swinhoei bacterial symbiont clone pSW1H8]	ABE03935
KS_422	1286	SPV_cluster_149	80.562	427	0	SupA	SupA [Theonella swinhoei bacterial symbiont clone pSW1H8]	ABE03935
KS_425	1232	SPV_cluster_201	74.453	411	0		Beta-ketoacyl-[acyl-carrier-protein] synthase II [Chloroflexi bacterium]	RLT35348
KS_426	1211	SPV_cluster_339	76.19	399	0		Beta-ketoacyl-ACP synthase II [Rhodospirillaceae bacterium SYSU D60014]	WP_119459592
KS_427	1175	SPV_cluster_215	68.901	373	2.00E-171		3-Oxoacyl-[acyl-carrier-protein] synthase II [Chloroflexi bacterium]	PZC48599
KS_432	1286	SPV_cluster_146	82.904	427	0		Putative polyketide synthase [uncultured bacterium 2063G]	ABB73286
KS_433	1175	SPV_cluster_307	69.169	373	6.29E-171		3-Oxoacyl-[acyl-carrier-protein] synthase II [Chloroflexi bacterium]	PZC48599
KS_434	1223	SPV_cluster_340	66.256	406	0		3-Oxoacyl-[acyl-carrier-protein] synthase II [Chloroflexi bacterium]	PZC46532
KS_435	1094	SPV_cluster_345	33.636	330	5.31E-47		Hypothetical protein [Pseudobacteriovorax antillogorgiicola]	WP_132314814
KS_436	1232	SPV_cluster_216	60.247	405	2.08E-167		3-Oxoacyl-[acyl-carrier-protein] synthase II [Chloroflexi bacterium]	PZC48600
KS_438	1223	SPV_cluster_301	66.502	406	0		3-Oxoacyl-[acyl-carrier-protein] synthase II [Chloroflexi bacterium]	PZC46532
KS_441	1046	SPV_cluster_443	69.164	347	1.37E-157		3-Oxoacyl-[acyl-carrier-protein] synthase II [Chloroflexi bacterium]	PZC48599
KS_442	1175	SPV_cluster_152	68.633	373	1.23E-170		3-Oxoacyl-[acyl-carrier-protein] synthase II [Chloroflexi bacterium]	PZC48599
KS_443	1175	SPV_cluster_414	83.014	365	0		3-Oxoacyl-[acyl-carrier-protein] synthase II [Chloroflexi bacterium]	PZC48599
KS_444	1235	SPV_cluster_083	68.613	411	0		Beta-ketoacyl-[acyl-carrier-protein] synthase II [Gemmatimonadetes bacterium]	RMH15744
KS_445	1286	SPV_cluster_071	80.328	427	0	SupA	SupA [Theonella swinhoei bacterial symbiont clone pSW1H8]	ABE03935
KS_450	1292	SPV_cluster_412	93.78	418	0	SwfA	Type I polyketide synthase [bacterium symbiont of Plakortis simplex pPS11G3]	AGH13577
KS_451	1289	SPV_cluster_114	87.119	427	0	SupA	SupA [Theonella swinhoei bacterial symbiont clone pSW1H8]	ABE03935

KS_456	1184	SPV_cluster_263	72.237	389	0		3-Oxoacyl-ACP synthase [Candidatus Tenderia electrophaga]	ALP54806
KS_457	1286	SPV_cluster_047	67.529	425	0	SupA	SupA [Aplysina aerophoba bacterial symbiont clone pAPKS18]	ABE03915
KS_458	1145	SPV_cluster_236	56.349	378	9.66E-133		Beta-ketoacyl-[acyl-carrier-protein] synthase family protein [Lysobacter sp.]	TBR14181
KS_463	1190	SPV_cluster_066	69.424	399	0		Beta-ketoacyl-ACP synthase II [Pararhodobacter aggregans]	WP_107749575
KS_464	1178	SPV_cluster_069	67.519	391	0		Beta-ketoacyl-ACP synthase II [Bryobacter aggregatus]	WP_031496688
KS_467	1196	SPV_cluster_364	54.839	403	2.73E-147		Beta-ketoacyl-ACP synthase II [Pseudobacteriovorax antillogorgiicola]	WP_132326354
KS_470	977	SPV_cluster_330	92.26	323	0		Beta-ketoacyl-ACP synthase II [Rhodospirillaceae bacterium Spongia-Bin9]	WP_108792873
KS_472	1211	SPV_cluster_259	74.121	398	0		Beta-ketoacyl-ACP synthase II [Telmatospirillum siberiense]	WP_101252614
KS_475	1154	SPV_cluster_366	72.324	383	0		Beta-ketoacyl-ACP synthase I [Thermopetrobacter sp. TC1]	WP_038035251
KS_477	1286	SPV_cluster_291	80.796	427	0	SupA	SupA [Theonella swinhoei bacterial symbiont clone pSW1H8]	ABE03935
KS_478	1142	SPV_cluster_380	49.296	213	1.09E-62		3-Oxoacyl-[acyl-carrier-protein] synthase II [Chloroflexi bacterium]	PZC49279
KS_480	1199	SPV_cluster_334	79.188	394	0		Beta-ketoacyl-ACP synthase II [Rhodospirillaceae bacterium SYSU D60014]	WP_119459592
KS_482	1172	SPV_cluster_182	65.553	389	1.66E-180		3-Oxoacyl-[acyl-carrier-protein] synthase II [Chloroflexi bacterium]	PZC47099
KS_485	1298	SPV_cluster_226	64.884	430	1.25E-177	SupA	SupA [Theonella swinhoei bacterial symbiont clone pSW1H8]	ABE03935
KS_487	1112	SPV_cluster_179	74.857	350	0		Beta-ketoacyl synthase [Acidimicrobiales bacterium MED-G01]	PDH69975
KS_489	1292	SPV_cluster_220	77.986	427	0	SwfA	Type I polyketide synthase [bacterium symbiont of Plakortis simplex pPSA11D7]	AGH13590
KS_492	1172	SPV_cluster_240	69.251	387	0		3-Oxoacyl-[acyl-carrier-protein] synthase 2 [bacterium HR29]	GBD22791
KS_493	1286	SPV_cluster_134	82.436	427	0	SupA	SupA [Theonella swinhoei bacterial symbiont clone pSW1H8]	ABE03935
KS_496	1166	SPV_cluster_274	65.81	389	0		3-Oxoacyl-[acyl-carrier-protein] synthase II [Chloroflexi bacterium]	PZC47099
KS_500	1286	SPV_cluster_278	70.023	427	0	SupA	SupA [Aplysina aerophoba bacterial symbiont clone pAPKS18]	ABE03915
KS_502	1286	SPV_cluster_213	78.454	427	0	SupA	SupA [Theonella swinhoei bacterial symbiont clone pSW1H8]	ABE03935
KS_509	1292	SPV_cluster_154	85.948	427	0		Putative polyketide synthase [uncultured bacterium 2063G]	ABB73286
KS_510	1292	SPV_cluster_020	85.714	427	0		Putative polyketide synthase [uncultured bacterium 2063G]	ABB73286
KS_512	1292	SPV_cluster_264	87.857	420	0	SwfA	Type I polyketide synthase [bacterium symbiont of Plakortis simplex pPS11G3]	AGH13577
KS_513	1160	SPV_cluster_316	65.195	385	1.49E-176		3-Oxoacyl-[acyl-carrier-protein] synthase II [Chloroflexi bacterium]	PZC47099
KS_515	1244	SPV_cluster_029	72.104	423	0		SA1_PKSA [uncultured bacterial symbiont of Discodermia dissoluta]	AAY00025
KS_522	1229	SPV_cluster_433	66.829	410	0		Beta-ketoacyl-[acyl-carrier-protein] synthase II [Gemmatimonadetes bacterium]	RMH15744

KS_523	1283	SPV_cluster_208	87.089	426	0	SupA	SupA [Aplysina aerophoba bacterial symbiont clone pAPKS18]	ABE03915
KS_524	1286	SPV_cluster_051	80.562	427	0		Putative polyketide synthase [uncultured bacterium 2063G]	ABB73286
KS_530	1292	SPV_cluster_174	82.904	427	0	SupA	SupA [Theonella swinhoei bacterial symbiont clone pSW1H8]	ABE03935
KS_531	1058	SPV_cluster_337	93.023	258	3.14E-168		Beta-ketoacyl-ACP synthase [Rhodospirillaceae bacterium Spongia-Bin9]	WP_108793187
KS_533	1292	SPV_cluster_368	77.049	427	0	SwfA	Type I polyketide synthase [bacterium symbiont of Plakortis simplex pPSA11D7]	AGH13590
KS_534	1286	SPV_cluster_326	80.986	426	0	SupA	SupA [Aplysina aerophoba bacterial symbiont clone pAPKS18]	ABE03915
KS_535	1286	SPV_cluster_269	79.391	427	0	SupA	SupA [Theonella swinhoei bacterial symbiont clone pSW1H8]	ABE03935
KS_539	1286	SPV_cluster_102	88.263	426	0	SupA	Polyketide synthase, partial [Aplysina aerophoba bacterial symbiont clone pAE27P20]	ABE03895
KS_542	1286	SPV_cluster_299	81.733	427	0		Putative polyketide synthase [uncultured bacterium 2063G]	ABB73286
KS_544	1286	SPV_cluster_078	66.042	427	2.45E-178		SA1_PKSA [uncultured bacterial symbiont of Discodermia dissoluta]	AAY00025
KS_547	1292	SPV_cluster_008	80.094	427	0	SupA	SupA [Theonella swinhoei bacterial symbiont clone pSW1H8]	ABE03935
KS_552	1277	SPV_cluster_169	74.533	428	0		SA1_PKSA [uncultured bacterial symbiont of Discodermia dissoluta]	AAY00025
KS_556	1286	SPV_cluster_212	82.201	427	0	SupA	SupA [Theonella swinhoei bacterial symbiont clone pSW1H8]	ABE03935
KS_560	1283	SPV_cluster_082	77.934	426	0	SupA	SupA [uncultured marine bacterium 1k6]	ACX49735
KS_561	1250	SPV_cluster_221	70.29	414	1.36E-178		SA1_PKSA [uncultured bacterial symbiont of Discodermia dissoluta]	AAY00025
KS_562	1292	SPV_cluster_151	87.119	427	0	SupA	SupA [Theonella swinhoei bacterial symbiont clone pSW1H8]	ABE03935
KS_565	1259	SPV_cluster_030	75.234	428	0		SA1_PKSA [uncultured bacterial symbiont of Discodermia dissoluta]	AAY00025
KS_566	1286	SPV_cluster_311	79.859	427	0	SupA	SupA [Theonella swinhoei bacterial symbiont clone pSW1H8]	ABE03935
KS_567	1301	SPV_cluster_246	74.766	428	0	SupA	SupA [Aplysina aerophoba bacterial symbiont clone pAPKS18]	ABE03915
KS_568	1166	SPV_cluster_250	62.029	345	1.21E-154		Beta-ketoacyl-[acyl-carrier-protein] synthase family protein [Verrucomicrobia bacterium]	RME70472
KS_571	1082	SPV_cluster_376	61.176	340	2.44E-145		Beta-ketoacyl-ACP synthase I [Deltaproteobacteria bacterium]	RLB53742
KS_574	1292	SPV_cluster_052	86.651	427	0		Putative polyketide synthase [uncultured bacterium 2063G]	ABB73286
KS_578	1178	SPV_cluster_094	61.675	394	3.52E-156		Beta-ketoacyl-[acyl-carrier-protein] synthase family protein [Verminephrobacter aporrectodeae]	WP_081479930
KS_582	1010	SPV_cluster_137	55.67	194	3.63E-63		Beta-ketoacyl-ACP synthase [Xanthomonadaceae bacterium]	RYD16404
KS_584	1025	SPV_cluster_136	67.213	244	1.60E-108		Beta-ketoacyl-[acyl-carrier-protein] synthase family protein [Parasulfuritortus cantonensis]	WP_131445552
KS_587	1184	SPV_cluster_123	59.836	366	3.15E-136		Beta-ketoacyl-[acyl-carrier-protein] synthase family protein [Verminephrobacter aporrectodeae]	WP_081479930
KS_589	1259	SPV_cluster_338	72.249	418	0	SupA	SupA [Theonella swinhoei bacterial symbiont clone pSW1H8]	ABE03935

KS_592	1277	SPV_cluster_106	74.941	423	0		SA1_PKSA [uncultured bacterial symbiont of Discodermia dissoluta]	AAY00025
KS_594	293	SPV_cluster_111	66.667	93	7.36E-33	SupA	SupA [Aplysina aerophoba bacterial symbiont clone pAPKS18]	ABE03915
KS_595	1259	SPV_cluster_357	68.182	418	1.73E-179	SupA	SupA [Theonella swinhoei bacterial symbiont clone pSW1H8]	ABE03935
KS_597	1256	SPV_cluster_309	74.641	418	0	SupA	SupA [Theonella swinhoei bacterial symbiont clone pSW1H8]	ABE03935
KS_598	1259	SPV_cluster_394	83.453	417	0	SupA	SupA [Aplysina aerophoba bacterial symbiont clone pAPKS18]	ABE03915
KS_600	1079	SPV_cluster_145	65.138	327	7.89E-143		Beta-ketoacyl synthase [Gammaproteobacteria bacterium]	TDJ41416
KS_601	992	SPV_cluster_261	62.963	243	2.24E-102		Beta-ketoacyl synthase [Proteobacteria bacterium]	PZM93747
KS_604	851	SPV_cluster_016	72.587	259	1.25E-129		Beta-ketoacyl synthase [Acidobacteria bacterium]	PYQ67687
KS_606	653	SPV_cluster_348	78.605	215	2.84E-108		Putative polyketide synthase [uncultured bacterium 2063G]	ABB73286
KS_610	287	SPV_cluster_459	84.783	92	1.63E-46	SupA	SupA [Aplysina aerophoba bacterial symbiont clone pAPKS18]	ABE03915
KS_612	1256	SPV_cluster_106	75.117	426	0		SA1_PKSA [uncultured bacterial symbiont of Discodermia dissoluta]	AAY00025
KS_622	1259	SPV_cluster_315	59.809	418	2.39E-159	SupA	SupA [Theonella swinhoei bacterial symbiont clone pSW1H8]	ABE03935
KS_623	1259	SPV_cluster_305	85.372	417	0	SupA	SupA [Aplysina aerophoba bacterial symbiont clone pAPKS18]	ABE03915
KS_624	1259	SPV_cluster_266	71.053	418	0	SupA	SupA [Theonella swinhoei bacterial symbiont clone pSW1H8]	ABE03935
KS_629	1259	SPV_cluster_314	80.096	417	0		Putative polyketide synthase [uncultured bacterium 2063G]	ABB73286
KS_631	1235	SPV_cluster_377	72.464	414	0		SA1_PKSA [uncultured bacterial symbiont of Discodermia dissoluta]	AAY00025
KS_632	1259	SPV_cluster_167	73.986	419	0	SupA	Polyketide synthase, partial [Aplysina aerophoba bacterial symbiont clone pAE27P20]	ABE03895
KS_634	1265	SPV_cluster_030	75.177	423	0		SA1_PKSA [uncultured bacterial symbiont of Discodermia dissoluta]	AAY00025
KS_635	1262	SPV_cluster_030	76.123	423	0		SA1_PKSA [uncultured bacterial symbiont of Discodermia dissoluta]	AAY00025
KS_639	1259	SPV_cluster_260	74.82	417	0	SupA	SupA [Aplysina aerophoba bacterial symbiont clone pAPKS18]	ABE03915
KS_641	1259	SPV_cluster_052	71.223	417	0		SupA [Aplysina aerophoba bacterial symbiont clone pAPKS18]	ABE03915
KS_648	1265	SPV_cluster_211	79.616	417	0		Putative polyketide synthase [uncultured bacterium 2063G]	ABB73286
KS_651	1256	SPV_cluster_227	68.66	418	0	SupA	SupA [Theonella swinhoei bacterial symbiont clone pSW1H8]	ABE03935
KS_653	1259	SPV_cluster_306	76.259	417	0	SupA	SupA [Aplysina aerophoba bacterial symbiont clone pAPKS18]	ABE03915
KS_656	1244	SPV_cluster_237	66.118	425	2.01E-175		SA1_PKSA [uncultured bacterial symbiont of Discodermia dissoluta]	AAY00025
KS_658	1259	SPV_cluster_030	75.65	423	0		SA1_PKSA [uncultured bacterial symbiont of Discodermia dissoluta]	AAY00025
KS_663	1259	SPV_cluster_206	69.305	417	0	SupA	SupA [Aplysina aerophoba bacterial symbiont clone pAPKS18]	ABE03915
KS_665	1259	SPV_cluster_210	81.535	417	0		Putative polyketide synthase [uncultured bacterium 2063G]	ABB73286

KS_667	1259	SPV_cluster_181	76.555	418	0	SupA	SupA [Theonella swinhoei bacterial symbiont clone pSW1H8]	ABE03935
KS_675	1268	SPV_cluster_030	74.292	424	0		SA1_PKSA [uncultured bacterial symbiont of Discodermia dissoluta]	AAY00025
KS_676	1259	SPV_cluster_082	69.856	418	0	SupA	SupA [Theonella swinhoei bacterial symbiont clone pSW1H8]	ABE03935
KS_678	1253	SPV_cluster_221	58.554	415	6.49E-145		SA1_PKSA [uncultured bacterial symbiont of Discodermia dissoluta]	AAY00025
KS_680	1277	SPV_cluster_030	73.995	423	0		SA1_PKSA [uncultured bacterial symbiont of Discodermia dissoluta]	AAY00025
KS_684	1268	SPV_cluster_030	74.704	423	0		SA1_PKSA [uncultured bacterial symbiont of Discodermia dissoluta]	AAY00025
KS_686	1256	SPV_cluster_191	69.378	418	0		SupA [Theonella swinhoei bacterial symbiont clone pSW1H8]	ABE03935
KS_687	1259	SPV_cluster_051	68.106	417	0		SupA [Aplysina aerophoba bacterial symbiont clone pAPKS18]	ABE03915
KS_690	1259	SPV_cluster_096	77.218	417	0	SupA	SupA [Aplysina aerophoba bacterial symbiont clone pAPKS18]	ABE03915
KS_694	1259	SPV_cluster_057	95.204	417	0		Putative polyketide synthase [uncultured bacterium 2063G]	ABB73286
KS_696	1259	SPV_cluster_171	80.096	417	0		Putative polyketide synthase [uncultured bacterium 2063G]	ABB73286
KS_698	1259	SPV_cluster_040	73.445	418	0	SupA	SupA [Theonella swinhoei bacterial symbiont clone pSW1H8]	ABE03935
KS_699	1259	SPV_cluster_214	73.684	418	0	SupA	SupA [Theonella swinhoei bacterial symbiont clone pSW1H8]	ABE03935
KS_702	1271	SPV_cluster_029	74.232	423	0		SA1_PKSA [uncultured bacterial symbiont of Discodermia dissoluta]	AAY00025
KS_703	1259	SPV_cluster_134	72.249	418	0	SupA	SupA [Theonella swinhoei bacterial symbiont clone pSW1H8]	ABE03935
KS_706	1259	SPV_cluster_035	66.986	418	0	SupA	SupA [Theonella swinhoei bacterial symbiont clone pSW1H8]	ABE03935
KS_709	1265	SPV_cluster_071	68.66	418	0	SupA	SupA [Theonella swinhoei bacterial symbiont clone pSW1H8]	ABE03935
KS_710	1262	SPV_cluster_114	83.014	418	0	SupA	SupA [Theonella swinhoei bacterial symbiont clone pSW1H8]	ABE03935
KS_711	1259	SPV_cluster_084	67.386	417	0	SupA	SupA [uncultured marine bacterium 1k6]	ACX49735
KS_713	1259	SPV_cluster_146	80.815	417	0		Putative polyketide synthase [uncultured bacterium 2063G]	ABB73286
KS_716	1259	SPV_cluster_020	69.065	417	0		SupA [Aplysina aerophoba bacterial symbiont clone pAPKS18]	ABE03915
KS_717	1265	SPV_cluster_047	63.636	418	2.86E-173	SupA	SupA [Theonella swinhoei bacterial symbiont clone pSW1H8]	ABE03935
KS_718	1259	SPV_cluster_154	72.488	418	0		SupA [Theonella swinhoei bacterial symbiont clone pSW1H8]	ABE03935
KS_721	1211	SPV_cluster_149	75.062	405	0	SupA	SupA [Theonella swinhoei bacterial symbiont clone pSW1H8]	ABE03935
KS_722	1259	SPV_cluster_245	72.01	418	0	SupA	SupA [Theonella swinhoei bacterial symbiont clone pSW1H8]	ABE03935
KS_725	1259	SPV_cluster_150	73.684	418	0	SupA	SupA [Theonella swinhoei bacterial symbiont clone pSW1H8]	ABE03935
KS_731	1262	SPV_cluster_151	74.224	419	0	SupA	Polyketide synthase, partial [Aplysina aerophoba bacterial symbiont clone pAE27P20]	ABE03895
KS_734	1259	SPV_cluster_174	70.264	417	0	SupA	SupA [Aplysina aerophoba bacterial symbiont clone pAPKS18]	ABE03915
KS_736	1259	SPV_cluster_005	74.163	418	0	SupA	SupA [Theonella swinhoei bacterial symbiont clone pSW1H8]	ABE03935

KS_737	1277	SPV_cluster_169	76.359	423	0		SA1_PKSA [uncultured bacterial symbiont of Discodermia dissoluta]	AAY00025
KS_739	1259	SPV_cluster_102	81.579	418	0	SupA	Polyketide synthase, partial [Aplysina aerophoba bacterial symbiont clone pAE27P20]	ABE03895
KS_740	1262	SPV_cluster_246	70.167	419	0	SupA	SupA [Theonella swinhoei bacterial symbiont clone pSW1H8]	ABE03935
KS_742	1262	SPV_cluster_115	67.225	418	0	SupA	SupA [Theonella swinhoei bacterial symbiont clone pSW1H8]	ABE03935
KS_743	1262	SPV_cluster_241	66.746	418	0	SupA	SupA [uncultured marine bacterium 1k6]	ACX49735
KS_745	1259	SPV_cluster_078	72.01	418	0		SupA [Theonella swinhoei bacterial symbiont clone pSW1H8]	ABE03935
KS_748	1262	SPV_cluster_168	68.66	418	0	SupA	SupA [Theonella swinhoei bacterial symbiont clone pSW1H8]	ABE03935
KS_750	1319	SPV_cluster_008	64.76	437	2.71E-174	SupA	SupA [Aplysina aerophoba bacterial symbiont clone pAPKS18]	ABE03915
KS_752	176	SPV_cluster_457	73.214	56	5.97E-22		SA1_PKSB [uncultured bacterial symbiont of Discodermia dissoluta]	AAY00026
KS_753	1262	SPV_cluster_030	75.887	423	0		SA1_PKSA [uncultured bacterial symbiont of Discodermia dissoluta]	AAY00025
KS_756	1250	SPV_cluster_030	72.471	425	0		SA1_PKSA [uncultured bacterial symbiont of Discodermia dissoluta]	AAY00025
KS_760	1268	SPV_cluster_029	74.468	423	0		SA1_PKSA [uncultured bacterial symbiont of Discodermia dissoluta]	AAY00025
KS_762	1277	SPV_cluster_106	67.376	423	0		SA1_PKSA [uncultured bacterial symbiont of Discodermia dissoluta]	AAY00025

Table S11. BLAST search of the KS sequences from the metagenome of *C. reinwardti* against the refseq_protein database in NCBI

KS ID	Length (nt)	BGC ID	Identity (%)	Alignment length (aa)	E-value	The most homologenus sequences	Accession number
KS_013	792	CLR_cluster_248	61.154	260	2.49E-100	Type I polyketide synthase [Mycobacterium]	WP_024450282
KS_029	1194	CLR_cluster_183	54.39	410	2.01E-125	Type I polyketide synthase [Mycobacterium]	WP_024450282
KS_030	792	CLR_cluster_249	59.231	260	9.27E-99	Type I polyketide synthase [Mycolicibacterium aurum]	WP_048632560
KS_032	327	CLR_cluster_265	81.31	110	3.00E-21	Type I polyketide synthase [Paenibacillus pinihumi]	WP_051237173
KS_039	1269	CLR_cluster_225	53.081	422	4.33E-142	Polyketide synthase [Stanieria cyanosphaera]	WP_015193130
KS_041	975	CLR_cluster_202	56.426	319	1.62E-105	Type I polyketide synthase [Mycobacterium asiaticum]	WP_065157110
KS_044	363	CLR_cluster_181	63.025	119	1.74E-44	Type I polyketide synthase [Mycobacterium malmoense]	WP_065480691
KS_061	1113	CLR_cluster_192	57.453	369	1.20E-129	Type I polyketide synthase [Mycobacterium sp. 852002-30065_SCH5024008]	WP_067203355
KS_064	1221	CLR_cluster_021	60.68	412	6.30E-146	Type I polyketide synthase [Mycobacterium avium]	WP_095785889
KS_066	702	CLR_cluster_246	63.203	231	3.07E-90	Type I polyketide synthase [Mycobacterium asiaticum]	WP_065157110
KS_073	1112	CLR_cluster_222	57.027	370	1.64E-128	Type I polyketide synthase [Mycobacterium sp. 852002-30065_SCH5024008]	WP_067203355
KS_078	1064	CLR_cluster_252	75.714	350	0	Beta-ketoacyl-[acyl-carrier-protein] synthase II [Telmatospirillum siberiense]	WP_101252614
KS_079	713	CLR_cluster_062	48.182	220	8.63E-55	Type I polyketide synthase [Chondromyces crocatus]	WP_063796357
KS_084	977	CLR_cluster_279	38.806	201	3.36E-29	Beta-ketoacyl-[acyl-carrier-protein] synthase family protein [Streptomyces atratus]	WP_072488766
KS_095	1049	CLR_cluster_281	64.571	350	4.18E-161	Beta-ketoacyl-[acyl-carrier-protein] synthase II [Halomonas elongata]	WP_013331756
KS_101	1100	CLR_cluster_081	44.048	252	2.58E-59	Beta-ketoacyl-[acyl-carrier-protein] synthase family protein [Acidobacteria bacterium KBS 146]	WP_026387381
KS_102	1097	CLR_cluster_105	43.529	340	3.24E-77	Hypothetical protein [Sandaracinus amylolyticus]	WP_053231614
KS_105	1223	CLR_cluster_122	58.824	408	3.39E-179	Beta-ketoacyl-[acyl-carrier-protein] synthase II [Risungbinella massiliensis]	WP_044642594
KS_106	1223	CLR_cluster_041	60.197	407	3.85E-180	Beta-ketoacyl-[acyl-carrier-protein] synthase II [Brevibacillus borstelensis]	WP_003386033
KS_111	1223	CLR_cluster_006	82.02	406	0	Beta-ketoacyl-[acyl-carrier-protein] synthase II [Thioalkalivibrio sp. HK1]	WP_038098656
KS_115	1223	CLR_cluster_007	59.705	407	2.56E-177	Beta-ketoacyl-[acyl-carrier-protein] synthase II [Clostridiisalibacter paucivorans]	WP_026893881
KS_116	1202	CLR_cluster_110	71.859	398	0	Beta-ketoacyl-ACP synthase I [Salinicola sp. MIT1003]	WP_071230737
KS_117	1250	CLR_cluster_086	73.366	413	0	Beta-ketoacyl-[acyl-carrier-protein] synthase II [Rhodospirillales bacterium URHD0088]	WP_027300572
KS_120	1226	CLR_cluster_193	58.333	408	6.75E-169	Beta-ketoacyl-[acyl-carrier-protein] synthase II [Thermoflexus hugenholtzii]	WP_098504503

KS_126	1184	CLR_cluster_230	75.949	395	0	Beta-ketoacyl-ACP synthase I [Methylarcula marina]	WP_106692425
KS_134	1199	CLR_cluster_253	67.506	397	0	Beta-ketoacyl-[acyl-carrier-protein] synthase family protein [Halocynthiibacter namhaensis]	WP_039018003
KS_138	1223	CLR_cluster_097	59.705	407	1.90E-180	Beta-ketoacyl-[acyl-carrier-protein] synthase II [Risungbinella massiliensis]	WP_044642594
KS_142	1208	CLR_cluster_126	75	400	0	Beta-ketoacyl-ACP synthase I [Inquilinus limosus]	WP_026872496
KS_146	1223	CLR_cluster_082	74.138	406	0	Beta-ketoacyl-[acyl-carrier-protein] synthase II [Thioalkalivibrio sp. HK1]	WP_038098656
KS_150	1196	CLR_cluster_127	68.09	398	0	Beta-ketoacyl-ACP synthase I [Halomonas sp. HL-48]	WP_027337473
KS_154	1223	CLR_cluster_013	64.039	406	0	Beta-ketoacyl-[acyl-carrier-protein] synthase II [Moorella humiferrea]	WP_106006374
KS_159	1223	CLR_cluster_101	66.422	408	0	Beta-ketoacyl-[acyl-carrier-protein] synthase II [Pyrinomonas methylaliphatogenes]	WP_041976954
KS_163	1229	CLR_cluster_065	58.293	410	1.85E-168	Beta-ketoacyl-[acyl-carrier-protein] synthase II [Desmospora sp. 8437]	WP_040387222
KS_170	1217	CLR_cluster_085	58.025	405	4.80E-177	Beta-ketoacyl-[acyl-carrier-protein] synthase II [Bacillales]	WP_029100165
KS_179	1202	CLR_cluster_257	67.75	400	0	Beta-ketoacyl-ACP synthase I [Methyloterricola oryzae]	WP_045223829
KS_183	1223	CLR_cluster_165	57.248	407	3.69E-179	Beta-ketoacyl-[acyl-carrier-protein] synthase II [Planifilum fimeticola]	WP_106344983
KS_196	1280	CLR_cluster_139	43.972	423	3.07E-119	Hypothetical protein [Syntrophobacter sp. SbD1]	WP_106821393
KS_198	1223	CLR_cluster_001	71.253	407	0	Beta-ketoacyl-[acyl-carrier-protein] synthase II [Ectothiorhodospira haloalkaliphila]	WP_025281708
KS_199	1157	CLR_cluster_264	54.45	382	4.06E-134	Beta-ketoacyl-ACP synthase II [Ferrimicrobium acidiphilum]	WP_052566261
KS_201	1226	CLR_cluster_022	61.52	408	9.13E-180	Beta-ketoacyl-[acyl-carrier-protein] synthase II [Nitrosomonas europaea]	WP_011112207
KS_204	1163	CLR_cluster_064	62.304	382	1.50E-179	Beta-ketoacyl-[acyl-carrier-protein] synthase II [Pseudomonas pachastrellae]	WP_083725910
KS_206	1226	CLR_cluster_143	61.916	407	0	Beta-ketoacyl-[acyl-carrier-protein] synthase II [Pseudomonas pachastrellae]	WP_083725910
KS_208	1223	CLR_cluster_179	64.691	405	0	Beta-ketoacyl-[acyl-carrier-protein] synthase II [Pyrinomonas methylaliphatogenes]	WP_041976954
KS_212	1217	CLR_cluster_190	58.519	405	4.55E-177	Beta-ketoacyl-[acyl-carrier-protein] synthase II [Bacillales]	WP_029100165
KS_217	1217	CLR_cluster_015	64.109	404	0	Beta-ketoacyl-[acyl-carrier-protein] synthase II [Pseudomonas pachastrellae]	WP_083725910
KS_221	1064	CLR_cluster_102	69.057	265	2.37E-130	Beta-ketoacyl-ACP synthase [Cupriavidus oxalaticus]	WP_063237600
KS_222	1229	CLR_cluster_134	54.39	410	2.49E-144	Beta-ketoacyl-[acyl-carrier-protein] synthase II [Alicyclobacillus macrosporangiidus]	WP_074950143
KS_227	1217	CLR_cluster_220	65.926	405	0	Beta-ketoacyl-[acyl-carrier-protein] synthase II [Acidihalobacter ferrooxidans]	WP_076837285
KS_231	1217	CLR_cluster_049	66.256	406	0	Beta-ketoacyl-[acyl-carrier-protein] synthase II [Thioflavicoccus mobilis]	WP_015281247
KS_232	1223	CLR_cluster_224	65.517	406	0	Beta-ketoacyl-[acyl-carrier-protein] synthase II [Dyella]	WP_026634449

KS_239	1226	CLR_cluster_166	61.425	407	2.27E-179	Beta-ketoacyl-[acyl-carrier-protein] synthase II [Pseudomonas abyssi]	WP_096004673
KS_247	1223	CLR_cluster_162	67.734	406	0	Beta-ketoacyl-[acyl-carrier-protein] synthase II [Halomonas sp. A11-A]	WP_110068027
KS_250	1340	CLR_cluster_014	52.954	457	4.05E-139	Type I Polyketide synthase [Chondromyces crocatus]	WP_082362498
KS_253	1229	CLR_cluster_228	56.829	410	1.52E-163	Beta-ketoacyl-[acyl-carrier-protein] synthase II [Rhodothermus marinus]	WP_012845172
KS_262	1220	CLR_cluster_045	63.054	406	0	Beta-ketoacyl-[acyl-carrier-protein] synthase II [Thermoanaerobaculum aquaticum]	WP_038046901
KS_263	1292	CLR_cluster_076	44.749	438	4.91E-94	Type I polyketide synthase [Sciscionella marina]	WP_020497474
KS_266	1151	CLR_cluster_140	58.377	382	1.34E-141	Beta-ketoacyl-ACP synthase II [Streptomyces sp. NRRL F-2664]	WP_030765373
KS_279	1229	CLR_cluster_147	62.347	409	0	Beta-ketoacyl-[acyl-carrier-protein] synthase II [Nitrosomonas aestuarii]	WP_090702733
KS_283	1286	CLR_cluster_131	63.593	423	4.29E-180	Type I polyketide synthase [Marinobacterium profundum]	WP_067295857
KS_284	1292	CLR_cluster_108	41.136	440	4.86E-87	Type I polyketide synthase [Crossiella equi]	WP_086785133
KS_294	1223	CLR_cluster_060	65.517	406	0	Beta-ketoacyl-[acyl-carrier-protein] synthase II [Geobacter sp. DSM 2909]	WP_108103150
KS_302	1154	CLR_cluster_043	51.558	353	4.76E-110	Hypothetical protein [Tropicibacter phthalicicus]	WP_099241578
KS_309	1160	CLR_cluster_114	60.807	347	1.90E-131	Beta-ketoacyl-ACP synthase II [Streptomyces katrae]	WP_045950542
KS_311	1259	CLR_cluster_137	60.143	419	6.11E-179	Beta-ketoacyl-[acyl-carrier-protein] synthase family protein [Syntrophus aciditrophicus]	WP_011418884
KS_314	1160	CLR_cluster_214	61.96	347	3.94E-133	Beta-ketoacyl-ACP synthase II [Streptomyces katrae]	WP_045950542
KS_322	1214	CLR_cluster_098	55.941	404	9.82E-156	Beta-ketoacyl-[acyl-carrier-protein] synthase II [Thermorudis peleae]	WP_038037697
KS_325	1286	CLR_cluster_056	53.333	435	2.05E-138	Type I polyketide synthase, partial [Burkholderia vietnamiensis]	WP_059694304
KS_327	1154	CLR_cluster_009	53.786	383	2.20E-131	Hypothetical protein [Tropicibacter phthalicicus]	WP_099241578
KS_329	1160	CLR_cluster_069	62.402	383	7.80E-149	Beta-ketoacyl-ACP synthase II [Streptomyces katrae]	WP_045950542
KS_330	1223	CLR_cluster_201	60.197	407	0	Beta-ketoacyl-[acyl-carrier-protein] synthase II [Kroppenstedtia eburnea]	WP_076523249
KS_334	1229	CLR_cluster_079	56.968	409	1.29E-157	Beta-ketoacyl-[acyl-carrier-protein] synthase II [Desulfovirgula thermocuniculi]	WP_027718279
KS_338	1292	CLR_cluster_133	41.609	435	1.97E-87	Type I polyketide synthase [Streptomyces]	WP_028443132
KS_340	1154	CLR_cluster_118	58.398	387	1.23E-128	Beta-ketoacyl-ACP synthase II [Ilumatobacter nonamiensis]	WP_040493091
KS_342	1292	CLR_cluster_089	42.759	435	4.48E-89	Type I polyketide synthase [Streptomyces]	WP_028443132
KS_346	1229	CLR_cluster_096	56.098	410	7.70E-159	Beta-ketoacyl-[acyl-carrier-protein] synthase II [Melghirimyces profundicolus]	WP_108022554
KS_354	1232	CLR_cluster_231	70	410	0	Beta-ketoacyl-[acyl-carrier-protein] synthase II [Rhodothermus marinus]	WP_014068140
KS_358	1292	CLR_cluster_055	39.066	407	6.07E-79	Type I polyketide synthase [Stigmatella erecta]	WP_093522399
KS_364	1292	CLR_cluster_027	39.723	433	5.71E-80	Type I polyketide synthase [Burkholderia thailandensis]	WP_038712449
KS_366	1229	CLR_cluster_025	58.435	409	2.77E-164	Beta-ketoacyl-[acyl-carrier-protein] synthase II [Desulfovirgula thermocuniculi]	WP_027718279

KS_368	1247	CLR_cluster_204	71.981	414	0	Beta-ketoacyl-[acyl-carrier-protein] synthase II [Tistrella mobilis]	WP_014745380
KS_372	1298	CLR_cluster_132	42.759	435	3.75E-87	Type I polyketide synthase [Streptomyces]	WP_030626723
KS_373	1220	CLR_cluster_030	73.515	404	0	Beta-ketoacyl-[acyl-carrier-protein] synthase II [Candidatus Nitrospira nitrosa]	WP_090746581
KS_375	1100	CLR_cluster_032	48.221	253	1.18E-58	3-oxoacyl-ACP synthase [Lechevalieria fradiae]	WP_090045424
KS_376	1007	CLR_cluster_275	54.192	334	8.45E-119	Beta-ketoacyl-[acyl-carrier-protein] synthase II [Thermaerobacter marianensis]	WP_013495369
KS_382	1292	CLR_cluster_087	42.069	435	4.46E-85	Type I polyketide synthase, partial [Streptomyces sp. SM5]	WP_103492552
KS_384	1220	CLR_cluster_261	52.826	407	3.49E-158	Beta-ketoacyl-[acyl-carrier-protein] synthase II [Bacillus methanolicus]	WP_004433597
KS_393	1247	CLR_cluster_200	70.944	413	0	Beta-ketoacyl-[acyl-carrier-protein] synthase II [Magnetospira sp. QH-2]	WP_046021768
KS_395	995	CLR_cluster_262	70.517	329	4.29E-166	Beta-ketoacyl-[acyl-carrier-protein] synthase II [Tistlia consotensis]	WP_085125958
KS_403	1286	CLR_cluster_135	56.744	430	2.81E-139	Type I polyketide synthase [Mycolicibacterium austroafricanum]	WP_105389463
KS_407	1184	CLR_cluster_010	63.265	392	5.45E-171	Beta-ketoacyl-[acyl-carrier-protein] synthase family protein [Tropicibacter phthalicicus]	WP_099241574
KS_412	1223	CLR_cluster_191	58.088	408	3.16E-168	Beta-ketoacyl-[acyl-carrier-protein] synthase II [Thermoflexus hugenholtzii]	WP_098502948
KS_413	1184	CLR_cluster_042	62.5	392	4.71E-169	Beta-ketoacyl-[acyl-carrier-protein] synthase family protein [Tropicibacter phthalicicus]	WP_099241574
KS_414	1292	CLR_cluster_106	43.333	450	2.20E-93	Type I polyketide synthase [Sciscionella marina]	WP_020497474
KS_417	965	CLR_cluster_251	54.545	319	6.35E-117	Beta-ketoacyl-[acyl-carrier-protein] synthase II [Thermorudis peleae]	WP_038037697
KS_419	1286	CLR_cluster_212	59.207	429	2.35E-148	Type I polyketide synthase [Mycolicibacterium austroafricanum]	WP_105389463
KS_421	710	CLR_cluster_278	57.384	237	5.78E-85	Type I polyketide synthase [Moorea producens]	WP_071104824
KS_423	1064	CLR_cluster_218	63.636	352	1.60E-132	Hybrid non-ribosomal peptide synthetase/Type I polyketide synthase [Methylosinus sp. LW4]	WP_026191724
KS_424	1211	CLR_cluster_104	73.367	398	0	Beta-ketoacyl-[acyl-carrier-protein] synthase II [Rhodospirillales bacterium URHD0088]	WP_027300572
KS_428	1292	CLR_cluster_053	40.367	436	3.69E-87	Type I polyketide synthase [Sciscionella marina]	WP_020497474
KS_431	1235	CLR_cluster_184	57.664	411	1.35E-171	Beta-ketoacyl-[acyl-carrier-protein] synthase II [Desmospora sp. 8437]	WP_040387222
KS_440	1286	CLR_cluster_219	54.861	432	3.55E-141	Type I polyketide synthase, partial [Burkholderia vietnamiensis]	WP_059895285
KS_447	1292	CLR_cluster_177	40.138	436	4.26E-89	SDR family NAD(P)-dependent oxidoreductase [Leucothrix sp. IMCC9719]	WP_109824032
KS_454	1184	CLR_cluster_121	73.846	390	0	Beta-ketoacyl-[acyl-carrier-protein] synthase II [Thioalkalivibrio sp. HK1]	WP_038098656
KS_455	1286	CLR_cluster_203	53.828	431	2.74E-130	Type I polyketide synthase, partial [Mycobacterium parascrofulaceum]	WP_007169537
KS_461	1211	CLR_cluster_145	74.121	398	0	Beta-ketoacyl-[acyl-carrier-protein] synthase II [Telmatospirillum siberiense]	WP_101252614
KS_465	983	CLR_cluster_141	67.485	326	2.10E-159	Beta-ketoacyl-[acyl-carrier-protein] synthase II [Bryobacter aggregatus]	WP_031496688
KS_466	1190	CLR_cluster_128	69.424	399	0	Beta-ketoacyl-[acyl-carrier-protein] synthase II [Pararhodobacter	WP_107749575

						aggregans]	
KS_473	1175	CLR_cluster_012	81.538	390	0	Beta-ketoacyl-[acyl-carrier-protein] synthase II [Thioalkalivibrio sp. HK1]	WP_038098656
KS_479	1184	CLR_cluster_112	60.459	392	6.45E-180	Beta-ketoacyl-[acyl-carrier-protein] synthase II [Vulcanibacillus modesticaldus]	WP_069655673
KS_483	1112	CLR_cluster_017	62.216	352	1.20E-134	Beta-ketoacyl-ACP synthase II [Ilumatobacter nonamiensis]	WP_040493091
KS_488	1076	CLR_cluster_277	59.889	359	6.71E-152	Beta-ketoacyl-[acyl-carrier-protein] synthase II [Melioribacter roseus]	WP_014857116
KS_503	1286	CLR_cluster_174	54.545	429	2.10E-139	Hybrid non-ribosomal peptide synthetase/Type I polyketide synthase [Burkholderia vietnamiensis]	WP_059454702
KS_504	1157	CLR_cluster_176	44.615	390	8.25E-88	Type I polyketide synthase [Nocardiopsis sp. NRRL B-16309]	WP_053618745
KS_505	1286	CLR_cluster_005	52.727	440	3.19E-138	Type I polyketide synthase [Mycobacterium sp. IS-1590]	WP_064404744
KS_508	1292	CLR_cluster_107	43.218	435	1.69E-88	Type I polyketide synthase [Streptomyces rubellomurinus]	WP_078860657
KS_514	1184	CLR_cluster_072	63.265	392	0	Beta-ketoacyl-[acyl-carrier-protein] synthase II [Thermodesulfovibrio sp. N1]	WP_068860060
KS_518	1244	CLR_cluster_144	61.557	424	2.54E-152	Type I polyketide synthase [Mycolicibacterium austroafricanum]	WP_051558318
KS_521	1220	CLR_cluster_156	63.027	403	0	Beta-ketoacyl-[acyl-carrier-protein] synthase II [Albimonas donghaensis]	WP_092679786
KS_527	1286	CLR_cluster_149	57.674	430	1.27E-150	Type I polyketide synthase [Mycolicibacterium diernhoferi]	WP_073855454
KS_528	1292	CLR_cluster_024	52.214	429	1.95E-134	Hybrid non-ribosomal peptide synthetase/Type I polyketide synthase [Burkholderia vietnamiensis]	WP_059603382
KS_536	1292	CLR_cluster_029	53.846	429	2.94E-140	Hybrid non-ribosomal peptide synthetase/Type I polyketide synthase [Burkholderia vietnamiensis]	WP_059740130
KS_537	1286	CLR_cluster_153	53.132	431	3.70E-136	Type I polyketide synthase [Mycolicibacterium rhodesiae]	WP_005142704
KS_540	1286	CLR_cluster_040	54.312	429	5.27E-139	Type I polyketide synthase, partial [Burkholderia vietnamiensis]	WP_059567396
KS_546	1286	CLR_cluster_159	57.11	429	4.66E-145	Type I polyketide synthase [Mycolicibacterium obuense]	WP_048421716
KS_549	1283	CLR_cluster_123	60.047	428	3.16E-150	Type I polyketide synthase [Mycolicibacterium austroafricanum]	WP_051558318
KS_550	1277	CLR_cluster_066	62.441	426	2.34E-159	Type I polyketide synthase [Mycolicibacterium austroafricanum]	WP_105389463
KS_557	1301	CLR_cluster_194	58.837	430	2.48E-146	Type I polyketide synthase [Mycolicibacterium austroafricanum]	WP_105389463
KS_558	1250	CLR_cluster_059	60.28	428	4.17E-151	Type I polyketide synthase [Mycolicibacterium austroafricanum]	WP_105389463
KS_563	1292	CLR_cluster_034	53.613	429	2.01E-140	Type I polyketide synthase, partial [Burkholderia vietnamiensis]	WP_059567396
KS_564	1286	CLR_cluster_124	55.349	430	2.54E-141	Type I polyketide synthase [Mycolicibacterium tusciae]	WP_006241768
KS_576	1094	CLR_cluster_232	50.138	363	6.06E-119	Beta-ketoacyl-[acyl-carrier-protein] synthase II [Effusibacillus pohliae]	WP_026174345
KS_579	1178	CLR_cluster_008	61.675	394	2.00E-156	Hypothetical protein [Verminephrobacter aporrectodeae]	WP_081479930
KS_580	1010	CLR_cluster_070	53.266	199	2.08E-62	Beta-ketoacyl-ACP synthase [Cellvibrio sp. pealriver]	WP_082191725
KS_585	989	CLR_cluster_221	65.702	242	2.23E-103	Beta-ketoacyl-ACP synthase [Halothiobacillus neapolitanus]	WP_041601094
KS_586	1184	CLR_cluster_044	59.836	366	1.79E-136	Hypothetical protein [Verminephrobacter aporrectodeae]	WP_081479930

KS_590	1094	CLR_cluster_020	65.479	365	5.69E-147	Type I polyketide synthase [Mycobacterium sp. 852002-53434_SCH5985345]	WP_066952779
KS_591	1283	CLR_cluster_026	60.941	425	5.36E-159	Type I polyketide synthase [Mycobacterium sp. EPa45]	WP_047332984
KS_602	722	CLR_cluster_247	45.455	242	3.01E-56	Type I polyketide synthase [Halomicronema hongdechloris]	WP_088431332
KS_605	851	CLR_cluster_109	68.726	259	3.09E-125	Beta-ketoacyl synthase [Vitiosangium sp. GDMCC 1.1324]	WP_108066665
KS_607	653	CLR_cluster_068	65.728	213	9.29E-86	Type I polyketide synthase [Chondromyces crocatus]	WP_082362832
KS_608	599	CLR_cluster_234	66.327	196	9.91E-78	Type I polyketide synthase [Mycobacterium palustre]	WP_085077785
KS_609	545	CLR_cluster_163	63.218	174	4.67E-63	Type I polyketide synthase [Mycobacterium]	WP_024450282
KS_611	449	CLR_cluster_235	50.336	149	4.12E-34	Type I Polyketide synthase [Sciscionella sp. SE31]	WP_031469821
KS_613	362	CLR_cluster_180	63.025	119	1.09E-44	Type I polyketide synthase [Mycobacterium malmoense]	WP_065480691
KS_615	1262	CLR_cluster_197	54.953	424	7.75E-136	Type I polyketide synthase [Mycobacterium sp. 852002-51613_SCH5001154]	WP_067261613
KS_616	341	CLR_cluster_245	69.091	110	1.93E-42	Type I polyketide synthase [Mycobacterium palustre]	WP_085077785
KS_617	233	CLR_cluster_250	62.162	74	1.44E-21	Type I polyketide synthase [Streptomyces oceani]	WP_070196673
KS_618	233	CLR_cluster_255	59.459	74	1.32E-21	Type I polyketide synthase [Archangium violaceum]	WP_052519209
KS_619	245	CLR_cluster_268	62.5	80	5.18E-25	Type I polyketide synthase [Streptomyces oceani]	WP_070196673
KS_620	1259	CLR_cluster_125	56.44	427	2.21E-141	Type I polyketide synthase [Mycolicibacterium tusciae]	WP_006241768
KS_626	1265	CLR_cluster_136	57.412	425	6.81E-145	Type I polyketide synthase [Mycolicibacterium austroafricanum]	WP_051558318
KS_630	1259	CLR_cluster_175	60.235	425	2.83E-148	Type I polyketide synthase [Mycolicibacterium austroafricanum]	WP_105389463
KS_633	1265	CLR_cluster_093	59.811	423	6.27E-149	Type I polyketide synthase [Mycolicibacterium austroafricanum]	WP_051558318
KS_637	1262	CLR_cluster_160	61.412	425	3.76E-157	Type I polyketide synthase [Mycolicibacterium austroafricanum]	WP_105389463
KS_640	1259	CLR_cluster_154	54.273	433	1.56E-137	Type I polyketide synthase [Mycobacterium avium]	WP_033720430
KS_644	1256	CLR_cluster_026	61.647	425	2.67E-156	Type I polyketide synthase [Mycolicibacterium austroafricanum]	WP_105389463
KS_645	1259	CLR_cluster_161	57.075	424	4.43E-144	Type I polyketide synthase [Mycolicibacterium vanbaalenii]	WP_011778276
KS_646	1256	CLR_cluster_117	58.156	423	4.92E-141	Type I polyketide synthase [Mycolicibacterium austroafricanum]	WP_051558318
KS_649	1259	CLR_cluster_080	54.93	426	4.19E-136	Type I polyketide synthase [Mycolicibacterium austroafricanum]	WP_105389463
KS_654	1244	CLR_cluster_058	57.569	436	3.25E-145	Type I polyketide synthase [Mycolicibacterium austroafricanum]	WP_105389463
KS_659	1259	CLR_cluster_047	60.142	424	6.00E-151	Type I polyketide synthase [Mycolicibacterium austroafricanum]	WP_105389463
KS_661	1265	CLR_cluster_103	55.529	425	3.02E-140	Type I polyketide synthase [Mycobacterium parmense]	WP_085270770
KS_669	1235	CLR_cluster_208	53.938	419	2.05E-136	Type I polyketide synthase [Mycobacterium palustre]	WP_085077785
KS_671	1259	CLR_cluster_048	56.206	427	4.81E-142	Type I polyketide synthase [Mycobacterium gordonae]	WP_065044175
KS_674	1268	CLR_cluster_020	62.411	423	3.97E-160	Type I polyketide synthase [Mycolicibacterium austroafricanum]	WP_105389463
KS_681	1277	CLR_cluster_075	60.78	436	8.34E-157	Type I polyketide synthase [Mycolicibacterium austroafricanum]	WP_105389463

KS_683	1268	CLR_cluster_075	60.757	423	2.61E-154	Type I polyketide synthase [Mycobacterium sp. TBL 1200985]	WP_085324847
KS_688	1259	CLR_cluster_068	54.717	424	1.34E-138	Type I polyketide synthase [Mycobacterium sp. 1245111.1]	WP_067331050
KS_695	1259	CLR_cluster_056	58.156	423	2.13E-143	Type I polyketide synthase [Mycolicibacterium austroafricanum]	WP_105389463
KS_701	1271	CLR_cluster_173	60.757	423	3.73E-153	Type I polyketide synthase [Mycobacterium asiaticum]	WP_065146316
KS_705	1259	CLR_cluster_206	53.412	425	1.06E-137	Type I polyketide synthase [Mycolicibacterium vanbaalenii]	WP_011778276
KS_708	1262	CLR_cluster_242	54.439	428	3.32E-139	Type I polyketide synthase [Mycolicibacterium bacteremicum]	WP_083056565
KS_720	1259	CLR_cluster_155	54.588	425	7.94E-135	Type I polyketide synthase [Mycobacterium avium]	WP_062889895
KS_726	1259	CLR_cluster_005	57.176	425	4.59E-142	Type I polyketide synthase [Mycolicibacterium austroafricanum]	WP_105389463
KS_728	1259	CLR_cluster_057	56	425	4.08E-135	Type I polyketide synthase [Mycolicibacterium austroafricanum]	WP_105389463
KS_730	1265	CLR_cluster_040	56.132	424	1.84E-134	Type I polyketide synthase [Mycolicibacterium austroafricanum]	WP_105389463
KS_732	1262	CLR_cluster_034	55.164	426	1.85E-139	Type I polyketide synthase [Mycolicibacterium vanbaalenii]	WP_011778276
KS_747	1109	CLR_cluster_146	65.847	366	1.18E-149	Type I polyketide synthase [Mycolicibacterium austroafricanum]	WP_105389463
KS_749	1319	CLR_cluster_050	52.822	443	2.88E-137	Type I polyketide synthase [Mycolicibacterium austroafricanum]	WP_105389463
KS_751	224	CLR_cluster_130	62.5	72	5.60E-21	Type I polyketide synthase [Streptomyces bungoensis]	WP_079060019
KS_755	1262	CLR_cluster_047	61.939	423	5.53E-157	Type I polyketide synthase [Mycolicibacterium austroafricanum]	WP_105389463
KS_757	1250	CLR_cluster_020	59.155	426	6.74E-151	Type I polyketide synthase [Mycolicibacterium austroafricanum]	WP_105389463
KS_761	1277	CLR_cluster_026	58.156	423	1.25E-144	Type I polyketide synthase [Mycobacterium sp. IS-1744]	WP_064399965

Table S12. BLAST search of the KS sequences from the metagenome of *R. globostellata* against the refseq_protein database in NCBI

KS ID	Length (nt)	BGC ID	Identity (%)	Alignment length (aa)	E-value	The homologenus sequences	Accession number
KS_001	696	RHG_cluster_258	67.686	229	1.03E-91	Type I polyketide synthase [Mycolicibacterium austroafricanum]	WP_051558318
KS_004	1185	RHG_cluster_332	51.005	398	2.89E-135	Beta-ketoacyl-[acyl-carrier-protein] synthase II [Phormidium willei]	WP_068789276
KS_006	1128	RHG_cluster_316	57.808	365	4.49E-129	Type I polyketide synthase [Mycobacterium sp. 1245805.9]	WP_067160573
KS_007	1059	RHG_cluster_341	42.407	349	1.39E-74	Type I polyketide synthase [Mycolicibacter sinensis]	WP_065023448
KS_010	1176	RHG_cluster_342	59.788	378	1.42E-136	Type I polyketide synthase [Mycolicibacterium austroafricanum]	WP_105389463
KS_011	801	RHG_cluster_340	52.574	272	4.83E-90	Type I polyketide synthase [Mycobacterium sp. EPG1]	WP_104861258
KS_012	429	RHG_cluster_282	65.942	138	5.20E-56	Type I polyketide synthase [Mycobacterium sp. YC-RL4]	WP_082901975
KS_014	1086	RHG_cluster_275	54.571	361	1.38E-141	Beta-ketoacyl-[acyl-carrier-protein] synthase II [Thermosulfidibacter takaii]	WP_068550102
KS_016	1239	RHG_cluster_191	62.028	424	6.86E-157	Type I polyketide synthase [Mycobacterium asiaticum]	WP_065036774
KS_019	1017	RHG_cluster_352	68.437	339	3.82E-176	Beta-ketoacyl-ACP synthase I [Methyloterricola oryzae]	WP_045223829
KS_020	705	RHG_cluster_308	65.939	229	3.01E-91	Type I polyketide synthase [Mycobacterium sp. 1137317.9]	WP_067320514
KS_021	771	RHG_cluster_354	49.219	256	1.20E-65	Type I polyketide synthase [Sciscionella marina]	WP_020497474
KS_026	1080	RHG_cluster_321	62.291	358	8.60E-171	Beta-ketoacyl-[acyl-carrier-protein] synthase II [Geobacter metallireducens]	WP_004511462
KS_028	591	RHG_cluster_221	66.495	194	8.64E-78	Type I polyketide synthase [Mycobacterium sp. YR782]	WP_108054808
KS_033	1266	RHG_cluster_087	59.674	429	1.45E-143	Type I polyketide synthase [Mycolicibacterium vanbaalenii]	WP_011778276
KS_036	792	RHG_cluster_345	62.548	259	1.08E-99	Type I polyketide synthase [Mycobacterium sp. 1137317.9]	WP_067320514
KS_037	390	RHG_cluster_351	66.667	126	3.75E-47	Type I polyketide synthase [Mycolicibacterium diernhoferi]	WP_073855454
KS_040	1038	RHG_cluster_295	58.841	345	7.23E-119	Type I polyketide synthase [Mycolicibacterium austroafricanum]	WP_051558318
KS_043	540	RHG_cluster_283	45.055	182	6.17E-38	Type I polyketide synthase [Streptomyces cattleya]	WP_014144002
KS_045	741	RHG_cluster_338	61.411	241	1.90E-90	Type I polyketide synthase [Mycolicibacterium diernhoferi]	WP_073855454
KS_046	354	RHG_cluster_362	50	118	1.33E-25	Type I polyketide synthase [Streptomyces]	WP_030626723
KS_048	1266	RHG_cluster_136	40.61	426	2.55E-83	Type I polyketide synthase [Streptomyces sp. WMMB 714]	WP_045862080
KS_052	285	RHG_cluster_367	61.111	90	6.77E-30	Type I polyketide synthase [filamentous cyanobacterium CCP3]	WP_106919617
KS_053	543	RHG_cluster_357	67.797	177	1.32E-68	Type I polyketide synthase [Mycolicibacterium diernhoferi]	WP_073855454
KS_054	525	RHG_cluster_356	67.442	172	1.46E-68	Type I polyketide synthase [Mycobacterium colombiense]	WP_065125467
KS_056	1008	RHG_cluster_355	72.321	336	0	Beta-ketoacyl-[acyl-carrier-protein] synthase II [Rhodothermus marinus]	WP_014068140
KS_060	444	RHG_cluster_183	49.664	149	1.79E-33	Type I polyketide synthase [Nocardiopsis sp. NRRL B-16309]	WP_053618745
KS_062	657	RHG_cluster_318	65.238	210	2.06E-84	Type I polyketide synthase [Mycolicibacterium diernhoferi]	WP_073855454

KS_063	1230	RHG_cluster_019	61.722	418	1.22E-149	Type I polyketide synthase [Mycolicibacterium austroafricanum]	WP_051558318
KS_065	591	RHG_cluster_255	65.464	194	1.82E-76	Type I polyketide synthase [Mycobacterium sp. YR782]	WP_108054808
KS_067	1272	RHG_cluster_124	58.629	423	4.31E-148	Type I polyketide synthase [Mycobacterium colombiense]	WP_044486300
KS_069	825	RHG_cluster_168	61.397	272	3.43E-103	Type I polyketide synthase [Mycolicibacterium diernhoferi]	WP_073855454
KS_071	1152	RHG_cluster_294	58.743	366	6.93E-127	Type I polyketide synthase [Mycobacterium sp. PYR15]	WP_096309613
KS_074	1187	RHG_cluster_151	66.667	396	0	Beta-ketoacyl-[acyl-carrier-protein] synthase II [Bryobacter aggregatus]	WP_031496688
KS_075	1136	RHG_cluster_164	61.538	351	7.49E-131	Hybrid non-ribosomal peptide synthetase/Type I polyketide synthase [Methylosinus sp. LW4]	WP_026191724
KS_077	1097	RHG_cluster_310	73.889	360	0	Beta-ketoacyl-[acyl-carrier-protein] synthase II [marine gamma proteobacterium HTCC2148]	WP_007230914
KS_080	1094	RHG_cluster_343	59.178	365	8.84E-163	Beta-ketoacyl-[acyl-carrier-protein] synthase II [Vulcanibacillus modesticaldus]	WP_069655673
KS_083	1130	RHG_cluster_336	53.439	378	2.38E-120	Type I polyketide synthase [Mycobacterium sp. UNC267MFSha1.1M11]	WP_090362897
KS_088	893	RHG_cluster_279	62.766	282	2.96E-110	Type I polyketide synthase [Mycobacterium sp. 1137317.9]	WP_067320514
KS_091	977	RHG_cluster_119	66.254	323	2.35E-129	Type I polyketide synthase [Mycobacterium sp. 1137317.9]	WP_067320514
KS_094	1097	RHG_cluster_111	43.529	340	3.24E-77	Hypothetical protein [Sandaracinus amylolyticus]	WP_053231614
KS_096	1097	RHG_cluster_116	45.507	345	1.98E-79	Hypothetical protein [Sandaracinus amylolyticus]	WP_053231614
KS_098	1100	RHG_cluster_184	44.048	252	2.58E-59	Beta-ketoacyl-[acyl-carrier-protein] synthase family protein [Acidobacteria bacterium KBS 146]	WP_026387381
KS_103	1007	RHG_cluster_365	58.333	336	2.18E-128	Beta-ketoacyl-[acyl-carrier-protein] synthase II [Roseiflexus castenholzii]	WP_041330400
KS_104	1223	RHG_cluster_007	82.02	406	0	Beta-ketoacyl-[acyl-carrier-protein] synthase II [Thioalkalivibrio sp. HK1]	WP_038098656
KS_107	1223	RHG_cluster_018	59.705	407	2.56E-177	Beta-ketoacyl-[acyl-carrier-protein] synthase II [Clostridiisalibacter paucivorans]	WP_026893881
KS_108	1217	RHG_cluster_278	95.05	404	0	Beta-ketoacyl-ACP synthase I [Rhodospirillaceae bacterium Spongia-Bin9]	WP_108794197
KS_109	1208	RHG_cluster_153	75	400	0	Beta-ketoacyl-ACP synthase I [Inquilinus limosus]	WP_026872496
KS_110	1202	RHG_cluster_073	71.859	398	0	Beta-ketoacyl-ACP synthase I [Salinicola sp. MIT1003]	WP_071230737
KS_112	1250	RHG_cluster_127	74.576	413	0	Beta-ketoacyl-[acyl-carrier-protein] synthase II [Rhodospirillales bacterium URHD0088]	WP_027300572
KS_113	1259	RHG_cluster_237	51.878	426	3.08E-126	Type I polyketide synthase [Mycobacterium colombiense]	WP_065030390
KS_130	1091	RHG_cluster_315	65.123	367	2.50E-171	Beta-ketoacyl-[acyl-carrier-protein] synthase II [Amphritea japonica]	WP_026340126
KS_131	1226	RHG_cluster_098	58.333	408	6.75E-169	Beta-ketoacyl-[acyl-carrier-protein] synthase II [Thermoflexus hugenholtzii]	WP_098504503
KS_135	1199	RHG_cluster_033	67.758	397	0	Beta-ketoacyl-[acyl-carrier-protein] synthase family protein [Rhodovulum sp. MB263]	WP_080616320
KS_137	1184	RHG_cluster_156	77.215	395	0	Beta-ketoacyl-ACP synthase I [Albidovulum inexpectatum]	WP_104068930

KS_140	1250	RHG_cluster_277	73.366	413	0	Beta-ketoacyl-[acyl-carrier-protein] synthase II [Rhodospirillales bacterium URHD0088]	WP_027300572
KS_143	1223	RHG_cluster_049	74.138	406	0	Beta-ketoacyl-[acyl-carrier-protein] synthase II [Thioalkalivibrio sp. HK1]	WP_038098656
KS_144	1223	RHG_cluster_138	59.705	407	1.90E-180	Beta-ketoacyl-[acyl-carrier-protein] synthase II [Risungbinella massiliensis]	WP_044642594
KS_148	1214	RHG_cluster_231	67.782	239	1.03E-117	Beta-ketoacyl-ACP synthase [Aeromonas sp. HMWF016]	WP_108570661
KS_151	1196	RHG_cluster_250	68.09	398	0	Beta-ketoacyl-ACP synthase I [Halomonas sp. HL-48]	WP_027337473
KS_153	1250	RHG_cluster_079	51.914	418	4.60E-142	Beta-ketoacyl-[acyl-carrier-protein] synthase family protein [Syntrophobacter sp. SbD1]	WP_106821390
KS_161	1073	RHG_cluster_292	78.492	358	0	Beta-ketoacyl-ACP synthase I [Haliea salexigens]	WP_027950912
KS_162	1202	RHG_cluster_108	67.75	400	0	Beta-ketoacyl-ACP synthase I [Methyloterricola oryzae]	WP_045223829
KS_166	1223	RHG_cluster_023	66.667	408	0	Beta-ketoacyl-[acyl-carrier-protein] synthase II [Pyrinomonas methylaliphatogenes]	WP_041976954
KS_167	1151	RHG_cluster_271	69.531	384	0	Beta-ketoacyl-[acyl-carrier-protein] synthase II [Solibacteres bacterium SbA2]	WP_105314403
KS_173	1253	RHG_cluster_326	55.66	424	1.22E-141	Type I polyketide synthase [Mycolicibacterium austroafricanum]	WP_051558318
KS_174	1229	RHG_cluster_130	58.293	410	1.23E-167	Beta-ketoacyl-[acyl-carrier-protein] synthase II [Desmospora sp. 8437]	WP_040387222
KS_175	1217	RHG_cluster_114	58.025	405	4.80E-177	Beta-ketoacyl-[acyl-carrier-protein] synthase II [Bacillales]	WP_029100165
KS_176	1265	RHG_cluster_335	56.338	426	9.28E-143	Type I polyketide synthase, partial [Mycobacterium colombiense]	WP_082282154
KS_178	1187	RHG_cluster_217	57.027	370	2.90E-128	Type I polyketide synthase [Mycobacterium sp. 852002-30065_SCH5024008]	WP_067203355
KS_181	1223	RHG_cluster_188	64.532	406	0	Beta-ketoacyl-[acyl-carrier-protein] synthase II [Moorella humiferrea]	WP_106006374
KS_182	1223	RHG_cluster_225	60.688	407	0	Beta-ketoacyl-[acyl-carrier-protein] synthase II [Kroppenstedtia eburnea]	WP_076523249
KS_186	1280	RHG_cluster_078	43.972	423	2.44E-119	Hypothetical protein [Syntrophobacter sp. SbD1]	WP_106821393
KS_188	1217	RHG_cluster_009	64.109	404	0	Beta-ketoacyl-[acyl-carrier-protein] synthase II [Pseudomonas pachastrellae]	WP_083725910
KS_189	1196	RHG_cluster_334	65.163	399	0	Beta-ketoacyl-[acyl-carrier-protein] synthase II [Dyella]	WP_026634449
KS_191	1226	RHG_cluster_142	61.916	407	0	Beta-ketoacyl-[acyl-carrier-protein] synthase II [Pseudomonas pachastrellae]	WP_083725910
KS_193	1091	RHG_cluster_359	66.027	365	0	Beta-ketoacyl-[acyl-carrier-protein] synthase II [Sulfuricaulis limicola]	WP_096361882
KS_195	1163	RHG_cluster_122	62.304	382	1.50E-179	Beta-ketoacyl-[acyl-carrier-protein] synthase II [Pseudomonas pachastrellae]	WP_083725910
KS_197	1226	RHG_cluster_305	61.52	408	1.94E-179	Beta-ketoacyl-[acyl-carrier-protein] synthase II [Nitrosomonas europaea]	WP_011112207
KS_202	1223	RHG_cluster_344	64.491	383	0	Beta-ketoacyl-[acyl-carrier-protein] synthase II [Sulfuricaulis limicola]	WP_096361882
KS_203	1217	RHG_cluster_235	66.502	406	0	Beta-ketoacyl-[acyl-carrier-protein] synthase II [Thioflavicoccus mobilis]	WP_015281247

KS_205	1064	RHG_cluster_120	69.057	265	2.37E-130	Beta-ketoacyl-ACP synthase [Cupriavidus oxalaticus]	WP_063237600
KS_218	1136	RHG_cluster_080	34.072	361	3.96E-45	Hypothetical protein [Nitrospirae bacterium HCH-1]	WP_085053639
KS_220	1223	RHG_cluster_274	67.885	383	0	Beta-ketoacyl-[acyl-carrier-protein] synthase II [Oceanicoccus sagamiensis]	WP_085759718
KS_223	1223	RHG_cluster_012	71.253	407	0	Beta-ketoacyl-[acyl-carrier-protein] synthase II [Ectothiorhodospira haloalkaliphila]	WP_025281708
KS_224	1259	RHG_cluster_296	56.235	425	2.55E-142	Type I polyketide synthase [Mycolicibacterium austroafricanum]	WP_051558318
KS_225	1223	RHG_cluster_319	64.691	405	0	Beta-ketoacyl-[acyl-carrier-protein] synthase II [Pyrinomonas methylaliphatogenes]	WP_041976954
KS_235	1217	RHG_cluster_324	56.724	409	8.77E-172	Beta-ketoacyl-[acyl-carrier-protein] synthase II [Candidatus Desulfofervidus auxilii]	WP_066064784
KS_236	1223	RHG_cluster_347	67.734	406	0	Beta-ketoacyl-[acyl-carrier-protein] synthase II [Halomonas sp. A11-A]	WP_110068027
KS_237	1223	RHG_cluster_281	69.608	408	0	Beta-ketoacyl-[acyl-carrier-protein] synthase II [Solibacteres bacterium SbA2]	WP_105314403
KS_240	1157	RHG_cluster_051	54.45	382	4.06E-134	Beta-ketoacyl-ACP synthase II [Ferrimicrobium acidiphilum]	WP_052566261
KS_244	1220	RHG_cluster_263	68.227	406	0	Beta-ketoacyl-[acyl-carrier-protein] synthase II [Methylomagnum ishizawai]	WP_085216214
KS_249	1226	RHG_cluster_220	61.916	407	0	Beta-ketoacyl-[acyl-carrier-protein] synthase II [Pseudomonas pachastrellae]	WP_083725910
KS_252	1292	RHG_cluster_011	44.749	438	3.54E-94	Type I polyketide synthase [Sciscionella marina]	WP_020497474
KS_254	1226	RHG_cluster_301	61.029	408	0	Beta-ketoacyl-[acyl-carrier-protein] synthase II [Aneurinibacillus terranovensis]	WP_027414564
KS_256	1229	RHG_cluster_039	56.829	410	1.52E-163	Beta-ketoacyl-[acyl-carrier-protein] synthase II [Rhodothermus marinus]	WP_012845172
KS_259	1220	RHG_cluster_148	53.35	403	6.60E-137	Beta-ketoacyl-[acyl-carrier-protein] synthase family protein [Nibricoccus aquaticus]	WP_096057284
KS_261	1223	RHG_cluster_035	74.384	406	0	Beta-ketoacyl-[acyl-carrier-protein] synthase II [Thioalkalivibrio sulfidiphilus]	WP_026289556
KS_264	1214	RHG_cluster_227	63.366	404	0	Beta-ketoacyl-[acyl-carrier-protein] synthase II [Thermoanaerobaculum aquaticum]	WP_038046901
KS_267	1292	RHG_cluster_028	44.749	438	4.91E-94	Type I polyketide synthase [Sciscionella marina]	WP_020497474
KS_269	1151	RHG_cluster_212	58.377	382	1.34E-141	Beta-ketoacyl-ACP synthase II [Streptomyces sp. NRRL F-2664]	WP_030765373
KS_270	1229	RHG_cluster_185	62.347	409	0	Beta-ketoacyl-[acyl-carrier-protein] synthase II [Nitrosomonas aestuarii]	WP_090702733
KS_278	1226	RHG_cluster_020	60.294	408	0	Beta-ketoacyl-[acyl-carrier-protein] synthase II [Vulcanibacillus modesticaldus]	WP_069655673
KS_285	1280	RHG_cluster_081	55.556	423	1.73E-163	Beta-ketoacyl-[acyl-carrier-protein] synthase family protein [Syntrophobacter sp. SbD1]	WP_106821387
KS_290	1292	RHG_cluster_091	41.136	440	4.86E-87	Type I polyketide synthase [Crossiella equi]	WP_086785133
KS_292	1292	RHG_cluster_096	44.266	436	9.46E-99	Type I polyketide synthase [Sciscionella marina]	WP_020497474
KS_295	1286	RHG_cluster_094	63.593	423	4.29E-180	Type I polyketide synthase [Marinobacterium profundum]	WP_067295857

KS_296	1229	RHG_cluster_244	55.774	407	6.45E-159	Beta-ketoacyl-[acyl-carrier-protein] synthase II [Desulfotomaculum acetoxidans]	WP_015756782
KS_298	1259	RHG_cluster_253	60.143	419	7.55E-178	Beta-ketoacyl-[acyl-carrier-protein] synthase family protein [Syntrophus aciditrophicus]	WP_011418884
KS_299	1160	RHG_cluster_273	61.96	347	3.94E-133	Beta-ketoacyl-ACP synthase II [Streptomyces katrae]	WP_045950542
KS_300	1226	RHG_cluster_075	63.391	407	0	Beta-ketoacyl-[acyl-carrier-protein] synthase II [Nitrospina gracilis]	WP_042251114
KS_304	1154	RHG_cluster_045	53.786	383	2.20E-131	Hypothetical protein [Tropicibacter phthalicicus]	WP_099241578
KS_313	1226	RHG_cluster_178	61.671	407	0	Beta-ketoacyl-[acyl-carrier-protein] synthase II [Geobacter metallireducens]	WP_004511462
KS_316	1217	RHG_cluster_264	54.208	404	6.71E-152	Beta-ketoacyl-[acyl-carrier-protein] synthase II [Thermorudis peleae]	WP_038037697
KS_318	1214	RHG_cluster_030	55.941	404	9.82E-156	Beta-ketoacyl-[acyl-carrier-protein] synthase II [Thermorudis peleae]	WP_038037697
KS_319	1160	RHG_cluster_259	60.519	347	9.63E-131	Beta-ketoacyl-ACP synthase II [Streptomyces katrae]	WP_045950542
KS_320	1286	RHG_cluster_215	53.103	435	2.46E-137	Type I polyketide synthase, partial [Burkholderia vietnamiensis]	WP_059694304
KS_324	1223	RHG_cluster_313	60.197	407	0	Beta-ketoacyl-[acyl-carrier-protein] synthase II [Kroppenstedtia eburnea]	WP_076523249
KS_328	1154	RHG_cluster_133	51.558	353	4.76E-110	Hypothetical protein [Tropicibacter phthalicicus]	WP_099241578
KS_331	1160	RHG_cluster_085	62.402	383	7.80E-149	Beta-ketoacyl-ACP synthase II [Streptomyces katrae]	WP_045950542
KS_333	1286	RHG_cluster_165	53.333	435	2.05E-138	Type I polyketide synthase, partial [Burkholderia vietnamiensis]	WP_059694304
KS_335	1298	RHG_cluster_302	42.759	435	3.75E-87	Type I polyketide synthase [Streptomyces]	WP_030626723
KS_343	1229	RHG_cluster_222	57.073	410	1.21E-169	Beta-ketoacyl-[acyl-carrier-protein] synthase II [Selenihalanaerobacter shriftii]	WP_078809651
KS_344	1292	RHG_cluster_016	42.759	435	4.48E-89	Type I polyketide synthase [Streptomyces]	WP_028443132
KS_347	1292	RHG_cluster_034	39.723	433	5.04E-80	Type I polyketide synthase [Burkholderia thailandensis]	WP_038712449
KS_348	1226	RHG_cluster_348	60.539	408	2.96E-168	Beta-ketoacyl-[acyl-carrier-protein] synthase II [Thermoflexus hugenholtzii]	WP_098502948
KS_352	1229	RHG_cluster_004	58.435	409	2.77E-164	Beta-ketoacyl-[acyl-carrier-protein] synthase II [Desulfovirgula thermocuniculi]	WP_027718279
KS_355	1154	RHG_cluster_236	58.398	387	1.23E-128	Beta-ketoacyl-ACP synthase II [Ilumatobacter nonamiensis]	WP_040493091
KS_359	1292	RHG_cluster_206	42.308	442	4.00E-89	Type I polyketide synthase [Crossiella equi]	WP_086785133
KS_365	1220	RHG_cluster_031	73.267	404	0	Beta-ketoacyl-[acyl-carrier-protein] synthase II [Candidatus Nitrospira nitrosa]	WP_090746581
KS_367	1229	RHG_cluster_084	56.968	409	1.29E-157	Beta-ketoacyl-[acyl-carrier-protein] synthase II [Desulfovirgula thermocuniculi]	WP_027718279
KS_374	1184	RHG_cluster_360	55.584	394	9.43E-150	Beta-ketoacyl-[acyl-carrier-protein] synthase II [Thermaerobacter subterraneus]	WP_006905000
KS_377	1229	RHG_cluster_150	57.843	408	8.43E-164	Beta-ketoacyl-[acyl-carrier-protein] synthase II [Thermoflexus hugenholtzii]	WP_098502948
KS_380	1292	RHG_cluster_135	42.069	435	4.46E-85	Type I polyketide synthase, partial [Streptomyces sp. SM5]	WP_103492552

KS_387	1292	RHG_cluster_067	41.609	435	1.97E-87	Type I polyketide synthase [Streptomyces]	WP_028443132
KS_388	1229	RHG_cluster_317	55.746	409	3.04E-153	Beta-ketoacyl-[acyl-carrier-protein] synthase II [Thermaerobacter subterraneus]	WP_006905000
KS_390	1223	RHG_cluster_172	61.084	406	0	Beta-ketoacyl-[acyl-carrier-protein] synthase II [Melghirimyces profundicolus]	WP_108022554
KS_392	1247	RHG_cluster_097	69.976	413	0	Beta-ketoacyl-[acyl-carrier-protein] synthase II [Rhodospirillales bacterium URHD0088]	WP_027300572
KS_396	1292	RHG_cluster_269	42.857	434	4.35E-87	Type I polyketide synthase [Streptomyces rubellomurinus]	WP_078860657
KS_397	1292	RHG_cluster_202	40.138	436	1.29E-90	SDR family NAD(P)-dependent oxidoreductase [Leucothrix sp. IMCC9719]	WP_109824032
KS_398	1286	RHG_cluster_167	54.398	432	6.98E-141	Type I polyketide synthase, partial [Burkholderia vietnamiensis]	WP_059895285
KS_400	1184	RHG_cluster_132	62.5	392	4.71E-169	Beta-ketoacyl-[acyl-carrier-protein] synthase family protein [Tropicibacter phthalicicus]	WP_099241574
KS_406	1184	RHG_cluster_044	63.265	392	5.45E-171	Beta-ketoacyl-[acyl-carrier-protein] synthase family protein [Tropicibacter phthalicicus]	WP_099241574
KS_408	1292	RHG_cluster_128	41.685	451	1.64E-92	Type I polyketide synthase [Sciscionella marina]	WP_020497474
KS_410	1286	RHG_cluster_037	56.744	430	2.23E-139	Type I polyketide synthase [Mycolicibacterium austroafricanum]	WP_105389463
KS_411	1223	RHG_cluster_214	58.088	408	3.16E-168	Beta-ketoacyl-[acyl-carrier-protein] synthase II [Thermoflexus hugenholtzii]	WP_098502948
KS_429	1292	RHG_cluster_040	57.009	428	1.06E-141	Type I polyketide synthase [Mycolicibacterium austroafricanum]	WP_105389463
KS_430	1292	RHG_cluster_109	40.732	437	8.44E-87	Type I polyketide synthase [Sciscionella marina]	WP_020497474
KS_437	1235	RHG_cluster_077	57.421	411	3.82E-171	Beta-ketoacyl-[acyl-carrier-protein] synthase II [Desmospora sp. 8437]	WP_040387222
KS_439	1211	RHG_cluster_086	73.367	398	0	Beta-ketoacyl-[acyl-carrier-protein] synthase II [Rhodospirillales bacterium URHD0088]	WP_027300572
KS_446	1289	RHG_cluster_069	52.914	429	1.34E-136	Type I polyketide synthase [Mycobacterium colombiense]	WP_007770214
KS_448	1292	RHG_cluster_209	40.138	436	4.26E-89	SDR family NAD(P)-dependent oxidoreductase [Leucothrix sp. IMCC9719]	WP_109824032
KS_449	1160	RHG_cluster_241	70.284	387	0	Beta-ketoacyl-ACP synthase I [Methylosarcina lacus]	WP_024296926
KS_452	1286	RHG_cluster_249	53.364	431	2.10E-138	Hybrid non-ribosomal peptide synthetase/Type I polyketide synthase [Burkholderia vietnamiensis]	WP_059740130
KS_453	1286	RHG_cluster_174	53.828	431	2.74E-130	Type I polyketide synthase, partial [Mycobacterium parascrofulaceum]	WP_007169537
KS_459	1184	RHG_cluster_218	73.59	390	0	Beta-ketoacyl-[acyl-carrier-protein] synthase II [Thioalkalivibrio sp. HK1]	WP_038098656
KS_460	1145	RHG_cluster_074	57.937	378	4.72E-130	Beta-ketoacyl-[acyl-carrier-protein] synthase family protein [Luteibacter yeojuensis]	WP_084621559
KS_462	1190	RHG_cluster_143	69.424	399	0	Beta-ketoacyl-[acyl-carrier-protein] synthase II [Pararhodobacter aggregans]	WP_107749575
KS_468	1175	RHG_cluster_068	81.538	390	0	Beta-ketoacyl-[acyl-carrier-protein] synthase II [Thioalkalivibrio sp. HK1]	WP_038098656
KS_469	1286	RHG_cluster_100	54.63	432	4.20E-140	Type I polyketide synthase, partial [Burkholderia vietnamiensis]	WP_059895285

KS_471	1175	RHG_cluster_144	80	390	0	Beta-ketoacyl-[acyl-carrier-protein] synthase II [Thioalkalivibrio sp. HK1]	WP_038098656
KS_474	1211	RHG_cluster_216	73.869	398	0	Beta-ketoacyl-[acyl-carrier-protein] synthase II [Telmatospirillum siberiense]	WP_101252614
KS_476	1133	RHG_cluster_061	43.343	353	2.63E-80	Beta-ketoacyl-ACP synthase I [Shewanella amazonensis]	WP_011760267
KS_481	1154	RHG_cluster_303	72.063	383	0	Beta-ketoacyl-ACP synthase I [Thermopetrobacter sp. TC1]	WP_038035251
KS_484	1298	RHG_cluster_298	55.787	432	2.91E-139	Type I polyketide synthase [Mycobacterium malmoense]	WP_071509339
KS_486	1112	RHG_cluster_059	62.216	352	1.20E-134	Beta-ketoacyl-ACP synthase II [Ilumatobacter nonamiensis]	WP_040493091
KS_490	1292	RHG_cluster_213	42.793	444	5.36E-99	Type I polyketide synthase [Sciscionella marina]	WP_020497474
KS_491	1286	RHG_cluster_154	51.981	429	7.51E-134	Type I polyketide synthase [Mycobacterium palustre]	WP_085077785
KS_494	1292	RHG_cluster_072	42.045	440	6.20E-91	Type I polyketide synthase [Sciscionella marina]	WP_020497474
KS_495	1292	RHG_cluster_272	44.091	440	1.36E-93	Type I polyketide synthase [Sciscionella marina]	WP_020497474
KS_497	1286	RHG_cluster_163	54.545	429	2.10E-139	Hybrid non-ribosomal peptide synthetase/Type I polyketide synthase [Burkholderia vietnamiensis]	WP_059454702
KS_498	1286	RHG_cluster_322	53.704	432	2.55E-138	Type I polyketide synthase, partial [Burkholderia vietnamiensis]	WP_059694304
KS_499	1094	RHG_cluster_265	73.973	365	0	Beta-ketoacyl-[acyl-carrier-protein] synthase II [Rhodothermus marinus]	WP_014068140
KS_501	1286	RHG_cluster_204	54.419	430	4.52E-141	Hybrid non-ribosomal peptide synthetase/Type I polyketide synthase [Burkholderia vietnamiensis]	WP_069223642
KS_506	1286	RHG_cluster_179	59.674	429	2.77E-148	Type I polyketide synthase [Mycolicibacterium austroafricanum]	WP_105389463
KS_507	1286	RHG_cluster_057	52.727	440	3.19E-138	Type I polyketide synthase [Mycobacterium sp. IS-1590]	WP_064404744
KS_511	1292	RHG_cluster_090	54.63	432	1.07E-138	Type I polyketide synthase [Mycolicibacterium komanii]	WP_090277733
KS_516	1292	RHG_cluster_162	54.419	430	1.43E-140	Type I polyketide synthase, partial [Burkholderia vietnamiensis]	WP_059668134
KS_517	1220	RHG_cluster_155	63.027	403	0	Beta-ketoacyl-[acyl-carrier-protein] synthase II [Albimonas donghaensis]	WP_092679786
KS_519	1184	RHG_cluster_065	63.265	392	0	Beta-ketoacyl-[acyl-carrier-protein] synthase II [Thermodesulfovibrio sp. N1]	WP_068860060
KS_520	1286	RHG_cluster_195	52.448	429	6.01E-136	Type I polyketide synthase, partial [Burkholderia vietnamiensis]	WP_059567396
KS_525	1286	RHG_cluster_192	54.312	429	1.21E-143	Type I polyketide synthase, partial [Burkholderia vietnamiensis]	WP_059567396
KS_526	1148	RHG_cluster_103	64.491	383	0	Beta-ketoacyl-[acyl-carrier-protein] synthase II [Chromatiaceae bacterium 2141T.STBD.0c.01a]	WP_078119642
KS_529	1292	RHG_cluster_194	52.214	429	1.95E-134	Hybrid non-ribosomal peptide synthetase/Type I polyketide synthase [Burkholderia vietnamiensis]	WP_059603382
KS_532	1286	RHG_cluster_266	57.674	430	3.20E-151	Type I polyketide synthase [Mycolicibacterium diernhoferi]	WP_073855454
KS_538	1286	RHG_cluster_160	53.756	426	2.06E-137	Type I polyketide synthase [Mycolicibacterium austroafricanum]	WP_105389463
KS_541	1286	RHG_cluster_027	54.312	429	5.27E-139	Type I polyketide synthase, partial [Burkholderia vietnamiensis]	WP_059567396
KS_543	1286	RHG_cluster_054	53.132	431	3.70E-136	Type I polyketide synthase [Mycolicibacterium rhodesiae]	WP_005142704

KS_545	1292	RHG_cluster_242	53.846	429	5.50E-140	Hybrid non-ribosomal peptide synthetase/Type I polyketide synthase [Burkholderia vietnamiensis]	WP_059740130
KS_548	1277	RHG_cluster_159	62.441	426	4.99E-160	Type I polyketide synthase [Mycolicibacterium vanbaalenii]	WP_011778276
KS_551	1292	RHG_cluster_147	53.613	429	2.01E-140	Type I polyketide synthase, partial [Burkholderia vietnamiensis]	WP_059567396
KS_553	1250	RHG_cluster_053	60.28	428	4.17E-151	Type I polyketide synthase [Mycolicibacterium austroafricanum]	WP_105389463
KS_554	1283	RHG_cluster_008	60.047	428	3.16E-150	Type I polyketide synthase [Mycolicibacterium austroafricanum]	WP_051558318
KS_555	1286	RHG_cluster_177	55.349	430	2.54E-141	Type I polyketide synthase [Mycolicibacterium tusciae]	WP_006241768
KS_559	1301	RHG_cluster_285	58.837	430	6.44E-146	Type I polyketide synthase [Mycolicibacterium austroafricanum]	WP_105389463
KS_569	1292	RHG_cluster_238	52.995	434	4.16E-135	Type I polyketide synthase, partial [Burkholderia vietnamiensis]	WP_059694304
KS_570	1073	RHG_cluster_095	53.067	326	4.62E-111	Beta-ketoacyl-[acyl-carrier-protein] synthase family protein [Rhizobiales bacterium]	WP_109794138
KS_572	1286	RHG_cluster_288	52.668	431	3.25E-137	Hybrid non-ribosomal peptide synthetase/Type I polyketide synthase [Burkholderia vietnamiensis]	WP_059991443
KS_573	1292	RHG_cluster_056	52.315	432	5.03E-137	Type I polyketide synthase, partial [Burkholderia vietnamiensis]	WP_059694304
KS_575	1067	RHG_cluster_232	60.596	302	3.34E-118	Beta-ketoacyl-ACP synthase [Xanthomonas floridensis]	WP_064508482
KS_577	1178	RHG_cluster_046	61.675	394	2.00E-156	Hypothetical protein [Verminephrobacter aporrectodeae]	WP_081479930
KS_581	1010	RHG_cluster_121	53.266	199	2.08E-62	Beta-ketoacyl-ACP synthase [Cellvibrio sp. pealriver]	WP_082191725
KS_583	1184	RHG_cluster_134	59.836	366	1.79E-136	Hypothetical protein [Verminephrobacter aporrectodeae]	WP_081479930
KS_588	1016	RHG_cluster_125	67.755	245	9.71E-107	Beta-ketoacyl-ACP synthase [Thiobacillus denitrificans]	WP_059752414
KS_593	1283	RHG_cluster_306	60.941	425	5.36E-159	Type I polyketide synthase [Mycobacterium sp. EPa45]	WP_047332984
KS_596	1259	RHG_cluster_261	53.63	427	6.14E-129	Type I polyketide synthase [Mycobacterium gordonae]	WP_065161832
KS_599	1079	RHG_cluster_182	59.259	324	1.94E-133	Beta-ketoacyl synthase [Pseudomonas flexibilis]	WP_039561123
KS_603	851	RHG_cluster_270	70.204	245	3.12E-119	Beta-ketoacyl synthase [Vitiosangium sp. GDMCC 1.1324]	WP_108066665
KS_614	1259	RHG_cluster_262	54.846	423	1.02E-140	Type I polyketide synthase [Mycobacterium asiaticum]	WP_065157110
KS_621	1259	RHG_cluster_208	56.44	427	2.21E-141	Type I polyketide synthase [Mycolicibacterium tusciae]	WP_006241768
KS_625	1262	RHG_cluster_289	55.189	424	4.52E-136	Type I polyketide synthase [Mycobacterium sp. 852002-53434_SCH5985345]	WP_066952779
KS_627	1265	RHG_cluster_162	57.412	425	6.81E-145	Type I polyketide synthase [Mycolicibacterium austroafricanum]	WP_051558318
KS_628	1277	RHG_cluster_248	55.245	429	2.64E-140	Type I polyketide synthase [Mycolicibacterium austroafricanum]	WP_105389463
KS_636	1262	RHG_cluster_119	61.412	425	3.76E-157	Type I polyketide synthase [Mycolicibacterium austroafricanum]	WP_105389463
KS_638	1259	RHG_cluster_226	54.61	423	5.54E-132	Type I polyketide synthase [Mycobacterium palustre]	WP_085078235
KS_642	713	RHG_cluster_140	71.795	234	7.44E-100	Type I polyketide synthase [Mycobacterium malmoense]	WP_065517313
KS_643	1256	RHG_cluster_233	61.647	425	2.67E-156	Type I polyketide synthase [Mycolicibacterium austroafricanum]	WP_105389463
KS_647	1256	RHG_cluster_229	58.156	423	3.22E-141	Type I polyketide synthase [Mycolicibacterium austroafricanum]	WP_051558318

KS_650	1235	RHG_cluster_191	58.095	420	2.17E-137	Type I polyketide synthase [Mycolicibacterium austroafricanum]	WP_105389463
KS_652	1259	RHG_cluster_268	55.189	424	1.12E-137	Type I polyketide synthase [Mycobacterium sp. GA-1331]	WP_064424921
KS_655	1244	RHG_cluster_203	57.569	436	3.25E-145	Type I polyketide synthase [Mycolicibacterium austroafricanum]	WP_105389463
KS_657	1259	RHG_cluster_140	60.142	424	6.00E-151	Type I polyketide synthase [Mycolicibacterium austroafricanum]	WP_105389463
KS_660	1259	RHG_cluster_197	54.907	428	7.98E-140	Type I polyketide synthase [Mycobacterium gordonae]	WP_065161832
KS_662	1265	RHG_cluster_062	55.529	425	3.02E-140	Type I polyketide synthase [Mycobacterium parmense]	WP_085270770
KS_664	1277	RHG_cluster_145	58.156	423	1.10E-144	Type I polyketide synthase [Mycobacterium sp. IS-1744]	WP_064399965
KS_666	1265	RHG_cluster_246	57.412	425	5.89E-145	Type I polyketide synthase [Mycobacterium asiaticum]	WP_065120279
KS_668	1259	RHG_cluster_240	51.415	424	1.04E-143	Type I polyketide synthase [Pleurocapsa minor]	WP_015144800
KS_670	1259	RHG_cluster_014	56.206	427	4.81E-142	Type I polyketide synthase [Mycobacterium gordonae]	WP_065044175
KS_672	1262	RHG_cluster_198	53.505	428	1.83E-130	Type I polyketide synthase [Mycolicibacterium vanbaalenii]	WP_011778276
KS_673	1268	RHG_cluster_119	62.411	423	3.97E-160	Type I polyketide synthase [Mycolicibacterium austroafricanum]	WP_105389463
KS_677	1259	RHG_cluster_149	56.471	425	8.66E-139	Type I polyketide synthase [Mycolicibacterium vanbaalenii]	WP_011778276
KS_679	1253	RHG_cluster_053	52.927	427	8.13E-130	Type I polyketide synthase [Mycobacterium colombiense]	WP_077092494
KS_682	1277	RHG_cluster_140	60.78	436	8.34E-157	Type I polyketide synthase [Mycolicibacterium austroafricanum]	WP_105389463
KS_685	1268	RHG_cluster_140	60.757	423	2.61E-154	Type I polyketide synthase [Mycobacterium sp. TBL 1200985]	WP_085324847
KS_689	1259	RHG_cluster_161	54.717	424	1.34E-138	Type I polyketide synthase [Mycobacterium sp. 1245111.1]	WP_067331050
KS_691	1259	RHG_cluster_164	56	425	4.66E-136	Type I polyketide synthase [Mycolicibacterium austroafricanum]	WP_105389463
KS_692	1259	RHG_cluster_124	56.706	425	4.38E-141	Type I polyketide synthase [Mycolicibacterium austroafricanum]	WP_105389463
KS_693	1259	RHG_cluster_037	55.972	427	4.19E-143	Type I polyketide synthase [Mycobacterium parmense]	WP_085270770
KS_697	1265	RHG_cluster_165	58.156	423	2.13E-143	Type I polyketide synthase [Mycolicibacterium austroafricanum]	WP_105389463
KS_700	1271	RHG_cluster_019	60.757	423	3.73E-153	Type I polyketide synthase [Mycobacterium asiaticum]	WP_065146316
KS_704	1262	RHG_cluster_154	53.901	423	6.42E-139	Type I polyketide synthase [Mycolicibacterium austroafricanum]	WP_051558318
KS_707	1262	RHG_cluster_069	54.439	428	3.32E-139	Type I polyketide synthase [Mycolicibacterium bacteremicum]	WP_083056565
KS_712	1259	RHG_cluster_100	57.309	431	5.25E-142	Type I polyketide synthase [Mycolicibacterium vanbaalenii]	WP_011778276
KS_714	1256	RHG_cluster_040	52.358	424	1.97E-134	Type I polyketide synthase [Mycolicibacterium austroafricanum]	WP_051558318
KS_715	1259	RHG_cluster_090	53.774	424	4.02E-137	Type I polyketide synthase [Mycobacterium colombiense]	WP_077092494
KS_719	1259	RHG_cluster_056	54.588	425	7.94E-135	Type I polyketide synthase [Mycobacterium avium]	WP_062889895
KS_723	1256	RHG_cluster_054	55.869	426	8.93E-140	Hybrid non-ribosomal peptide synthetase/Type I polyketide synthase [Burkholderia sp. KJ006]	WP_014725946
KS_724	1259	RHG_cluster_057	57.176	425	4.59E-142	Type I polyketide synthase [Mycolicibacterium austroafricanum]	WP_105389463
KS_727	1259	RHG_cluster_179	56	425	4.08E-135	Type I polyketide synthase [Mycolicibacterium austroafricanum]	WP_105389463

KS_729	1265	RHG_cluster_027	56.132	424	1.84E-134	Type I polyketide synthase [Mycolicibacterium austroafricanum]	WP_105389463
KS_733	1262	RHG_cluster_147	54.93	426	1.37E-138	Type I polyketide synthase [Mycolicibacterium austroafricanum]	WP_051558318
KS_735	1124	RHG_cluster_194	54.569	383	5.67E-122	Type I polyketide synthase [Mycolicibacterium austroafricanum]	WP_105389463
KS_738	1277	RHG_cluster_159	61.982	434	4.74E-161	Type I polyketide synthase [Mycolicibacterium austroafricanum]	WP_105389463
KS_741	1259	RHG_cluster_008	55.529	425	4.25E-142	Type I polyketide synthase [Mycobacterium colombiense]	WP_076099902
KS_744	1262	RHG_cluster_087	51.288	427	1.14E-126	Hybrid non-ribosomal peptide synthetase/Type I polyketide synthase [Burkholderia sp. KJ006]	WP_014725946
KS_746	1274	RHG_cluster_177	56.206	427	9.85E-136	Type I polyketide synthase [Mycolicibacterium vanbaalenii]	WP_011778276
KS_754	1262	RHG_cluster_193	61.939	423	5.53E-157	Type I polyketide synthase [Mycolicibacterium austroafricanum]	WP_105389463
KS_758	1250	RHG_cluster_119	59.155	426	6.74E-151	Type I polyketide synthase [Mycolicibacterium austroafricanum]	WP_105389463
KS_759	1268	RHG_cluster_019	60.748	428	7.57E-152	Type I polyketide synthase [Mycolicibacterium austroafricanum]	WP_105389463

Table S13. BLAST search of the KS sequences from the metagenome of *Speciospongia* sp. against refseq_protein database in NCBI

KS ID	Length (nt)	BGC ID	Identity (%)	Alignment length (aa)	E-value	The homologenus sequences	Accession number
KS_002	1086	SPV_cluster_403	81.844	358	0	Beta-ketoacyl-[acyl-carrier-protein] synthase II [Thioalkalivibrio sp. HK1]	WP_038098656
KS_003	459	SPV_cluster_447	64	150	2.39E-56	Type I polyketide synthase [Mycobacterium shimoidei]	WP_069394598
KS_005	1266	SPV_cluster_194	42.118	425	4.08E-85	Type I polyketide synthase [Streptomyces rubellomurinus]	WP_078860657
KS_008	1266	SPV_cluster_115	59.294	425	1.35E-143	Type I polyketide synthase [Mycobacterium celatum]	WP_062540382
KS_009	663	SPV_cluster_191	66.507	209	5.00E-85	Type I polyketide synthase [Mycobacterium sp. 1137317.9]	WP_067320514
KS_015	435	SPV_cluster_422	64.336	143	1.01E-54	Type I polyketide synthase, partial [Mycobacterium avium]	WP_062887549
KS_017	696	SPV_cluster_454	46.789	218	2.30E-53	Polyketide synthase [Verrucomicrobium spinosum]	WP_075088152
KS_018	1266	SPV_cluster_241	59.207	429	1.10E-142	Type I polyketide synthase [Mycolicibacterium vanbaalenii]	WP_011778276
KS_022	783	SPV_cluster_440	64.341	258	2.44E-99	Type I polyketide synthase [Mycolicibacterium austroafricanum]	WP_105389463
KS_023	297	SPV_cluster_287	58.333	96	7.82E-28	Type I polyketide synthase [Mycobacterium conspicuum]	WP_085233715
KS_024	945	SPV_cluster_405	58.741	286	9.94E-105	Type I polyketide synthase [Mycobacterium sp. 1137317.9]	WP_067320514
KS_025	1221	SPV_cluster_202	56.618	408	1.94E-135	Type I polyketide synthase, partial [Mycobacteroides abscessus]	WP_079625060
KS_027	351	SPV_cluster_456	62.281	114	1.04E-39	Type I polyketide synthase [filamentous cyanobacterium CCP3]	WP_106919617
KS_031	744	SPV_cluster_302	63.415	246	8.08E-94	Type I polyketide synthase [Mycolicibacterium austroafricanum]	WP_105389463
KS_034	1077	SPV_cluster_360	60.446	359	6.34E-128	Type I polyketide synthase [Mycobacterium celatum]	WP_062540382
KS_035	1014	SPV_cluster_461	67.16	338	3.55E-165	Beta-ketoacyl-[acyl-carrier-protein] synthase II [Balneatrix alpica]	WP_035461346
KS_038	1113	SPV_cluster_096	61.141	368	1.35E-137	Type I polyketide synthase [Mycolicibacterium vanbaalenii]	WP_011778276
KS_042	669	SPV_cluster_382	68.868	212	1.26E-90	Type I polyketide synthase [Mycobacterium colombiense]	WP_075236679
KS_047	1113	SPV_cluster_418	57.221	367	1.50E-128	Type I polyketide synthase [Mycolicibacterium austroafricanum]	WP_105389463
KS_049	438	SPV_cluster_450	61.538	143	1.67E-52	Type I polyketide synthase [Mycolicibacterium diernhoferi]	WP_073855454
KS_050	279	SPV_cluster_446	61.538	91	1.02E-29	Type I polyketide synthase [Nostocales cyanobacterium HT-58-2]	WP_087541150
KS_051	531	SPV_cluster_451	62.573	171	1.59E-64	Type I polyketide synthase [Mycobacterium conspicuum]	WP_085235287
KS_055	312	SPV_cluster_391	47.475	99	6.47E-20	Type I polyketide synthase, partial [Actinomadura meyerae]	WP_089329605
KS_057	849	SPV_cluster_378	61.232	276	4.28E-103	Type I polyketide synthase [Mycobacterium]	WP_029121205
KS_058	954	SPV_cluster_384	58.621	319	5.36E-110	Type I polyketide synthase [Mycobacterium]	WP_036463411
KS_059	570	SPV_cluster_455	63.978	186	5.16E-68	Type I polyketide synthase [Mycobacterium palustre]	WP_085077785
KS_068	888	SPV_cluster_333	59.498	279	1.94E-101	Type I polyketide synthase, partial [Burkholderia vietnamiensis]	WP_059895285
KS_070	1023	SPV_cluster_408	68.373	332	3.12E-170	Beta-ketoacyl synthase [marine gamma proteobacterium HTCC2148]	WP_007229230
KS_072	912	SPV_cluster_428	66.784	283	6.20E-115	Type I polyketide synthase [Mycolicibacterium austroafricanum]	WP_105389463

KS_076	713	SPV_cluster_204	48.182	220	8.63E-55	Type I polyketide synthase [Chondromyces crocatus]	WP_063796357
KS_081	1118	SPV_cluster_373	58.839	379	1.05E-129	Type I polyketide synthase [Mycolicibacterium vanbaalenii]	WP_011778276
KS_082	1166	SPV_cluster_273	59.452	365	1.00E-129	Type I polyketide synthase [Mycolicibacterium austroafricanum]	WP_051558318
KS_085	1112	SPV_cluster_396	56.757	370	1.60E-127	Type I polyketide synthase [Mycobacterium sp. 852002-30065_SCH5024008]	WP_067203355
KS_086	671	SPV_cluster_323	61.972	213	2.42E-82	Type I polyketide synthase [Mycobacterium asiaticum]	WP_065157110
KS_087	1097	SPV_cluster_425	55.342	365	4.57E-126	Type I polyketide synthase, partial [Burkholderia vietnamiensis]	WP_059668134
KS_089	929	SPV_cluster_347	67.491	283	2.30E-116	Type I polyketide synthase [Mycolicibacterium vanbaalenii]	WP_011778276
KS_090	398	SPV_cluster_386	53.383	133	6.15E-32	Type I polyketide synthase [Nocardiopsis sp. NRRL B-16309]	WP_053618745
KS_092	1133	SPV_cluster_423	60.212	377	2.59E-168	Beta-ketoacyl-[acyl-carrier-protein] synthase II [Dehalobacterium formicoaceticum]	WP_089609693
KS_093	1085	SPV_cluster_279	69.806	361	2.22E-177	Beta-ketoacyl-[acyl-carrier-protein] synthase II [Solibacteres bacterium SbA2]	WP_105314403
KS_097	914	SPV_cluster_292	58.974	273	4.94E-99	Type I polyketide synthase [Mycobacterium sp. 1554424.7]	WP_066930359
KS_099	1061	SPV_cluster_262	51.695	354	3.12E-115	Beta-ketoacyl-[acyl-carrier-protein] synthase II [Desulfotomaculum acetoxidans]	WP_015756782
KS_100	1292	SPV_cluster_356	40.46	435	3.86E-86	Type I polyketide synthase [Streptomyces sp. WMMB 714]	WP_045862080
KS_114	1043	SPV_cluster_318	74.203	345	0	Beta-ketoacyl-ACP synthase I [Neptuniibacter pectenicola]	WP_067983865
KS_118	1223	SPV_cluster_209	59.705	407	1.01E-176	Beta-ketoacyl-[acyl-carrier-protein] synthase II [Clostridiisalibacter paucivorans]	WP_026893881
KS_119	1199	SPV_cluster_043	67.506	397	0	Beta-ketoacyl-[acyl-carrier-protein] synthase family protein [Halocynthiibacter namhaensis]	WP_039018003
KS_121	1223	SPV_cluster_417	60.934	407	0	Beta-ketoacyl-[acyl-carrier-protein] synthase II [Brevibacillus borstelensis]	WP_003386033
KS_122	1184	SPV_cluster_401	75.949	395	0	Beta-ketoacyl-ACP synthase I [Methylarcula marina]	WP_106692425
KS_123	1223	SPV_cluster_099	74.138	406	0	Beta-ketoacyl-[acyl-carrier-protein] synthase II [Thioalkalivibrio sp. HK1]	WP_038098656
KS_124	1202	SPV_cluster_375	67.419	399	0	Beta-ketoacyl-ACP synthase I [Methylomarinum vadi]	WP_031432763
KS_125	1250	SPV_cluster_160	73.366	413	0	Beta-ketoacyl-[acyl-carrier-protein] synthase II [Rhodospirillales bacterium URHD0088]	WP_027300572
KS_127	1205	SPV_cluster_228	96.02	402	0	Beta-ketoacyl-ACP synthase I [Rhodospirillaceae bacterium Spongia-Bin9]	WP_108794197
KS_128	1223	SPV_cluster_054	60.197	407	3.85E-180	Beta-ketoacyl-[acyl-carrier-protein] synthase II [Brevibacillus borstelensis]	WP_003386033
KS_129	1223	SPV_cluster_033	58.824	408	3.39E-179	Beta-ketoacyl-[acyl-carrier-protein] synthase II [Risungbinella massiliensis]	WP_044642594
KS_132	1196	SPV_cluster_416	67.839	398	0	Beta-ketoacyl-ACP synthase I [Halomonas sp. HL-48]	WP_027337473
KS_133	998	SPV_cluster_358	67.164	335	3.62E-165	Beta-ketoacyl-[acyl-carrier-protein] synthase II [Caldilinea aerophila]	WP_044276402
KS_136	1223	SPV_cluster_324	59.705	407	0	Beta-ketoacyl-[acyl-carrier-protein] synthase II [Risungbinella	WP_044642594

						massiliensis]	
KS_139	1202	SPV_cluster_293	71.859	398	0	Beta-ketoacyl-ACP synthase I [Salinicola sp. MIT1003]	WP_071230737
KS_141	1211	SPV_cluster_230	78.5	400	0	Beta-ketoacyl-ACP synthase I [Paracoccus sphaerophysae]	WP_036717493
KS_145	1256	SPV_cluster_074	70.12	415	0	Beta-ketoacyl-[acyl-carrier-protein] synthase II [alpha proteobacterium BAL199]	WP_040746409
KS_147	1208	SPV_cluster_155	75.25	400	0	Beta-ketoacyl-ACP synthase I [Inquilinus limosus]	WP_026872496
KS_149	1223	SPV_cluster_076	81.773	406	0	Beta-ketoacyl-[acyl-carrier-protein] synthase II [Thioalkalivibrio sp. HK1]	WP_038098656
KS_152	1226	SPV_cluster_251	54.902	408	4.48E-172	Beta-ketoacyl-[acyl-carrier-protein] synthase II [Spirochaeta lutea]	WP_037548043
KS_155	1058	SPV_cluster_438	67.898	352	1.23E-177	Beta-ketoacyl-ACP synthase I [Beggiatoa alba]	WP_002683102
KS_156	1100	SPV_cluster_349	61.538	364	6.12E-161	Beta-ketoacyl-[acyl-carrier-protein] synthase II [Spirochaeta lutea]	WP_037548043
KS_157	1223	SPV_cluster_032	66.422	408	0	Beta-ketoacyl-[acyl-carrier-protein] synthase II [Pyrinomonas methylaliphatogenes]	WP_041976954
KS_158	1220	SPV_cluster_233	52.854	403	1.91E-141	Beta-ketoacyl-[acyl-carrier-protein] synthase family protein [Nibricoccus aquaticus]	WP_096057284
KS_160	1286	SPV_cluster_245	57.71	428	3.29E-146	Type I polyketide synthase [Mycolicibacterium vanbaalenii]	WP_011778276
KS_164	1223	SPV_cluster_329	66.667	408	0	Beta-ketoacyl-[acyl-carrier-protein] synthase II [Pyrinomonas methylaliphatogenes]	WP_041976954
KS_165	1223	SPV_cluster_039	57.248	407	3.69E-179	Beta-ketoacyl-[acyl-carrier-protein] synthase II [Planifilum fimeticola]	WP_106344983
KS_168	1223	SPV_cluster_002	64.039	406	0	Beta-ketoacyl-[acyl-carrier-protein] synthase II [Moorella humiferrea]	WP_106006374
KS_169	1253	SPV_cluster_413	55.66	424	1.22E-141	Type I polyketide synthase [Mycolicibacterium austroafricanum]	WP_051558318
KS_171	1223	SPV_cluster_235	69.779	407	0	Beta-ketoacyl-[acyl-carrier-protein] synthase II [Solibacteres bacterium SbA2]	WP_105314403
KS_172	1229	SPV_cluster_014	57.805	410	5.53E-170	Beta-ketoacyl-[acyl-carrier-protein] synthase II [Numidum massiliense]	WP_054949919
KS_177	1292	SPV_cluster_421	42.529	435	5.09E-87	Type I polyketide synthase [Nocardiopsis sp. NRRL B-16309]	WP_053618745
KS_180	1028	SPV_cluster_432	65.116	344	3.10E-153	Beta-ketoacyl-[acyl-carrier-protein] synthase II [Thermoflexus hugenholtzii]	WP_098504503
KS_184	1217	SPV_cluster_430	57.778	405	2.78E-174	Beta-ketoacyl-[acyl-carrier-protein] synthase II [Bacillales]	WP_029100165
KS_185	1052	SPV_cluster_411	52.454	326	2.19E-109	Beta-ketoacyl-[acyl-carrier-protein] synthase family protein [Rhizobiales bacterium]	WP_109794138
KS_187	1223	SPV_cluster_254	65.517	406	0	Beta-ketoacyl-[acyl-carrier-protein] synthase II [Dyella]	WP_026634449
KS_190	1223	SPV_cluster_284	67.801	382	0	Beta-ketoacyl-[acyl-carrier-protein] synthase II [Oceanicoccus sagamiensis]	WP_085759718
KS_192	1202	SPV_cluster_138	61.443	402	1.14E-176	Beta-ketoacyl-ACP synthase [Pseudoduganella violaceinigra]	WP_028104973
KS_194	1229	SPV_cluster_317	54.39	410	2.49E-144	Beta-ketoacyl-[acyl-carrier-protein] synthase II [Alicyclobacillus macrosporangiidus]	WP_074950143
KS_200	1223	SPV_cluster_258	64.691	405	0	Beta-ketoacyl-[acyl-carrier-protein] synthase II [Pyrinomonas methylaliphatogenes]	WP_041976954

KS_207	1223	SPV_cluster_389	74.278	381	0	Beta-ketoacyl-[acyl-carrier-protein] synthase II [Thioalkalivibrio sp. HK1]	WP_038098656
KS_209	1220	SPV_cluster_128	65.356	407	0	Beta-ketoacyl-[acyl-carrier-protein] synthase II [Halomonas pantelleriensis]	WP_089658562
KS_210	1214	SPV_cluster_009	63.793	406	0	Beta-ketoacyl-[acyl-carrier-protein] synthase II [Reinekea blandensis]	WP_008043526
KS_211	1340	SPV_cluster_001	52.954	457	4.05E-139	Type I Polyketide synthase [Chondromyces crocatus]	WP_082362498
KS_213	1229	SPV_cluster_441	54.657	408	2.92E-165	Beta-ketoacyl-[acyl-carrier-protein] synthase II [Spirochaeta lutea]	WP_037548043
KS_214	1223	SPV_cluster_289	63.951	405	0	Beta-ketoacyl-[acyl-carrier-protein] synthase II [Woeseia oceani]	WP_068615706
KS_215	1214	SPV_cluster_239	71.921	406	4.31E-180	Beta-ketoacyl-[acyl-carrier-protein] synthase II [Thermoflexus hugenholtzii]	WP_098504331
KS_216	1226	SPV_cluster_003	61.425	407	2.27E-179	Beta-ketoacyl-[acyl-carrier-protein] synthase II [Pseudomonas abyssi]	WP_096004673
KS_219	1199	SPV_cluster_148	72.475	396	0	Beta-ketoacyl-[acyl-carrier-protein] synthase family protein [Rhodovulum sulfidophilum]	WP_075786012
KS_226	1157	SPV_cluster_086	54.45	382	4.06E-134	Beta-ketoacyl-ACP synthase II [Ferrimicrobium acidiphilum]	WP_052566261
KS_228	1223	SPV_cluster_195	50.735	408	1.55E-145	Beta-ketoacyl-[acyl-carrier-protein] synthase II [Effusibacillus pohliae]	WP_026174345
KS_229	1217	SPV_cluster_280	64.461	408	0	Beta-ketoacyl-[acyl-carrier-protein] synthase II [Acidihalobacter prosperus]	WP_038086398
KS_230	1223	SPV_cluster_037	71.253	407	0	Beta-ketoacyl-[acyl-carrier-protein] synthase II [Ectothiorhodospira haloalkaliphila]	WP_025281708
KS_233	1217	SPV_cluster_060	65.926	405	0	Beta-ketoacyl-[acyl-carrier-protein] synthase II [Acidihalobacter ferrooxidans]	WP_076837285
KS_234	1217	SPV_cluster_121	64.706	408	0	Beta-ketoacyl-[acyl-carrier-protein] synthase II [Acidihalobacter prosperus]	WP_038086398
KS_238	1217	SPV_cluster_180	58.519	405	4.55E-177	Beta-ketoacyl-[acyl-carrier-protein] synthase II [Bacillales]	WP_029100165
KS_241	1163	SPV_cluster_207	63.089	382	0	Beta-ketoacyl-[acyl-carrier-protein] synthase II [Pseudomonas pachastrellae]	WP_083725910
KS_242	1226	SPV_cluster_445	61.916	407	0	Beta-ketoacyl-[acyl-carrier-protein] synthase II [Pseudomonas pachastrellae]	WP_083725910
KS_243	1217	SPV_cluster_015	64.109	404	0	Beta-ketoacyl-[acyl-carrier-protein] synthase II [Pseudomonas pachastrellae]	WP_083725910
KS_245	1235	SPV_cluster_178	69.082	414	0	Beta-ketoacyl-[acyl-carrier-protein] synthase II [Albidovulum xiamenense]	WP_092498652
KS_246	1187	SPV_cluster_126	50.598	251	1.10E-71	Beta-ketoacyl-[acyl-carrier-protein] synthase family protein [Legionella drozanskii]	WP_058494995
KS_248	1220	SPV_cluster_197	65.111	407	0	Beta-ketoacyl-[acyl-carrier-protein] synthase II [Halomonas halodenitrificans]	WP_027960955
KS_251	1247	SPV_cluster_101	71.117	412	0	Beta-ketoacyl-[acyl-carrier-protein] synthase II [Rhodospirillales bacterium URHD0088]	WP_027300572
KS_255	1229	SPV_cluster_222	62.347	409	0	Beta-ketoacyl-[acyl-carrier-protein] synthase II [Nitrosomonas aestuarii]	WP_090702733
KS_257	1292	SPV_cluster_426	42.299	435	3.14E-87	Type I polyketide synthase, partial [Streptomyces sp. SM5]	WP_103492552
KS_258	1220	SPV_cluster_036	53.35	403	2.98E-137	Beta-ketoacyl-[acyl-carrier-protein] synthase family protein	WP_096057284

						[Nibricoccus aquaticus]	
KS_260	1226	SPV_cluster_419	61.029	408	0	Beta-ketoacyl-[acyl-carrier-protein] synthase II [Aneurinibacillus terranovensis]	WP_027414564
KS_265	1292	SPV_cluster_091	44.749	438	6.24E-94	Type I polyketide synthase [Sciscionella marina]	WP_020497474
KS_268	1292	SPV_cluster_172	42.257	452	7.20E-92	Type I polyketide synthase [Sciscionella marina]	WP_020497474
KS_271	1220	SPV_cluster_255	63.793	406	0	Beta-ketoacyl-[acyl-carrier-protein] synthase II [Thermoanaerobaculum aquaticum]	WP_038046901
KS_272	1286	SPV_cluster_005	52.174	437	1.11E-136	Hybrid non-ribosomal peptide synthetase/Type I polyketide synthase [Burkholderia vietnamiensis]	WP_059991443
KS_273	1220	SPV_cluster_025	63.054	406	0	Beta-ketoacyl-[acyl-carrier-protein] synthase II [Thermoanaerobaculum aquaticum]	WP_038046901
KS_274	1226	SPV_cluster_024	60.294	408	0	Beta-ketoacyl-[acyl-carrier-protein] synthase II [Vulcanibacillus modesticaldus]	WP_069655673
KS_275	1229	SPV_cluster_031	56.829	410	7.38E-163	Beta-ketoacyl-[acyl-carrier-protein] synthase II [Rhodothermus marinus]	WP_012845172
KS_276	1223	SPV_cluster_217	72.861	409	0	Beta-ketoacyl-[acyl-carrier-protein] synthase II [Thiothrix caldifontis]	WP_093066550
KS_277	1151	SPV_cluster_129	58.377	382	1.34E-141	Beta-ketoacyl-ACP synthase II [Streptomyces sp. NRRL F-2664]	WP_030765373
KS_280	1151	SPV_cluster_229	33.995	403	3.01E-55	Beta-ketoacyl-[acyl-carrier-protein] synthase II [Synechococcus]	WP_011243296
KS_281	1292	SPV_cluster_035	54.318	440	2.71E-138	Type I polyketide synthase [Mycolicibacter icosiumassiliensis]	WP_078058707
KS_282	1226	SPV_cluster_159	61.975	405	1.76E-173	Beta-ketoacyl-[acyl-carrier-protein] synthase II [Thermoflexus hugenholtzii]	WP_098502948
KS_286	1229	SPV_cluster_402	55.774	407	6.45E-159	Beta-ketoacyl-[acyl-carrier-protein] synthase II [Desulfotomaculum acetoxidans]	WP_015756782
KS_287	1289	SPV_cluster_214	50.465	430	1.77E-132	Type I polyketide synthase [Mycobacterium bohemicum]	WP_085180210
KS_288	1289	SPV_cluster_040	50.698	430	2.39E-132	Type I polyketide synthase [Mycobacterium bohemicum]	WP_085180210
KS_289	1286	SPV_cluster_225	63.593	423	3.69E-180	Type I polyketide synthase [Marinobacterium profundum]	WP_067295857
KS_291	1223	SPV_cluster_107	65.517	406	0	Beta-ketoacyl-[acyl-carrier-protein] synthase II [Geobacter sp. DSM 2909]	WP_108103150
KS_293	1292	SPV_cluster_161	41.136	440	2.37E-87	Type I polyketide synthase [Crossiella equi]	WP_086785133
KS_297	1214	SPV_cluster_085	55.693	404	3.80E-155	Beta-ketoacyl-[acyl-carrier-protein] synthase II [Thermorudis peleae]	WP_038037697
KS_301	1154	SPV_cluster_093	53.786	383	2.20E-131	Hypothetical protein [Tropicibacter phthalicicus]	WP_099241578
KS_303	1286	SPV_cluster_084	55.374	428	2.61E-149	Acyltransferase domain-containing protein [Nostoc commune]	WP_109009597
KS_305	1067	SPV_cluster_406	55.12	332	3.72E-113	Beta-ketoacyl-[acyl-carrier-protein] synthase II [Roseiflexus castenholzii]	WP_041330400
KS_306	1286	SPV_cluster_268	52.644	435	4.67E-136	Type I polyketide synthase, partial [Burkholderia vietnamiensis]	WP_059668134
KS_307	1259	SPV_cluster_312	59.905	419	1.89E-177	Beta-ketoacyl-[acyl-carrier-protein] synthase family protein [Syntrophus aciditrophicus]	WP_011418884
KS_308	1154	SPV_cluster_124	51.558	353	4.76E-110	Hypothetical protein [Tropicibacter phthalicicus]	WP_099241578
KS_310	1223	SPV_cluster_400	60.197	407	0	Beta-ketoacyl-[acyl-carrier-protein] synthase II [Kroppenstedtia	WP_076523249

						eburnea]	
KS_312	1160	SPV_cluster_113	60.807	347	1.90E-131	Beta-ketoacyl-ACP synthase II [Streptomyces katrae]	WP_045950542
KS_315	1160	SPV_cluster_320	62.141	383	3.80E-147	Beta-ketoacyl-ACP synthase II [Streptomyces katrae]	WP_045950542
KS_317	1160	SPV_cluster_224	61.96	347	3.80E-133	Beta-ketoacyl-ACP synthase II [Streptomyces katrae]	WP_045950542
KS_321	1217	SPV_cluster_081	54.455	404	4.36E-154	Beta-ketoacyl-[acyl-carrier-protein] synthase II [Thermomicrobium roseum]	WP_012642981
KS_323	1286	SPV_cluster_253	53.472	432	1.67E-138	Type I polyketide synthase, partial [Burkholderia vietnamiensis]	WP_059668134
KS_326	1223	SPV_cluster_353	57.843	408	1.63E-169	Beta-ketoacyl-[acyl-carrier-protein] synthase II [Alicyclobacillus sendaiensis]	WP_062309646
KS_332	1286	SPV_cluster_171	53.563	435	7.50E-139	Type I polyketide synthase, partial [Burkholderia vietnamiensis]	WP_059895285
KS_336	1292	SPV_cluster_163	43.119	436	1.02E-93	SDR family NAD(P)-dependent oxidoreductase [Burkholderia cenocepacia]	WP_109341745
KS_337	1292	SPV_cluster_153	44.138	435	6.00E-89	Type I polyketide synthase [Streptomyces rubellomurinus]	WP_078860657
KS_339	1292	SPV_cluster_310	42.166	434	3.47E-88	SDR family NAD(P)-dependent oxidoreductase [Burkholderia cenocepacia]	WP_109341745
KS_341	1226	SPV_cluster_276	60.784	408	3.48E-169	Beta-ketoacyl-[acyl-carrier-protein] synthase II [Thermoflexus hugenholtzii]	WP_098502948
KS_345	1154	SPV_cluster_290	58.398	387	1.23E-128	Beta-ketoacyl-ACP synthase II [Ilumatobacter nonamiensis]	WP_040493091
KS_349	1247	SPV_cluster_205	72.222	414	0	Beta-ketoacyl-[acyl-carrier-protein] synthase II [Tistrella mobilis]	WP_062765245
KS_350	1292	SPV_cluster_300	40.23	435	1.37E-85	Type I polyketide synthase [Streptomyces sp. WMMB 714]	WP_045862080
KS_351	1148	SPV_cluster_436	71.316	380	0	Beta-ketoacyl-[acyl-carrier-protein] synthase II [Oceanibaculum pacificum]	WP_067553414
KS_353	1292	SPV_cluster_108	44.545	440	6.87E-96	Type I polyketide synthase [Sciscionella marina]	WP_020497474
KS_356	1292	SPV_cluster_166	39.066	407	6.07E-79	Type I polyketide synthase [Stigmatella erecta]	WP_093522399
KS_357	1292	SPV_cluster_281	40.183	438	2.83E-85	Type I polyketide synthase [Vulcanococcus limneticus]	WP_094591717
KS_360	1232	SPV_cluster_192	70.244	410	0	Beta-ketoacyl-[acyl-carrier-protein] synthase II [Rhodothermus marinus]	WP_014068140
KS_361	1292	SPV_cluster_065	39.723	433	5.71E-80	Type I polyketide synthase [Burkholderia thailandensis]	WP_038712449
KS_362	1220	SPV_cluster_271	66.256	406	0	Beta-ketoacyl-[acyl-carrier-protein] synthase II [Opitutaceae bacterium EW11]	WP_107743231
KS_363	1220	SPV_cluster_045	52.826	407	3.49E-158	Beta-ketoacyl-[acyl-carrier-protein] synthase II [Bacillus methanolicus]	WP_004433597
KS_369	1229	SPV_cluster_007	58.68	409	5.74E-165	Beta-ketoacyl-[acyl-carrier-protein] synthase II [Desulfovirgula thermocuniculi]	WP_027718279
KS_370	1100	SPV_cluster_112	48.221	253	1.18E-58	3-oxoacyl-ACP synthase [Lechevalieria fradiae]	WP_090045424
KS_371	1298	SPV_cluster_141	42.759	435	3.75E-87	Type I polyketide synthase [Streptomyces]	WP_030626723
KS_378	1223	SPV_cluster_363	56.479	409	1.26E-163	Beta-ketoacyl-[acyl-carrier-protein] synthase II [Chloroflexus sp. MS-G]	WP_031461218
KS_379	1253	SPV_cluster_362	72.837	416	0	Beta-ketoacyl-[acyl-carrier-protein] synthase II [Tistrella mobilis]	WP_062765245

KS_381	1229	SPV_cluster_367	56.127	408	4.56E-159	Beta-ketoacyl-[acyl-carrier-protein] synthase II [Thermaerobacter subterraneus]	WP_006905000
KS_383	1220	SPV_cluster_027	73.515	404	0	Beta-ketoacyl-[acyl-carrier-protein] synthase II [Candidatus Nitrospira nitrosa]	WP_090746581
KS_385	1229	SPV_cluster_341	56.098	410	7.70E-159	Beta-ketoacyl-[acyl-carrier-protein] synthase II [Melghirimyces profundicolus]	WP_108022554
KS_386	1232	SPV_cluster_173	69.756	410	0	Beta-ketoacyl-[acyl-carrier-protein] synthase II [Rhodothermus marinus]	WP_014068140
KS_389	1247	SPV_cluster_042	69.976	413	0	Beta-ketoacyl-[acyl-carrier-protein] synthase II [Rhodospirillales bacterium URHD0088]	WP_027300572
KS_391	1154	SPV_cluster_442	71.99	382	0	Beta-ketoacyl-[acyl-carrier-protein] synthase II [Thermoflexus hugenholtzii]	WP_098502948
KS_394	1226	SPV_cluster_053	48.905	411	9.42E-134	Beta-ketoacyl-[acyl-carrier-protein] synthase II [Ardenticatena maritima]	WP_054491905
KS_399	1286	SPV_cluster_057	56.744	430	4.55E-139	Type I polyketide synthase [Mycolicibacterium austroafricanum]	WP_105389463
KS_401	1184	SPV_cluster_125	60.204	392	1.50E-169	Beta-ketoacyl-[acyl-carrier-protein] synthase family protein [Pseudovibrio sp. AU243]	WP_109314797
KS_402	1184	SPV_cluster_092	63.52	392	2.15E-171	Beta-ketoacyl-[acyl-carrier-protein] synthase family protein [Tropicibacter phthalicicus]	WP_099241574
KS_404	1223	SPV_cluster_275	51.106	407	6.81E-143	Beta-ketoacyl-[acyl-carrier-protein] synthase II [Ardenticatena maritima]	WP_054493616
KS_405	1292	SPV_cluster_049	43.333	450	2.20E-93	Type I polyketide synthase [Sciscionella marina]	WP_020497474
KS_409	1292	SPV_cluster_067	41.907	451	6.03E-93	Type I polyketide synthase [Sciscionella marina]	WP_020497474
KS_415	1232	SPV_cluster_308	46.341	410	6.62E-134	Beta-ketoacyl-[acyl-carrier-protein] synthase II [Vulcanibacillus modesticaldus]	WP_069655673
KS_416	1286	SPV_cluster_168	59.207	429	2.35E-148	Type I polyketide synthase [Mycolicibacterium austroafricanum]	WP_105389463
KS_418	1088	SPV_cluster_444	57.576	363	1.22E-151	Beta-ketoacyl-[acyl-carrier-protein] synthase II [Desulfuribacillus stibiiarsenatis]	WP_069701864
KS_420	1286	SPV_cluster_371	55.478	429	3.09E-140	Type I polyketide synthase [Mycobacterium]	WP_041309443
KS_422	1286	SPV_cluster_149	52.204	431	5.25E-134	Type I polyketide synthase, partial [Burkholderia vietnamiensis]	WP_059694304
KS_425	1232	SPV_cluster_201	70	410	0	Beta-ketoacyl-[acyl-carrier-protein] synthase II [Caldilinea aerophila]	WP_044276164
KS_426	1211	SPV_cluster_339	73.367	398	0	Beta-ketoacyl-[acyl-carrier-protein] synthase II [Rhodospirillales bacterium URHD0088]	WP_027300572
KS_427	1175	SPV_cluster_215	57.682	371	1.69E-150	Beta-ketoacyl-[acyl-carrier-protein] synthase II [Thermomicrobium roseum]	WP_012642981
KS_432	1286	SPV_cluster_146	54.861	432	3.55E-141	Type I polyketide synthase, partial [Burkholderia vietnamiensis]	WP_059895285
KS_433	1175	SPV_cluster_307	56.065	371	2.71E-148	Beta-ketoacyl-[acyl-carrier-protein] synthase II [Thermomicrobium roseum]	WP_012642981
KS_434	1223	SPV_cluster_340	56.436	404	4.91E-163	Beta-ketoacyl-[acyl-carrier-protein] synthase II [Desulfotomaculum acetoxidans]	WP_015756782
KS_435	1094	SPV_cluster_345	30.699	329	3.91E-45	Hypothetical protein [Flavobacterium aurantiibacter]	WP_094485954
KS_436	1232	SPV_cluster_216	48.78	410	3.04E-131	Beta-ketoacyl-[acyl-carrier-protein] synthase II [Bacillus oleivorans]	WP_097159866

KS_438	1223	SPV_cluster_301	58.867	406	1.04E-162	Beta-ketoacyl-[acyl-carrier-protein] synthase II [Thermoflexus hugenholtzii]	WP_098502948
KS_441	1046	SPV_cluster_443	56.232	345	2.75E-135	Beta-ketoacyl-[acyl-carrier-protein] synthase II [Thermomicrobium roseum]	WP_012642981
KS_442	1175	SPV_cluster_152	56.604	371	6.34E-149	Beta-ketoacyl-[acyl-carrier-protein] synthase II [Thermomicrobium roseum]	WP_012642981
KS_443	1175	SPV_cluster_414	61.983	363	2.40E-139	Beta-ketoacyl-[acyl-carrier-protein] synthase II [Thermoflexus hugenholtzii]	WP_098504331
KS_444	1235	SPV_cluster_083	57.664	411	9.24E-171	Beta-ketoacyl-[acyl-carrier-protein] synthase II [Desmospora sp. 8437]	WP_040387222
KS_445	1286	SPV_cluster_071	54.545	429	1.14E-139	Type I polyketide synthase, partial [Burkholderia vietnamiensis]	WP_059567396
KS_450	1292	SPV_cluster_412	41.333	450	5.04E-90	Type I polyketide synthase [Sciscionella marina]	WP_020497474
KS_451	1289	SPV_cluster_114	54.206	428	3.08E-137	Type I polyketide synthase [Mycolicibacterium austroafricanum]	WP_051558318
KS_456	1184	SPV_cluster_263	73.59	390	0	Beta-ketoacyl-[acyl-carrier-protein] synthase II [Thioalkalivibrio sp. HK1]	WP_038098656
KS_457	1286	SPV_cluster_047	53.828	431	2.74E-130	Type I polyketide synthase, partial [Mycobacterium parascrofulaceum]	WP_007169537
KS_458	1145	SPV_cluster_236	55.263	380	1.32E-128	Beta-ketoacyl-[acyl-carrier-protein] synthase family protein [Mizugakiibacter sediminis]	WP_082306570
KS_463	1190	SPV_cluster_066	69.424	399	0	Beta-ketoacyl-[acyl-carrier-protein] synthase II [Pararhodobacter aggregans]	WP_107749575
KS_464	1178	SPV_cluster_069	67.519	391	0	Beta-ketoacyl-[acyl-carrier-protein] synthase II [Bryobacter aggregatus]	WP_031496688
KS_467	1196	SPV_cluster_364	54.68	406	1.49E-137	Beta-ketoacyl-[acyl-carrier-protein] synthase II [Thermocrinis albus]	WP_012991141
KS_470	977	SPV_cluster_330	92.26	323	0	Beta-ketoacyl-[acyl-carrier-protein] synthase II [Rhodospirillaceae bacterium Spongia-Bin9]	WP_108792873
KS_472	1211	SPV_cluster_259	74.121	398	0	Beta-ketoacyl-[acyl-carrier-protein] synthase II [Telmatospirillum siberiense]	WP_101252614
KS_475	1154	SPV_cluster_366	72.324	383	0	Beta-ketoacyl-ACP synthase I [Thermopetrobacter sp. TC1]	WP_038035251
KS_477	1286	SPV_cluster_291	52.558	430	4.86E-138	Polyketide synthase [Scytonema sp. HK-05]	WP_073629410
KS_478	1142	SPV_cluster_380	38.806	201	3.36E-29	Beta-ketoacyl-[acyl-carrier-protein] synthase family protein [Streptomyces atratus]	WP_072488766
KS_480	1199	SPV_cluster_334	75.127	394	0	Beta-ketoacyl-[acyl-carrier-protein] synthase II [Rhodospirillales bacterium URHD0088]	WP_027300572
KS_482	1172	SPV_cluster_182	59.383	389	1.88E-172	Beta-ketoacyl-[acyl-carrier-protein] synthase II [Vulcanibacillus modesticaldus]	WP_069655673
KS_485	1298	SPV_cluster_226	56.019	432	2.47E-139	Type I polyketide synthase [Mycobacterium malmoense]	WP_071509339
KS_487	1112	SPV_cluster_179	62.216	352	1.20E-134	Beta-ketoacyl-ACP synthase II [Ilumatobacter nonamiensis]	WP_040493091
KS_489	1292	SPV_cluster_220	43.779	434	2.87E-92	Type I polyketide synthase, partial [Streptomyces sp. SM5]	WP_103492552
KS_492	1172	SPV_cluster_240	68.475	387	0	Beta-ketoacyl-[acyl-carrier-protein] synthase II [Thermoflexus hugenholtzii]	WP_098504330
KS_493	1286	SPV_cluster_134	50.577	433	1.62E-133	Type I polyketide synthase [Moorea producens]	WP_071104824

KS_496	1166	SPV_cluster_274	58.098	389	1.05E-170	Beta-ketoacyl-[acyl-carrier-protein] synthase II [Vulcanibacillus modesticaldus]	WP_069655673
KS_500	1286	SPV_cluster_278	60.047	428	1.02E-151	Type I polyketide synthase [Mycolicibacterium austroafricanum]	WP_105389463
KS_502	1286	SPV_cluster_213	55.012	429	5.02E-142	Type I polyketide synthase, partial [Burkholderia vietnamiensis]	WP_059668134
KS_509	1292	SPV_cluster_154	53.009	432	1.01E-136	Hybrid non-ribosomal peptide synthetase/Type I polyketide synthase [Burkholderia vietnamiensis]	WP_059603382
KS_510	1292	SPV_cluster_020	53.704	432	2.44E-139	Type I polyketide synthase, partial [Burkholderia vietnamiensis]	WP_059567396
KS_512	1292	SPV_cluster_264	43.218	435	1.69E-88	Type I polyketide synthase [Streptomyces rubellomurinus]	WP_078860657
KS_513	1160	SPV_cluster_316	56.623	385	2.31E-164	Beta-ketoacyl-[acyl-carrier-protein] synthase II [Vulcanibacillus modesticaldus]	WP_069655673
KS_515	1244	SPV_cluster_029	61.557	424	2.54E-152	Type I polyketide synthase [Mycolicibacterium austroafricanum]	WP_051558318
KS_522	1229	SPV_cluster_433	57.702	409	1.83E-168	Beta-ketoacyl-[acyl-carrier-protein] synthase II [Thermicanus aegyptius]	WP_028987687
KS_523	1283	SPV_cluster_208	60.047	428	5.12E-150	Type I polyketide synthase [Mycolicibacterium vanbaalenii]	WP_011778276
KS_524	1286	SPV_cluster_051	54.079	429	1.08E-141	Type I polyketide synthase, partial [Burkholderia vietnamiensis]	WP_059567396
KS_530	1292	SPV_cluster_174	52.214	429	2.02E-134	Hybrid non-ribosomal peptide synthetase/Type I polyketide synthase [Burkholderia vietnamiensis]	WP_059603382
KS_531	1058	SPV_cluster_337	93.023	258	1.78E-168	Beta-ketoacyl-ACP synthase [Rhodospirillaceae bacterium Spongia-Bin9]	WP_108793187
KS_533	1292	SPV_cluster_368	41.002	439	1.25E-90	Type I polyketide synthase [Crossiella equi]	WP_086785133
KS_534	1286	SPV_cluster_326	57.674	430	1.27E-150	Type I polyketide synthase [Mycolicibacterium diernhoferi]	WP_073855454
KS_535	1286	SPV_cluster_269	54.545	429	6.68E-139	Hybrid non-ribosomal peptide synthetase/Type I polyketide synthase [Burkholderia vietnamiensis]	WP_059603382
KS_539	1286	SPV_cluster_102	57.11	429	4.66E-145	Type I polyketide synthase [Mycolicibacterium obuense]	WP_048421716
KS_542	1286	SPV_cluster_299	53.935	432	1.04E-140	Type I polyketide synthase, partial [Burkholderia vietnamiensis]	WP_059668134
KS_544	1286	SPV_cluster_078	54.801	427	3.54E-153	Polyketide synthase [Stanieria cyanosphaera]	WP_015193130
KS_547	1292	SPV_cluster_008	53.846	429	2.94E-140	Hybrid non-ribosomal peptide synthetase/Type I polyketide synthase [Burkholderia vietnamiensis]	WP_059740130
KS_552	1277	SPV_cluster_169	62.441	426	2.34E-159	Type I polyketide synthase [Mycolicibacterium austroafricanum]	WP_105389463
KS_556	1286	SPV_cluster_212	55.116	430	7.99E-141	Type I polyketide synthase [Mycolicibacterium tusciae]	WP_006241768
KS_560	1283	SPV_cluster_082	57.11	429	1.64E-143	Type I polyketide synthase [Mycobacterium]	WP_064413884
KS_561	1250	SPV_cluster_221	60.28	428	4.17E-151	Type I polyketide synthase [Mycolicibacterium austroafricanum]	WP_105389463
KS_562	1292	SPV_cluster_151	55.711	429	2.14E-140	Type I polyketide synthase [Mycolicibacter icosiumassiliensis]	WP_078058707
KS_565	1259	SPV_cluster_030	61.502	426	2.52E-158	Type I polyketide synthase [Mycobacterium colombiense]	WP_076107742
KS_566	1286	SPV_cluster_311	54.503	433	3.00E-139	Type I polyketide synthase [Mycobacterium sp. 852002-51961_SCH5331710]	WP_067083408
KS_567	1301	SPV_cluster_246	58.605	430	7.73E-146	Type I polyketide synthase [Mycolicibacterium austroafricanum]	WP_105389463

KS_568	1166	SPV_cluster_250	61.538	351	8.15E-145	Beta-ketoacyl-[acyl-carrier-protein] synthase family protein [Cephaloticoccus capnophilus]	WP_068711256
KS_571	1082	SPV_cluster_376	44.798	346	1.78E-80	Beta-ketoacyl-ACP synthase I [Melaminivora sp. SC2-7]	WP_106847441
KS_574	1292	SPV_cluster_052	52.315	432	5.03E-137	Type I polyketide synthase, partial [Burkholderia vietnamiensis]	WP_059694304
KS_578	1178	SPV_cluster_094	61.675	394	2.00E-156	Hypothetical protein [Verminephrobacter aporrectodeae]	WP_081479930
KS_582	1010	SPV_cluster_137	53.266	199	2.08E-62	Beta-ketoacyl-ACP synthase [Cellvibrio sp. pealriver]	WP_082191725
KS_584	1025	SPV_cluster_136	67.755	245	1.28E-106	Beta-ketoacyl-ACP synthase [Thiobacillus denitrificans]	WP_059752414
KS_587	1184	SPV_cluster_123	59.836	366	1.79E-136	Hypothetical protein [Verminephrobacter aporrectodeae]	WP_081479930
KS_589	1259	SPV_cluster_338	57.21	423	2.30E-140	Type I polyketide synthase [Mycolicibacterium austroafricanum]	WP_105389463
KS_592	1277	SPV_cluster_106	60.941	425	5.36E-159	Type I polyketide synthase [Mycobacterium sp. EPa45]	WP_047332984
KS_594	293	SPV_cluster_111	59.783	92	7.54E-28	Type I polyketide synthase [Mycobacterium sp. YR782]	WP_108054808
KS_595	1259	SPV_cluster_357	55.896	424	2.81E-140	Type I polyketide synthase [filamentous cyanobacterium CCP3]	WP_106919616
KS_597	1256	SPV_cluster_309	57.447	423	3.60E-139	Type I polyketide synthase [Mycolicibacterium austroafricanum]	WP_105389463
KS_598	1259	SPV_cluster_394	51.294	425	4.70E-139	Type I polyketide synthase [Pleurocapsa minor]	WP_015144800
KS_600	1079	SPV_cluster_145	59.259	324	1.94E-133	Beta-ketoacyl synthase [Pseudomonas flexibilis]	WP_039561123
KS_601	992	SPV_cluster_261	60.905	243	1.27E-102	3-oxoacyl-ACP synthase [Myxococcus stipitatus]	WP_015352009
KS_604	851	SPV_cluster_016	68.726	259	3.09E-125	Beta-ketoacyl synthase [Vitiosangium sp. GDMCC 1.1324]	WP_108066665
KS_606	653	SPV_cluster_348	63.721	215	5.53E-86	Type I polyketide synthase, partial [Burkholderia vietnamiensis]	WP_059694304
KS_610	287	SPV_cluster_459	66.304	92	5.21E-33	Type I polyketide synthase [Mycobacterium sherrisii]	WP_085167517
KS_612	1256	SPV_cluster_106	61.647	425	1.92E-156	Type I polyketide synthase [Mycolicibacterium austroafricanum]	WP_105389463
KS_622	1259	SPV_cluster_315	50.355	423	3.36E-126	Type I polyketide synthase [Mycobacterium colombiense]	WP_044486300
KS_623	1259	SPV_cluster_305	56.235	425	1.91E-134	Type I polyketide synthase [Mycolicibacterium austroafricanum]	WP_105389463
KS_624	1259	SPV_cluster_266	55.319	423	5.14E-139	Type I polyketide synthase [Mycobacterium palustre]	WP_085077785
KS_629	1259	SPV_cluster_314	60.235	425	2.83E-148	Type I polyketide synthase [Mycolicibacterium austroafricanum]	WP_105389463
KS_631	1235	SPV_cluster_377	61.792	424	3.72E-156	Type I polyketide synthase [Mycobacterium asiaticum]	WP_065036774
KS_632	1259	SPV_cluster_167	56.44	427	9.37E-145	Type I polyketide synthase [Mycobacterium bohemicum]	WP_085180210
KS_634	1265	SPV_cluster_030	59.811	423	6.27E-149	Type I polyketide synthase [Mycolicibacterium austroafricanum]	WP_051558318
KS_635	1262	SPV_cluster_030	61.412	425	3.76E-157	Type I polyketide synthase [Mycolicibacterium austroafricanum]	WP_105389463
KS_639	1259	SPV_cluster_260	57.683	423	5.45E-143	Type I polyketide synthase, partial [Mycolicibacterium iranicum]	WP_081660130
KS_641	1259	SPV_cluster_052	54.273	433	1.56E-137	Type I polyketide synthase [Mycobacterium avium]	WP_033720430
KS_648	1265	SPV_cluster_211	57.21	423	2.82E-143	Type I polyketide synthase [Mycolicibacterium austroafricanum]	WP_051558318
KS_651	1256	SPV_cluster_227	54.439	428	3.73E-128	Type I polyketide synthase [Mycolicibacterium austroafricanum]	WP_051558318

KS_653	1259	SPV_cluster_306	57.075	424	5.62E-140	Type I polyketide synthase, partial [Mycolicibacterium iranicum]	WP_085171523
KS_656	1244	SPV_cluster_237	57.569	436	3.25E-145	Type I polyketide synthase [Mycolicibacterium austroafricanum]	WP_105389463
KS_658	1259	SPV_cluster_030	60.142	424	6.00E-151	Type I polyketide synthase [Mycolicibacterium austroafricanum]	WP_105389463
KS_663	1259	SPV_cluster_206	54.206	428	1.59E-137	Type I polyketide synthase [Mycobacterium gordonae]	WP_065161832
KS_665	1259	SPV_cluster_210	57.882	425	6.62E-142	Type I polyketide synthase [Mycolicibacterium austroafricanum]	WP_105389463
KS_667	1259	SPV_cluster_181	57.845	427	2.82E-143	Type I polyketide synthase [Mycolicibacterium austroafricanum]	WP_105389463
KS_675	1268	SPV_cluster_030	62.411	423	3.97E-160	Type I polyketide synthase [Mycolicibacterium austroafricanum]	WP_105389463
KS_676	1259	SPV_cluster_082	56.471	425	8.66E-139	Type I polyketide synthase [Mycolicibacterium vanbaalenii]	WP_011778276
KS_678	1253	SPV_cluster_221	52.927	427	8.13E-130	Type I polyketide synthase [Mycobacterium colombiense]	WP_077092494
KS_680	1277	SPV_cluster_030	60.78	436	8.34E-157	Type I polyketide synthase [Mycolicibacterium austroafricanum]	WP_105389463
KS_684	1268	SPV_cluster_030	60.757	423	2.61E-154	Type I polyketide synthase [Mycobacterium sp. TBL 1200985]	WP_085324847
KS_686	1256	SPV_cluster_191	55.478	429	1.03E-143	Type I polyketide synthase [Mycobacterium sp. UNC280MFTsu5.1]	WP_043403121
KS_687	1259	SPV_cluster_051	54.717	424	1.34E-138	Type I polyketide synthase [Mycobacterium sp. 1245111.1]	WP_067331050
KS_690	1259	SPV_cluster_096	55.529	425	4.39E-137	Type I polyketide synthase [Mycolicibacterium austroafricanum]	WP_105389463
KS_694	1259	SPV_cluster_057	56	425	2.23E-142	Type I polyketide synthase [Mycobacterium parmense]	WP_085270770
KS_696	1259	SPV_cluster_171	58.156	423	2.13E-143	Type I polyketide synthase [Mycolicibacterium austroafricanum]	WP_105389463
KS_698	1259	SPV_cluster_040	55.634	426	3.57E-139	Type I polyketide synthase [Mycolicibacterium vanbaalenii]	WP_011778276
KS_699	1259	SPV_cluster_214	55.399	426	9.00E-139	Type I polyketide synthase [Mycolicibacterium vanbaalenii]	WP_011778276
KS_702	1271	SPV_cluster_029	60.757	423	3.73E-153	Type I polyketide synthase [Mycobacterium asiaticum]	WP_065146316
KS_703	1259	SPV_cluster_134	53.412	425	1.06E-137	Type I polyketide synthase [Mycolicibacterium vanbaalenii]	WP_011778276
KS_706	1259	SPV_cluster_035	55.319	423	4.14E-138	Type I polyketide synthase [Mycobacterium parmense]	WP_085270770
KS_709	1265	SPV_cluster_071	54.953	424	1.27E-134	Type I polyketide synthase [Mycobacterium asiaticum]	WP_065157110
KS_710	1262	SPV_cluster_114	54.481	424	6.33E-140	Type I polyketide synthase [Mycobacterium sp. EPa45]	WP_047332984
KS_711	1259	SPV_cluster_084	54.245	424	1.88E-132	Type I polyketide synthase [Mycolicibacterium austroafricanum]	WP_051558318
KS_713	1259	SPV_cluster_146	57.309	431	5.25E-142	Type I polyketide synthase [Mycolicibacterium vanbaalenii]	WP_011778276
KS_716	1259	SPV_cluster_020	53.538	424	8.87E-137	Type I polyketide synthase [Mycobacterium colombiense]	WP_077092494
KS_717	1265	SPV_cluster_047	55.529	425	7.06E-140	Type I polyketide synthase [Mycobacterium parmense]	WP_085270770
KS_718	1259	SPV_cluster_154	56.974	423	1.83E-142	Type I polyketide synthase [Mycolicibacterium vanbaalenii]	WP_011778276
KS_721	1211	SPV_cluster_149	53.382	414	5.56E-132	Type I polyketide synthase [Mycobacterium gordonae]	WP_065161832
KS_722	1259	SPV_cluster_245	57.981	426	2.27E-145	Type I polyketide synthase [Mycolicibacterium austroafricanum]	WP_105389463
KS_725	1259	SPV_cluster_150	57.176	425	4.59E-142	Type I polyketide synthase [Mycolicibacterium austroafricanum]	WP_105389463
KS_731	1262	SPV_cluster_151	55.164	426	1.85E-139	Type I polyketide synthase [Mycolicibacterium vanbaalenii]	WP_011778276

KS_734	1259	SPV_cluster_174	55.399	426	1.18E-137	Type I polyketide synthase [Mycolicibacterium austroafricanum]	WP_105389463
KS_736	1259	SPV_cluster_005	55.189	424	1.26E-144	Type I polyketide synthase [Mycobacterium asiaticum]	WP_065120279
KS_737	1277	SPV_cluster_169	61.982	434	4.74E-161	Type I polyketide synthase [Mycolicibacterium austroafricanum]	WP_105389463
KS_739	1259	SPV_cluster_102	56.674	427	3.95E-144	Type I polyketide synthase [Mycolicibacterium vanbaalenii]	WP_011778276
KS_740	1262	SPV_cluster_246	55.189	424	7.32E-136	Type I polyketide synthase [Mycobacterium sp. 852002-51613_SCH5001154]	WP_067261613
KS_742	1262	SPV_cluster_115	52.57	428	1.55E-128	Type I polyketide synthase [Mycolicibacterium austroafricanum]	WP_051558318
KS_743	1262	SPV_cluster_241	52.103	428	3.94E-127	Type I polyketide synthase [Mycolicibacterium vanbaalenii]	WP_011778276
KS_745	1259	SPV_cluster_078	56.808	426	3.96E-146	Type I polyketide synthase [Mycobacterium colombiense]	WP_076054218
KS_748	1262	SPV_cluster_168	53.037	428	5.24E-130	Type I polyketide synthase [Mycolicibacterium vanbaalenii]	WP_011778276
KS_750	1319	SPV_cluster_008	52.822	443	2.88E-137	Type I polyketide synthase [Mycolicibacterium austroafricanum]	WP_105389463
KS_752	176	SPV_cluster_457	60.714	56	2.03E-15	Type I polyketide synthase [Streptomyces sp. NBS 14/10]	WP_089509394
KS_753	1262	SPV_cluster_030	61.939	423	5.53E-157	Type I polyketide synthase [Mycolicibacterium austroafricanum]	WP_105389463
KS_756	1250	SPV_cluster_030	59.155	426	6.74E-151	Type I polyketide synthase [Mycolicibacterium austroafricanum]	WP_105389463
KS_760	1268	SPV_cluster_029	60.748	428	7.57E-152	Type I polyketide synthase [Mycolicibacterium austroafricanum]	WP_105389463
KS_762	1277	SPV_cluster_106	58.156	423	1.25E-144	Type I polyketide synthase [Mycobacterium sp. IS-1744]	WP_064399965

Table S14. BLAST search of the BGCs identified from the metagenome of *Clathria reinwardti* against the MiBIG database

BGC ID	Predicted BGC type	MiBIG Protein	MiBIG Cluster	MiBIG Product	Identity (%)	Coverage (%)	BLAST Score	E-value
CLR_cluster_008	Arylpolyene	AAO39098	BGC0000956	Andrimid	34	63.7	314	5.0E-85
CLR_cluster_032	Arylpolyene	ADI58650	BGC0000187	Asukamycin	40	91.0	214	2.8E-55
CLR_cluster_044	Arylpolyene	ANY57968	BGC0001369	Metatricycloene	34	91.9	321	3.1E-87
CLR_cluster_070	Arylpolyene	AAW85352	BGC0000837	APE_Vf	38	97.8	240	2.6E-63
CLR_cluster_081	Arylpolyene	ADI58650	BGC0000187	Asukamycin	40	61.6	212	1.6E-54
CLR_cluster_102	Arylpolyene	AAW85355	BGC0000837	APE_Vf	57	100.0	424	1.5E-118
CLR_cluster_137	Arylpolyene	AHA12083	BGC0001172	Chlorizidine_A	35	99.5	177	3.7E-44
CLR_cluster_221	Arylpolyene	CAAN79651	BGC0000836	APE_Ec	42	98.2	248	1.6E-65
CLR_cluster_028	Bacteriocin	-	-	-	-	-	-	-
CLR_cluster_037	Bacteriocin	ACA04490	BGC0000477	Patellin_2/patellin_3	40	103.4	528	1.5E-149
CLR_cluster_063	Bacteriocin	BAE46919	BGC0000565	Goadsporin	44	95.7	569	8.0E-162
CLR_cluster_067	Bacteriocin	CCH92967	BGC0000483	Aeruginosamide	41	101.6	539	5.2E-153
CLR_cluster_092	Bacteriocin	BAE46919	BGC0000565	Goadsporin	44	96.2	558	1.1E-158
CLR_cluster_119	Bacteriocin	CAO82084	BGC0000473	Microcyclamide	42	100.9	533	4.8E-151
CLR_cluster_120	Bacteriocin	ZP_00672901	BGC0000481	Trichamide	39	102.8	515	1.3E-145
CLR_cluster_169	Bacteriocin	-	-	-	-	-	-	-
CLR_cluster_170	Bacteriocin	BAP82649	BGC0001148	Pheganomycin	42	94.0	290	2.8E-78
CLR_cluster_195	Bacteriocin	BAE46919	BGC0000565	Goadsporin	41	98.7	534	2.8E-151
CLR_cluster_274	Bacteriocin	-	-	-	-	-	-	-
CLR_cluster_280	Bacteriocin	AAY21153	BGC0000475	Patellamide_A/patellamide_C	33	106.4	187	3.1E-47
CLR_cluster_001	Cf_fatty_acid	ALV82456	BGC0001382	Svaricin	46	98.8	349	5.3E-96
CLR_cluster_006	Cf_fatty_acid	ALV82456	BGC0001382	Svaricin	48	99.3	379	3.6E-105
CLR_cluster_007	Cf_fatty_acid	LV82456	BGC0001382	Svaricin	44	99.0	354	2.1E-97
CLR_cluster_009	Cf_fatty_acid	AIL50179	BGC0000213	Colabomycin	27	103.6	89	1.2E-17
CLR_cluster_012	Cf_fatty_acid	ALV82456	BGC0001382	Svaricin	48	99.3	376	3.1E-104
CLR_cluster_013	Cf_fatty_acid	ALV82456	BGC0001382	Svaricin	48	98.3	385	6.7E-107
CLR_cluster_015	Cf_fatty_acid	ALV82456	BGC0001382	Svaricin	47	98.5	362	7.9E-100
CLR_cluster_017	Cf_fatty_acid	CAA16177	BGC0001063	Undecylprodigiosin	44	103.1	279	6.4E-75
CLR_cluster_022	Cf_fatty_acid	ALV82456	BGC0001382	Svaricin	49	99.0	371	1.0E-102

CLR_cluster_023	Cf_fatty_acid	CAA16179	BGC0001063	Undecylprodigiosin	45	97.6	264	1.8E-70
CLR_cluster_025	Cf_fatty_acid	ALV82456	BGC0001382	Svaricin	48	97.4	367	2.5E-101
CLR_cluster_030	Cf_fatty_acid	ALV82456	BGC0001382	Svaricin	50	98.3	399	4.6E-111
CLR_cluster_031	Cf_fatty_acid	CAA16179	BGC0001063	Undecylprodigiosin	47	91.9	275	1.1E-73
CLR_cluster_041	Cf_fatty_acid	ALV82456	BGC0001382	Svaricin	46	100.0	366	4.2E-101
CLR_cluster_043	Cf_fatty_acid	AJO72736	BGC0001381	Brasilinolide	28	46.6	62	1.4E-09
CLR_cluster_045	Cf_fatty_acid	ALV82456	BGC0001382	Svaricin	47	98.3	367	2.5E-101
CLR_cluster_046	Cf_fatty_acid	CAA16179	BGC0001063	Undecylprodigiosin	46	94.8	269	5.9E-72
CLR_cluster_049	Cf_fatty_acid	ALV82456	BGC0001382	Svaricin	46	98.8	366	4.2E-101
CLR_cluster_060	Cf_fatty_acid	ALV82456	BGC0001382	Svaricin	46	97.6	372	6.0E-103
CLR_cluster_064	Cf_fatty_acid	ALV82456	BGC0001382	Svaricin	48	98.5	374	1.2E-103
CLR_cluster_065	Cf_fatty_acid	ALV82456	BGC0001382	Svaricin	49	99.3	364	1.2E-100
CLR_cluster_069	Cf_fatty_acid	AHF22848	BGC0000091	Marineosin	46	103.8	305	8.4E-83
CLR_cluster_072	Cf_fatty_acid	ALV82456	BGC0001382	Svaricin	48	98.4	376	4.2E-104
CLR_cluster_077	Cf_fatty_acid	CAA16179	BGC0001063	Undecylprodigiosin	35	84.2	161	1.4E-39
CLR_cluster_079	Cf_fatty_acid	ALV82456	BGC0001382	Svaricin	50	98.3	370	2.3E-102
CLR_cluster_082	Cf_fatty_acid	ALV82456	BGC0001382	Svaricin	47	99.8	382	7.3E-106
CLR_cluster_085	Cf_fatty_acid	ALV82456	BGC0001382	Svaricin	48	98.0	375	9.0E-104
CLR_cluster_086	Cf_fatty_acid	ALV82456	BGC0001382	Svaricin	50	98.8	387	1.8E-107
CLR_cluster_096	Cf_fatty_acid	ALV82456	BGC0001382	Svaricin	50	98.3	370	2.3E-102
CLR_cluster_097	Cf_fatty_acid	ALV82456	BGC0001382	Svaricin	46	99.0	362	7.9E-100
CLR_cluster_098	Cf_fatty_acid	AHA12083	BGC0001172	Chlorizidine_A	46	97.1	309	8.0E-84
CLR_cluster_101	Cf_fatty_acid	ALV82456	BGC0001382	Svaricin	46	98.1	365	7.2E-101
CLR_cluster_104	Cf_fatty_acid	ALV82456	BGC0001382	Svaricin	49	98.8	377	1.8E-104
CLR_cluster_109	Cf_fatty_acid	-	-	-	-	-	-	-
CLR_cluster_110	Cf_fatty_acid	ABQ04139	BGC0000838	Flexirubin	40	100.5	294	1.5E-79
CLR_cluster_112	Cf_fatty_acid	ALV82456	BGC0001382	Svaricin	50	98.2	367	1.8E-101
CLR_cluster_113	Cf_fatty_acid	CAA16179	BGC0001063	Undecylprodigiosin	46	97.0	269	7.4E-72
CLR_cluster_114	Cf_fatty_acid	AHF22848	BGC0000091	Marineosin	47	103.0	306	4.9E-83
CLR_cluster_118	Cf_fatty_acid	CAA16177	BGC0001063	Undecylprodigiosin	44	102.8	297	2.3E-80
CLR_cluster_121	Cf_fatty_acid	ALV82456	BGC0001382	Svaricin	49	98.8	395	6.4E-110
CLR_cluster_122	Cf_fatty_acid	ALV82456	BGC0001382	Svaricin	46	99.0	359	6.7E-99

CLR_cluster_126	Cf_fatty_acid	ABQ04139	BGC0000838	Flexirubin	40	100.7	292	7.6E-79
CLR_cluster_127	Cf_fatty_acid	ACU59351	BGC0000839	Flexirubin	42	101.5	272	6.1E-73
CLR_cluster_128	Cf_fatty_acid	ALV82456	BGC0001382	Svaricin	45	99.0	345	1.0E-94
CLR_cluster_134	Cf_fatty_acid	CAA16177	BGC0001063	Undecylprodigiosin	52	98.8	348	1.2E-95
CLR_cluster_138	Cf_fatty_acid	AEK75497	BGC0000001	Abyssomicin	26	85.7	61	3.1E-09
CLR_cluster_139	Cf_fatty_acid	ALV82456	BGC0001382	Svaricin	30	90.0	139	1.1E-32
CLR_cluster_140	Cf_fatty_acid	AHF22848	BGC0000091	Marineosin	44	105.6	289	8.0E-78
CLR_cluster_141	Cf_fatty_acid	ALV82456	BGC0001382	Svaricin	47	99.1	302	8.2E-82
CLR_cluster_143	Cf_fatty_acid	ALV82456	BGC0001382	Svaricin	48	100.2	363	3.6E-100
CLR_cluster_145	Cf_fatty_acid	ALV82456	BGC0001382	Svaricin	48	99.1	368	1.1E-101
CLR_cluster_147	Cf_fatty_acid	ALV82456	BGC0001382	Svaricin	47	98.1	369	6.5E-102
CLR_cluster_148	Cf_fatty_acid	CAA16179	BGC0001063	Undecylprodigiosin	40	87.1	225	7.9E-59
CLR_cluster_156	Cf_fatty_acid	ALV82456	BGC0001382	Svaricin	49	95.6	349	7.3E-96
CLR_cluster_162	Cf_fatty_acid	ALV82456	BGC0001382	Svaricin	47	98.1	361	1.0E-99
CLR_cluster_165	Cf_fatty_acid	ALV82456	BGC0001382	Svaricin	47	98.3	373	3.4E-103
CLR_cluster_166	Cf_fatty_acid	ALV82456	BGC0001382	Svaricin	48	100.2	367	2.5E-101
CLR_cluster_179	Cf_fatty_acid	CAA16177	BGC0001063	Undecylprodigiosin	50	99.3	386	3.0E-107
CLR_cluster_184	Cf_fatty_acid	AHA12083	BGC0001172	Chlorizidine_A	49	99.3	356	5.9E-98
CLR_cluster_190	Cf_fatty_acid	ALV82456	BGC0001382	Svaricin	47	97.8	372	7.6E-103
CLR_cluster_191	Cf_fatty_acid	ALV82456	BGC0001382	Svaricin	45	100.2	361	1.8E-99
CLR_cluster_193	Cf_fatty_acid	ALV82456	BGC0001382	Svaricin	46	98.5	346	5.9E-95
CLR_cluster_200	Cf_fatty_acid	ALV82456	BGC0001382	Svaricin	47	97.7	363	4.8E-100
CLR_cluster_201	Cf_fatty_acid	ALV82456	BGC0001382	Svaricin	48	97.4	370	2.2E-102
CLR_cluster_204	Cf_fatty_acid	ALV82456	BGC0001382	Svaricin	50	97.9	375	9.3E-104
CLR_cluster_214	Cf_fatty_acid	AHF22848	BGC0000091	Marineosin	47	102.5	309	4.4E-84
CLR_cluster_220	Cf_fatty_acid	ALV82456	BGC0001382	Svaricin	45	99.3	356	5.6E-98
CLR_cluster_223	Cf_fatty_acid	CAA16179	BGC0001063	Undecylprodigiosin	46	92.6	254	2.0E-67
CLR_cluster_224	Cf_fatty_acid	ALV82456	BGC0001382	Svaricin	46	99.3	355	9.7E-98
CLR_cluster_226	Cf_fatty_acid	CAA16179	BGC0001063	Undecylprodigiosin	42	90.7	235	7.5E-62
CLR_cluster_227	Cf_fatty_acid	CCP20057	BGC0001119	Divergolide	43	97.3	231	1.3E-60
CLR_cluster_228	Cf_fatty_acid	CAA16177	BGC0001063	Undecylprodigiosin	49	97.4	366	3.2E-101
CLR_cluster_230	Cf_fatty_acid	ABQ04139	BGC0000838	Flexirubin	41	99.0	284	2.7E-76

CLR_cluster_231	Cf_fatty_acid	ALV82456	BGC0001382	Svaricin	46	97.4	368	1.1E-101
CLR_cluster_232	Cf_fatty_acid	ALV82456	BGC0001382	Svaricin	43	99.5	282	7.3E-76
CLR_cluster_241	Cf_fatty_acid	CAA16179	BGC0001063	Undecylprodigiosin	50	98.8	272	5.1E-73
CLR_cluster_251	Cf_fatty_acid	CAA16177	BGC0001063	Undecylprodigiosin	48	98.8	275	1.0E-73
CLR_cluster_252	Cf_fatty_acid	ACA34361	BGC0001152	Fusaricidin	51	98.6	334	1.2E-91
CLR_cluster_253	Cf_fatty_acid	ALV82456	BGC0001382	Svaricin	38	101.2	284	2.7E-76
CLR_cluster_257	Cf_fatty_acid	ABQ04139	BGC0000838	Flexirubin	41	100.7	294	2.0E-79
CLR_cluster_261	Cf_fatty_acid	ALV82456	BGC0001382	Svaricin	44	96.2	339	5.6E-93
CLR_cluster_262	Cf_fatty_acid	ALV82456	BGC0001382	Svaricin	48	97.4	299	6.8E-81
CLR_cluster_264	Cf_fatty_acid	AHF22848	BGC0000091	Marineosin	45	103.1	303	4.1E-82
CLR_cluster_275	Cf_fatty_acid	ALV82456	BGC0001382	Svaricin	40	98.1	268	1.0E-71
CLR_cluster_277	Cf_fatty_acid	ALV82456	BGC0001382	Svaricin	48	96.6	327	2.0E-89
CLR_cluster_281	Cf_fatty_acid	ALV82456	BGC0001382	Svaricin	50	98.9	322	4.5E-88
CLR_cluster_010	Ladderane	AAV66110	BGC0000992	Fusaridione_A	31	65.4	96	1.1E-19
CLR_cluster_042	Ladderane	CAL48955	BGC0001343	Aurachin	39	98.3	258	1.6E-68
CLR_cluster_105	Ladderane	ANY57968	BGC0001369	Metatricycloene	38	64.0	239	9.2E-63
CLR_cluster_187	Lantipeptide	ACR33053	BGC0000495	Actagardine	30	100.3	267	6.5E-71
CLR_cluster_215	Lantipeptide	WP_006126651	BGC0000554	SRO15-3108	31	97.8	256	1.3E-67
CLR_cluster_011	Other	ABF88003	BGC0000871	VEPE/AEPE/TG-1	30	104.8	594	2.5E-169
CLR_cluster_018	Other	ABF88003	BGC0000871	VEPE/AEPE/TG-1	33	102.3	635	1.3E-181
CLR_cluster_036	Other	ACR50797	BGC0000163	Tetronasin	37	62.8	337	3.6E-92
CLR_cluster_051	Other	ABF87031	BGC0000393	Myxoprincomide	42	53.9	364	5.2E-100
CLR_cluster_054	Other	ABF87031	BGC0000393	Myxoprincomide	41	54.9	354	4.1E-97
CLR_cluster_083	Other	ABF88003	BGC0000871	VEPE/AEPE/TG-1	29	107.4	285	2.6E-76
CLR_cluster_090	Other	ABF88003	BGC0000871	VEPE/AEPE/TG-1	30	104.7	478	2.0E-134
CLR_cluster_094	Other	AEU11005	BGC0001029	Nostophycin	29	91.6	420	7.0E-117
CLR_cluster_111	Other	ABF88003	BGC0000871	VEPE/AEPE/TG-1	34	99.2	447	4.0E-125
CLR_cluster_142	Other	ABF87031	BGC0000393	Myxoprincomide	37	59.4	298	3.5E-80
CLR_cluster_151	Other	CAQ71826	BGC0001189	Taiwachelin	41	81.1	318	1.4E-86
CLR_cluster_164	Other	ABF88003	BGC0000871	VEPE/AEPE/TG-1	30	96.6	283	7.4E-76
CLR_cluster_182	Other	ABF88003	BGC0000871	VEPE/AEPE/TG-1	43	100.6	944	1.5E-274
CLR_cluster_196	Other	ABF88003	BGC0000871	VEPE/AEPE/TG-1	30	71.0	257	8.4E-68

CLR_cluster_238	Other	ABF88003	BGC0000871	VEPE/AEPE/TG-1	32	96.0	343	5.1E-94
CLR_cluster_258	Other	CAQ71826	BGC0001189	Taiwachelin	40	77.0	275	1.3E-73
CLR_cluster_259	Other	CAQ71826	BGC0001189	Taiwachelin	40	73.4	250	3.2E-66
CLR_cluster_273	Other	ABF88003	BGC0000871	VEPE/AEPE/TG-1	27	104.4	106	1.0E-22
CLR_cluster_276	Other	CAI94682	BGC0000141	Rubradirin	37	52.3	127	3.1E-29
CLR_cluster_158	Phosphonate	ADB43073	BGC0000926	Rhizocticin	50	51.9	280	4.0E-75
CLR_cluster_171	Phosphonate	ADB43073	BGC0000926	Rhizocticin	48	62.1	255	1.1E-67
CLR_cluster_209	Phosphonate	ADB43073	BGC0000926	Rhizocticin	43	54.6	249	7.5E-66
CLR_cluster_240	Phosphonate	ADB43073	BGC0000926	Rhizocticin	44	54.9	250	5.7E-66
CLR_cluster_267	Phosphonate	ADB43073	BGC0000926	Rhizocticin	48	54.6	257	3.2E-68
CLR_cluster_002	Terpene	NP_199612	BGC0000670	Thalianol	32	58.9	329	1.2E-89
CLR_cluster_003	Terpene	CAB39693	BGC0000663	Hopene	40	56.7	135	1.4E-31
CLR_cluster_004	Terpene	-	-	-	-	-	-	-
CLR_cluster_016	Terpene	-	-	-	-	-	-	-
CLR_cluster_019	Terpene	BAG16278	BGC0000632	Brasilicardin_A	33	32.6	76	1.6E-13
CLR_cluster_033	Terpene	NP_199612	BGC0000670	Thalianol	32	59.0	319	1.2E-86
CLR_cluster_035	Terpene	CAB39697	BGC0000663	Hopene	42	90.3	520	2.3E-147
CLR_cluster_038	Terpene	CAB39697	BGC0000663	Hopene	55	98.9	727	1.2E-209
CLR_cluster_039	Terpene	CAB39693	BGC0000663	Hopene	47	89.9	257	2.2E-68
CLR_cluster_052	Terpene	AMY15074	BGC0001339	Squalestatin_S1	26	83.1	90	5.0E-18
CLR_cluster_061	Terpene	CAB39697	BGC0000663	Hopene	32	94.0	153	5.8E-37
CLR_cluster_071	Terpene	-	-	-	-	-	-	-
CLR_cluster_073	Terpene	CAB39697	BGC0000663	Hopene	59	97.5	651	1.2E-186
CLR_cluster_074	Terpene	-	-	-	-	-	-	-
CLR_cluster_078	Terpene	NP_199612	BGC0000670	Thalianol	33	62.0	318	3.4E-86
CLR_cluster_084	Terpene	-	-	-	-	-	-	-
CLR_cluster_088	Terpene	-	-	-	-	-	-	-
CLR_cluster_091	Terpene	NP_199612	BGC0000670	Thalianol	32	60.4	321	5.5E-87
CLR_cluster_095	Terpene	NP_199612	BGC0000670	Thalianol	32	58.5	319	1.6E-86
CLR_cluster_099	Terpene	-	-	-	-	-	-	-
CLR_cluster_100	Terpene	-	-	-	-	-	-	-
CLR_cluster_115	Terpene	-	-	-	-	-	-	-

CLR_cluster_116	Terpene	-	-	-	-	-	-	-
CLR_cluster_129	Terpene	CAB39697	BGC0000663	Hopene	38	94.5	473	4.1E-133
CLR_cluster_150	Terpene	-	-	-	-	-	-	-
CLR_cluster_152	Terpene	CAB39697	BGC0000663	Hopene	42	99.1	412	8.8E-115
CLR_cluster_157	Terpene	-	-	-	-	-	-	-
CLR_cluster_167	Terpene	NP_199612	BGC0000670	Thalianol	32	87.4	310	5.1E-84
CLR_cluster_168	Terpene	chr3.CM0292.40.r2.m	BGC0001317	Lupeol	34	100.5	366	8.3E-101
CLR_cluster_172	Terpene	CAB39697	BGC0000663	Hopene	40	92.8	406	4.9E-113
CLR_cluster_178	Terpene	-	-	-	-	-	-	-
CLR_cluster_185	Terpene	-	-	-	-	-	-	-
CLR_cluster_186	Terpene	chr3.CM0292.40.r2.m	BGC0001317	Lupeol	35	60.4	384	5.1E-106
CLR_cluster_188	Terpene	CAB39697	BGC0000663	Hopene	49	97.9	623	3.8E-178
CLR_cluster_189	Terpene	chr3.CM0292.40.r2.m	BGC0001317	Lupeol	36	96.1	220	2.8E-57
CLR_cluster_198	Terpene	-	-	-	-	-	-	-
CLR_cluster_199	Terpene	-	-	-	-	-	-	-
CLR_cluster_205	Terpene	CAB39697	BGC0000663	Hopene	37	92.3	299	6.8E-81
CLR_cluster_207	Terpene	NP_199612	BGC0000670	Thalianol	31	56.9	125	2.0E-28
CLR_cluster_210	Terpene	ABP56869	BGC0001087	Sioxanthin	29	71.5	66	5.7E-11
CLR_cluster_211	Terpene	-	-	-	-	-	-	-
CLR_cluster_213	Terpene	-	-	-	-	-	-	-
CLR_cluster_216	Terpene	chr3.CM0292.40.r2.m	BGC0001317	Lupeol	35	95.3	395	1.1E-109
CLR_cluster_217	Terpene	CAB39697	BGC0000663	Hopene	48	98.5	614	1.4E-175
CLR_cluster_229	Terpene	-	-	-	-	-	-	-
CLR_cluster_233	Terpene	-	-	-	-	-	-	-
CLR_cluster_236	Terpene	CAB39697	BGC0000663	Hopene	38	92.7	341	1.7E-93
CLR_cluster_237	Terpene	CAB39697	BGC0000663	Hopene	44	99.6	425	1.2E-118
CLR_cluster_239	Terpene	CAB39692	BGC0000663	Hopene	39	39.3	168	2.7E-41
CLR_cluster_244	Terpene	NP_199612	BGC0000670	Thalianol	31	94.0	243	4.1E-64
CLR_cluster_254	Terpene	-	-	-	-	-	-	-
CLR_cluster_256	Terpene	-	-	-	-	-	-	-
CLR_cluster_260	Terpene	AED94049	BGC0001314	Tirucalla	30	103.2	289	6.7E-78
CLR_cluster_266	Terpene	CAB39697	BGC0000663	Hopene	59	98.5	485	4.6E-137

CLR_cluster_269	Terpene	-	-	-	-	-	-	-
CLR_cluster_271	Terpene	NP_199612	BGC0000670	Thalianol	36	84.3	177	3.3E-44
CLR_cluster_272	Terpene	CAB39697	BGC0000663	Hopene	47	100.9	379	6.4E-105
CLR_cluster_282	Terpene	AAB31139	BGC0000647	Carotenoid	36	76.0	144	2.1E-34
CLR_cluster_048	TransAT-Type I PKS	AIW82279	BGC0001125	Puwainaphycins	36	78.3	1154	0.0E+00
CLR_cluster_068	TransAT-Type I PKS	AIW82279	BGC0001125	Puwainaphycins	37	80.0	1342	0.0E+00
CLR_cluster_005	Type I PKS	AIW82279	BGC0001125	Puwainaphycins	37	76.7	1513	0.0E+00
CLR_cluster_014	Type I PKS	AAF26921	BGC0000988	Epothilone	38	101.7	1287	0.0E+00
CLR_cluster_020	Type I PKS	AHA38199	BGC0000069	Gephyronic_acid	38	48.1	1404	0.0E+00
CLR_cluster_021	Type I PKS	AIW82279	BGC0001125	Puwainaphycins	39	102.0	1497	0.0E+00
CLR_cluster_024	Type I PKS	CAQ18828	BGC0000954	Ajudazol	47	98.5	648	1.1E-185
CLR_cluster_026	Type I PKS	AAK19883	BGC0000147	Soraphen	35	61.3	1728	0.0E+00
CLR_cluster_027	Type I PKS	CAQ43075	BGC0000970	Chondrochloren	31	55.5	481	5.1E-135
CLR_cluster_029	Type I PKS	AAS98781	BGC0001001	Jamaicamide	35	99.5	1420	0.0E+00
CLR_cluster_034	Type I PKS	AIW82279	BGC0001125	Puwainaphycins	37	75.2	1498	0.0E+00
CLR_cluster_040	Type I PKS	AIW82279	BGC0001125	Puwainaphycins	38	77.5	1467	0.0E+00
CLR_cluster_047	Type I PKS	AIW82279	BGC0001125	Puwainaphycins	36	48.3	1308	0.0E+00
CLR_cluster_050	Type I PKS	CAQ18828	BGC0000954	Ajudazol	42	84.3	535	1.2E-151
CLR_cluster_053	Type I PKS	BAG17643	BGC0001043	SGR_PTMs	33	52.8	544	8.4E-154
CLR_cluster_055	Type I PKS	AGC45622	BGC0001394	Phenalamide	34	49.3	529	1.6E-149
CLR_cluster_056	Type I PKS	AIW82279	BGC0001125	Puwainaphycins	38	77.0	1512	0.0E+00
CLR_cluster_057	Type I PKS	CAQ18829	BGC0000954	Ajudazol	44	83.0	529	8.3E-150
CLR_cluster_058	Type I PKS	AIW82279	BGC0001125	Puwainaphycins	36	68.7	1376	0.0E+00
CLR_cluster_059	Type I PKS	AAU04878	BGC0000365	Glycopeptidolipid	49	98.4	689	6.0E-198
CLR_cluster_062	Type I PKS	BAG17643	BGC0001043	SGR_PTMs	35	41.2	438	3.5E-122
CLR_cluster_066	Type I PKS	AAU04878	BGC0000365	Glycopeptidolipid	43	88.9	890	3.0E-258
CLR_cluster_075	Type I PKS	AAK73514	BGC0000015	Amphotericin	36	102.3	1636	0.0E+00
CLR_cluster_076	Type I PKS	BAG17643	BGC0001043	SGR_PTMs	35	102.1	512	9.0E-145
CLR_cluster_080	Type I PKS	AAF26921	BGC0000988	Epothilone	38	90.1	895	1.1E-259
CLR_cluster_087	Type I PKS	BAG17643	BGC0001043	SGR_PTMs	35	82.6	541	3.1E-153
CLR_cluster_089	Type I PKS	BAG17643	BGC0001043	SGR_PTMs	35	65.3	515	1.7E-145
CLR_cluster_093	Type I PKS	AIW82279	BGC0001125	Puwainaphycins	38	82.8	1433	0.0E+00

CLR_cluster_103	Type I PKS	ACB46195	BGC0000989	Epothilone	40	69.2	906	6.0E-263
CLR_cluster_106	Type I PKS	BAG17643	BGC0001043	SGR_PTMs	34	82.3	540	4.3E-153
CLR_cluster_107	Type I PKS	BAG17643	BGC0001043	SGR_PTMs	35	48.8	548	4.5E-155
CLR_cluster_108	Type I PKS	BAG17643	BGC0001043	SGR_PTMs	35	97.3	506	6.7E-143
CLR_cluster_117	Type I PKS	BAK64637	BGC0000135	Reveromycin	38	96.4	875	1.2E-253
CLR_cluster_123	Type I PKS	AIW82279	BGC0001125	Puwainaphycins	36	100.9	1116	0.0E+00
CLR_cluster_124	Type I PKS	CAQ18828	BGC0000954	Ajudazol	44	87.7	846	3.8E-245
CLR_cluster_125	Type I PKS	AEU11006	BGC0001029	Nostophycin	39	109.2	567	3.3E-161
CLR_cluster_130	Type I PKS	ABX60162	BGC0000978	Cylindrospermopsin	37	68.6	199	7.9E-51
CLR_cluster_131	Type I PKS	AGC45621	BGC0001394	Phenalamide	46	100.5	798	7.7E-231
CLR_cluster_132	Type I PKS	BAG17643	BGC0001043	SGR_PTMs	36	75.7	547	4.7E-155
CLR_cluster_133	Type I PKS	BAG17643	BGC0001043	SGR_PTMs	35	63.5	542	2.5E-153
CLR_cluster_135	Type I PKS	CAQ18828	BGC0000954	Ajudazol	44	101.4	782	5.1E-226
CLR_cluster_136	Type I PKS	CAQ18829	BGC0000954	Ajudazol	44	83.9	811	1.7E-234
CLR_cluster_144	Type I PKS	AAU04878	BGC0000365	Glycopeptidolipid	47	99.4	679	4.6E-195
CLR_cluster_146	Type I PKS	AIW82279	BGC0001125	Puwainaphycins	37	102.0	1018	9.0E-297
CLR_cluster_149	Type I PKS	AEE88280	BGC0000976	Curacin	36	100.5	950	2.2E-276
CLR_cluster_153	Type I PKS	AIW82279	BGC0001125	Puwainaphycins	37	99.6	970	2.0E-282
CLR_cluster_154	Type I PKS	AAF26921	BGC0000988	Epothilone	39	100.6	906	3.3E-263
CLR_cluster_155	Type I PKS	AAU04878	BGC0000365	Glycopeptidolipid	44	88.3	577	3.5E-164
CLR_cluster_159	Type I PKS	AAU04878	BGC0000365	Glycopeptidolipid	42	100.7	740	3.6E-213
CLR_cluster_160	Type I PKS	CAQ18829	BGC0000954	Ajudazol	43	97.7	1001	1.4E-291
CLR_cluster_161	Type I PKS	ABW96541	BGC0000159	Tautomycin	39	93.9	865	1.1E-250
CLR_cluster_163	Type I PKS	AIW82279	BGC0001125	Puwainaphycins	35	102.5	821	1.8E-237
CLR_cluster_173	Type I PKS	CAQ18829	BGC0000954	Ajudazol	45	91.3	865	7.6E-251
CLR_cluster_174	Type I PKS	AAU04878	BGC0000365	Glycopeptidolipid	44	100.5	678	8.4E-195
CLR_cluster_175	Type I PKS	AAF62883	BGC0000991	Epothilone	42	91.6	798	1.1E-230
CLR_cluster_176	Type I PKS	BAG17643	BGC0001043	SGR_PTMs	33	90.5	527	4.7E-149
CLR_cluster_177	Type I PKS	BAG17643	BGC0001043	SGR_PTMs	36	96.7	548	1.6E-155
CLR_cluster_180	Type I PKS	AIW82279	BGC0001125	Puwainaphycins	34	103.0	700	5.0E-201
CLR_cluster_181	Type I PKS	AAU04878	BGC0000365	Glycopeptidolipid	37	99.3	539	1.1E-152
CLR_cluster_183	Type I PKS	CAQ18829	BGC0000954	Ajudazol	44	83.8	493	5.8E-139

CLR_cluster_192	Type I PKS	CAQ18828	BGC0000954	Ajudazol	43	100.2	790	1.9E-228
CLR_cluster_194	Type I PKS	AAU04878	BGC0000365	Glycopeptidolipid	43	97.6	718	1.1E-206
CLR_cluster_197	Type I PKS	CAQ18829	BGC0000954	Ajudazol	42	97.7	667	3.1E-191
CLR_cluster_202	Type I PKS	CAQ18834	BGC0000954	Ajudazol	38	101.2	412	1.3E-114
CLR_cluster_203	Type I PKS	AAU04878	BGC0000365	Glycopeptidolipid	44	98.9	742	6.8E-214
CLR_cluster_206	Type I PKS	AGC45620	BGC0001394	Phenalamide	42	86.8	579	5.4E-165
CLR_cluster_208	Type I PKS	CAQ18829	BGC0000954	Ajudazol	43	94.3	536	4.6E-152
CLR_cluster_212	Type I PKS	AAU04878	BGC0000365	Glycopeptidolipid	44	99.3	613	3.2E-175
CLR_cluster_218	Type I PKS	AAU04878	BGC0000365	Glycopeptidolipid	46	99.1	691	1.6E-198
CLR_cluster_219	Type I PKS	CAQ18828	BGC0000954	Ajudazol	46	99.8	646	3.3E-185
CLR_cluster_222	Type I PKS	CAQ18828	BGC0000954	Ajudazol	45	101.2	672	7.5E-193
CLR_cluster_225	Type I PKS	CAQ18829	BGC0000954	Ajudazol	47	101.1	640	3.1E-183
CLR_cluster_234	Type I PKS	CAQ18828	BGC0000954	Ajudazol	45	101.7	558	1.4E-158
CLR_cluster_235	Type I PKS	BAG17643	BGC0001043	SGR_PTMs	35	102.7	368	2.6E-101
CLR_cluster_242	Type I PKS	AGC45620	BGC0001394	Phenalamide	48	98.9	562	8.8E-160
CLR_cluster_245	Type I PKS	AHB82052	BGC0001019	Microsclerodermins	37	101.2	265	1.6E-70
CLR_cluster_246	Type I PKS	AAU04878	BGC0000365	Glycopeptidolipid	46	74.9	374	2.5E-103
CLR_cluster_247	Type I PKS	CBD77732	BGC0000974	Crocacin	40	65.2	280	4.9E-75
CLR_cluster_248	Type I PKS	AEU11005	BGC0001029	Nostophycin	40	93.3	418	1.5E-116
CLR_cluster_249	Type I PKS	AIW82282	BGC0001125	Puwainaphycins	42	79.9	416	4.4E-116
CLR_cluster_250	Type I PKS	AAU04878	BGC0000365	Glycopeptidolipid	39	102.1	402	8.4E-112
CLR_cluster_255	Type I PKS	CAQ18828	BGC0000954	Ajudazol	41	99.2	369	7.3E-102
CLR_cluster_265	Type I PKS	CBD77732	BGC0000974	Crocacin	38	102.0	295	1.1E-79
CLR_cluster_268	Type I PKS	ABX60162	BGC0000978	Cylindrospermopsin	38	101.5	313	4.8E-85
CLR_cluster_278	Type I PKS	AAF62883	BGC0000991	Epothilone	41	117.5	312	5.0E-85
CLR_cluster_279	Type II PKS	ABP54674	BGC0000241	Lomaiviticin	31	101.8	129	9.3E-30
CLR_cluster_243	Type III PKS	BAG17301	BGC0000282	Alkylresorcinol	39	101.5	208	9.1E-54
CLR_cluster_263	Type III PKS	BAG17301	BGC0000282	Alkylresorcinol	40	101.7	227	3.3E-59
CLR_cluster_270	Type III PKS	BAG17301	BGC0000282	Alkylresorcinol	31	97.5	193	3.3E-49

Table S15. BLAST search of the BGCs identified from the metagenome of *Rhabdastrella globostellata* against the MiBIG database

BGC ID	Predicted BGC type	MiBIG Protein	MiBIG Cluster	MiBIG Product	Identity (%)	Coverage (%)	BLAST Score	E-value
RHG_cluster_046	Arylpolyene	AAO39098	BGC0000956	Andrimid	34	63.7	314	5.0E-85
RHG_cluster_061	Arylpolyene	CUI25675	BGC0001353	Thiolactomycin	38	88.2	193	6.1E-49
RHG_cluster_120	Arylpolyene	AAW85355	BGC0000837	APE_Vf	57	100.0	423	2.5E-118
RHG_cluster_121	Arylpolyene	AAW85352	BGC0000837	APE_Vf	38	97.8	240	2.6E-63
RHG_cluster_125	Arylpolyene	AAW85352	BGC0000837	APE_Vf	45	82.9	277	2.5E-74
RHG_cluster_134	Arylpolyene	ANY57968	BGC0001369	Metatricycloene	34	91.9	321	3.1E-87
RHG_cluster_184	Arylpolyene	ADI58650	BGC0000187	Asukamycin	40	61.6	212	1.6E-54
RHG_cluster_231	Arylpolyene	AAN79648	BGC0000836	APE_Ec	57	99.0	498	5.4E-141
RHG_cluster_232	Arylpolyene	AAW85352	BGC0000837	APE_Vf	42	95.6	239	5.9E-63
RHG_cluster_253	Arylpolyene	AHA12083	BGC0001172	Chlorizidine_A	35	99.5	177	2.9E-44
RHG_cluster_005	Bacteriocin	CAO82084	BGC0000473	Microcyclamide	42	100.9	532	8.2E-151
RHG_cluster_021	Bacteriocin	ZP_00672901	BGC0000481	Trichamide	39	99.1	534	2.2E-151
RHG_cluster_024	Bacteriocin	-	-	-	-	-	-	-
RHG_cluster_025	Bacteriocin	ACA04490	BGC0000477	Patellin_2/patellin_3	40	103.4	528	1.5E-149
RHG_cluster_032	Bacteriocin	ZP_00672901	BGC0000481	Trichamide	39	102.8	515	1.0E-145
RHG_cluster_038	Bacteriocin	BAE46919	BGC0000565	Goadsporin	44	96.2	558	1.1E-158
RHG_cluster_083	Bacteriocin	BAE46919	BGC0000565	Goadsporin	44	95.7	569	8.0E-162
RHG_cluster_092	Bacteriocin	BAP82649	BGC0001148	Pheganomycin	39	96.1	435	1.5E-121
RHG_cluster_115	Bacteriocin	CCH92967	BGC0000483	Aeruginosamide	41	101.6	539	5.2E-153
RHG_cluster_139	Bacteriocin	BAE46919	BGC0000565	Goadsporin	44	97.6	556	4.0E-158
RHG_cluster_181	Bacteriocin	CCH92967	BGC0000483	Aeruginosamide	42	101.0	560	3.7E-159
RHG_cluster_189	Bacteriocin	-	-	-	-	-	-	-
RHG_cluster_251	Bacteriocin	-	-	-	-	-	-	-
RHG_cluster_257	Bacteriocin	ACA04483	BGC0000480	Tenuecyclamide_A/C	39	100.8	423	5.8E-118
RHG_cluster_297	Bacteriocin	WP_006126653	BGC0000554	SRO15-3108	36	72.8	414	3.2E-115
RHG_cluster_325	Bacteriocin	-	-	-	-	-	-	-
RHG_cluster_004	Cf_fatty_acid	ALV82456	BGC0001382	Svaricin	48	97.4	367	2.5E-101
RHG_cluster_007	Cf_fatty_acid	ALV82456	BGC0001382	Svaricin	48	99.3	379	3.6E-105
RHG_cluster_009	Cf_fatty_acid	ALV82456	BGC0001382	Svaricin	47	98.5	362	7.9E-100

RHG_cluster_010	Cf_fatty_acid	CAA16179	BGC0001063	Undecylprodigiosin	45	97.6	264	1.8E-70
RHG_cluster_012	Cf_fatty_acid	ALV82456	BGC0001382	Svaricin	46	98.8	349	5.3E-96
RHG_cluster_018	Cf_fatty_acid	ALV82456	BGC0001382	Svaricin	44	99.0	354	2.1E-97
RHG_cluster_020	Cf_fatty_acid	ALV82456	BGC0001382	Svaricin	50	98.1	387	1.8E-107
RHG_cluster_023	Cf_fatty_acid	ALV82456	BGC0001382	Svaricin	46	98.1	364	1.2E-100
RHG_cluster_030	Cf_fatty_acid	AHA12083	BGC0001172	Chlorizidine_A	46	97.1	309	8.0E-84
RHG_cluster_031	Cf_fatty_acid	ALV82456	BGC0001382	Svaricin	50	98.3	398	1.0E-110
RHG_cluster_033	Cf_fatty_acid	ALV82456	BGC0001382	Svaricin	38	101.2	279	6.6E-75
RHG_cluster_035	Cf_fatty_acid	ALV82456	BGC0001382	Svaricin	46	99.3	367	1.9E-101
RHG_cluster_039	Cf_fatty_acid	CAA16177	BGC0001063	Undecylprodigiosin	49	97.4	366	3.2E-101
RHG_cluster_045	Cf_fatty_acid	AIL50179	BGC0000213	Colabomycin	27	103.6	89	1.2E-17
RHG_cluster_049	Cf_fatty_acid	ALV82456	BGC0001382	Svaricin	47	99.8	382	7.3E-106
RHG_cluster_051	Cf_fatty_acid	AHF22848	BGC0000091	Marineosin	45	103.1	303	4.1E-82
RHG_cluster_059	Cf_fatty_acid	CAA16177	BGC0001063	Undecylprodigiosin	44	103.1	279	6.4E-75
RHG_cluster_065	Cf_fatty_acid	ALV82456	BGC0001382	Svaricin	48	98.4	376	4.2E-104
RHG_cluster_068	Cf_fatty_acid	ALV82456	BGC0001382	Svaricin	48	99.3	376	3.1E-104
RHG_cluster_073	Cf_fatty_acid	ABQ04139	BGC0000838	Flexirubin	40	100.5	294	1.5E-79
RHG_cluster_074	Cf_fatty_acid	ALV82456	BGC0001382	Svaricin	41	102.0	245	1.0E-64
RHG_cluster_075	Cf_fatty_acid	ALV82456	BGC0001382	Svaricin	48	98.1	354	1.3E-97
RHG_cluster_077	Cf_fatty_acid	CAA16177	BGC0001063	Undecylprodigiosin	47	96.0	357	2.6E-98
RHG_cluster_078	Cf_fatty_acid	ALV82456	BGC0001382	Svaricin	30	90.0	139	8.7E-33
RHG_cluster_079	Cf_fatty_acid	ALV82456	BGC0001382	Svaricin	39	100.0	269	7.1E-72
RHG_cluster_081	Cf_fatty_acid	ALV82456	BGC0001382	Svaricin	44	98.4	307	3.2E-83
RHG_cluster_084	Cf_fatty_acid	ALV82456	BGC0001382	Svaricin	50	98.3	370	2.3E-102
RHG_cluster_085	Cf_fatty_acid	AHF22848	BGC0000091	Marineosin	46	103.8	305	8.4E-83
RHG_cluster_086	Cf_fatty_acid	ALV82456	BGC0001382	Svaricin	49	98.8	377	1.8E-104
RHG_cluster_089	Cf_fatty_acid	CAA16179	BGC0001063	Undecylprodigiosin	44	94.2	260	3.5E-69
RHG_cluster_095	Cf_fatty_acid	CAA16177	BGC0001063	Undecylprodigiosin	37	87.6	186	4.4E-47
RHG_cluster_097	Cf_fatty_acid	ALV82456	BGC0001382	Svaricin	48	97.7	368	1.1E-101
RHG_cluster_098	Cf_fatty_acid	ALV82456	BGC0001382	Svaricin	46	98.5	346	5.9E-95
RHG_cluster_099	Cf_fatty_acid	CAA16179	BGC0001063	Undecylprodigiosin	41	98.5	243	4.3E-64
RHG_cluster_103	Cf_fatty_acid	ACA34361	BGC0001152	Fusaricidin	50	90.4	345	7.1E-95

RHG_cluster_108	Cf_fatty_acid	ABQ04139	BGC0000838	Flexirubin	41	100.7	294	1.5E-79
RHG_cluster_112	Cf_fatty_acid	CAA16179	BGC0001063	Undecylprodigiosin	45	97.0	265	1.1E-70
RHG_cluster_114	Cf_fatty_acid	ALV82456	BGC0001382	Svaricin	48	98.0	375	9.0E-104
RHG_cluster_122	Cf_fatty_acid	ALV82456	BGC0001382	Svaricin	48	98.5	374	1.2E-103
RHG_cluster_127	Cf_fatty_acid	ALV82456	BGC0001382	Svaricin	48	98.8	370	2.9E-102
RHG_cluster_130	Cf_fatty_acid	ALV82456	BGC0001382	Svaricin	49	99.3	360	2.3E-99
RHG_cluster_133	Cf_fatty_acid	AJO72736	BGC0001381	Brasilinolide	28	46.6	62	1.4E-09
RHG_cluster_138	Cf_fatty_acid	ALV82456	BGC0001382	Svaricin	46	99.0	362	7.9E-100
RHG_cluster_141	Cf_fatty_acid	CAA16179	BGC0001063	Undecylprodigiosin	35	84.4	161	1.4E-39
RHG_cluster_142	Cf_fatty_acid	ALV82456	BGC0001382	Svaricin	48	100.2	363	3.6E-100
RHG_cluster_143	Cf_fatty_acid	ALV82456	BGC0001382	Svaricin	45	99.0	345	1.0E-94
RHG_cluster_144	Cf_fatty_acid	ALV82456	BGC0001382	Svaricin	49	99.3	390	2.1E-108
RHG_cluster_148	Cf_fatty_acid	AHF22848	BGC0000091	Marineosin	40	101.9	267	2.6E-71
RHG_cluster_150	Cf_fatty_acid	AFJ52674	BGC0001073	Kosinostatin	44	99.0	331	1.2E-90
RHG_cluster_151	Cf_fatty_acid	ALV82456	BGC0001382	Svaricin	46	99.0	338	1.2E-92
RHG_cluster_153	Cf_fatty_acid	ABQ04139	BGC0000838	Flexirubin	40	100.7	292	7.6E-79
RHG_cluster_155	Cf_fatty_acid	ALV82456	BGC0001382	Svaricin	49	98.1	349	7.1E-96
RHG_cluster_156	Cf_fatty_acid	ABQ04139	BGC0000838	Flexirubin	42	99.0	287	3.2E-77
RHG_cluster_172	Cf_fatty_acid	ALV82456	BGC0001382	Svaricin	50	96.7	382	5.7E-106
RHG_cluster_173	Cf_fatty_acid	CAA16179	BGC0001063	Undecylprodigiosin	43	97.3	254	1.4E-67
RHG_cluster_175	Cf_fatty_acid	CAA16179	BGC0001063	Undecylprodigiosin	46	95.4	255	1.1E-67
RHG_cluster_176	Cf_fatty_acid	ALV82391	BGC0001370	Lipopeptide_8D1-1/8D1-2	33	50.5	124	4.4E-28
RHG_cluster_178	Cf_fatty_acid	ALV82456	BGC0001382	Svaricin	46	98.1	346	3.5E-95
RHG_cluster_182	Cf_fatty_acid	CBJ83012	BGC0000189	Xenocyloins	50	76.7	458	1.6E-128
RHG_cluster_185	Cf_fatty_acid	ALV82456	BGC0001382	Svaricin	47	98.1	369	6.5E-102
RHG_cluster_186	Cf_fatty_acid	CAA16179	BGC0001063	Undecylprodigiosin	48	96.2	270	2.0E-72
RHG_cluster_188	Cf_fatty_acid	ALV82456	BGC0001382	Svaricin	48	98.3	386	5.1E-107
RHG_cluster_190	Cf_fatty_acid	CAB38890	BGC0000315	Calcium-dependent_antibiotic	43	88.5	231	1.9E-60
RHG_cluster_196	Cf_fatty_acid	CAA16179	BGC0001063	Undecylprodigiosin	40	87.1	224	1.8E-58
RHG_cluster_211	Cf_fatty_acid	CAA16179	BGC0001063	Undecylprodigiosin	47	91.9	274	1.9E-73
RHG_cluster_212	Cf_fatty_acid	AHF22848	BGC0000091	Marineosin	44	105.6	289	8.0E-78
RHG_cluster_214	Cf_fatty_acid	ALV82456	BGC0001382	Svaricin	45	100.2	361	1.8E-99

RHG_cluster_216	Cf_fatty_acid	ALV82456	BGC0001382	Svaricin	48	99.8	365	7.3E-101
RHG_cluster_218	Cf_fatty_acid	ALV82456	BGC0001382	Svaricin	49	98.8	396	2.9E-110
RHG_cluster_220	Cf_fatty_acid	ALV82456	BGC0001382	Svaricin	48	100.2	363	3.6E-100
RHG_cluster_222	Cf_fatty_acid	ALV82456	BGC0001382	Svaricin	48	98.3	366	4.3E-101
RHG_cluster_225	Cf_fatty_acid	ALV82456	BGC0001382	Svaricin	48	99.0	389	6.0E-108
RHG_cluster_227	Cf_fatty_acid	ALV82456	BGC0001382	Svaricin	47	98.8	367	2.4E-101
RHG_cluster_228	Cf_fatty_acid	CAA16179	BGC0001063	Undecylprodigiosin	46	94.8	269	5.9E-72
RHG_cluster_235	Cf_fatty_acid	ALV82456	BGC0001382	Svaricin	46	99.3	367	1.9E-101
RHG_cluster_236	Cf_fatty_acid	CAA16177	BGC0001063	Undecylprodigiosin	44	102.8	297	2.3E-80
RHG_cluster_239	Cf_fatty_acid	ctg1_orf534	BGC0001199	Akaeolide	42	97.1	223	2.8E-58
RHG_cluster_241	Cf_fatty_acid	ACU59351	BGC0000839	Flexirubin	44	100.2	320	3.4E-87
RHG_cluster_244	Cf_fatty_acid	ALV82456	BGC0001382	Svaricin	47	98.1	351	1.4E-96
RHG_cluster_245	Cf_fatty_acid	CAB38890	BGC0000315	Calcium-dependent_antibiotic	42	89.1	229	6.9E-60
RHG_cluster_250	Cf_fatty_acid	ACU59351	BGC0000839	Flexirubin	42	101.5	272	6.1E-73
RHG_cluster_254	Cf_fatty_acid	AEK75497	BGC0000001	Abyssomicin	26	85.7	61	3.1E-09
RHG_cluster_259	Cf_fatty_acid	AHF22848	BGC0000091	Marineosin	46	103.0	303	3.2E-82
RHG_cluster_263	Cf_fatty_acid	ALV82456	BGC0001382	Svaricin	46	99.0	366	4.2E-101
RHG_cluster_264	Cf_fatty_acid	AHA12083	BGC0001172	Chlorizidine_A	45	97.6	305	1.2E-82
RHG_cluster_265	Cf_fatty_acid	ACA34361	BGC0001152	Fusaricidin	49	90.5	343	3.6E-94
RHG_cluster_270	Cf_fatty_acid	-	-	-	-	-	-	-
RHG_cluster_271	Cf_fatty_acid	ALV82456	BGC0001382	Svaricin	47	90.0	354	2.2E-97
RHG_cluster_273	Cf_fatty_acid	AHF22848	BGC0000091	Marineosin	47	102.5	309	4.4E-84
RHG_cluster_274	Cf_fatty_acid	ALV82456	BGC0001382	Svaricin	48	99.0	370	2.9E-102
RHG_cluster_275	Cf_fatty_acid	ACA34361	BGC0001152	Fusaricidin	44	96.4	314	2.2E-85
RHG_cluster_277	Cf_fatty_acid	ALV82456	BGC0001382	Svaricin	50	98.8	387	1.8E-107
RHG_cluster_278	Cf_fatty_acid	ACU59351	BGC0000839	Flexirubin	41	100.2	293	4.5E-79
RHG_cluster_281	Cf_fatty_acid	ALV82456	BGC0001382	Svaricin	47	98.3	357	2.0E-98
RHG_cluster_286	Cf_fatty_acid	CCP20057	BGC0001119	Divergolide	43	97.3	227	1.9E-59
RHG_cluster_292	Cf_fatty_acid	ABQ04139	BGC0000838	Flexirubin	42	101.4	267	1.8E-71
RHG_cluster_293	Cf_fatty_acid	CAA16179	BGC0001063	Undecylprodigiosin	40	98.2	241	1.3E-63
RHG_cluster_301	Cf_fatty_acid	ALV82456	BGC0001382	Svaricin	49	99.0	381	9.7E-106
RHG_cluster_303	Cf_fatty_acid	ABQ04139	BGC0000838	Flexirubin	39	100.2	270	2.4E-72

RHG_cluster_305	Cf_fatty_acid	ALV82456	BGC0001382	Svaricin	49	99.0	371	1.3E-102
RHG_cluster_310	Cf_fatty_acid	ALV82456	BGC0001382	Svaricin	47	99.5	329	5.3E-90
RHG_cluster_313	Cf_fatty_acid	ALV82456	BGC0001382	Svaricin	48	97.4	370	2.2E-102
RHG_cluster_315	Cf_fatty_acid	ALV82456	BGC0001382	Svaricin	48	98.4	326	5.6E-89
RHG_cluster_317	Cf_fatty_acid	ALV82456	BGC0001382	Svaricin	50	98.3	356	3.4E-98
RHG_cluster_319	Cf_fatty_acid	CAA16177	BGC0001063	Undecylprodigiosin	50	99.3	386	3.0E-107
RHG_cluster_321	Cf_fatty_acid	ACA34361	BGC0001152	Fusaricidin	52	95.4	368	9.9E-102
RHG_cluster_324	Cf_fatty_acid	ALV82456	BGC0001382	Svaricin	44	99.3	347	2.6E-95
RHG_cluster_328	Cf_fatty_acid	CAC36759	BGC0000914	Methylenomycin	41	97.6	232	9.9E-61
RHG_cluster_332	Cf_fatty_acid	ALV82456	BGC0001382	Svaricin	43	99.7	312	6.9E-85
RHG_cluster_334	Cf_fatty_acid	ALV82456	BGC0001382	Svaricin	46	99.3	349	6.8E-96
RHG_cluster_343	Cf_fatty_acid	ACA34361	BGC0001152	Fusaricidin	51	95.6	351	1.2E-96
RHG_cluster_344	Cf_fatty_acid	ALV82456	BGC0001382	Svaricin	48	98.8	373	3.4E-103
RHG_cluster_347	Cf_fatty_acid	ALV82456	BGC0001382	Svaricin	47	98.1	361	1.0E-99
RHG_cluster_348	Cf_fatty_acid	ALV82456	BGC0001382	Svaricin	42	98.3	320	2.7E-87
RHG_cluster_352	Cf_fatty_acid	ABQ04139	BGC0000838	Flexirubin	41	100.6	251	1.2E-66
RHG_cluster_355	Cf_fatty_acid	ACA34361	BGC0001152	Fusaricidin	50	100.0	326	3.0E-89
RHG_cluster_359	Cf_fatty_acid	ALV82456	BGC0001382	Svaricin	49	98.6	363	3.2E-100
RHG_cluster_360	Cf_fatty_acid	AEE65468	BGC0000223	Fluostatin	42	99.5	304	1.5E-82
RHG_cluster_365	Cf_fatty_acid	ACA34361	BGC0001152	Fusaricidin	46	98.8	286	3.5E-77
RHG_cluster_044	Ladderane	CAL48955	BGC0001343	Aurachin	38	97.4	254	1.8E-67
RHG_cluster_111	Ladderane	ANY57968	BGC0001369	Metatricycloene	38	64.0	239	9.2E-63
RHG_cluster_116	Ladderane	ANY57968	BGC0001369	Metatricycloene	37	72.5	234	3.9E-61
RHG_cluster_132	Ladderane	CAL48955	BGC0001343	Aurachin	39	98.3	258	1.6E-68
RHG_cluster_093	Lantipeptide	WP_006126651	BGC0000554	SRO15-3108	29	105.2	279	1.3E-74
RHG_cluster_001	Other	ABF88003	BGC0000871	VEPE/AEPE/TG-1	30	104.8	594	2.5E-169
RHG_cluster_022	Other	ABF88003	BGC0000871	VEPE/AEPE/TG-1	26	106.5	232	2.5E-60
RHG_cluster_029	Other	ABF88003	BGC0000871	VEPE/AEPE/TG-1	29	108.1	285	2.0E-76
RHG_cluster_048	Other	ABF88003	BGC0000871	VEPE/AEPE/TG-1	29	106.6	508	2.3E-143
RHG_cluster_064	Other	ABF88003	BGC0000871	VEPE/AEPE/TG-1	30	96.6	283	7.4E-76
RHG_cluster_066	Other	BAB69698	BGC0001098	Iturin	37	101.1	445	8.5E-125
RHG_cluster_070	Other	BAE93741	BGC0000164	Tetronomycin	38	60.6	334	3.1E-91

RHG_cluster_082	Other	ACR50797	BGC0000163	Tetronasin	39	63.7	356	7.5E-98
RHG_cluster_088	Other	ABF87031	BGC0000393	Myxoprincomide	42	53.9	364	5.2E-100
RHG_cluster_110	Other	AAO23334	BGC0000397	Nostocyclopeptide	33	97.7	464	2.7E-130
RHG_cluster_126	Other	CAQ71826	BGC0001189	Taiwachelin	40	57.7	345	2.3E-94
RHG_cluster_131	Other	ACR50797	BGC0000163	Tetronasin	37	63.6	329	1.7E-89
RHG_cluster_171	Other	ABF88003	BGC0000871	VEPE/AEPE/TG-1	34	99.2	447	3.0E-125
RHG_cluster_187	Other	ABF88003	BGC0000871	VEPE/AEPE/TG-1	32	76.1	212	2.7E-54
RHG_cluster_205	Other	CAA16183	BGC0001063	Undecylprodigiosin	38	56.6	299	1.6E-80
RHG_cluster_210	Other	ABF88003	BGC0000871	VEPE/AEPE/TG-1	42	100.8	565	1.1E-160
RHG_cluster_223	Other	ABF87031	BGC0000393	Myxoprincomide	41	54.9	354	4.1E-97
RHG_cluster_260	Other	ABF88003	BGC0000871	VEPE/AEPE/TG-1	30	89.0	260	7.9E-69
RHG_cluster_287	Other	ABF87031	BGC0000393	Myxoprincomide	42	60.3	363	6.1E-100
RHG_cluster_300	Other	ABF88003	BGC0000871	VEPE/AEPE/TG-1	32	78.5	288	2.5E-77
RHG_cluster_312	Other	BAK64635	BGC0000135	Reveromycin	40	60.5	356	1.0E-97
RHG_cluster_329	Other	ABF88003	BGC0000871	VEPE/AEPE/TG-1	36	95.0	422	9.6E-118
RHG_cluster_333	Other	ABF88003	BGC0000871	VEPE/AEPE/TG-1	34	101.4	407	4.0E-113
RHG_cluster_339	Other	ABF87031	BGC0000393	Myxoprincomide	40	74.9	227	4.2E-59
RHG_cluster_346	Other	ABF88003	BGC0000871	VEPE/AEPE/TG-1	28	104.2	205	1.4E-52
RHG_cluster_349	Other	ABF88003	BGC0000871	VEPE/AEPE/TG-1	48	102.6	528	8.9E-150
RHG_cluster_361	Other	ABF88003	BGC0000871	VEPE/AEPE/TG-1	35	102.0	219	6.2E-57
RHG_cluster_368	Other	ABF88003	BGC0000871	VEPE/AEPE/TG-1	30	90.5	144	2.3E-34
RHG_cluster_371	Other	ABF88003	BGC0000871	VEPE/AEPE/TG-1	31	95.2	100	3.5E-21
RHG_cluster_166	Phosphonate	ADB43073	BGC0000926	Rhizocticin	50	51.9	280	4.0E-75
RHG_cluster_230	Phosphonate	ADB43073	BGC0000926	Rhizocticin	44	54.6	249	7.5E-66
RHG_cluster_290	Phosphonate	ADB43073	BGC0000926	Rhizocticin	48	50.6	255	1.4E-67
RHG_cluster_291	Phosphonate	CAJ14044	BGC0000406	Phosphinothricin	48	55.1	270	3.2E-72
RHG_cluster_370	Phosphonate	ADB43073	BGC0000926	Rhizocticin	51	74.9	253	4.3E-67
RHG_cluster_002	Terpene	CAB39697	BGC0000663	Hopene	43	97.1	495	1.3E-139
RHG_cluster_003	Terpene	NP_199612	BGC0000670	Thalianol	32	58.9	329	1.2E-89
RHG_cluster_006	Terpene	-	-	-	-	-	-	-
RHG_cluster_013	Terpene	-	-	-	-	-	-	-
RHG_cluster_015	Terpene	CAB39697	BGC0000663	Hopene	42	90.3	520	2.3E-147

RHG_cluster_026	Terpene	-	-	-	-	-	-	-
RHG_cluster_036	Terpene	BAG16278	BGC0000632	Brasilicardin_A	33	32.9	76	1.6E-13
RHG_cluster_041	Terpene	chr3.CM0292.40.r2.m	BGC0001317	Lupeol	34	56.6	378	3.9E-104
RHG_cluster_042	Terpene	-	-	-	-	-	-	-
RHG_cluster_043	Terpene	NP_199612	BGC0000670	Thalianol	32	59.0	319	1.2E-86
RHG_cluster_047	Terpene	-	-	-	-	-	-	-
RHG_cluster_050	Terpene	NP_199612	BGC0000670	Thalianol	32	59.3	310	7.5E-84
RHG_cluster_052	Terpene	-	-	-	-	-	-	-
RHG_cluster_055	Terpene	AED94049	BGC0001314	Tirucalla	30	58.6	336	1.7E-91
RHG_cluster_058	Terpene	AED94049	BGC0001314	Tirucalla	31	57.7	341	3.1E-93
RHG_cluster_060	Terpene	NP_199612	BGC0000670	Thalianol	31	56.9	125	2.0E-28
RHG_cluster_063	Terpene	-	-	-	-	-	-	-
RHG_cluster_071	Terpene	-	-	-	-	-	-	-
RHG_cluster_101	Terpene	-	-	-	-	-	-	-
RHG_cluster_102	Terpene	chr3.CM0292.40.r2.m	BGC0001317	Lupeol	35	59.3	395	1.8E-109
RHG_cluster_104	Terpene	CAB39692	BGC0000663	Hopene	39	36.4	168	2.9E-41
RHG_cluster_105	Terpene	-	-	-	-	-	-	-
RHG_cluster_106	Terpene	-	-	-	-	-	-	-
RHG_cluster_107	Terpene	-	-	-	-	-	-	-
RHG_cluster_113	Terpene	AGS67377	BGC0001227	Isorenieratene	35	29.7	50	5.6E-06
RHG_cluster_118	Terpene	-	-	-	-	-	-	-
RHG_cluster_123	Terpene	NP_199612	BGC0000670	Thalianol	32	60.4	321	5.5E-87
RHG_cluster_137	Terpene	CAB39697	BGC0000663	Hopene	59	96.6	748	4.7E-216
RHG_cluster_146	Terpene	chr3.CM0292.40.r2.m	BGC0001317	Lupeol	34	98.5	378	1.7E-104
RHG_cluster_152	Terpene	CAB39697	BGC0000663	Hopene	40	93.9	452	9.5E-127
RHG_cluster_157	Terpene	AED94049	BGC0001314	Tirucalla	30	59.1	326	1.0E-88
RHG_cluster_158	Terpene	-	-	-	-	-	-	-
RHG_cluster_169	Terpene	NP_199612	BGC0000670	Thalianol	31	58.5	306	1.1E-82
RHG_cluster_170	Terpene	ABP55685	BGC0001087	Sioxanthin	44	99.4	244	1.6E-64
RHG_cluster_180	Terpene	-	-	-	-	-	-	-
RHG_cluster_199	Terpene	-	-	-	-	-	-	-
RHG_cluster_200	Terpene	-	-	-	-	-	-	-

RHG_cluster_201	Terpene	AMY15074	BGC0001339	Squalestatin_S1	26	83.1	90	5.0E-18
RHG_cluster_207	Terpene	-	-	-	-	-	-	-
RHG_cluster_219	Terpene	-	-	-	-	-	-	-
RHG_cluster_224	Terpene	chr3.CM0292.40.r2.m	BGC0001317	Lupeol	34	58.2	362	1.7E-99
RHG_cluster_234	Terpene	CAB39697	BGC0000663	Hopene	39	93.9	447	2.3E-125
RHG_cluster_243	Terpene	ABP55685	BGC0001087	Sioxanthin	42	99.7	237	3.3E-62
RHG_cluster_247	Terpene	chr3.CM0292.40.r2.m	BGC0001317	Lupeol	34	63.0	378	2.7E-104
RHG_cluster_252	Terpene	AEE83589	BGC0001313	Arabidiol/Baruol.	24	37.9	56	1.5E-07
RHG_cluster_256	Terpene	CAB39697	BGC0000663	Hopene	55	98.9	727	1.2E-209
RHG_cluster_276	Terpene	CAB39697	BGC0000663	Hopene	44	92.8	493	3.6E-139
RHG_cluster_280	Terpene	CAB39697	BGC0000663	Hopene	49	97.9	623	3.8E-178
RHG_cluster_284	Terpene	CAB39697	BGC0000663	Hopene	40	93.8	487	2.7E-137
RHG_cluster_299	Terpene	CAB39697	BGC0000663	Hopene	46	99.0	475	1.1E-133
RHG_cluster_304	Terpene	CAB39697	BGC0000663	Hopene	42	98.9	487	3.4E-137
RHG_cluster_309	Terpene	CAB39697	BGC0000663	Hopene	48	96.9	614	1.1E-175
RHG_cluster_311	Terpene	NP_199612	BGC0000670	Thalianol	32	99.8	272	1.1E-72
RHG_cluster_314	Terpene	CAB39697	BGC0000663	Hopene	51	97.5	408	9.3E-114
RHG_cluster_320	Terpene	-	-	-	-	-	-	-
RHG_cluster_323	Terpene	-	-	-	-	-	-	-
RHG_cluster_327	Terpene	CAB39697	BGC0000663	Hopene	42	99.3	330	3.5E-90
RHG_cluster_330	Terpene	CAB39697	BGC0000663	Hopene	51	96.3	440	2.0E-123
RHG_cluster_331	Terpene	CAB39697	BGC0000663	Hopene	60	99.5	740	1.6E-213
RHG_cluster_337	Terpene	-	-	-	-	-	-	-
RHG_cluster_350	Terpene	-	-	-	-	-	-	-
RHG_cluster_353	Terpene	NP_199612	BGC0000670	Thalianol	32	86.6	203	5.4E-52
RHG_cluster_363	Terpene	-	-	-	-	-	-	-
RHG_cluster_364	Terpene	CAB39697	BGC0000663	Hopene	44	96.4	322	8.6E-88
RHG_cluster_366	Terpene	-	-	-	-	-	-	-
RHG_cluster_369	Terpene	CAA79957	BGC0000648	Carotenoid	34	91.3	156	5.5E-38
RHG_cluster_014	TransAT-PKS	AAF62883	BGC0000991	Epothilone	39	91.6	847	2.4E-245
RHG_cluster_161	TransAT-PKS	AAF26921	BGC0000988	Epothilone	39	91.1	830	3.9E-240
RHG_cluster_008	Type I PKS	AIW82279	BGC0001125	Puwainaphycins	37	75.4	1442	0.0E+00

RHG_cluster_011	Type I PKS	AGC45622	BGC0001394	Phenalamide	36	44.7	513	1.2E-144
RHG_cluster_016	Type I PKS	BAG17643	BGC0001043	SGR_PTMs	35	45.9	515	2.4E-145
RHG_cluster_017	Type I PKS	AHB82052	BGC0001019	Microsclerodermins	33	100.2	200	3.5E-51
RHG_cluster_019	Type I PKS	AIW82279	BGC0001125	Puwainaphycins	39	35.7	1516	0.0E+00
RHG_cluster_027	Type I PKS	AIW82279	BGC0001125	Puwainaphycins	38	77.9	1467	0.0E+00
RHG_cluster_028	Type I PKS	BAG17643	BGC0001043	SGR_PTMs	35	47.0	522	3.4E-147
RHG_cluster_034	Type I PKS	AGC45622	BGC0001394	Phenalamide	31	49.5	481	6.6E-135
RHG_cluster_037	Type I PKS	AIW82279	BGC0001125	Puwainaphycins	37	75.6	1485	0.0E+00
RHG_cluster_040	Type I PKS	AAS98781	BGC0001001	Jamaicamide	35	82.8	1423	0.0E+00
RHG_cluster_053	Type I PKS	AHA38199	BGC0000069	Gephyronic_acid	36	98.8	1285	0.0E+00
RHG_cluster_054	Type I PKS	AIW82279	BGC0001125	Puwainaphycins	37	81.6	1499	0.0E+00
RHG_cluster_056	Type I PKS	AIW82279	BGC0001125	Puwainaphycins	38	76.1	1505	0.0E+00
RHG_cluster_057	Type I PKS	AIW82279	BGC0001125	Puwainaphycins	37	76.7	1513	0.0E+00
RHG_cluster_062	Type I PKS	ACB46195	BGC0000989	Epothilone	40	76.3	906	5.5E-263
RHG_cluster_067	Type I PKS	BAG17643	BGC0001043	SGR_PTMs	35	49.1	542	3.2E-153
RHG_cluster_069	Type I PKS	AIW82279	BGC0001125	Puwainaphycins	37	75.9	1494	0.0E+00
RHG_cluster_072	Type I PKS	BAG17643	BGC0001043	SGR_PTMs	34	47.2	511	4.6E-144
RHG_cluster_087	Type I PKS	AIW82279	BGC0001125	Puwainaphycins	37	81.0	1453	0.0E+00
RHG_cluster_090	Type I PKS	AIW82279	BGC0001125	Puwainaphycins	37	76.5	1517	0.0E+00
RHG_cluster_091	Type I PKS	BAG17643	BGC0001043	SGR_PTMs	34	49.4	514	5.4E-145
RHG_cluster_094	Type I PKS	AGC45621	BGC0001394	Phenalamide	42	78.0	850	3.5E-246
RHG_cluster_096	Type I PKS	BAG17643	BGC0001043	SGR_PTMs	33	49.7	525	3.1E-148
RHG_cluster_100	Type I PKS	AIW82279	BGC0001125	Puwainaphycins	37	76.6	1506	0.0E+00
RHG_cluster_109	Type I PKS	BAG17643	BGC0001043	SGR_PTMs	33	67.9	544	6.5E-154
RHG_cluster_119	Type I PKS	AIW82279	BGC0001125	Puwainaphycins	39	26.6	1460	0.0E+00
RHG_cluster_124	Type I PKS	AIW82279	BGC0001125	Puwainaphycins	37	81.8	1483	0.0E+00
RHG_cluster_128	Type I PKS	BAG17643	BGC0001043	SGR_PTMs	34	48.5	526	1.8E-148
RHG_cluster_135	Type I PKS	BAG17643	BGC0001043	SGR_PTMs	35	47.3	541	5.4E-153
RHG_cluster_136	Type I PKS	CAQ43075	BGC0000970	Chondrochloren	32	49.5	514	7.0E-145
RHG_cluster_140	Type I PKS	AAK73514	BGC0000015	Amphotericin	36	51.8	1819	0.0E+00
RHG_cluster_145	Type I PKS	AAF26921	BGC0000988	Epothilone	36	74.8	925	1.5E-268
RHG_cluster_147	Type I PKS	AIW82279	BGC0001125	Puwainaphycins	37	75.2	1501	0.0E+00

RHG_cluster_149	Type I PKS	AIW82279	BGC0001125	Puwainaphycins	37	75.9	1139	0.0E+00
RHG_cluster_154	Type I PKS	AIW82279	BGC0001125	Puwainaphycins	36	76.0	1485	0.0E+00
RHG_cluster_159	Type I PKS	AIW82279	BGC0001125	Puwainaphycins	38	56.6	1531	0.0E+00
RHG_cluster_160	Type I PKS	AIW82279	BGC0001125	Puwainaphycins	37	97.1	1484	0.0E+00
RHG_cluster_162	Type I PKS	AIW82279	BGC0001125	Puwainaphycins	37	75.4	1512	0.0E+00
RHG_cluster_163	Type I PKS	CAQ18828	BGC0000954	Ajudazol	42	89.5	830	3.5E-240
RHG_cluster_164	Type I PKS	AIW82279	BGC0001125	Puwainaphycins	38	76.0	1427	0.0E+00
RHG_cluster_165	Type I PKS	AIW82279	BGC0001125	Puwainaphycins	38	77.0	1512	0.0E+00
RHG_cluster_167	Type I PKS	AAT70105	BGC0001165	Curacin	36	102.2	978	7.2E-285
RHG_cluster_168	Type I PKS	AHB82063	BGC0001231	Microsclerodermins	40	103.0	441	1.7E-123
RHG_cluster_174	Type I PKS	AAU04878	BGC0000365	Glycopeptidolipid	40	95.1	827	2.3E-239
RHG_cluster_177	Type I PKS	AIW82279	BGC0001125	Puwainaphycins	38	74.7	1512	0.0E+00
RHG_cluster_179	Type I PKS	AIW82279	BGC0001125	Puwainaphycins	37	75.7	1444	0.0E+00
RHG_cluster_183	Type I PKS	CAQ18832	BGC0000954	Ajudazol	34	39.5	383	1.7E-105
RHG_cluster_191	Type I PKS	CAQ18834	BGC0000954	Ajudazol	46	103.3	1096	0.0E+00
RHG_cluster_192	Type I PKS	AIW82279	BGC0001125	Puwainaphycins	37	100.0	967	2.8E-281
RHG_cluster_193	Type I PKS	AIW82279	BGC0001125	Puwainaphycins	38	65.2	1512	0.0E+00
RHG_cluster_194	Type I PKS	AIW82279	BGC0001125	Puwainaphycins	37	86.9	1460	0.0E+00
RHG_cluster_195	Type I PKS	AIW82279	BGC0001125	Puwainaphycins	37	93.9	1469	0.0E+00
RHG_cluster_197	Type I PKS	AAF62883	BGC0000991	Epothilone	38	83.7	924	1.9E-268
RHG_cluster_198	Type I PKS	AIW82279	BGC0001125	Puwainaphycins	36	76.8	1101	0.0E+00
RHG_cluster_202	Type I PKS	BAG17643	BGC0001043	SGR_PTMs	34	51.8	540	1.2E-152
RHG_cluster_203	Type I PKS	AIW82279	BGC0001125	Puwainaphycins	37	63.1	1323	0.0E+00
RHG_cluster_204	Type I PKS	AIW82279	BGC0001125	Puwainaphycins	37	101.0	1477	0.0E+00
RHG_cluster_206	Type I PKS	BAG17643	BGC0001043	SGR_PTMs	35	68.8	533	1.0E-150
RHG_cluster_208	Type I PKS	CAQ18829	BGC0000954	Ajudazol	44	91.2	747	2.0E-215
RHG_cluster_209	Type I PKS	BAG17643	BGC0001043	SGR_PTMs	35	48.8	555	2.8E-157
RHG_cluster_213	Type I PKS	ABL86391	BGC0000999	Heat-stable_antifungal_factor	33	51.3	534	5.1E-151
RHG_cluster_215	Type I PKS	CAQ18828	BGC0000954	Ajudazol	48	101.0	587	2.0E-167
RHG_cluster_217	Type I PKS	AIW82279	BGC0001125	Puwainaphycins	36	100.9	961	9.7E-280
RHG_cluster_221	Type I PKS	AAU04878	BGC0000365	Glycopeptidolipid	44	85.7	356	6.6E-98
RHG_cluster_226	Type I PKS	AAF62883	BGC0000991	Epothilone	40	95.3	819	5.0E-237

RHG_cluster_229	Type I PKS	BAK64637	BGC0000135	Reveromycin	38	96.0	875	9.3E-254
RHG_cluster_233	Type I PKS	AAU04878	BGC0000365	Glycopeptidolipid	38	93.5	1352	0.0E+00
RHG_cluster_237	Type I PKS	AAU04878	BGC0000365	Glycopeptidolipid	42	87.1	484	2.8E-136
RHG_cluster_238	Type I PKS	CAQ18828	BGC0000954	Ajudazol	47	97.6	568	1.3E-161
RHG_cluster_240	Type I PKS	AIW82279	BGC0001125	Puwainaphycins	36	82.3	1162	0.0E+00
RHG_cluster_242	Type I PKS	CAQ18828	BGC0000954	Ajudazol	47	97.3	564	1.8E-160
RHG_cluster_246	Type I PKS	AIW82279	BGC0001125	Puwainaphycins	36	77.7	964	2.6E-280
RHG_cluster_248	Type I PKS	AEU11005	BGC0001029	Nostophycin	37	88.8	765	8.2E-221
RHG_cluster_249	Type I PKS	AAU04878	BGC0000365	Glycopeptidolipid	46	99.8	669	6.2E-192
RHG_cluster_255	Type I PKS	CAQ18834	BGC0000954	Ajudazol	43	79.5	344	2.6E-94
RHG_cluster_258	Type I PKS	AIW82279	BGC0001125	Puwainaphycins	37	102.4	1105	0.0E+00
RHG_cluster_261	Type I PKS	AAU04878	BGC0000365	Glycopeptidolipid	40	85.2	530	5.1E-150
RHG_cluster_262	Type I PKS	CAQ18829	BGC0000954	Ajudazol	43	90.5	689	6.1E-198
RHG_cluster_266	Type I PKS	AAT70105	BGC0001165	Curacin	36	99.8	950	3.7E-276
RHG_cluster_268	Type I PKS	AAF62883	BGC0000991	Epothilone	40	86.3	933	4.8E-271
RHG_cluster_269	Type I PKS	CBD77732	BGC0000974	Crocacin	36	102.0	475	1.8E-133
RHG_cluster_272	Type I PKS	CBD77732	BGC0000974	Crocacin	37	99.1	376	6.3E-104
RHG_cluster_279	Type I PKS	AHB82063	BGC0001231	Microsclerodermins	42	98.3	452	7.7E-127
RHG_cluster_282	Type I PKS	CAQ18834	BGC0000954	Ajudazol	43	76.7	282	1.3E-75
RHG_cluster_283	Type I PKS	BAG17643	BGC0001043	SGR_PTMs	35	60.0	423	1.1E-117
RHG_cluster_285	Type I PKS	CAQ18828	BGC0000954	Ajudazol	42	88.4	808	1.1E-233
RHG_cluster_288	Type I PKS	CAQ18828	BGC0000954	Ajudazol	42	98.4	799	7.8E-231
RHG_cluster_289	Type I PKS	AAF62883	BGC0000991	Epothilone	41	88.5	737	3.9E-212
RHG_cluster_294	Type I PKS	AGC45620	BGC0001394	Phenalamide	47	80.6	520	3.3E-147
RHG_cluster_295	Type I PKS	CAQ18828	BGC0000954	Ajudazol	40	95.5	755	1.2E-217
RHG_cluster_296	Type I PKS	CAQ18829	BGC0000954	Ajudazol	45	86.5	547	3.6E-155
RHG_cluster_298	Type I PKS	AAU04878	BGC0000365	Glycopeptidolipid	43	99.6	731	1.3E-210
RHG_cluster_302	Type I PKS	BAG17643	BGC0001043	SGR_PTMs	38	102.2	488	2.0E-137
RHG_cluster_306	Type I PKS	AAU04878	BGC0000365	Glycopeptidolipid	46	98.9	825	6.6E-239
RHG_cluster_308	Type I PKS	ABX60162	BGC0000978	Cylindrospermopsin	47	85.0	423	5.4E-118
RHG_cluster_316	Type I PKS	CAQ18829	BGC0000954	Ajudazol	44	83.8	508	1.0E-143
RHG_cluster_318	Type I PKS	CAQ18828	BGC0000954	Ajudazol	45	75.8	373	7.1E-103

RHG_cluster_322	Type I PKS	CAQ18828	BGC0000954	Ajudazol	46	96.2	679	6.4E-195
RHG_cluster_326	Type I PKS	AGC45620	BGC0001394	Phenalamide	45	85.9	559	7.1E-159
RHG_cluster_335	Type I PKS	CAQ18829	BGC0000954	Ajudazol	47	98.0	542	1.0E-153
RHG_cluster_336	Type I PKS	AAU04878	BGC0000365	Glycopeptidolipid	45	85.2	449	8.7E-126
RHG_cluster_338	Type I PKS	AAU04878	BGC0000365	Glycopeptidolipid	45	101.0	534	1.5E-151
RHG_cluster_340	Type I PKS	CBD77732	BGC0000974	Crocacin	40	96.3	429	6.6E-120
RHG_cluster_341	Type I PKS	CBD77732	BGC0000974	Crocacin	38	101.0	404	3.0E-112
RHG_cluster_342	Type I PKS	AAU04878	BGC0000365	Glycopeptidolipid	49	96.9	525	8.4E-149
RHG_cluster_345	Type I PKS	AAU04878	BGC0000365	Glycopeptidolipid	48	82.7	423	5.8E-118
RHG_cluster_351	Type I PKS	CAQ18834	BGC0000954	Ajudazol	45	101.1	398	1.7E-110
RHG_cluster_354	Type I PKS	AIT55264	BGC0000072	Gulmirecin	41	99.8	334	1.7E-91
RHG_cluster_356	Type I PKS	AAU04878	BGC0000365	Glycopeptidolipid	48	101.9	398	1.5E-110
RHG_cluster_357	Type I PKS	AAU04878	BGC0000365	Glycopeptidolipid	48	102.1	382	6.6E-106
RHG_cluster_362	Type I PKS	BAG17643	BGC0001043	SGR_PTMs	39	103.7	232	1.2E-60
RHG_cluster_367	Type I PKS	CAQ18834	BGC0000954	Ajudazol	39	102.7	217	2.8E-56
RHG_cluster_080	Type II PKS	AAC18108	BGC0000225	Frenolicin	27	107.1	97	4.5E-20
RHG_cluster_076	Type III PKS	BAG17301	BGC0000282	Alkylresorcinol	40	99.7	226	5.7E-59
RHG_cluster_117	Type III PKS	BAG17301	BGC0000282	Alkylresorcinol	40	97.5	222	1.1E-57
RHG_cluster_129	Type III PKS	BAG17301	BGC0000282	Alkylresorcinol	37	99.7	211	1.9E-54
RHG_cluster_267	Type III PKS	BAG17301	BGC0000282	Alkylresorcinol	40	97.5	224	1.7E-58
RHG_cluster_307	Type III PKS	BAG17301	BGC0000282	Alkylresorcinol	43	100.0	249	6.3E-66
RHG_cluster_358	Type III PKS	BAG17301	BGC0000282	Alkylresorcinol	40	97.5	235	1.3E-61

Table S16. BLAST search of the BGCs identified from the metagenome of *Speciospongia* sp. against the MiBIG database

BGC ID	Predicted BGC type	MiBIG Protein	MiBIG Cluster	MiBIG Product	Identity (%)	Coverage (%)	BLAST Score	E-value
SPV_cluster_094	Arylpolyene	AAO39098	BGC0000956	Andrimid	34	63.7	314	5.0E-85
SPV_cluster_112	Arylpolyene	ADI58650	BGC0000187	Asukamycin	40	91.0	214	2.8E-55
SPV_cluster_123	Arylpolyene	ANY57968	BGC0001369	Metatricycloene	34	91.9	321	3.1E-87
SPV_cluster_136	Arylpolyene	AAW85352	BGC0000837	APE_Vf	46	82.9	283	3.5E-76
SPV_cluster_137	Arylpolyene	AAW85352	BGC0000837	APE_Vf	38	97.8	238	1.7E-62
SPV_cluster_138	Arylpolyene	AAW85355	BGC0000837	APE_Vf	56	99.3	465	8.5E-131
SPV_cluster_312	Arylpolyene	AHA12083	BGC0001172	Chlorizidine_A	35	99.5	176	6.4E-44
SPV_cluster_345	Arylpolyene	ABQ04139	BGC0000838	Flexirubin	28	68.5	67	5.0E-11
SPV_cluster_376	Arylpolyene	CUI25675	BGC0001353	Thiolactomycin	38	88.2	191	2.3E-48
SPV_cluster_021	Bacteriocin	-	-	-	-	-	-	-
SPV_cluster_064	Bacteriocin	BAP82649	BGC0001148	Pheganomycin	38	98.8	432	9.7E-121
SPV_cluster_135	Bacteriocin	ZP_00672901	BGC0000481	Trichamide	39	102.8	514	1.7E-145
SPV_cluster_139	Bacteriocin	BAE46919	BGC0000565	Goadsporin	41	97.9	534	2.8E-151
SPV_cluster_176	Bacteriocin	-	-	-	-	-	-	-
SPV_cluster_198	Bacteriocin	-	-	-	-	-	-	-
SPV_cluster_199	Bacteriocin	CAO82084	BGC0000473	Microcyclamide	42	100.8	537	3.3E-152
SPV_cluster_297	Bacteriocin	ACA04490	BGC0000477	Patellin_2/patellin_3	42	103.8	555	8.9E-158
SPV_cluster_346	Bacteriocin	ACA04483	BGC0000480	Tenuecyclamide_A/C	39	100.5	388	2.0E-107
SPV_cluster_350	Bacteriocin	CAO82084	BGC0000473	Microcyclamide	41	102.0	516	5.9E-146
SPV_cluster_355	Bacteriocin	WP_006126653	BGC0000554	SRO15-3108	36	71.5	411	2.1E-114
SPV_cluster_398	Bacteriocin	CAA09633	BGC0000227	Granaticin	32	91.8	115	1.1E-25
SPV_cluster_424	Bacteriocin	ACA04483	BGC0000480	Tenuecyclamide_A/C	38	102.9	347	3.5E-95
SPV_cluster_431	Bacteriocin	CAO82084	BGC0000473	Microcyclamide	38	101.5	210	3.8E-54
SPV_cluster_002	Cf_fatty_acid	ALV82456	BGC0001382	Svaricin	48	98.3	385	6.7E-107
SPV_cluster_003	Cf_fatty_acid	ALV82456	BGC0001382	Svaricin	48	100.2	367	2.5E-101
SPV_cluster_007	Cf_fatty_acid	ALV82456	BGC0001382	Svaricin	48	97.4	368	1.1E-101
SPV_cluster_009	Cf_fatty_acid	ALV82456	BGC0001382	Svaricin	48	99.3	367	1.9E-101
SPV_cluster_014	Cf_fatty_acid	ALV82456	BGC0001382	Svaricin	50	99.3	366	4.2E-101
SPV_cluster_015	Cf_fatty_acid	ALV82456	BGC0001382	Svaricin	47	98.5	362	7.9E-100

SPV_cluster_016	Cf_fatty_acid	-	-	-	-	-	-	-
SPV_cluster_024	Cf_fatty_acid	ALV82456	BGC0001382	Svaricin	50	98.1	387	1.8E-107
SPV_cluster_025	Cf_fatty_acid	ALV82456	BGC0001382	Svaricin	47	98.3	367	2.5E-101
SPV_cluster_026	Cf_fatty_acid	CAA16179	BGC0001063	Undecylprodigiosin	46	94.8	269	5.9E-72
SPV_cluster_027	Cf_fatty_acid	ALV82456	BGC0001382	Svaricin	50	98.3	399	4.6E-111
SPV_cluster_028	Cf_fatty_acid	CAA16179	BGC0001063	Undecylprodigiosin	43	94.3	241	1.3E-63
SPV_cluster_031	Cf_fatty_acid	CAA16177	BGC0001063	Undecylprodigiosin	49	97.4	365	9.4E-101
SPV_cluster_032	Cf_fatty_acid	ALV82456	BGC0001382	Svaricin	46	98.1	365	7.2E-101
SPV_cluster_033	Cf_fatty_acid	ALV82456	BGC0001382	Svaricin	46	99.0	359	6.7E-99
SPV_cluster_034	Cf_fatty_acid	CAA16179	BGC0001063	Undecylprodigiosin	35	84.4	161	1.4E-39
SPV_cluster_036	Cf_fatty_acid	AHF22848	BGC0000091	Marineosin	40	101.9	267	2.6E-71
SPV_cluster_037	Cf_fatty_acid	ALV82456	BGC0001382	Svaricin	46	98.8	349	5.3E-96
SPV_cluster_038	Cf_fatty_acid	CAA16179	BGC0001063	Undecylprodigiosin	44	94.2	258	1.3E-68
SPV_cluster_039	Cf_fatty_acid	ALV82456	BGC0001382	Svaricin	47	98.3	373	3.4E-103
SPV_cluster_042	Cf_fatty_acid	ALV82456	BGC0001382	Svaricin	47	97.7	360	2.4E-99
SPV_cluster_043	Cf_fatty_acid	ALV82456	BGC0001382	Svaricin	38	101.2	284	2.7E-76
SPV_cluster_045	Cf_fatty_acid	ALV82456	BGC0001382	Svaricin	44	96.2	339	5.6E-93
SPV_cluster_046	Cf_fatty_acid	CAA16179	BGC0001063	Undecylprodigiosin	42	85.9	248	1.6E-65
SPV_cluster_053	Cf_fatty_acid	ALV82456	BGC0001382	Svaricin	43	98.6	312	7.3E-85
SPV_cluster_054	Cf_fatty_acid	ALV82456	BGC0001382	Svaricin	46	100.0	366	4.2E-101
SPV_cluster_060	Cf_fatty_acid	ALV82456	BGC0001382	Svaricin	45	99.3	356	5.6E-98
SPV_cluster_066	Cf_fatty_acid	ALV82456	BGC0001382	Svaricin	45	99.0	344	1.3E-94
SPV_cluster_069	Cf_fatty_acid	ALV82456	BGC0001382	Svaricin	47	98.5	358	1.5E-98
SPV_cluster_073	Cf_fatty_acid	CAA16179	BGC0001063	Undecylprodigiosin	44	88.4	245	7.4E-65
SPV_cluster_074	Cf_fatty_acid	ALV82456	BGC0001382	Svaricin	48	99.1	364	1.2E-100
SPV_cluster_076	Cf_fatty_acid	ALV82456	BGC0001382	Svaricin	48	99.3	382	5.6E-106
SPV_cluster_081	Cf_fatty_acid	AHA12083	BGC0001172	Chlorizidine_A	46	97.1	315	8.5E-86
SPV_cluster_083	Cf_fatty_acid	AHA12083	BGC0001172	Chlorizidine_A	49	99.3	360	2.4E-99
SPV_cluster_085	Cf_fatty_acid	AHA12083	BGC0001172	Chlorizidine_A	46	97.1	310	3.6E-84
SPV_cluster_086	Cf_fatty_acid	AHF22848	BGC0000091	Marineosin	45	103.1	303	4.1E-82
SPV_cluster_093	Cf_fatty_acid	AIL50179	BGC0000213	Colabomycin	27	103.6	89	1.2E-17
SPV_cluster_099	Cf_fatty_acid	ALV82456	BGC0001382	Svaricin	47	99.8	382	7.3E-106

SPV_cluster_101	Cf_fatty_acid	ALV82456	BGC0001382	Svaricin	49	98.6	373	4.6E-103
SPV_cluster_107	Cf_fatty_acid	ALV82456	BGC0001382	Svaricin	46	97.6	372	6.0E-103
SPV_cluster_109	Cf_fatty_acid	CAA16179	BGC0001063	Undecylprodigiosin	46	97.0	269	7.4E-72
SPV_cluster_113	Cf_fatty_acid	AHF22848	BGC0000091	Marineosin	47	103.0	306	4.9E-83
SPV_cluster_119	Cf_fatty_acid	CAA16179	BGC0001063	Undecylprodigiosin	42	61.5	226	6.6E-59
SPV_cluster_121	Cf_fatty_acid	ALV82456	BGC0001382	Svaricin	45	99.3	364	1.6E-100
SPV_cluster_122	Cf_fatty_acid	CAA16179	BGC0001063	Undecylprodigiosin	40	98.2	243	4.3E-64
SPV_cluster_124	Cf_fatty_acid	AJO72736	BGC0001381	Brasilinolide	28	46.6	62	1.4E-09
SPV_cluster_126	Cf_fatty_acid	AHF22848	BGC0000091	Marineosin	36	100.5	199	6.6E-51
SPV_cluster_128	Cf_fatty_acid	ALV82456	BGC0001382	Svaricin	49	98.5	372	7.6E-103
SPV_cluster_129	Cf_fatty_acid	AHF22848	BGC0000091	Marineosin	44	105.6	289	8.0E-78
SPV_cluster_130	Cf_fatty_acid	CAA16179	BGC0001063	Undecylprodigiosin	41	98.5	241	1.3E-63
SPV_cluster_133	Cf_fatty_acid	CAA16179	BGC0001063	Undecylprodigiosin	47	91.9	274	1.9E-73
SPV_cluster_145	Cf_fatty_acid	CBJ83012	BGC0000189	Xenocyloins	50	76.7	458	1.6E-128
SPV_cluster_148	Cf_fatty_acid	ALV82456	BGC0001382	Svaricin	42	100.7	285	1.2E-76
SPV_cluster_152	Cf_fatty_acid	CAA16177	BGC0001063	Undecylprodigiosin	47	98.8	336	3.5E-92
SPV_cluster_155	Cf_fatty_acid	ABQ04139	BGC0000838	Flexirubin	40	100.7	294	2.6E-79
SPV_cluster_159	Cf_fatty_acid	ALV82456	BGC0001382	Svaricin	46	99.8	332	8.8E-91
SPV_cluster_160	Cf_fatty_acid	ALV82456	BGC0001382	Svaricin	50	98.8	387	1.8E-107
SPV_cluster_162	Cf_fatty_acid	AAG04388	BGC0000922	Pseudomonas_quinolone_signal	40	94.6	231	1.4E-60
SPV_cluster_173	Cf_fatty_acid	ALV82456	BGC0001382	Svaricin	46	97.4	370	3.0E-102
SPV_cluster_178	Cf_fatty_acid	ALV82456	BGC0001382	Svaricin	46	100.0	351	1.4E-96
SPV_cluster_179	Cf_fatty_acid	CAA16177	BGC0001063	Undecylprodigiosin	44	103.1	279	6.4E-75
SPV_cluster_180	Cf_fatty_acid	ALV82456	BGC0001382	Svaricin	47	97.8	372	7.6E-103
SPV_cluster_182	Cf_fatty_acid	ALV82456	BGC0001382	Svaricin	49	98.3	362	6.0E-100
SPV_cluster_189	Cf_fatty_acid	CAA16179	BGC0001063	Undecylprodigiosin	43	89.9	242	8.1E-64
SPV_cluster_192	Cf_fatty_acid	ALV82456	BGC0001382	Svaricin	46	97.4	372	6.0E-103
SPV_cluster_195	Cf_fatty_acid	ALV82456	BGC0001382	Svaricin	42	98.3	326	4.8E-89
SPV_cluster_197	Cf_fatty_acid	ALV82456	BGC0001382	Svaricin	48	98.5	371	1.3E-102
SPV_cluster_200	Cf_fatty_acid	CAA16179	BGC0001063	Undecylprodigiosin	45	75.9	254	2.4E-67
SPV_cluster_201	Cf_fatty_acid	ALV82456	BGC0001382	Svaricin	46	96.5	369	6.7E-102
SPV_cluster_205	Cf_fatty_acid	ALV82456	BGC0001382	Svaricin	50	97.4	375	9.3E-104

SPV_cluster_207	Cf_fatty_acid	ALV82456	BGC0001382	Svaricin	48	98.5	373	4.5E-103
SPV_cluster_209	Cf_fatty_acid	ALV82456	BGC0001382	Svaricin	44	99.0	352	8.2E-97
SPV_cluster_215	Cf_fatty_acid	CAA16177	BGC0001063	Undecylprodigiosin	46	98.8	332	6.6E-91
SPV_cluster_216	Cf_fatty_acid	ALV82456	BGC0001382	Svaricin	37	98.1	305	1.2E-82
SPV_cluster_217	Cf_fatty_acid	ALV82456	BGC0001382	Svaricin	46	99.3	355	7.4E-98
SPV_cluster_219	Cf_fatty_acid	AAF81236	BGC0000243	Macrotetrolide	27	103.0	94	3.3E-19
SPV_cluster_222	Cf_fatty_acid	ALV82456	BGC0001382	Svaricin	47	98.1	369	6.5E-102
SPV_cluster_224	Cf_fatty_acid	AHF22848	BGC0000091	Marineosin	47	102.5	309	4.4E-84
SPV_cluster_228	Cf_fatty_acid	ACU59351	BGC0000839	Flexirubin	41	100.7	290	2.9E-78
SPV_cluster_229	Cf_fatty_acid	EDY42532	BGC0000212	Cinerubin_B	34	107.0	160	4.4E-39
SPV_cluster_230	Cf_fatty_acid	ABQ04139	BGC0000838	Flexirubin	40	100.0	269	6.9E-72
SPV_cluster_233	Cf_fatty_acid	AHA12083	BGC0001172	Chlorizidine_A	42	98.3	274	2.2E-73
SPV_cluster_235	Cf_fatty_acid	ALV82456	BGC0001382	Svaricin	47	98.5	377	1.8E-104
SPV_cluster_236	Cf_fatty_acid	ALV82456	BGC0001382	Svaricin	41	102.2	245	1.0E-64
SPV_cluster_239	Cf_fatty_acid	ALV82456	BGC0001382	Svaricin	47	99.5	339	4.2E-93
SPV_cluster_240	Cf_fatty_acid	ALV82456	BGC0001382	Svaricin	40	98.3	301	1.3E-81
SPV_cluster_243	Cf_fatty_acid	AIT38298	BGC0000382	Lipstatin	33	47.3	156	1.4E-37
SPV_cluster_250	Cf_fatty_acid	ACU59351	BGC0000839	Flexirubin	34	100.7	218	1.9E-56
SPV_cluster_251	Cf_fatty_acid	ALV82456	BGC0001382	Svaricin	47	98.8	373	2.6E-103
SPV_cluster_254	Cf_fatty_acid	ALV82456	BGC0001382	Svaricin	46	99.3	355	9.7E-98
SPV_cluster_255	Cf_fatty_acid	ALV82456	BGC0001382	Svaricin	47	98.3	358	1.5E-98
SPV_cluster_256	Cf_fatty_acid	CAA16179	BGC0001063	Undecylprodigiosin	45	93.5	267	1.7E-71
SPV_cluster_258	Cf_fatty_acid	CAA16177	BGC0001063	Undecylprodigiosin	50	99.3	386	3.0E-107
SPV_cluster_259	Cf_fatty_acid	ALV82456	BGC0001382	Svaricin	48	99.1	367	2.5E-101
SPV_cluster_261	Cf_fatty_acid	ACU59351	BGC0000839	Flexirubin	27	44.4	78	2.9E-14
SPV_cluster_262	Cf_fatty_acid	ACA34361	BGC0001152	Fusaricidin	42	99.2	277	2.2E-74
SPV_cluster_263	Cf_fatty_acid	ALV82456	BGC0001382	Svaricin	49	98.8	396	3.8E-110
SPV_cluster_271	Cf_fatty_acid	ALV82456	BGC0001382	Svaricin	48	97.8	381	9.7E-106
SPV_cluster_274	Cf_fatty_acid	ALV82456	BGC0001382	Svaricin	50	98.3	371	1.7E-102
SPV_cluster_275	Cf_fatty_acid	ADG86315	BGC0000190	A-74528	40	96.9	298	1.4E-80
SPV_cluster_276	Cf_fatty_acid	ALV82456	BGC0001382	Svaricin	42	97.4	317	2.3E-86
SPV_cluster_279	Cf_fatty_acid	ACA34361	BGC0001152	Fusaricidin	49	96.2	350	2.7E-96

SPV_cluster_280	Cf_fatty_acid	ALV82456	BGC0001382	Svaricin	45	98.8	363	3.5E-100
SPV_cluster_284	Cf_fatty_acid	ALV82456	BGC0001382	Svaricin	47	99.3	369	3.8E-102
SPV_cluster_285	Cf_fatty_acid	CAA16179	BGC0001063	Undecylprodigiosin	43	94.3	240	2.9E-63
SPV_cluster_289	Cf_fatty_acid	ALV82456	BGC0001382	Svaricin	48	98.8	364	2.1E-100
SPV_cluster_290	Cf_fatty_acid	CAA16177	BGC0001063	Undecylprodigiosin	44	102.8	297	2.3E-80
SPV_cluster_293	Cf_fatty_acid	ABQ04139	BGC0000838	Flexirubin	40	100.5	294	1.5E-79
SPV_cluster_296	Cf_fatty_acid	CAA16179	BGC0001063	Undecylprodigiosin	40	87.1	225	7.9E-59
SPV_cluster_301	Cf_fatty_acid	ALV82456	BGC0001382	Svaricin	44	95.8	339	5.6E-93
SPV_cluster_307	Cf_fatty_acid	CAA16177	BGC0001063	Undecylprodigiosin	46	98.8	329	4.3E-90
SPV_cluster_308	Cf_fatty_acid	AHA12083	BGC0001172	Chlorizidine_A	39	97.6	280	3.1E-75
SPV_cluster_313	Cf_fatty_acid	AEK75497	BGC0000001	Abyssomicin	26	85.7	61	3.1E-09
SPV_cluster_316	Cf_fatty_acid	ALV82456	BGC0001382	Svaricin	49	98.5	371	1.0E-102
SPV_cluster_317	Cf_fatty_acid	CAA16177	BGC0001063	Undecylprodigiosin	52	98.8	348	1.2E-95
SPV_cluster_318	Cf_fatty_acid	ABQ04139	BGC0000838	Flexirubin	42	100.6	265	6.5E-71
SPV_cluster_320	Cf_fatty_acid	AHF22848	BGC0000091	Marineosin	46	103.8	305	8.4E-83
SPV_cluster_322	Cf_fatty_acid	CAB38890	BGC0000315	Calcium-dependent_antibiotic	44	87.8	240	2.4E-63
SPV_cluster_324	Cf_fatty_acid	ALV82456	BGC0001382	Svaricin	44	99.0	353	3.7E-97
SPV_cluster_329	Cf_fatty_acid	ALV82456	BGC0001382	Svaricin	46	98.5	364	1.6E-100
SPV_cluster_330	Cf_fatty_acid	ALV82456	BGC0001382	Svaricin	46	99.4	294	1.3E-79
SPV_cluster_334	Cf_fatty_acid	ALV82456	BGC0001382	Svaricin	49	99.0	377	1.8E-104
SPV_cluster_337	Cf_fatty_acid	AHA12083	BGC0001172	Chlorizidine_A	47	73.0	224	1.8E-58
SPV_cluster_339	Cf_fatty_acid	ALV82456	BGC0001382	Svaricin	49	98.8	377	1.8E-104
SPV_cluster_340	Cf_fatty_acid	ALV82456	BGC0001382	Svaricin	44	96.0	344	1.7E-94
SPV_cluster_341	Cf_fatty_acid	ALV82456	BGC0001382	Svaricin	50	98.3	370	2.3E-102
SPV_cluster_349	Cf_fatty_acid	ALV82456	BGC0001382	Svaricin	50	98.7	339	3.8E-93
SPV_cluster_352	Cf_fatty_acid	CAA16179	BGC0001063	Undecylprodigiosin	42	92.9	242	6.0E-64
SPV_cluster_353	Cf_fatty_acid	ALV82456	BGC0001382	Svaricin	50	96.9	369	5.0E-102
SPV_cluster_354	Cf_fatty_acid	AMEG_HIPS_SALB_00467	BGC0001150	Pamamycin	36	85.4	178	1.4E-44
SPV_cluster_358	Cf_fatty_acid	ALV82456	BGC0001382	Svaricin	43	100.6	261	1.2E-69
SPV_cluster_359	Cf_fatty_acid	CAA16179	BGC0001063	Undecylprodigiosin	46	92.6	254	2.0E-67
SPV_cluster_362	Cf_fatty_acid	ALV82456	BGC0001382	Svaricin	49	97.4	373	4.6E-103

SPV_cluster_363	Cf_fatty_acid	ALV82456	BGC0001382	Svaricin	43	98.6	324	2.4E-88
SPV_cluster_364	Cf_fatty_acid	CAA16177	BGC0001063	Undecylprodigiosin	45	96.4	320	3.5E-87
SPV_cluster_366	Cf_fatty_acid	ABQ04139	BGC0000838	Flexirubin	39	100.2	270	2.4E-72
SPV_cluster_367	Cf_fatty_acid	AEE65468	BGC0000223	Fluostatin	42	99.5	317	2.3E-86
SPV_cluster_375	Cf_fatty_acid	ABQ04139	BGC0000838	Flexirubin	40	100.2	292	9.8E-79
SPV_cluster_379	Cf_fatty_acid	CAA16179	BGC0001063	Undecylprodigiosin	42	90.7	235	7.5E-62
SPV_cluster_389	Cf_fatty_acid	ALV82456	BGC0001382	Svaricin	50	98.8	401	9.0E-112
SPV_cluster_400	Cf_fatty_acid	ALV82456	BGC0001382	Svaricin	48	97.4	370	2.2E-102
SPV_cluster_401	Cf_fatty_acid	ACU59351	BGC0000839	Flexirubin	40	99.0	282	1.0E-75
SPV_cluster_402	Cf_fatty_acid	ALV82456	BGC0001382	Svaricin	47	98.1	351	1.4E-96
SPV_cluster_403	Cf_fatty_acid	ACA34361	BGC0001152	Fusaricidin	48	96.4	333	2.6E-91
SPV_cluster_406	Cf_fatty_acid	ALV82456	BGC0001382	Svaricin	47	98.6	315	7.5E-86
SPV_cluster_408	Cf_fatty_acid	CBJ83012	BGC0000189	Xenocyloins	49	99.8	411	1.2E-114
SPV_cluster_410	Cf_fatty_acid	CAB38890	BGC0000315	Calcium-dependent_antibiotic	42	89.1	229	6.9E-60
SPV_cluster_411	Cf_fatty_acid	CAA16177	BGC0001063	Undecylprodigiosin	38	97.2	186	4.0E-47
SPV_cluster_414	Cf_fatty_acid	CAA16177	BGC0001063	Undecylprodigiosin	47	98.3	310	3.5E-84
SPV_cluster_416	Cf_fatty_acid	ACU59351	BGC0000839	Flexirubin	42	101.5	270	3.1E-72
SPV_cluster_417	Cf_fatty_acid	ALV82456	BGC0001382	Svaricin	45	100.0	359	3.9E-99
SPV_cluster_419	Cf_fatty_acid	ALV82456	BGC0001382	Svaricin	49	99.0	381	9.7E-106
SPV_cluster_423	Cf_fatty_acid	ACA34361	BGC0001152	Fusaricidin	49	91.9	336	5.6E-92
SPV_cluster_430	Cf_fatty_acid	ALV82456	BGC0001382	Svaricin	48	99.5	374	2.0E-103
SPV_cluster_432	Cf_fatty_acid	ALV82456	BGC0001382	Svaricin	48	99.7	303	3.7E-82
SPV_cluster_433	Cf_fatty_acid	ALV82456	BGC0001382	Svaricin	50	94.5	375	9.5E-104
SPV_cluster_436	Cf_fatty_acid	ALV82456	BGC0001382	Svaricin	50	96.7	346	4.3E-95
SPV_cluster_438	Cf_fatty_acid	ABQ04139	BGC0000838	Flexirubin	41	100.6	259	4.8E-69
SPV_cluster_441	Cf_fatty_acid	ALV82456	BGC0001382	Svaricin	44	98.3	340	2.5E-93
SPV_cluster_442	Cf_fatty_acid	ALV82456	BGC0001382	Svaricin	42	96.5	310	2.6E-84
SPV_cluster_443	Cf_fatty_acid	CAA16177	BGC0001063	Undecylprodigiosin	48	99.2	301	1.1E-81
SPV_cluster_444	Cf_fatty_acid	ALV82456	BGC0001382	Svaricin	48	97.3	327	2.6E-89
SPV_cluster_445	Cf_fatty_acid	ALV82456	BGC0001382	Svaricin	48	100.2	363	3.6E-100
SPV_cluster_461	Cf_fatty_acid	ACA34361	BGC0001152	Fusaricidin	51	97.9	329	6.1E-90
SPV_cluster_092	Ladderane	CAL48955	BGC0001343	Aurachin	38	97.4	254	2.4E-67

SPV_cluster_125	Ladderane	CAL48955	BGC0001343	Aurachin	39	98.3	257	2.7E-68
SPV_cluster_044	Lantipeptide	ACR33053	BGC0000495	Actagardine	30	101.6	277	5.0E-74
SPV_cluster_061	Lantipeptide	CAX48971	BGC0000519	Labyrinthopeptin_A1,A3/ A2	29	93.8	278	2.5E-74
SPV_cluster_365	Lantipeptide	CAX48971	BGC0000519	Labyrinthopeptin_A1,A3/ A2	34	94.4	356	7.2E-98
SPV_cluster_012	Other	ABF88003	BGC0000871	VEPE/AEPE/TG-1	33	102.7	639	6.7E-183
SPV_cluster_017	Other	ABF88003	BGC0000871	VEPE/AEPE/TG-1	29	73.7	306	1.1E-82
SPV_cluster_018	Other	ABF88003	BGC0000871	VEPE/AEPE/TG-1	31	104.4	619	7.3E-177
SPV_cluster_022	Other	ABF87031	BGC0000393	Myxoprincomide	42	53.9	364	5.2E-100
SPV_cluster_068	Other	ACR50797	BGC0000163	Tetronasin	37	62.8	337	3.6E-92
SPV_cluster_088	Other	ABF88003	BGC0000871	VEPE/AEPE/TG-1	29	107.4	285	2.6E-76
SPV_cluster_097	Other	BAE93741	BGC0000164	Tetronomycin	37	60.4	329	1.7E-89
SPV_cluster_098	Other	ABF88003	BGC0000871	VEPE/AEPE/TG-1	33	61.9	469	1.3E-131
SPV_cluster_104	Other	ABF88003	BGC0000871	VEPE/AEPE/TG-1	30	104.8	593	7.3E-169
SPV_cluster_117	Other	ABF88003	BGC0000871	VEPE/AEPE/TG-1	28	92.9	223	9.5E-58
SPV_cluster_132	Other	ABF88003	BGC0000871	VEPE/AEPE/TG-1	44	101.9	1125	0.0E+00
SPV_cluster_157	Other	ACR50797	BGC0000163	Tetronasin	39	55.0	367	5.8E-101
SPV_cluster_165	Other	ABF88003	BGC0000871	VEPE/AEPE/TG-1	31	95.4	396	8.2E-110
SPV_cluster_185	Other	ABF87031	BGC0000393	Myxoprincomide	42	57.9	363	6.4E-100
SPV_cluster_193	Other	ACR50797	BGC0000163	Tetronasin	37	58.9	351	3.1E-96
SPV_cluster_238	Other	ABF88003	BGC0000871	VEPE/AEPE/TG-1	32	102.2	603	5.4E-172
SPV_cluster_252	Other	ABF88003	BGC0000871	VEPE/AEPE/TG-1	31	102.3	619	7.4E-177
SPV_cluster_265	Other	ABF88003	BGC0000871	VEPE/AEPE/TG-1	32	102.5	472	9.4E-133
SPV_cluster_267	Other	ABF87031	BGC0000393	Myxoprincomide	41	54.9	354	4.1E-97
SPV_cluster_270	Other	ACR50797	BGC0000163	Tetronasin	36	84.0	347	3.2E-95
SPV_cluster_277	Other	ABF88003	BGC0000871	VEPE/AEPE/TG-1	32	97.6	302	1.8E-81
SPV_cluster_283	Other	CAQ71826	BGC0001189	Taiwachelin	41	74.5	252	1.1E-66
SPV_cluster_294	Other	BAK64635	BGC0000135	Reveromycin	40	60.1	357	5.9E-98
SPV_cluster_295	Other	AEU11005	BGC0001029	Nostophycin	29	91.6	420	7.0E-117
SPV_cluster_342	Other	ADC79613	BGC0000028	Bafilomycin	28	62.6	151	5.7E-36
SPV_cluster_343	Other	ABF88003	BGC0000871	VEPE/AEPE/TG-1	31	100.8	293	7.2E-79
SPV_cluster_344	Other	ABF88003	BGC0000871	VEPE/AEPE/TG-1	28	102.2	215	2.2E-55
SPV_cluster_361	Other	ABF88003	BGC0000871	VEPE/AEPE/TG-1	30	97.0	128	1.6E-29

SPV_cluster_369	Other	AAY93445	BGC0000413	Pyoverdine	40	66.6	123	5.1E-28
SPV_cluster_392	Other	CAQ71826	BGC0001189	Taiwachelin	40	74.5	325	2.0E-88
SPV_cluster_393	Other	ABF88003	BGC0000871	VEPE/AEPE/TG-1	28	99.8	212	2.0E-54
SPV_cluster_407	Other	BAE93741	BGC0000164	Tetronomycin	37	83.0	340	5.2E-93
SPV_cluster_420	Other	ABF88003	BGC0000871	VEPE/AEPE/TG-1	33	105.1	338	1.5E-92
SPV_cluster_437	Other	ABF87031	BGC0000393	Myxoprincomide	40	71.3	232	9.1E-61
SPV_cluster_449	Other	ABF88003	BGC0000871	VEPE/AEPE/TG-1	27	104.0	143	6.3E-34
SPV_cluster_089	Phosphonate	ADB43073	BGC0000926	Rhizocticin	48	50.6	255	1.4E-67
SPV_cluster_158	Phosphonate	ADB43073	BGC0000926	Rhizocticin	51	53.7	290	5.0E-78
SPV_cluster_170	Phosphonate	ADB43073	BGC0000926	Rhizocticin	48	52.7	275	9.8E-74
SPV_cluster_319	Phosphonate	ADB43073	BGC0000926	Rhizocticin	43	54.6	249	7.5E-66
SPV_cluster_335	Phosphonate	CAJ14044	BGC0000406	Phosphinothricin	50	49.6	233	6.6E-61
SPV_cluster_452	Phosphonate	ADB43073	BGC0000926	Rhizocticin	48	43.0	177	2.9E-44
SPV_cluster_004	Terpene	NP_199612	BGC0000670	Thalianol	32	59.3	319	1.6E-86
SPV_cluster_006	Terpene	-	-	-	-	-	-	-
SPV_cluster_010	Terpene	-	-	-	-	-	-	-
SPV_cluster_011	Terpene	NP_199612	BGC0000670	Thalianol	32	58.9	329	1.2E-89
SPV_cluster_013	Terpene	-	-	-	-	-	-	-
SPV_cluster_019	Terpene	CAB39693	BGC0000663	Hopene	40	56.7	135	1.4E-31
SPV_cluster_023	Terpene	CAB39697	BGC0000663	Hopene	32	70.8	153	7.7E-37
SPV_cluster_041	Terpene	CAB39697	BGC0000663	Hopene	30	67.0	137	4.4E-32
SPV_cluster_048	Terpene	-	-	-	-	-	-	-
SPV_cluster_050	Terpene	CAB39697	BGC0000663	Hopene	41	88.4	481	1.5E-135
SPV_cluster_055	Terpene	ABP56869	BGC0001087	Sioxanthin	29	71.5	66	5.7E-11
SPV_cluster_056	Terpene	-	-	-	-	-	-	-
SPV_cluster_058	Terpene	CAB39697	BGC0000663	Hopene	46	99.3	575	9.6E-164
SPV_cluster_059	Terpene	-	-	-	-	-	-	-
SPV_cluster_062	Terpene	-	-	-	-	-	-	-
SPV_cluster_063	Terpene	ABP55685	BGC0001087	Sioxanthin	43	99.7	243	4.6E-64
SPV_cluster_070	Terpene	CAA79957	BGC0000648	Carotenoid	34	90.2	153	4.7E-37
SPV_cluster_072	Terpene	CAB39697	BGC0000663	Hopene	42	90.3	520	3.0E-147
SPV_cluster_075	Terpene	-	-	-	-	-	-	-

SPV_cluster_077	Terpene	-	-	-	-	-	-	-
SPV_cluster_079	Terpene	CAB39697	BGC0000663	Hopene	40	93.9	452	9.5E-127
SPV_cluster_080	Terpene	-	-	-	-	-	-	-
SPV_cluster_087	Terpene	CAB39697	BGC0000663	Hopene	59	96.6	751	7.3E-217
SPV_cluster_095	Terpene	-	-	-	-	-	-	-
SPV_cluster_100	Terpene	NP_199612	BGC0000670	Thalianol	31	57.4	129	1.4E-29
SPV_cluster_103	Terpene	-	-	-	-	-	-	-
SPV_cluster_105	Terpene	chr3.CM0292.40.r2.m	BGC0001317	Lupeol	34	98.5	378	1.7E-104
SPV_cluster_110	Terpene	-	-	-	-	-	-	-
SPV_cluster_116	Terpene	AMY15074	BGC0001339	Squalestatin_S1	26	83.1	90	5.0E-18
SPV_cluster_118	Terpene	NP_199612	BGC0000670	Thalianol	32	60.4	319	2.1E-86
SPV_cluster_120	Terpene	-	-	-	-	-	-	-
SPV_cluster_127	Terpene	-	-	-	-	-	-	-
SPV_cluster_140	Terpene	ACF70484	BGC0001248	Clavaric_acid	34	58.1	370	6.1E-102
SPV_cluster_142	Terpene	-	-	-	-	-	-	-
SPV_cluster_143	Terpene	-	-	-	-	-	-	-
SPV_cluster_144	Terpene	-	-	-	-	-	-	-
SPV_cluster_147	Terpene	-	-	-	-	-	-	-
SPV_cluster_156	Terpene	ABP55685	BGC0001087	Sioxanthin	44	99.4	243	2.7E-64
SPV_cluster_164	Terpene	-	-	-	-	-	-	-
SPV_cluster_175	Terpene	CAB39697	BGC0000663	Hopene	45	98.4	569	7.0E-162
SPV_cluster_177	Terpene	AIG94830	BGC0001086	Astaxanthin_dideoxyglycoside	44	95.9	260	4.0E-69
SPV_cluster_183	Terpene	-	-	-	-	-	-	-
SPV_cluster_184	Terpene	-	-	-	-	-	-	-
SPV_cluster_186	Terpene	-	-	-	-	-	-	-
SPV_cluster_187	Terpene	-	-	-	-	-	-	-
SPV_cluster_188	Terpene	-	-	-	-	-	-	-
SPV_cluster_190	Terpene	NP_199612	BGC0000670	Thalianol	33	62.0	318	3.4E-86
SPV_cluster_196	Terpene	chr3.CM0292.40.r2.m	BGC0001317	Lupeol	35	57.8	376	8.6E-104
SPV_cluster_203	Terpene	ABP55685	BGC0001087	Sioxanthin	44	99.4	247	2.4E-65
SPV_cluster_223	Terpene	-	-	-	-	-	-	-
SPV_cluster_231	Terpene	ACF70484	BGC0001248	Clavaric_acid	32	61.4	366	1.5E-100

SPV_cluster_232	Terpene	chr3.CM0292.40.r2.m	BGC0001317	Lupeol	35	59.4	381	4.4E-105
SPV_cluster_234	Terpene	-	-	-	-	-	-	-
SPV_cluster_242	Terpene	CAB39697	BGC0000663	Hopene	51	98.1	664	1.2E-190
SPV_cluster_244	Terpene	CAB39697	BGC0000663	Hopene	41	96.8	476	6.0E-134
SPV_cluster_247	Terpene	CAB39693	BGC0000663	Hopene	47	89.9	257	2.2E-68
SPV_cluster_248	Terpene	CAB39697	BGC0000663	Hopene	55	98.9	727	1.2E-209
SPV_cluster_249	Terpene	AAB31139	BGC0000647	Carotenoid	37	40.7	159	1.6E-38
SPV_cluster_257	Terpene	-	-	-	-	-	-	-
SPV_cluster_272	Terpene	CAB39697	BGC0000663	Hopene	43	97.1	497	3.3E-140
SPV_cluster_282	Terpene	CAB39697	BGC0000663	Hopene	48	96.9	614	1.1E-175
SPV_cluster_286	Terpene	CAB39697	BGC0000663	Hopene	51	98.4	661	9.8E-190
SPV_cluster_288	Terpene	chr3.CM0292.40.r2.m	BGC0001317	Lupeol	34	61.5	381	4.4E-105
SPV_cluster_298	Terpene	NP_199612	BGC0000670	Thalianol	34	101.8	250	5.2E-66
SPV_cluster_303	Terpene	BAG16278	BGC0000632	Brasilicardin_A	33	32.9	75	2.0E-13
SPV_cluster_304	Terpene	CAB39697	BGC0000663	Hopene	39	94.0	446	5.2E-125
SPV_cluster_321	Terpene	CAB39697	BGC0000663	Hopene	60	98.2	766	2.1E-221
SPV_cluster_325	Terpene	CAB39697	BGC0000663	Hopene	42	97.0	485	7.6E-137
SPV_cluster_327	Terpene	chr3.CM0292.40.r2.m	BGC0001317	Lupeol	34	56.6	368	2.4E-101
SPV_cluster_328	Terpene	chr3.CM0292.40.r2.m	BGC0001317	Lupeol	34	92.2	233	3.5E-61
SPV_cluster_331	Terpene	chr3.CM0292.40.r2.m	BGC0001317	Lupeol	35	100.5	345	1.4E-94
SPV_cluster_332	Terpene	-	-	-	-	-	-	-
SPV_cluster_336	Terpene	CAB39697	BGC0000663	Hopene	47	100.9	519	4.1E-147
SPV_cluster_351	Terpene	ABP55685	BGC0001087	Sioxanthin	44	99.4	247	2.4E-65
SPV_cluster_370	Terpene	-	-	-	-	-	-	-
SPV_cluster_372	Terpene	CAB39697	BGC0000663	Hopene	40	94.1	406	6.4E-113
SPV_cluster_374	Terpene	AED94049	BGC0001314	Tirucalla	32	90.4	336	6.3E-92
SPV_cluster_381	Terpene	-	-	-	-	-	-	-
SPV_cluster_383	Terpene	-	-	-	-	-	-	-
SPV_cluster_385	Terpene	chr3.CM0292.40.r2.m	BGC0001317	Lupeol	34	70.0	360	6.9E-99
SPV_cluster_387	Terpene	ACF70484	BGC0001248	Clavaric_acid	35	92.2	379	6.3E-105
SPV_cluster_388	Terpene	ACM07426	BGC0000645	Carotenoid	35	41.7	132	1.6E-30
SPV_cluster_390	Terpene	NP_199612	BGC0000670	Thalianol	32	102.4	276	7.3E-74

SPV_cluster_395	Terpene	-	-	-	-	-	-	-
SPV_cluster_397	Terpene	CAB39697	BGC0000663	Hopene	40	92.8	408	1.3E-113
SPV_cluster_399	Terpene	chr3.CM0292.40.r2.m	BGC0001317	Lupeol	34	101.7	236	4.9E-62
SPV_cluster_409	Terpene	CAB39697	BGC0000663	Hopene	43	98.1	405	1.1E-112
SPV_cluster_415	Terpene	CAB39697	BGC0000663	Hopene	40	97.7	371	1.6E-102
SPV_cluster_427	Terpene	CAB39697	BGC0000663	Hopene	51	97.7	508	6.6E-144
SPV_cluster_429	Terpene	CAB39697	BGC0000663	Hopene	40	99.4	386	3.9E-107
SPV_cluster_434	Terpene	CAB39697	BGC0000663	Hopene	39	97.8	319	6.6E-87
SPV_cluster_435	Terpene	CAB39697	BGC0000663	Hopene	53	99.2	512	4.3E-145
SPV_cluster_439	Terpene	CAB39697	BGC0000663	Hopene	55	100.7	488	7.8E-138
SPV_cluster_453	Terpene	-	-	-	-	-	-	-
SPV_cluster_458	Terpene	CAB39697	BGC0000663	Hopene	39	90.9	250	2.3E-66
SPV_cluster_462	Terpene	CAB39697	BGC0000663	Hopene	49	100.5	387	2.1E-107
SPV_cluster_463	Terpene	-	-	-	-	-	-	-
SPV_cluster_051	TransAT-Type I PKS	AIW82279	BGC0001125	Puwainaphycins	37	80.9	1490	0.0E+00
SPV_cluster_115	TransAT-Type I PKS	AIW82279	BGC0001125	Puwainaphycins	37	80.5	1440	0.0E+00
SPV_cluster_001	Type I PKS	AAF26921	BGC0000988	Epothilone	38	101.7	1287	0.0E+00
SPV_cluster_005	Type I PKS	AIW82279	BGC0001125	Puwainaphycins	36	77.6	1461	0.0E+00
SPV_cluster_008	Type I PKS	AAS98781	BGC0001001	Jamaicamide	35	73.8	1420	0.0E+00
SPV_cluster_020	Type I PKS	AIW82279	BGC0001125	Puwainaphycins	37	76.0	1522	0.0E+00
SPV_cluster_029	Type I PKS	AIW82279	BGC0001125	Puwainaphycins	39	35.7	1516	0.0E+00
SPV_cluster_030	Type I PKS	AAX98191	BGC0000052	ECO-02301	35	20.0	1774	0.0E+00
SPV_cluster_035	Type I PKS	AIW82279	BGC0001125	Puwainaphycins	37	82.0	1512	0.0E+00
SPV_cluster_040	Type I PKS	AIW82279	BGC0001125	Puwainaphycins	37	76.8	1543	0.0E+00
SPV_cluster_047	Type I PKS	AIW82279	BGC0001125	Puwainaphycins	38	76.8	1502	0.0E+00
SPV_cluster_049	Type I PKS	BAG17643	BGC0001043	SGR_PTMs	34	49.2	540	7.1E-153
SPV_cluster_052	Type I PKS	AIW82279	BGC0001125	Puwainaphycins	38	75.4	1495	0.0E+00
SPV_cluster_057	Type I PKS	AIW82279	BGC0001125	Puwainaphycins	36	76.2	1472	0.0E+00
SPV_cluster_065	Type I PKS	AGC45622	BGC0001394	Phenalamide	31	49.5	481	6.6E-135
SPV_cluster_067	Type I PKS	BAG17643	BGC0001043	SGR_PTMs	34	48.5	525	3.1E-148
SPV_cluster_071	Type I PKS	AIW82279	BGC0001125	Puwainaphycins	37	77.9	1471	0.0E+00
SPV_cluster_078	Type I PKS	AIW82279	BGC0001125	Puwainaphycins	38	76.7	1488	0.0E+00

SPV_cluster_082	Type I PKS	AAS98781	BGC0001001	Jamaicamide	35	81.4	1400	0.0E+00
SPV_cluster_084	Type I PKS	AIW82279	BGC0001125	Puwainaphycins	37	82.6	1501	0.0E+00
SPV_cluster_091	Type I PKS	BAG17643	BGC0001043	SGR_PTMs	35	88.7	521	2.4E-147
SPV_cluster_096	Type I PKS	AIW82279	BGC0001125	Puwainaphycins	38	75.3	1480	0.0E+00
SPV_cluster_102	Type I PKS	AIW82279	BGC0001125	Puwainaphycins	38	75.7	1525	0.0E+00
SPV_cluster_106	Type I PKS	AAK19883	BGC0000147	Soraphen	35	64.5	1687	0.0E+00
SPV_cluster_108	Type I PKS	CAQ43075	BGC0000970	Chondrochloren	33	50.2	534	6.6E-151
SPV_cluster_111	Type I PKS	CAQ18834	BGC0000954	Ajudazol	40	77.3	231	2.7E-60
SPV_cluster_114	Type I PKS	AIW82279	BGC0001125	Puwainaphycins	37	76.3	1495	0.0E+00
SPV_cluster_134	Type I PKS	AIW82279	BGC0001125	Puwainaphycins	36	75.9	1474	0.0E+00
SPV_cluster_141	Type I PKS	BAG17643	BGC0001043	SGR_PTMs	36	47.4	547	7.5E-155
SPV_cluster_146	Type I PKS	AIW82279	BGC0001125	Puwainaphycins	37	76.5	1506	0.0E+00
SPV_cluster_149	Type I PKS	AIW82279	BGC0001125	Puwainaphycins	36	86.7	1453	0.0E+00
SPV_cluster_150	Type I PKS	AIW82279	BGC0001125	Puwainaphycins	36	74.0	1175	0.0E+00
SPV_cluster_151	Type I PKS	AIW82279	BGC0001125	Puwainaphycins	37	75.2	1495	0.0E+00
SPV_cluster_153	Type I PKS	BAG17643	BGC0001043	SGR_PTMs	35	49.3	539	1.6E-152
SPV_cluster_154	Type I PKS	AIW82279	BGC0001125	Puwainaphycins	37	76.8	1515	0.0E+00
SPV_cluster_161	Type I PKS	BAG17643	BGC0001043	SGR_PTMs	34	49.3	516	1.4E-145
SPV_cluster_163	Type I PKS	BAG17643	BGC0001043	SGR_PTMs	33	53.4	545	2.2E-154
SPV_cluster_166	Type I PKS	AGC45622	BGC0001394	Phenalamide	34	49.3	529	1.6E-149
SPV_cluster_167	Type I PKS	AAF62883	BGC0000991	Epothilone	42	89.9	792	6.1E-229
SPV_cluster_168	Type I PKS	AAS98781	BGC0001001	Jamaicamide	34	81.9	1307	0.0E+00
SPV_cluster_169	Type I PKS	AIW82279	BGC0001125	Puwainaphycins	38	52.7	1529	0.0E+00
SPV_cluster_171	Type I PKS	AIW82279	BGC0001125	Puwainaphycins	38	77.0	1510	0.0E+00
SPV_cluster_172	Type I PKS	AGC45622	BGC0001394	Phenalamide	34	47.8	526	1.4E-148
SPV_cluster_174	Type I PKS	AIW82279	BGC0001125	Puwainaphycins	37	75.3	1468	0.0E+00
SPV_cluster_181	Type I PKS	AIW82279	BGC0001125	Puwainaphycins	36	71.1	1157	0.0E+00
SPV_cluster_191	Type I PKS	AIW82279	BGC0001125	Puwainaphycins	37	75.2	1318	0.0E+00
SPV_cluster_194	Type I PKS	AGC45622	BGC0001394	Phenalamide	33	49.8	499	1.8E-140
SPV_cluster_202	Type I PKS	AAU04878	BGC0000365	Glycopeptidolipid	47	82.1	527	3.7E-149
SPV_cluster_204	Type I PKS	BAG17643	BGC0001043	SGR_PTMs	35	41.2	438	3.5E-122
SPV_cluster_206	Type I PKS	AAF62883	BGC0000991	Epothilone	40	67.8	930	4.0E-270

SPV_cluster_208	Type I PKS	AEE88280	BGC0000976	Curacin	36	101.4	971	1.7E-282
SPV_cluster_210	Type I PKS	AIW82279	BGC0001125	Puwainaphycins	37	66.8	945	1.0E-274
SPV_cluster_211	Type I PKS	AAQ82567	BGC0000034	Candicidin	40	90.7	902	7.0E-262
SPV_cluster_212	Type I PKS	AIW82279	BGC0001125	Puwainaphycins	38	98.0	1501	0.0E+00
SPV_cluster_213	Type I PKS	AIW82279	BGC0001125	Puwainaphycins	38	100.6	1523	0.0E+00
SPV_cluster_214	Type I PKS	AIW82279	BGC0001125	Puwainaphycins	37	76.7	1531	0.0E+00
SPV_cluster_220	Type I PKS	BAG17643	BGC0001043	SGR_PTMs	34	53.2	524	5.3E-148
SPV_cluster_221	Type I PKS	AHA38199	BGC0000069	Gephyronic_acid	36	98.8	1285	0.0E+00
SPV_cluster_225	Type I PKS	AGC45621	BGC0001394	Phenalamide	50	99.3	560	2.9E-159
SPV_cluster_226	Type I PKS	CAQ18828	BGC0000954	Ajudazol	37	77.2	884	2.4E-256
SPV_cluster_227	Type I PKS	AAF26921	BGC0000988	Epothilone	40	86.6	906	5.2E-263
SPV_cluster_237	Type I PKS	AIW82279	BGC0001125	Puwainaphycins	37	65.5	1323	0.0E+00
SPV_cluster_241	Type I PKS	AIW82279	BGC0001125	Puwainaphycins	37	84.1	1451	0.0E+00
SPV_cluster_245	Type I PKS	AIW82279	BGC0001125	Puwainaphycins	37	76.1	1448	0.0E+00
SPV_cluster_246	Type I PKS	AIW82279	BGC0001125	Puwainaphycins	38	78.1	1472	0.0E+00
SPV_cluster_253	Type I PKS	CAQ18828	BGC0000954	Ajudazol	45	100.2	660	2.3E-189
SPV_cluster_260	Type I PKS	AEU11005	BGC0001029	Nostophycin	39	94.1	882	6.7E-256
SPV_cluster_264	Type I PKS	BAG17643	BGC0001043	SGR_PTMs	35	48.8	548	4.5E-155
SPV_cluster_266	Type I PKS	CAQ18829	BGC0000954	Ajudazol	43	87.0	774	2.8E-223
SPV_cluster_268	Type I PKS	AEE88280	BGC0000976	Curacin	36	103.5	963	4.0E-280
SPV_cluster_269	Type I PKS	CAQ18828	BGC0000954	Ajudazol	44	98.3	734	1.5E-211
SPV_cluster_273	Type I PKS	AEU11006	BGC0001029	Nostophycin	46	77.8	521	1.5E-147
SPV_cluster_278	Type I PKS	CAQ18828	BGC0000954	Ajudazol	43	100.3	814	1.3E-235
SPV_cluster_281	Type I PKS	BAG17643	BGC0001043	SGR_PTMs	35	72.3	538	2.3E-152
SPV_cluster_287	Type I PKS	CAQ18828	BGC0000954	Ajudazol	38	72.0	228	2.3E-59
SPV_cluster_291	Type I PKS	CAQ18828	BGC0000954	Ajudazol	47	99.0	673	3.3E-193
SPV_cluster_292	Type I PKS	AHB82063	BGC0001231	Microsclerodermins	39	102.5	386	6.7E-107
SPV_cluster_299	Type I PKS	CAQ18828	BGC0000954	Ajudazol	43	99.4	801	1.5E-231
SPV_cluster_300	Type I PKS	CBD77732	BGC0000974	Crocacin	39	100.9	412	1.1E-114
SPV_cluster_302	Type I PKS	CAQ18829	BGC0000954	Ajudazol	45	80.8	365	1.2E-100
SPV_cluster_305	Type I PKS	CAQ18829	BGC0000954	Ajudazol	43	88.2	742	6.6E-214
SPV_cluster_306	Type I PKS	AAF26921	BGC0000988	Epothilone	39	90.4	885	9.7E-257

SPV_cluster_309	Type I PKS	CAQ18829	BGC0000954	Ajudazol	43	88.4	590	4.4E-168
SPV_cluster_310	Type I PKS	CAQ43075	BGC0000970	Chondrochloren	33	73.5	505	2.2E-142
SPV_cluster_311	Type I PKS	CAQ18828	BGC0000954	Ajudazol	42	89.1	829	7.9E-240
SPV_cluster_314	Type I PKS	AAF26921	BGC0000988	Epothilone	41	90.4	936	4.3E-272
SPV_cluster_315	Type I PKS	CAQ18829	BGC0000954	Ajudazol	38	93.8	646	4.8E-185
SPV_cluster_323	Type I PKS	AIW82279	BGC0001125	Puwainaphycins	35	101.6	915	8.0E-266
SPV_cluster_326	Type I PKS	AAT70105	BGC0001165	Curacin	37	100.5	939	6.3E-273
SPV_cluster_333	Type I PKS	AEE88280	BGC0000976	Curacin	35	100.2	822	8.4E-238
SPV_cluster_338	Type I PKS	AAU04878	BGC0000365	Glycopeptidolipid	44	88.2	554	2.5E-157
SPV_cluster_347	Type I PKS	AAT70105	BGC0001165	Curacin	36	101.4	773	3.8E-223
SPV_cluster_348	Type I PKS	AIW82279	BGC0001125	Puwainaphycins	36	101.9	788	1.5E-227
SPV_cluster_356	Type I PKS	AGC45622	BGC0001394	Phenalamide	34	90.1	534	4.8E-151
SPV_cluster_357	Type I PKS	CAQ18829	BGC0000954	Ajudazol	44	84.4	565	1.4E-160
SPV_cluster_360	Type I PKS	AAU04878	BGC0000365	Glycopeptidolipid	49	101.3	527	2.8E-149
SPV_cluster_368	Type I PKS	BAG17643	BGC0001043	SGR_PTMs	34	97.2	505	1.2E-142
SPV_cluster_371	Type I PKS	AAU04878	BGC0000365	Glycopeptidolipid	44	101.3	756	3.6E-218
SPV_cluster_373	Type I PKS	AAU04878	BGC0000365	Glycopeptidolipid	47	82.7	493	3.2E-139
SPV_cluster_377	Type I PKS	CAQ18834	BGC0000954	Ajudazol	45	104.0	716	4.1E-206
SPV_cluster_378	Type I PKS	CAQ18828	BGC0000954	Ajudazol	42	101.1	668	9.8E-192
SPV_cluster_382	Type I PKS	CAQ18828	BGC0000954	Ajudazol	42	99.5	631	2.2E-180
SPV_cluster_384	Type I PKS	AAU04878	BGC0000365	Glycopeptidolipid	45	79.5	448	1.5E-125
SPV_cluster_386	Type I PKS	BAG17643	BGC0001043	SGR_PTMs	34	91.2	373	7.8E-103
SPV_cluster_391	Type I PKS	BAG17643	BGC0001043	SGR_PTMs	32	88.5	344	4.9E-94
SPV_cluster_394	Type I PKS	CAQ18829	BGC0000954	Ajudazol	45	96.7	588	1.5E-167
SPV_cluster_396	Type I PKS	CAQ18828	BGC0000954	Ajudazol	45	101.1	673	2.6E-193
SPV_cluster_405	Type I PKS	CAQ18828	BGC0000954	Ajudazol	43	101.3	599	7.4E-171
SPV_cluster_412	Type I PKS	CBD77732	BGC0000974	Crocacin	38	101.3	446	7.8E-125
SPV_cluster_413	Type I PKS	AGC45620	BGC0001394	Phenalamide	47	89.6	551	1.3E-156
SPV_cluster_418	Type I PKS	AGC45620	BGC0001394	Phenalamide	46	86.7	480	3.5E-135
SPV_cluster_421	Type I PKS	AAF19810	BGC0001024	Myxothiazol	40	101.9	418	1.5E-116
SPV_cluster_422	Type I PKS	CAQ18834	BGC0000954	Ajudazol	42	97.6	464	1.8E-130
SPV_cluster_425	Type I PKS	CAQ18829	BGC0000954	Ajudazol	49	100.3	549	5.2E-156

SPV_cluster_426	Type I PKS	CBD77732	BGC0000974	Crocacin	39	102.0	366	6.2E-101
SPV_cluster_428	Type I PKS	AAU04878	BGC0000365	Glycopeptidolipid	51	100.0	527	2.1E-149
SPV_cluster_440	Type I PKS	AAK57186	BGC0001022	Myxalamid	46	100.8	397	2.1E-110
SPV_cluster_446	Type I PKS	CAD19092	BGC0000153	Stigmatellin	43	101.1	311	2.3E-84
SPV_cluster_447	Type I PKS	CAQ18828	BGC0000954	Ajudazol	46	101.1	342	5.4E-94
SPV_cluster_448	Type I PKS	AIW82282	BGC0001125	Puwainaphycins	38	101.8	296	5.8E-80
SPV_cluster_450	Type I PKS	CAQ18834	BGC0000954	Ajudazol	42	104.3	284	2.2E-76
SPV_cluster_451	Type I PKS	AAU04878	BGC0000365	Glycopeptidolipid	45	100.5	325	1.1E-88
SPV_cluster_454	Type I PKS	CBD77732	BGC0000974	Crocacin	41	100.2	270	4.1E-72
SPV_cluster_455	Type I PKS	CAQ18834	BGC0000954	Ajudazol	48	101.7	338	9.2E-93
SPV_cluster_456	Type I PKS	CAD19092	BGC0000153	Stigmatellin	45	100.5	299	6.2E-81
SPV_cluster_457	Type I PKS	CAQ18828	BGC0000954	Ajudazol	37	98.8	220	2.8E-57
SPV_cluster_459	Type I PKS	CAQ18828	BGC0000954	Ajudazol	45	100.3	289	8.2E-78
SPV_cluster_380	Type II PKS	AHZ61892	BGC0000240	Lomaiviticin	30	100.0	136	7.1E-32
SPV_cluster_090	Type III PKS	BAG17301	BGC0000282	Alkylresorcinol	39	99.1	228	8.9E-60
SPV_cluster_131	Type III PKS	BAG17301	BGC0000282	Alkylresorcinol	37	99.7	213	3.0E-55
SPV_cluster_218	Type III PKS	BAG17301	BGC0000282	Alkylresorcinol	31	97.5	193	3.3E-49
SPV_cluster_404	Type III PKS	BAG17301	BGC0000282	Alkylresorcinol	40	97.2	233	3.7E-61
SPV_cluster_460	Type III PKS	BAG17301	BGC0000282	Alkylresorcinol	39	96.4	224	2.3E-58
